# Stereoselective Synthesis of Mechanically Planar Chiral Rotaxanes

**DOI:** 10.1002/anie.201808990

**Published:** 2018-10-17

**Authors:** Michael A. Jinks, Alberto de Juan, Mathieu Denis, Catherine J. Fletcher, Marzia Galli, Ellen M. G. Jamieson, Florian Modicom, Zhihui Zhang, Stephen M. Goldup

**Affiliations:** ^1^ Chemistry University of Southampton, Highfield Southampton SO17 1BJ UK

**Keywords:** chirality, CuAAC, rotaxane, stereoselective synthesis, supramolecular chemistry

## Abstract

Chiral interlocked molecules in which the mechanical bond provides the sole stereogenic unit are typically produced with no control over the mechanical stereochemistry. Here we report a stereoselective approach to mechanically planar chiral rotaxanes in up to 98:2 *d.r*. using a readily available α‐amino acid‐derived azide. Symmetrization of the covalent stereocenter yields a rotaxane in which the mechanical bond provides the only stereogenic element.

Chiral molecules occupy a central role in chemistry due to their applications across a range of areas and thus the development of efficient, stereoselective syntheses of these targets is a central challenge for synthetic chemists.^1^, ^2^ In contrast, although mechanically interlocked molecules (MIMs)^3^ have long been known^4^, ^5^ to display stereogenic units^6^ as a result of the fixed relative orientation of achiral interlocked components,^7^, ^8^ or the topology of the mechanical bond itself,^9^, ^10^ the stereoselective synthesis of rotaxanes and catenanes exhibiting such stereochemistry remains largely unexplored,^11^ with the vast majority reported as racemic mixtures, or separated using preparative chiral stationary phase HPLC (PCSP‐HPLC).^12^

The development of stereoselective syntheses of mechanically planar chiral (MPC) rotaxanes, the stereogenic unit of which arises when a rotationally oriented macrocycle encircles a non‐centrosymmetric axle, has proved challenging. Takata, Okamoto and co‐workers reported an enantioselective synthesis of an MPC rotaxane by dynamic kinetic resolution in ≈4 % *e.e*.7e Lacour and co‐workers reported the diastereoselective formation of a pseudorotaxane possessing a covalent and an MPC stereogenic element in ≈8 % *d.e*.^13^ Indeed, the only highly stereoselective syntheses of MPC rotaxanes are not widely recognized as such;5c the threading of a cyclodextrin (CD) ring onto a non‐centrosymmetric axle produces an MPC stereogenic element. Thus, the selective formation of the different threading orientations corresponding to the stereoselective synthesis of MPC/covalent diastereomers.^14^ Unfortunately, the dense array of covalent stereogenic centers of the glucose‐derived CD macrocycle obscures the role of the MPC stereogenic element in the properties of these products.

Despite the lack of a general stereoselective approach to MPC rotaxanes, limited intriguing reports of the properties and potential applications of the MPC stereogenic unit have been disclosed.^15^ Vögtle, Okamoto, and co‐workers demonstrated that MPC rotaxanes display large Cotton effects, suggestive of a well‐expressed chiral environment.7a–7c More recently, Takata and co‐workers demonstrated that side chains containing MPC rotaxane units can direct the handedness of a helical polymer,7f and Hirose and co‐workers reported an MPC rotaxane that acts as a selective receptor for chiral analytes.7g, 16

In 2014 we reported that, by including a covalent point stereogenic unit in the axle of a crowded rotaxane, the mechanical epimers of the product could be separated using flash chromatography.^17^ The separated diastereomers were then converted to enantiopure MPC rotaxanes by removing the covalent stereogenic unit. However, although this approach allows the scalable synthesis of MPC enantiomers, its efficiency was reduced by a lack of stereoselectivity (*d.r*.=1:1) in the mechanical bond forming step.

Here we report a diastereoselective synthesis of MPC rotaxanes using a simple covalent stereodirecting moiety. Furthermore, by symmetrization of the covalent stereocenter, we demonstrate the first stereoselective synthesis of an MPC rotaxane where the mechanical bond provides the only stereogenic unit.

We previously reported that an active template^18^ Cu‐mediated alkyne–azide cycloaddition^19^ (AT‐CuAAC)^20^ reaction between small bipyridine macrocycle **1**,^21^ alkyne **2 a** and chiral azide (d)‐**3 a** gave the rotaxane product as an equimolar mixture of mechanical epimers (Table 1, entry 1) and that variation of reaction conditions failed to impart diastereoselectivity.^17^, ^22^ We hypothesized that placing the stereochemical information on the alkyne component might lead to selectivity in the formation of diastereomeric Cu^I^‐acetylide/macrocycle complexes in a biased pre‐equilibrium.^23^, ^24^ Unfortunately, the reaction of alkyne (d)‐**2 b** with azide **3 b** also produced an equimolar mixture of diastereomers (entry 2).

**Table 1:** Diastereoselectivity in the AT‐CuAAC reaction of macrocycle **1** with alkynes **2** and azides **3**.^[a]^ 
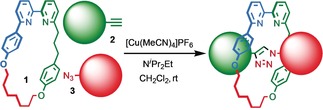

Entry	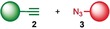	*d.r*.^[b]^ (yield)^[c]^
1	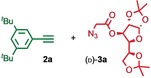	50:50
2	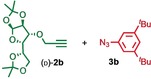	50:50 (60 %)
3	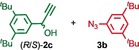	65:35 (40 %)
4	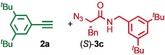	85:15 (84 %)
5	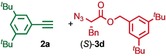	96:4 (64 %)
6	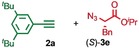	98:2 (88 %)
7	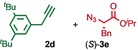	86:14 (80 %)
8	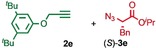	75:25 (93 %)
9	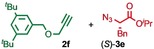	72:28 (64 %)
10	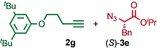	75:25 (67 %)
11	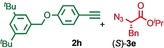	84:16 (76 %)

[a] Reagents and conditions: **1**, 1.5 equiv each **2** and **3**, 0.96 equiv [Cu(MeCN)_4_]PF_6_,^29^ N^*i*^Pr_2_Et, CH_2_Cl_2_, RT. [b] Determined by ^1^H NMR analysis of the crude reaction product. [c] Yield of isolated product.^30^

Moving the stereogenic unit closer to the reaction center improved the outcome; when α‐chiral acetylene (*R*/*S*)‐**2 c** was employed, a 65:35 *d.r*. was observed (entry 3). More promising still, when azide (*S*)‐**3 c** was employed a 85:15 *d.r*. was obtained (entry 4). Focusing on the readily available amino acid‐derived azide motif,^25^, ^26^ ester (*S*)‐**3 d** led to an excellent 96:4 *d.r*. (entry 5). Finally, simple ^*i*^Pr ester‐azide (*S*)‐**3 e** provided the corresponding [2]rotaxane in an excellent 98:2 *d.r*. and 88 % yield (entry 6).^27^ Single crystal X‐ray diffraction analysis of the interlocked product of macrocycle **1**, alkyne **2 a** and azide (*S*)‐**3 e** allowed the major product to be unambiguously assigned as (*S*,*S*_mp_)‐**4** (Figure 1, Scheme 1).^28^

**Figure 1:**
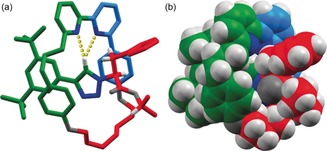
Solid‐state structure of [2]rotaxane (*S*,*S*_mp_)‐**4** in a) tube and b) space‐filling representation (O and N atoms in dark grey and blue, respectively). Selected distances [Å]: H_*h*_⋅⋅⋅N=2.47; H_*h*_⋅⋅⋅N=2.69).

**Scheme 1:**
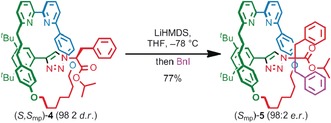
Synthesis of MPC rotaxane (*S*_mp_)‐**5** by symmetrization of the covalent stereocenter. [a] Reagents and conditions: LiHMDS, THF, −78 °C, BnI, −78 °C to RT.

Preliminary molecular modelling (PM6, gas phase; see the Supporting Information for a detailed discussion) suggests that the observed stereoselectivity is due to steric clash between the macrocycle and the Bn group of the azide component, with the favored isomer minimizing the interaction between the Bn and aryl‐pyridine units. The observed stereoselectivity is predicted to be due both to biasing of the equilibrium between diastereomeric macrocycle‐bound Cu^I^‐acetylide/azide complexes, and the different rates of the cycloaddition process that captures the interlocked structure from these diastereomeric intermediates.

Having identified (*S*)‐**3 e** as able to direct the diastereoselective formation of MPC rotaxanes, we investigated the generality of the reaction with respect to the alkyne. Benzylic acetylene **2 d** coupled with (*S*)‐**3 e** in the presence of **1** to give the corresponding rotaxane in 86:14 d.r. (entry 7). The reactions of (*S*)‐**3 e** with alkynes **2 e**–**g**, which provide decreased steric demand near the reaction centre, all produced the corresponding rotaxanes in a low but synthetically useful ≈3:1 *d.r*. (entries 8–10). These results suggest that AT‐CuAAC reactions of azide (*S*)‐**3 e** with macrocycle **1** have an inherent diastereoselectivity that is enhanced by steric crowding provided by the alkyne component. Consistent with this, extended aryl alkyne **2 h** gave an improved 84:16 *d.r*. (entry 11).

Highly enantioenriched mixed covalent/mechanical diastereomers such as (*S*,*S*_mp_)‐**4** are suitable for investigation in areas such as catalysis and sensing. However, in order to unambiguously identify the effect of the MPC stereogenic element it is necessary to produce rotaxanes in which the mechanical bond provides the sole source of stereochemical information. To demonstrate the utility of azide **3 e** in the stereoselective synthesis of such enantioenriched “simple” MPC rotaxanes, the covalent stereocentre of rotaxane (*S*,*S*_mp_)‐**4** was symmetrized by treatment with LiHMDS followed by BnI to yield MPC rotaxane (*S*_mp_)‐**5** in excellent yield (77 %) and enantiomeric purity (98:2 *e.r*.) (Scheme 1). Similarly, alkylation of (*R*,*R*_mp_)‐**4**, derived from azide (*R*)‐**3 e**, gave (*R*_mp_)‐**5** (98:2 *e.r*.).^31^, ^32^ Analytical CSP‐HPLC analysis established the enantiopurity of (*S*_mp_)‐**5** and (*R_mp_*)‐**5** to be 98:2 *e.r*. in keeping with the diastereomeric purity of the starting materials (Figure 2 a). Analysis of (*S*_mp_)‐**5** and (*R*_mp_)‐**5** by circular dichroism (CD) spectroscopy revealed mirror‐image Cotton effects (Figure 2 b).

**Figure 2:**
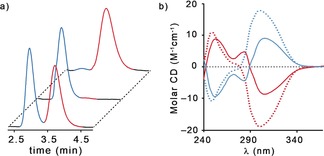
a) CSP‐HPLC (Chiralpak, 35 % MeOH (0.2 % NH_3_) in CO_2_, 4 mL min^−1^), of (front to back) (*R*_mp_/*S*_mp_)‐**5**,^33^ (*S*_mp_)‐**5** and (*R*_mp_)‐**5**. b) CD spectra of (*R*,*R*_mp_)‐**4** (red dashed), (*S*,*S*_mp_)‐**4** (blue dashed), (*R*_mp_)‐**5** (red), (*S*_mp_)‐**5** (blue).

In conclusion, we have demonstrated that azide **3 e** is able to direct a diastereoselective AT‐CuAAC reaction to deliver mechanically epimeric rotaxanes in up to 98:2 *d.r*. Furthermore, by symmetrization of the covalent stereocenter, our approach can be extended to give highly enantioenriched MPC rotaxanes in which the mechanical bond is the sole stereogenic element without the need to separate rotaxane stereoisomers.^30^ Given the synthetic flexibility of the AT‐CuAAC reaction, which allows the expedient synthesis of complex architectures^34^ and functional MIMs,^35^ we anticipate that this general approach will be useful for the stereoselective synthesis of functional MPC rotaxanes and other MIM stereogenic units.

## Conflict of interest

The authors declare no conflict of interest.

## Supporting information

As a service to our authors and readers, this journal provides supporting information supplied by the authors. Such materials are peer reviewed and may be re‐organized for online delivery, but are not copy‐edited or typeset. Technical support issues arising from supporting information (other than missing files) should be addressed to the authors.

Supplementary
