# Supplementary material for: Stereoselective Synthesis of Mechanically Planar Chiral Rotaxanes
Source: Angew Chem Int Ed Engl. 2018 Oct 17;57(45):14806–10. doi: 10.1002/anie.201808990 (PMC6220991; doi:10.1002/anie.201808990)
Supplement: Supplementary file 1 — Supplementary [file ANIE-57-14806-s001.pdf]

Angewandte Chemie
Eine Zeitschrift der Gesellschaft Deutscher Chemiker

Supporting Information

Stereoselective Synthesis of Mechanically Planar Chiral Rotaxanes

*Michael A. Jinks[+], Alberto de Juan[+], Mathieu Denis, Catherine J. Fletcher, Marzia Galli, Ellen M. G. Jamieson, Florian Modicom, Zhihui Zhang, and Stephen M. Goldup**

anie_201808990_sm_miscellaneous_information.pdf

Table Of Contents

1. General experimental procedures 2
2. Synthesis and characterisation of alkynes 2 and azides 3 4
3. Synthesis and characterisation of axles and rotaxanes (table 1) 26
Entry 1 – axle and rotaxanes derived from alkyne 2a and azide 3a 26
Entry 2 – axle S12 and rotaxanes S13 derived from alkyne 2b and azide 3b 26
Entry 3 – axle (*R*/*S*)-S14 and rotaxane (*R*/*S*)-S15 derived from alkyne 2c and azide 3b 33
Entry 4 – axle (*S*)-S16 and rotaxane (S,R_{mp}/S_{mp})-S17 derived from alkyne 2a and azide 3c 39
Entry 5 – axle (*S*)-S18 and rotaxanes (S,R_{mp}/S_{mp})-S19 derived from alkyne 2b and azide (*S*)-3b 47
Entry 6 – axle S20 and rotaxane 4 derived from alkyne 2a and azide 3e 55
Entry 7 – axle (*S*)-S21 and rotaxane S22 derived from alkyne 2d and azide (*S*)-3e 73
Entry 8 – axle (*S*)-S23 and rotaxane (S,R_{mp}/S_{mp})-S24 derived from alkyne 2e and azide (*S*)-3e 80
Entry 9 – axle (*S*)-S25 and rotaxane (S,R_{mp}/S_{mp})-S26 derived from alkyne 2f and azide (*S*)-3e 87
Entry 10 – axle (*S*)-S27 and rotaxanes (S,R_{mp}/S_{mp})-S28 derived from alkyne 2g and azide (*S*)-3e 93
Entry 11 – axle (*S*)-S29 and rotaxane (S,R_{mp}/S_{mp})-S30 derived from alkyne 2h and azide (*S*)-3e 100
4. Alkylation of axle S20 and rotaxanes 4 107
5. Comparative ^{1}H-NMR stack plots of axles and rotaxanes 118
6. Comparative circular dichroism spectra 122
7. Single crystal X-ray crystallographic data of (S,S_{mp})-4 124
8. Synthesis of enantiopure rotaxane (R_{mp})-5 125
9. Racemisation of axle (*S*)-S20 under CuAAc conditions 128
10. Diastereoselective Alkylation of Rotaxanes (S,S_{mp})-4 131
11. Preliminary Molecular Modelling 136
12. References 141

1. General experimental procedures

Unless otherwise stated, all reagents were purchased from commercial sources (Sigma Aldrich, Fisher Scientific, Alfa Aesar, Acros and Fluorochem) and used without further purification. $[Cu(MeCN)_4][PF_6]$ was prepared as described by Pigorsch and Köckerling.[1] Anhydrous solvents were purchased from Acros. Experiments carried out in sealed vessels were performed in CEM microwave vials, with crimped caps, with PTFE septa. Flash column chromatography was performed using Biotage Isolera-4 or Isolera-1 automated chromatography system, employing Biotage SNAP or ZIP cartridges (50 μm, irregular silica, default flow rates). All azide waste was disposed of as described by Gardiner and co-workers.[2]

Petrol refers to the fraction of petroleum ether boiling in the range 40-60 °C. IPA refers to isopropyl alcohol. DMF refers to *N,N*-dimethylformamide. DMAP refers to *N,N*-dimethylaminopyridine. TFA refers to trifluoroacetic acid. THF refers to tetrahydrofuran. DIPEA refers to *N,N*-diisopropylethylamine. Analytical TLC was performed on pre-coated silica gel plates on aluminum (0.25 mm thick, 60F254, Merck, Germany) and observed under UV light (254 nm). EDTA-NH_3 solution refers to an aqueous solution of NH_3 (17% w/w) saturated with sodium-ethylenediaminetetraacetate.

NMR spectra were recorded on Bruker AV400 or AV500 instrument, at a constant temperature of 298 K. Chemical shifts are reported in parts per million from low to high field and referenced to residual solvent. Coupling constants (*J*) are reported in Hertz (Hz). Standard abbreviations indicating multiplicity were used as follows: m = multiplet, quint = quintet, q = quartet, t = triplet, d = doublet, s = singlet, app. = apparent, br = broad, sept = septet. Signal assignment was carried out using 2D NMR methods (HSQC, HMBC, COSY, NOESY or TOCSY) where necessary. In the case of some complex multiplets with contributions from more than proton signals, such as diastereoisomers, exact assignment was not possible. Here indicative either/or assignments (e.g. H_A or H_B) are provided. For clarity all proton signals corresponding to the thread components are in lower case, and all proton signals corresponding to the macrocycle components are in upper case. Exact assignment of each diastereoisomer is not possible without an X-ray crystal structure, so distinguishing between different diastereoisomers is either as H_X or $H_{X'}$ or as H_X (*major*) or H_X (*minor*).

Low resolution mass spectrometry was carried out by the mass spectrometry services at University of Southampton (Waters TQD mass spectrometer equipped with a triple quadrupole analyser with UHPLC injection [BEH C_{18} column; MeCN-H_2O gradient {0.2% formic acid}]). High resolution mass spectrometry was carried out either by the mass spectrometry service at the University of Edinburgh (ThermoElectron MAT 900) or by the mass spectrometry services at the University of Southampton (MaXis, Bruker Daltonics, with a Time of Flight (TOF) analyser; samples were introduced to the mass spectrometer *via* a Dionex Ultimate 3000 autosampler and uHPLC pump in a gradient of 20% MeCN in hexane to 100% acetonitrile (0.2% formic acid) over 5-10 min at 0.6 mL/min; column: Acquity UPLC BEH C18 (Waters) 1.7 micron 50 × 2.1mm).

Chiral stationary phase SCFC was carried out by Reach Separations, based in Nottingham. Circular dichroism spectra were acquired on an Applied Photo-physics Chirascan spectropolarimeter, recorded using Applied Photophysics software Ver. 4.2.0 in dried spectroscopic grade $CHCl_3$, following overnight desiccation, at a concentration range of 0.5-100 10^{-4} M, in a quartz cell of 1 cm path length, at a constant temperature of 293 K.

The following compounds were synthesised according to literature procedures;
Macrocycle **1**,[3] **2a**,[4] , **2b**,[5] **3b**,[6] **S1**,[7] **S2**,[8] **S3**,[9] **S4**,[10] **S5**,[11] **S6**,[12] (*S*)-**S7**,[13] (*R*)-**S7**,[14](*R/S*)-**S7**,[14] **S8**[15], Macrocycle **S9**,[3] and **S32**.[16] Diazo-transfer reagent **S10** was purchased from Sigma Aldrich and stored under an inert atmosphere at -20 °C.[17]

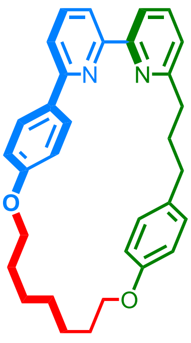

1

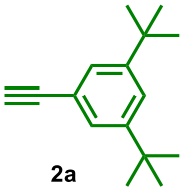

2a

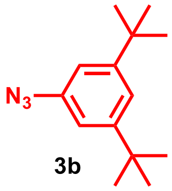

3b

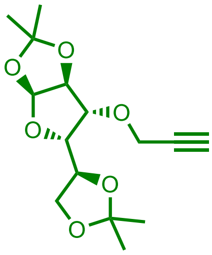

2b

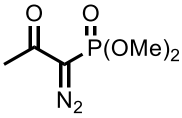

S1

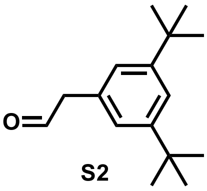

S2

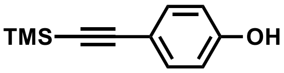

S3

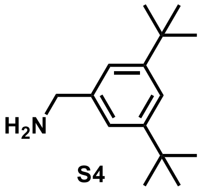

S4

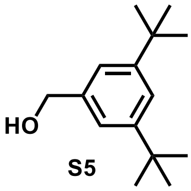

S5

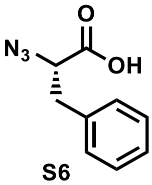

S6

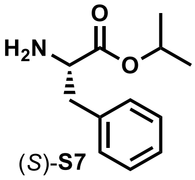

(*S*)-**S7**

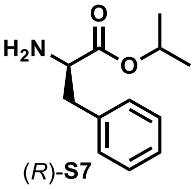

(*R*)-**S7**

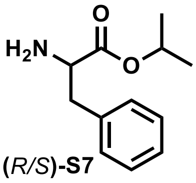

(*R/S*)-**S7**

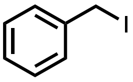

S8

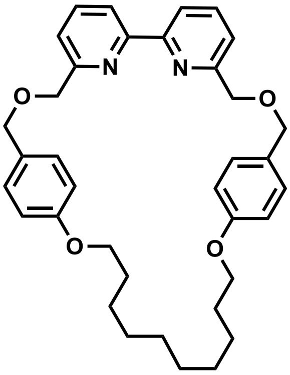

S9

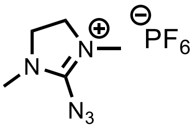

S10

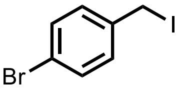

S32

2. Synthesis and characterisation of alkynes 2 and azides 3

(*R*/*S*)-1-(3,5-Di-*tert*-butylphenyl)prop-2-yn-1-ol ((*R*/*S*)-**2c**)

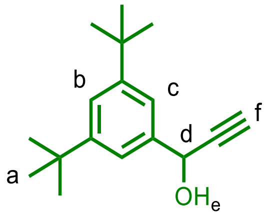

A 50 mL round bottomed flask was charged with 3,5-di-*tert*-butylbenzaldehyde (1.40 g, 6.40 mmol), which was dissolved in anhydrous THF (20 mL) at 0 °C, protected by a nitrogen atmosphere. A solution of ethynylmagnesium chloride (0.60 M, 10.0 mmol, 16 mL) was added dropwise. The mixture was stirred for 20 h at rt. A saturated aqueous NH_4Cl solution (10 mL) was added. The organic solvent was removed *in vacuo*. The residue was extracted with Et_2O (3 ×20 mL). The combined organic layers were washed with brine (10 mL), dried over $MgSO_4$, filtered, and the solvent removed *in vacuo*. The residue was purified by column chromatography (isocratic 10% EtOAc in petrol) to afford alkyne **2c** as a light-yellow oil (1.50 g, 97%). ^{1}H NMR (400 MHz, $CDCl_3$, 298 K) δ 7.33-7.28 (m, 3H, H_b and H_c), 5.31 (d, 1H, *J* = 2.2, H_d), 2.52 (d, 1H, *J* = 2.2, H_f), 2.51-2.39 (br s, 1H, H_e), 1.34 (s, 18H, H_a); ^{13}C NMR (101 MHz, $CDCl_3$, 298 K) δ 151.2, 139.3, 122.7, 121.0, 84.1, 74.7, 65.1, 35.0, 31.6; HR-EI-MS (+ve) *m/z* = 244.18118 [M^+] calc. 244.18217.

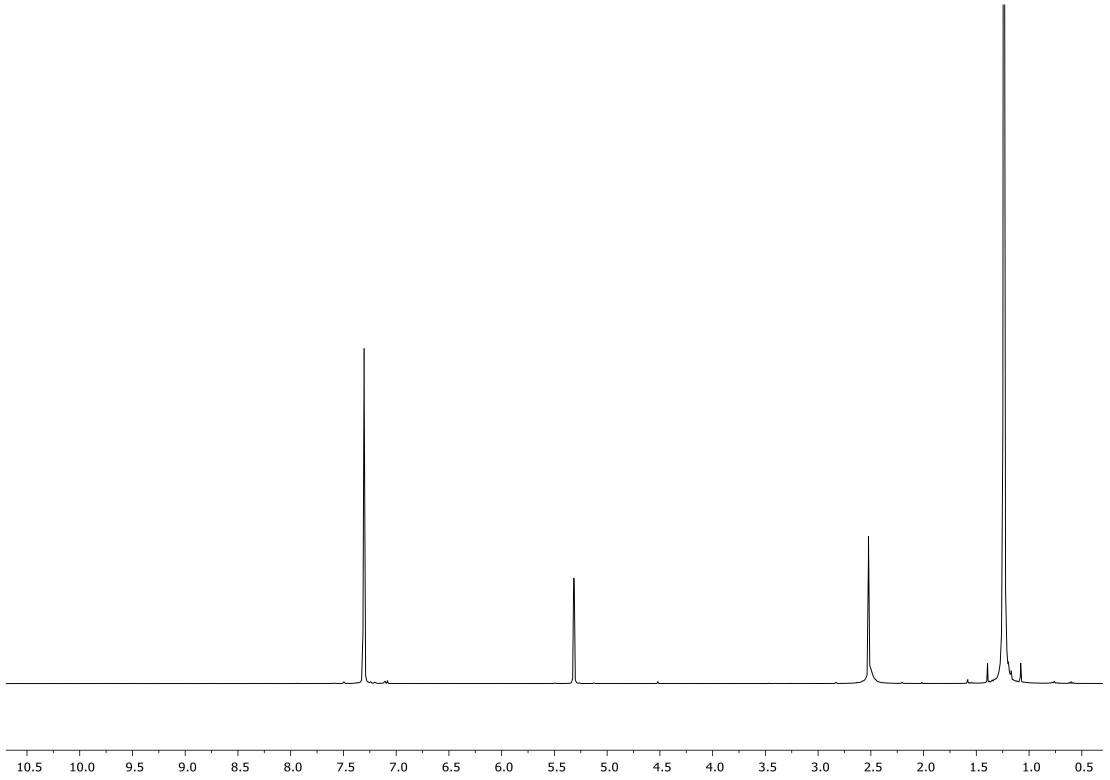

Figure S1. ^{1}H NMR (400 MHz, $CDCl_3$, 298 K) (*R*/*S*)-**2c**.

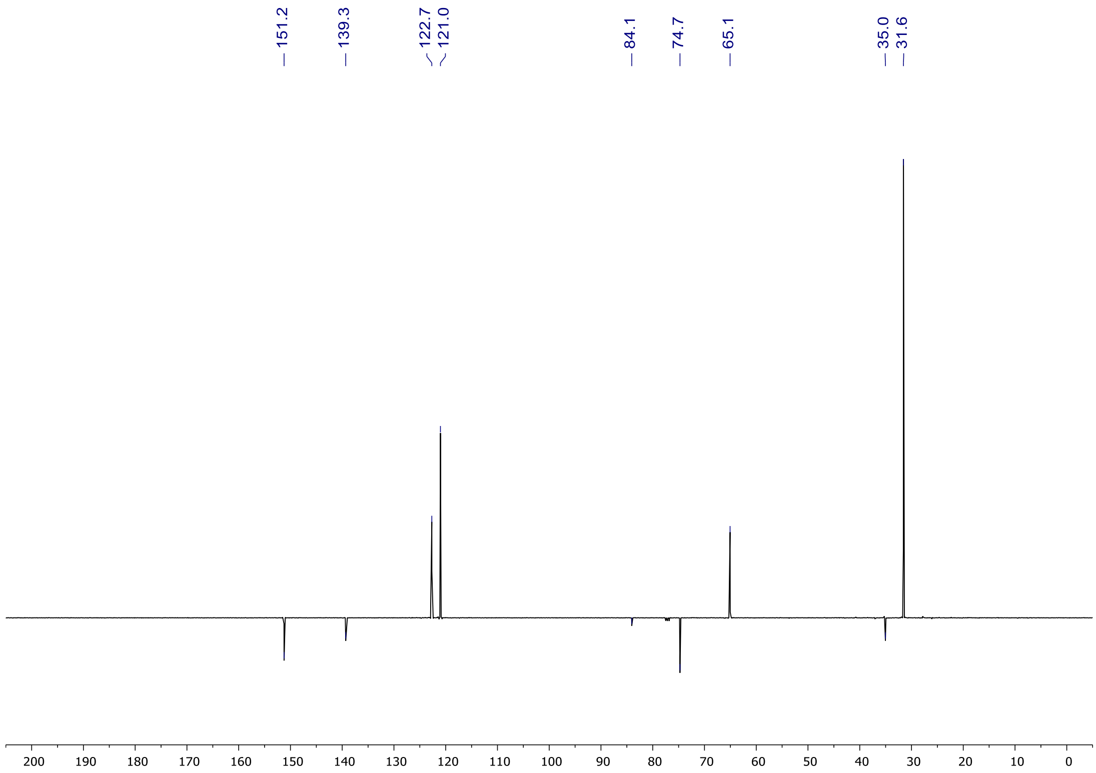

Figure S2. ^{13}C NMR (101 MHz, $CDCl_3$, 298 K) (*R*/*S*)-**2c**.

1,3-Di-*tert*-butyl-5-(prop-2-yn-1-yl)benzene (**2d**)

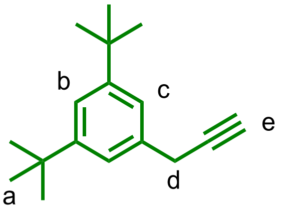

A 10 mL round bottomed flask was charged with **S1** (192 mg, 1.00 mmol), **S2** (200 mg, 0.86 mmol), K_2CO_3 (235 mg, 1.70 mmol) and MeOH (2 mL) and the mixture stirred for 19 h at rt. The mixture was diluted with CH_2Cl_2 (10 mL) and filtered,. The filtrate was washed with H_2O (5 mL) and brine (5 mL). The combined aqueous layers were extracted with CH_2Cl_2 (10 mL). The combined organic layers were dried over $MgSO_4$, filtered, and the solvent removed *in vacuo*. The residue was purified by column chromatography (isocratic 100% petrol) to afford alkyne **2d** as a colorless oil (160 mg, 81%); ^{1}H NMR (400 MHz, $CDCl_3$, 298 K) δ 7.31 (t, 1H, *J* = 1.8, H_b), 7.20 (d, 2H, *J* = 1.8, H_c), 3.60 (d, 2H, *J* = 2.6, H_d), 2.18 (t, 1H, *J* = 2.6, H_e), 1.33 (s, 18H, H_a); ^{13}C NMR (101 MHz, $CDCl_3$, 298 K) δ 151.2, 135.2, 122.3, 120.9, 82.6, 70.4, 35.0, 31.6, 25.3; HR-EI-MS (+ve) *m/z* = 228.18696 [M^+] calc. 228.18725.

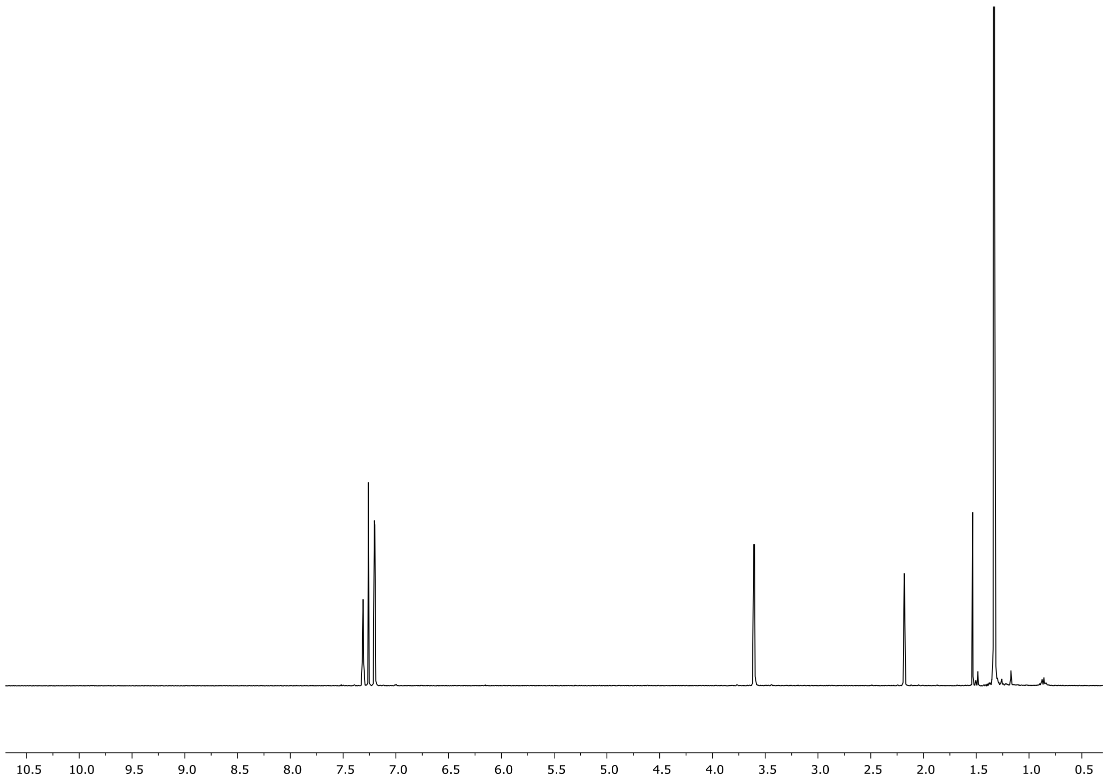

Figure S3. ^{1}H NMR (400 MHz, CDCl3, 298 K) **2d**.

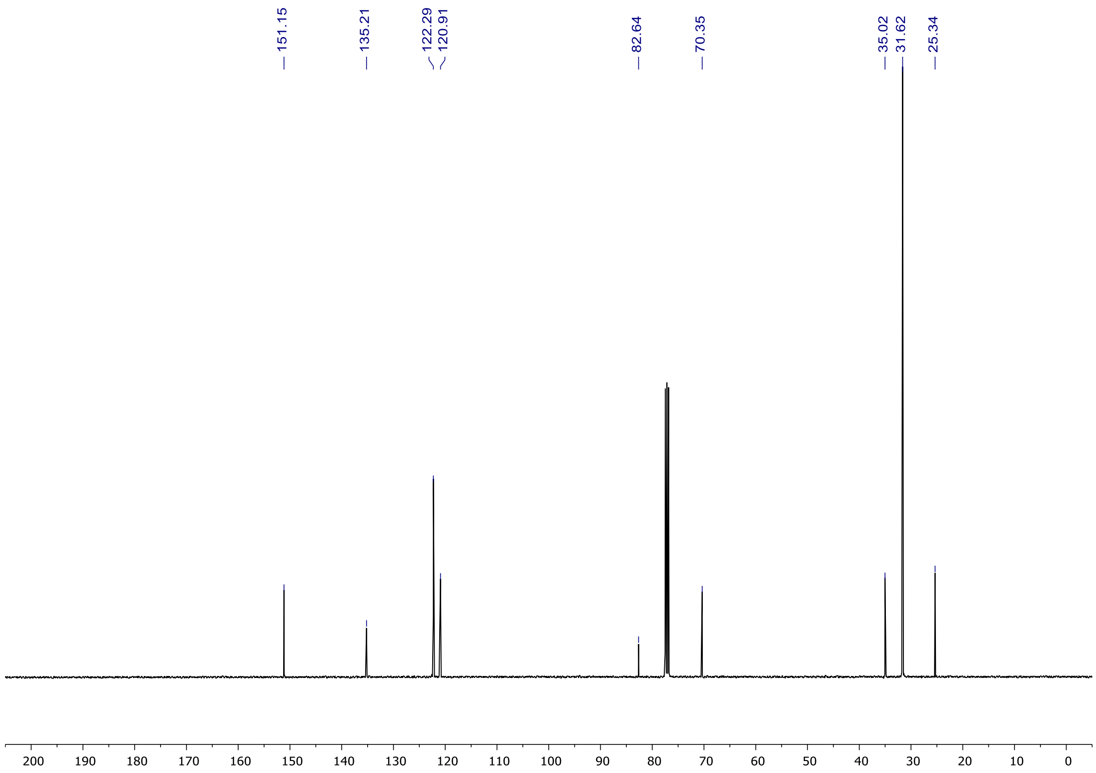

Figure S4. ^{13}C NMR (101 MHz, CDCl3, 298 K, 298 K) **2d**.

1,3-Di-*tert*-butyl-5-(prop-2-yn-1-yloxy)benzene (**2e**)

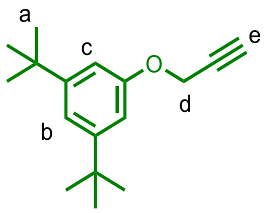

A 10 mL round bottomed flask was charged with 3,5-di-*tert*-butylphenol (231 mg, 1.12 mmol), propargyl bromide (0.40 mL of a 80% wgt solution in toluene), K_2CO_3 (424 mg, 3.36 mmol), and DMF (3 mL) and the mixture stirred at 70 °C for 16 h. After cooling to rt, H_2O (50 mL) was added, and the mixture was extracted with EtOAc (3 × 30 mL). The combined organic extracts were washed with 5% w/v LiCl (5 × 30 mL), brine (30 mL), were dried over $MgSO_4$, filtered, and the solvent removed *in vacuo*, to yield alkyne **2e** as a red oil (255 mg, 93%). No further purification was required; ^1H NMR (400 MHz, CDCl3, 298 K) δ 7.10 (t, 1H, *J* = 1.6, H_b), 6.88 (d, 2H, *J* = 1.6, H_c), 4.72 (d, 2H, *J* = 2.3, H_d), 2.54 (t, 1H, *J* = 2.3, H_e), 1.35 (s, 18H, H_a); ^{13}C NMR (101 MHz, CDCl3, 298 K) δ 157.3, 152.4, 115.9, 109.4, 79.1, 75.4, 55.9, 35.2, 31.6; LR-EI-MS (+ve) *m/z* (%) = 229.2 [M – CH_3]$^+$ (90) 244.4 [M$^+$] (58); HR-EI-MS (+ve) *m/z* = 244.18278 [M$^+$] calc. 244.18217.

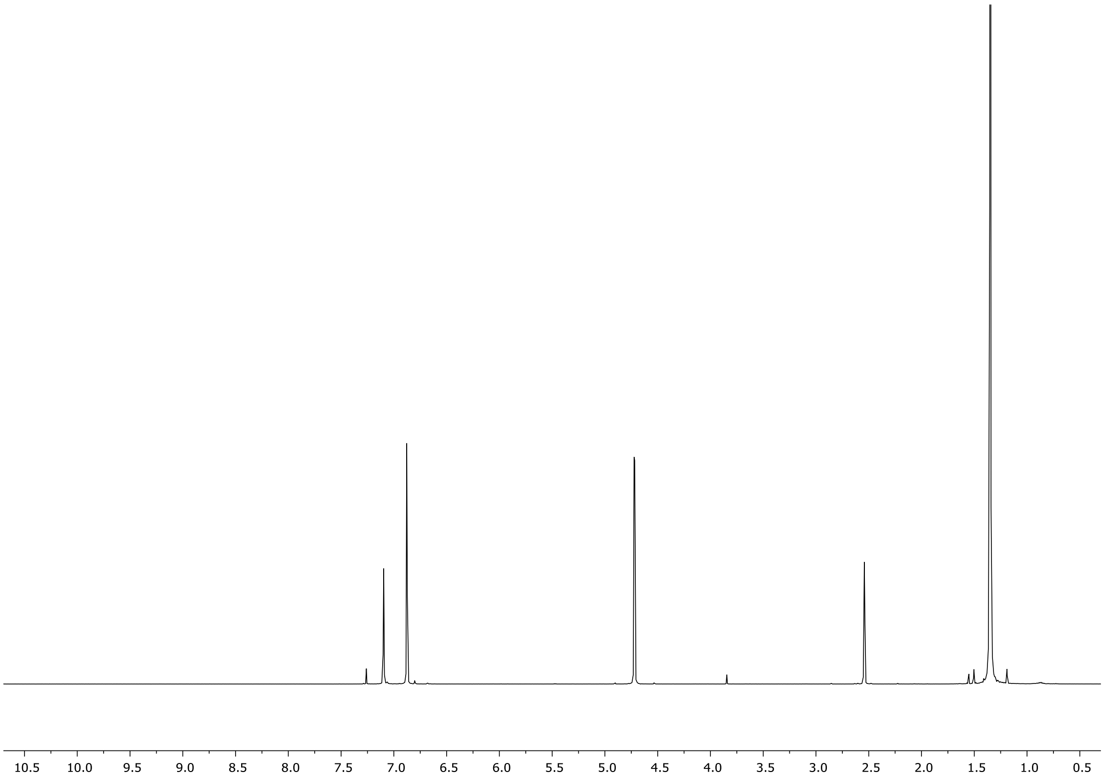

Figure S5. ^1H NMR (400 MHz, CDCl3, 298 K) **2e**.

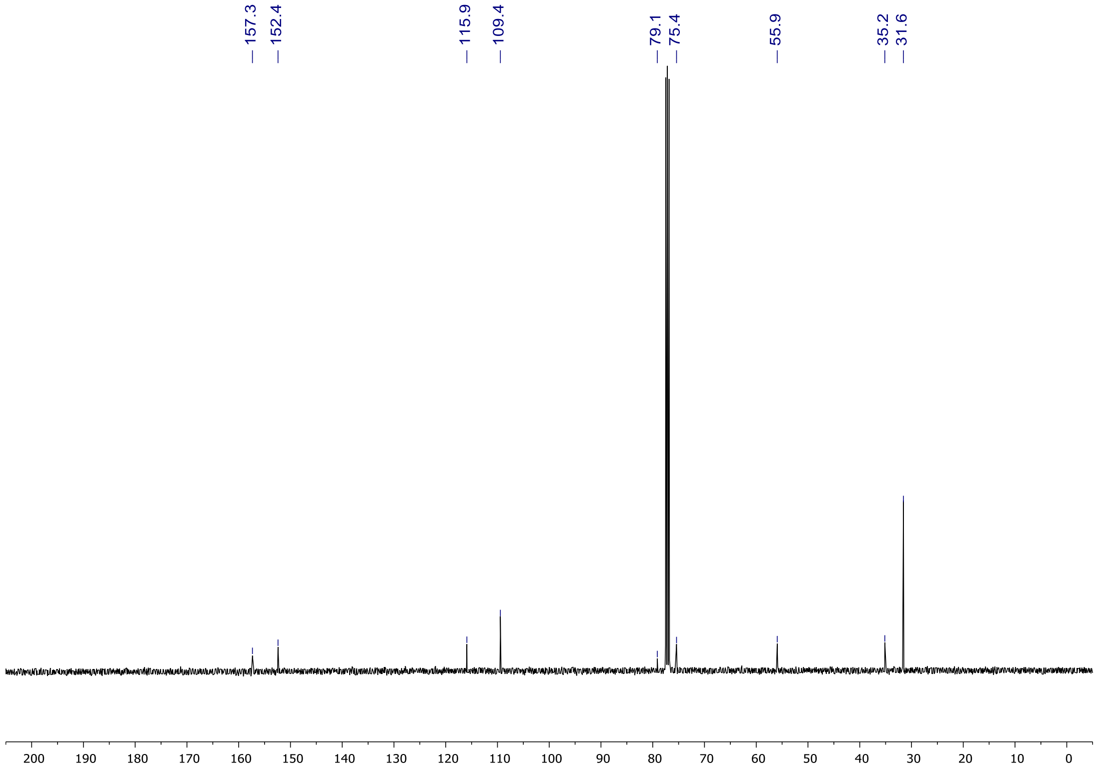

Figure S6. ^{13}C NMR (101 MHz, $CDCl_3$, 298 K) **2e**.

1,3-Di-*tert*-butyl-5-((prop-2-yn-1-yloxy)methyl)benzene (**2f**)

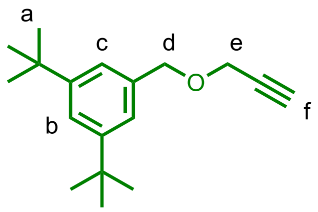

A 25 mL round bottomed flask was charged with NaH (1.21 g of a 60% wgt dispersion in mineral oil), propargyl alcohol (4.9 mL, 84.2 mmol), and anhydrous DMF (3 mL) and the mixture was stirred at 0 °C for 30 minutes, protected by an argon atmosphere. 3,5-Di-*tert*-butylbenzylbromide (403 mg, 1.43 mmol) in anhydrous DMF (3 mL) was added, and the reaction mixture was stirred with warming to rt for 16 h. H_2O (50 mL) was added slowly, and the mixture was extracted with EtOAc (2 × 50 mL). The combined organic extracts were washed with 5% w/v LiCl (5 × 50 mL), brine (50 mL), were dried over $MgSO_4$, filtered, and the solvent removed *in vacuo*, to yield alkyne **2f** as an orange oil (369 mg, quant.); ^{1}H NMR (400 MHz, $CDCl_3$, 298 K) δ 7.37 (t, 1H, *J* = 1.6, H_b), 7.20 (d, 2H, *J* = 1.6, H_c), 4.60 (s, 1H, H_d), 4.20 (d, 2H, *J* = 2.3, H_e), 2.47 (t, 1H, *J* = 2.3, H_f), 1.33 (s, 18H, H_a); ^{13}C NMR (101 MHz, $CDCl_3$, 298 K) δ 151.0, 136.4, 122.6, 122.1, 80.0, 74.6, 72.5, 57.3, 29.9; LR-EI-MS (+ve) *m/z* (%) = 258.4 [M^+] (12); HR-EI-MS (+ve) m/z = 258.19862 [M^+] calc. 258.19782.

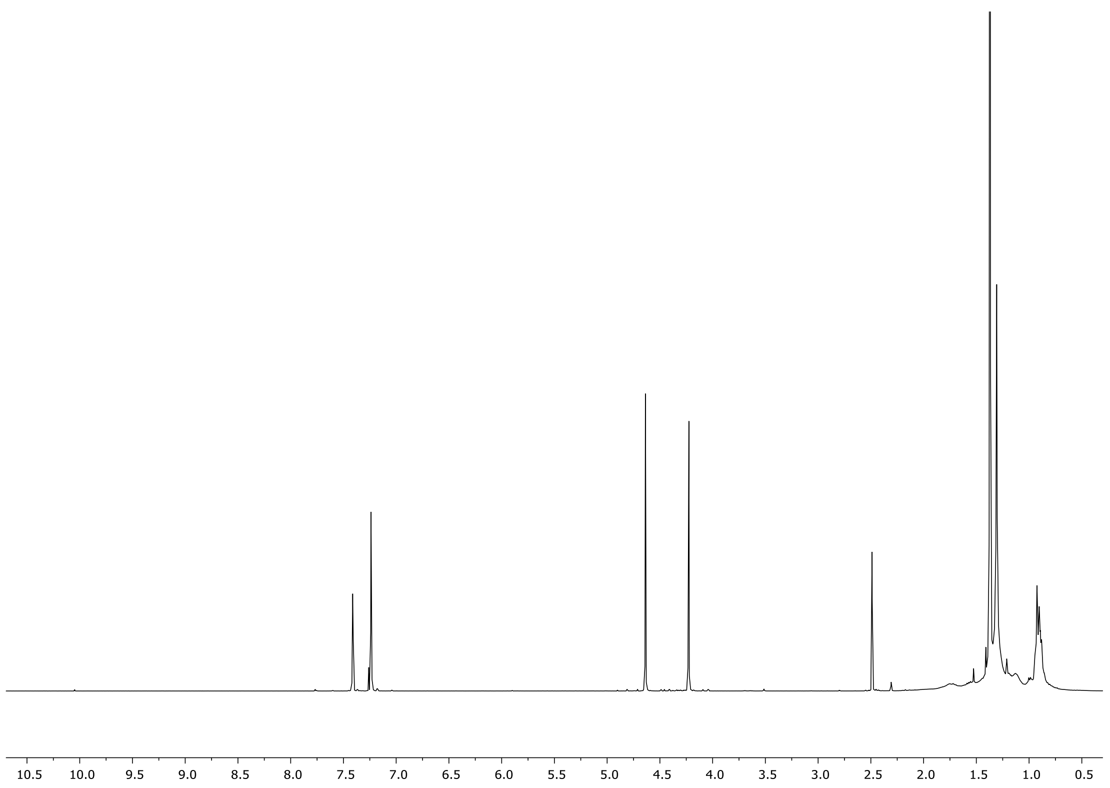

Figure S7. ^{1}H NMR (400 MHz, $CDCl_3$, 298 K) **2f**.

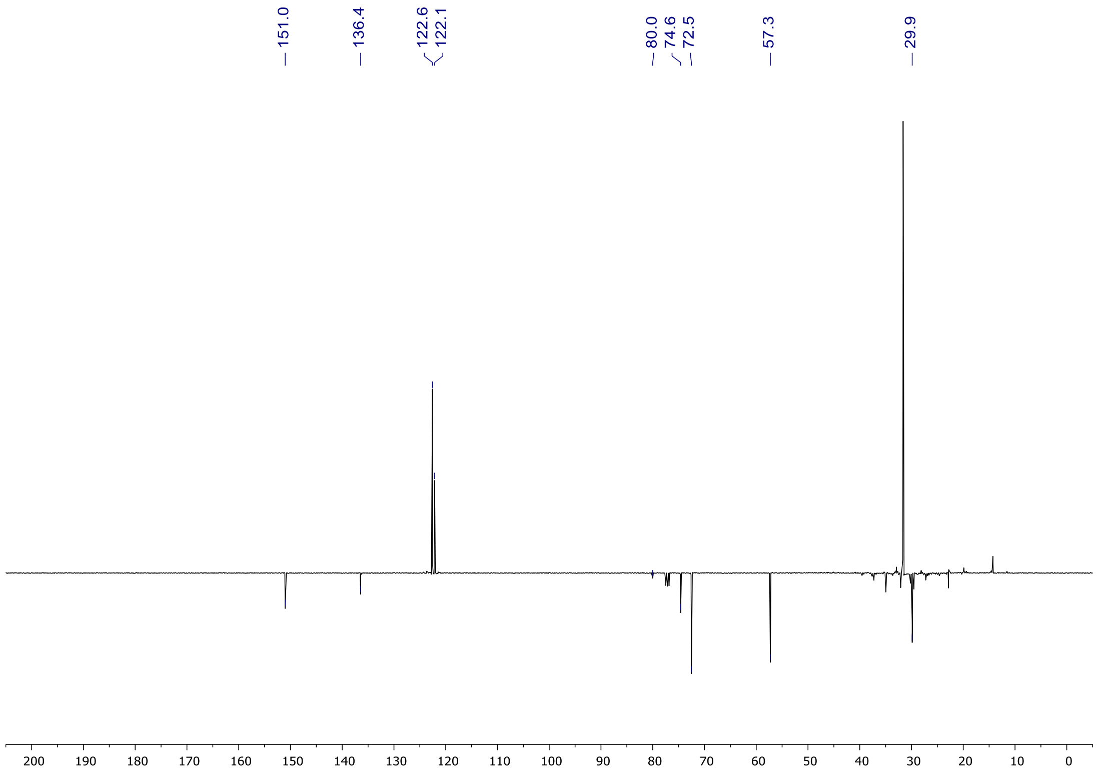

Figure S8. ^{13}C NMR (101 MHz, $CDCl_3$, 298 K) **2f**.

1,3-Di-*tert*-butyl-5-(pent-4-yn-1-yloxy)benzene (**2g**)

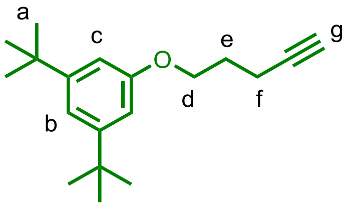

A 10 mL round bottom flask was charged with 3,5-di-*tert*-butylphenol (158 mg, 0.766 mmol), pent-4-yn-1-yl 4-methylbenzenesulfonate (308 mg, 1.29 mmol) K_2CO_3 (318 mg, 2.30 mmol), and DMF (5 mL), and the mixture was stirred at 80 °C for 16 h. After cooling to rt, H_2O (30 mL) was added, and the aqueous layer was extracted with EtOAc (3 × 30 mL). The combined organic extracts were washed with 5% w/v LiCl (5 × 30 mL), brine (30 mL), were dried over $MgSO_4$, filtered, and the solvent removed *in vacuo*. The residue was purified by chromatography (petrol with 0→15% CH_2Cl_2), to yield alkyne **2g** as a yellow oil (149 mg, 71%); ^{1}H NMR (400 MHz, $CDCl_3$, 298 K) δ 7.03 (t, 1H, *J* = 1.5, H_b), 6.77 (d, 2H, *J* = 1.5, H_c), 4.08 (d, 2H, *J* = 6.0, H_d), 2.43 (app. td, 1H, *J* = 9.7, 2.8 , H_e), 2.05-1.99 (m, 2H, H_f), 1.98 (t, 1H, *J* = 6.0, H_g), 1.32 (s, 18H, H_a); ^{13}C NMR (101 MHz, $CDCl_3$, 298 K) δ 158.6, 152.3, 115.2, 109.0, 83.9, 68.9, 66.1, 35.1, 31.6, 28.6, 15.4; LR-EI-MS (+ve) *m/z* (%) = 272.3 [M^+] (56); HR-EI-MS (+ve) *m/z* = 272.21218 [M^+] calc. 272.21347.

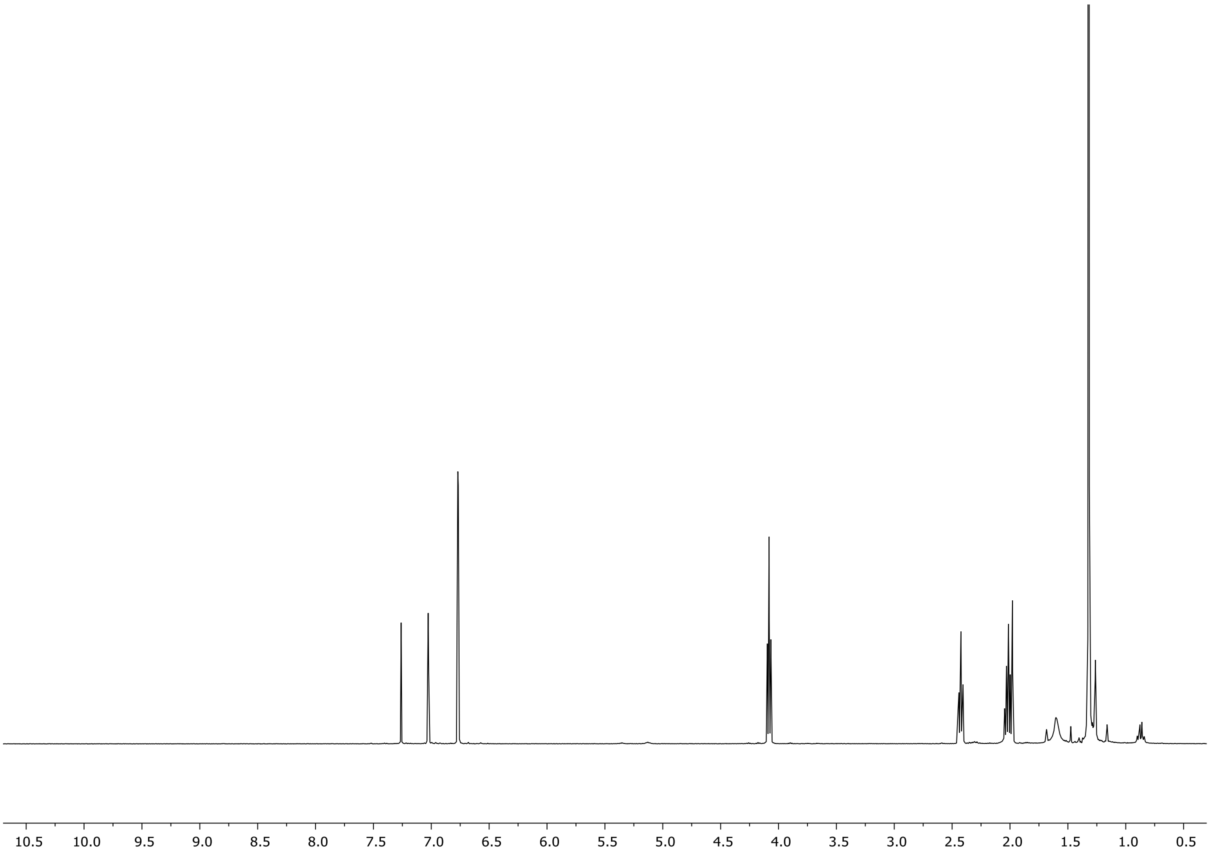

Figure S9. ^{1}H NMR (400 MHz, $CDCl_3$, 298 K) **2g**.

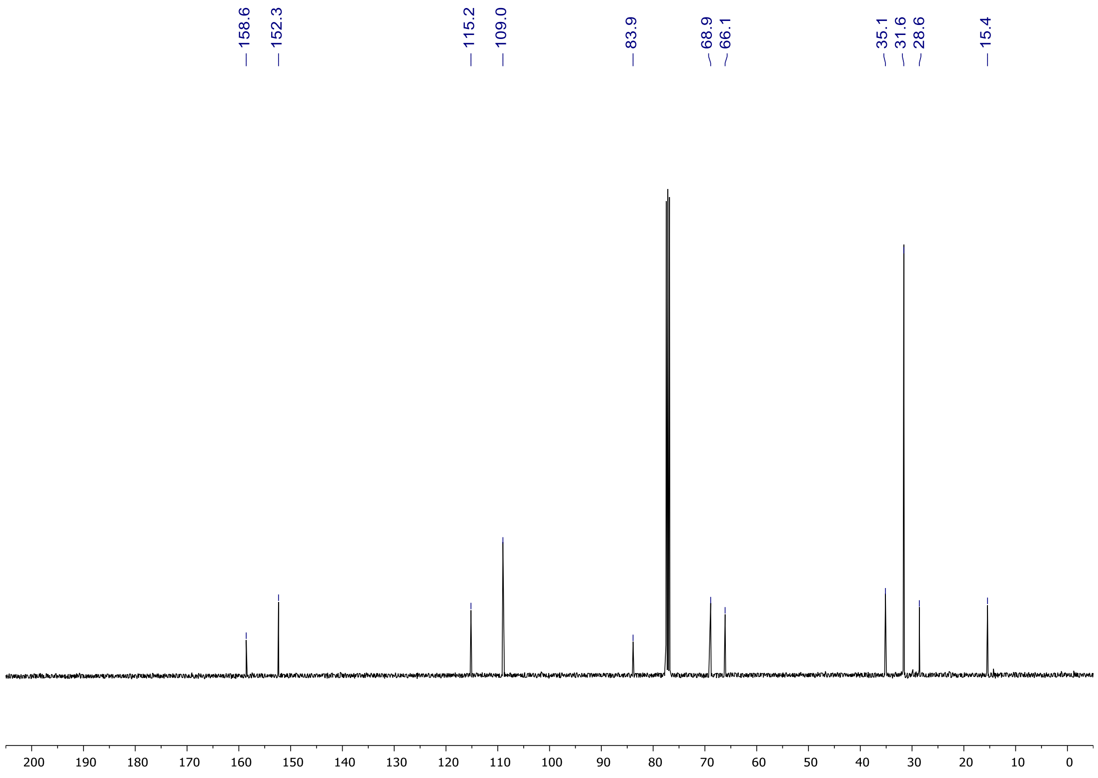

Figure S10. ^{13}C NMR (101 MHz, $CDCl_3$, 298 K) **2g**.

1,3-Di-*tert*-butyl-5-((4-ethynylphenoxy)methyl)benzene (**2h**)

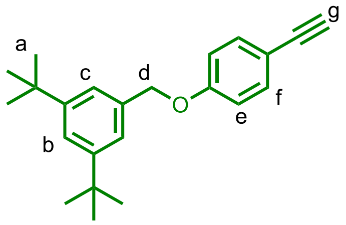

A 100 mL round bottomed flask was charged with alcohol **S3** (242 mg, 1.26 mmol), 3,5-di-*tert*-butylbenzyl bromide (337 mg, 1.39 mmol), K_2CO_3 (348 mg, 2.52 mmol), and DMF (50 mL) and the mixture was stirred at 80 °C for 16 h. After cooling to rt, H_2O (100 mL) was added, and the aqueous layer was extracted with petrol (3 × 50 mL). The combined organic extracts were washed with 5% w/v LiCl (3 × 50 mL), brine (50 mL), were dried over $MgSO_4$, filtered, and the solvent removed *in vacuo*, to yield alkyne **2h** as an orange solid (367 mg, 91%). No further purification was required; ^1H NMR (400 MHz, $CDCl_3$, 298 K) δ 7.47 (dt, 2H, *J* = 8.8, 2.0, H_f), 7.45 (t, 1H, *J* = 1.9, H_b), 6.29 (d, 2H, *J* = 1.9, H_c), 6.97 (dt, 2H, *J* = 8.8, 2.0, H_e), 5.06 (s, 2H, H_d), 3.02 (s, 1H, H_g), 1.37 (s, 18H, H_a); ^{13}C NMR (101 MHz, $CDCl_3$, 298 K) δ 159.6, 151.3, 135.6, 133.7, 122.5, 122.3, 115.0, 114.5, 83.9, 75.9, 71.1, 35.0, 31.6; LR-EI-MS (+ve) *m/z* (%) = 320.5 [M^+] (10); HR-EI-MS (+ve) *m/z* = 320.21245 [M^+] calc. 320.21347.

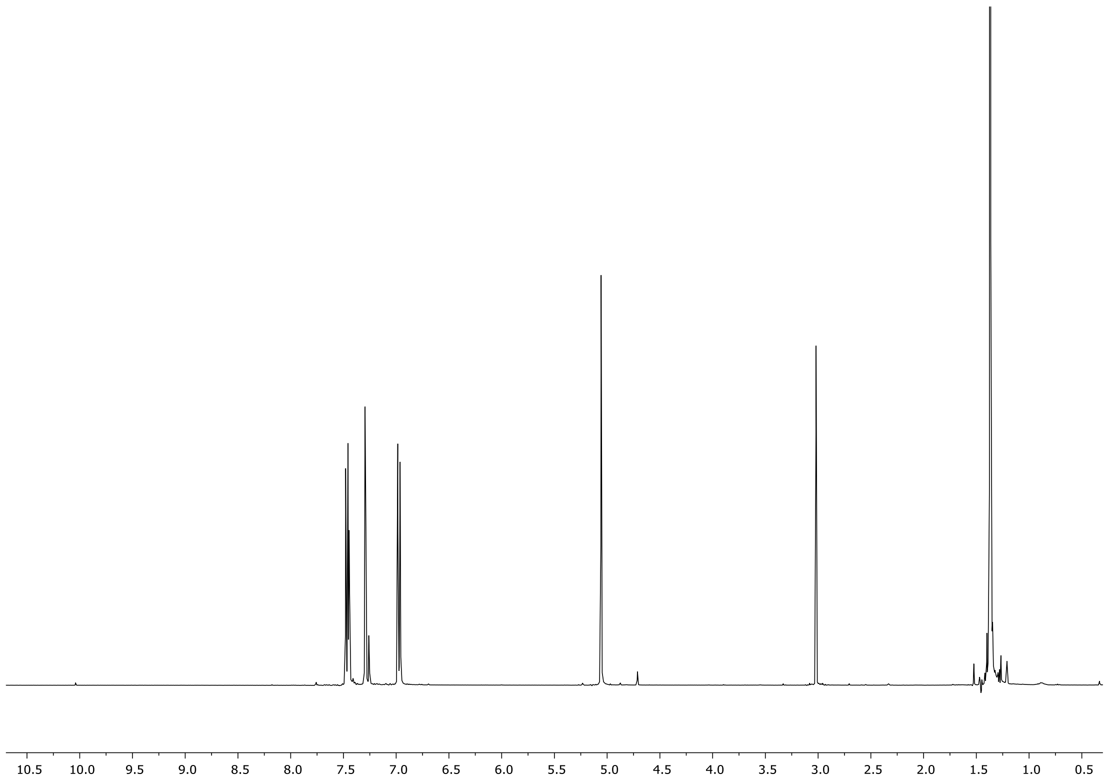

Figure S11. ^{1}H NMR (400 MHz, $CDCl_3$, 298 K) **2h**.

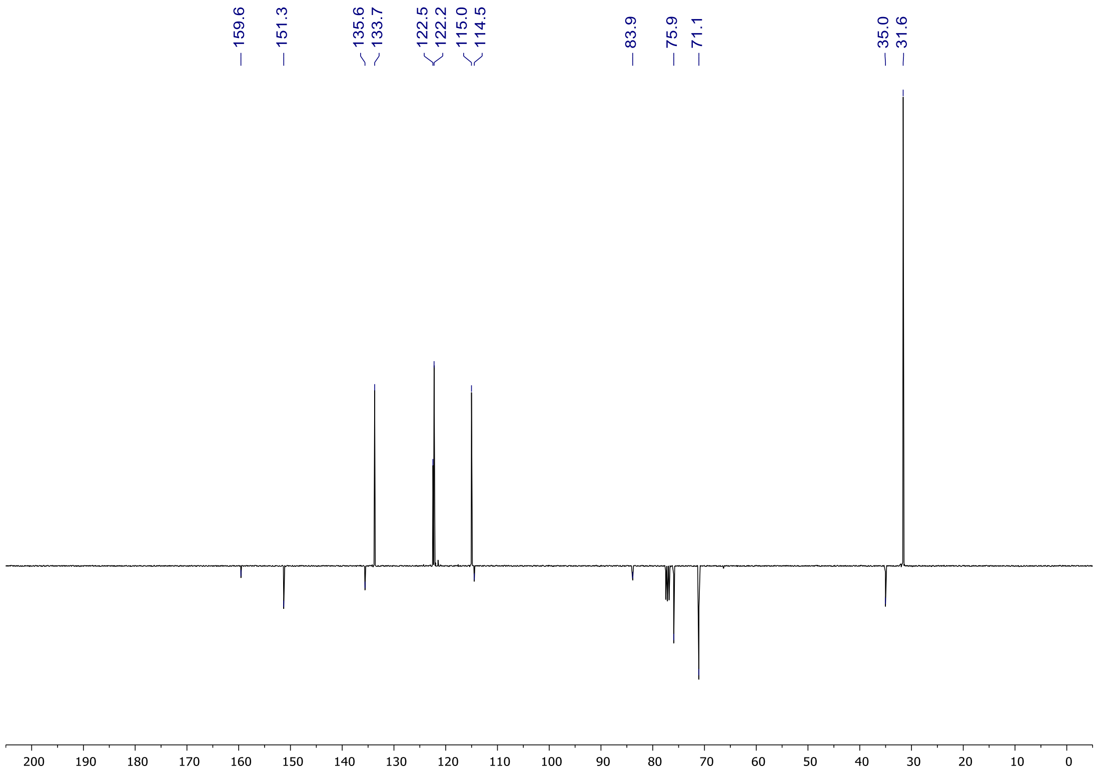

Figure S12. ^{13}C NMR (101 MHz, $CDCl_3$, 298 K) **2h**.

(*S*)-2-Amino-*N*-(3,5-di-*tert*-butylbenzyl)-3-phenylpropanamide ((*S*)-**S11**)

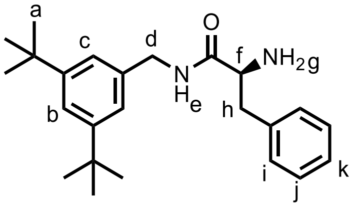

A 10 mL round bottom flask was charged with (*tert*-butoxycarbonyl)-*L*-phenylalanine (147 mg, 0.554 mmol), DMAP (74.3 mg, 0.608 mmol), 1-(3-dimethylaminopropyl)-3-ethyl-carboiimide hydrochloride (123 mg, 0.644 mmol), 1-hydroxybenzotriazole hydrate (113 mg, 0.738 mmol, assuming monohydrate), **S4** (108 mg, 0.498 mmol) and CH_2Cl_2 (5 mL). The reaction mixture was stirred at rt for 16 h. HCl (20 mL of a 1M solution) was added, and the aqueous layer was extracted with CH_2Cl_2 (3 × 20 mL). The combined organic extracts were washed with HCl (20 mL of a 1 M solution) and brine (20 mL), dried over $MgSO_4$, filtered, and the solvent removed *in vacuo*. In a 10 mL round bottom flask, the residue was dissolved in CH_2Cl_2 (5 mL). TFA (0.330 mL, 4.86 mmol) was added and the reaction mixture was stirred at rt for 16 h. The solution was concentrated *in vacuo*, dissolved in $CHCl_3$ (20 mL) and poured onto saturated Na_2CO_3 (20 mL). The phases were separated and the aqueous layer was extracted with $CHCl_3$ (2 × 20 mL), The combined organic extracts were dried over $MgSO_4$, filtered, and the solvent removed *in vacuo* to yield amide (*S*)-**S11** as an yellow oil (111 mg, 62% over two steps); ^1H NMR (400 MHz, $CDCl_3$, 298 K) δ 7.54-7.47 (br s, 1H, H_e), 7.37 (t, 1H, *J* = 2.0, H_b), 7.35-7.29 (m, 2H, H_i), 7.28-7.2- (m, 3H, H_j and H_k), 7.12 (d, 2H, *J* = 2.0, H_c), 4.45 (dd, 1H, *J* = 14.5, 6.0, one of H_d), 4.41 (dd, 1H, *J* = 14.5, 6.0, one of H_d), 3.58 (dd, 1H, *J* = 9.9, 4.4, H_f), 3.34 (dd, 1H, *J* = 13.4, 4.4, one of H_h), 2.75 (dd, 1H, *J* = 14.4, 9.9, H_h), 1.52-1.42 (br s, 2H, H_g), 1.39 (s, 18H, H_a); ^{13}C NMR (101 MHz, $CDCl_3$, 298 K) δ 174.1, 151.3, 138.1, 137.6, 129.5, 128.9, 127.0, 122.3, 121.7, 56.7, 44.0, 41.3, 35.0, 31.6; LR-ESI-MS (+ve) *m/z* (%) = 367.3 [M+H] (100); HR-ESI-MS (+ve) *m/z* = 367.2754 [M+H] calc. 367.2744.

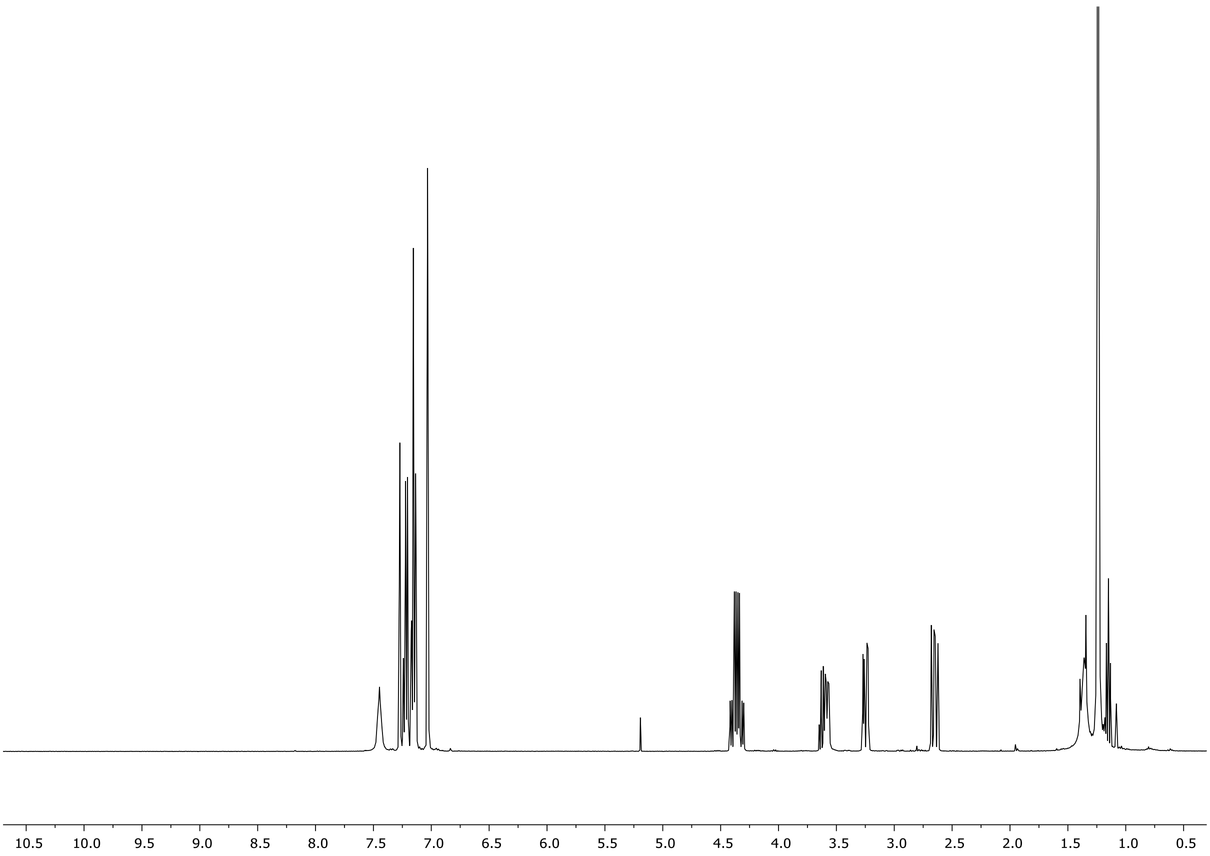

Figure S13. ^{1}H NMR (400 MHz, $CDCl_3$, 298 K) (*S*)-**S11**.

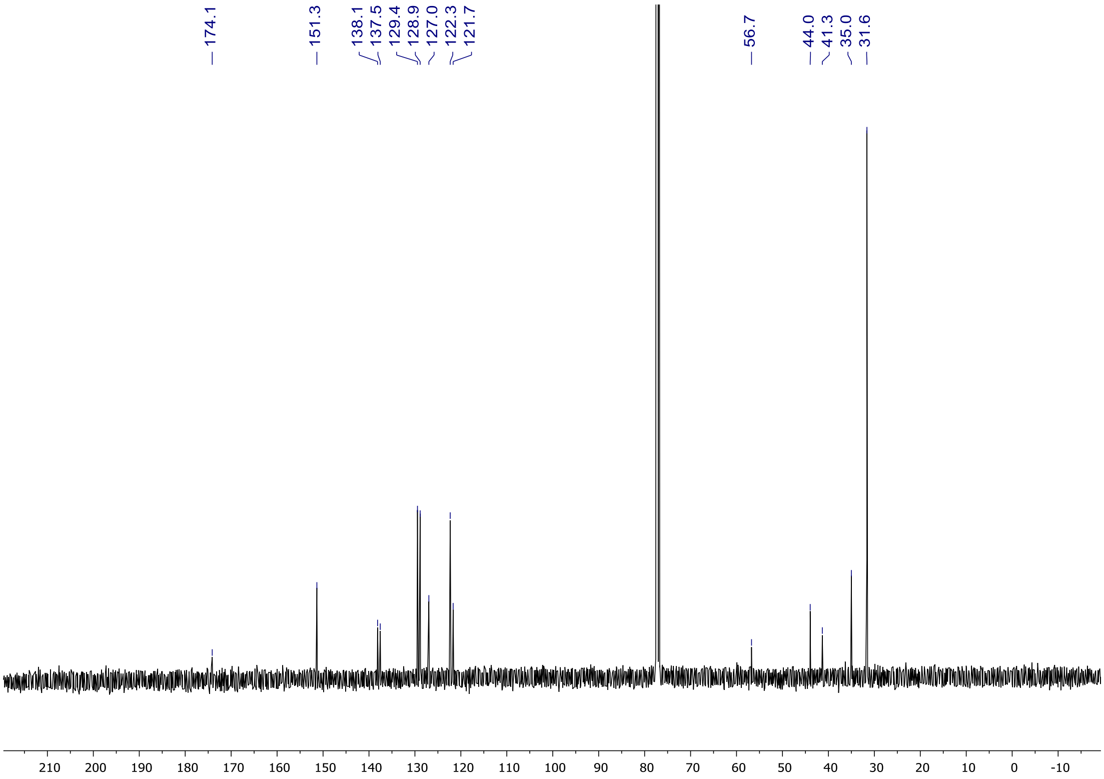

Figure S14. ^{13}C NMR (101 MHz, $CDCl_3$, 298 K) (*S*)-**S11**.

(*S*)-2-Azido-N-(3,5-di-*tert*-butylbenzyl)-3-phenylpropanamide ((*S*)-**3c**)

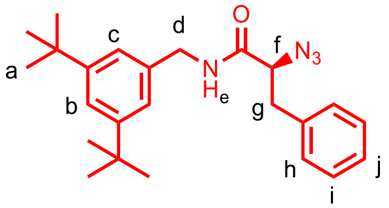

A dry 25 mL round bottomed flask was charged with **S11** (160 mg, 0.476 mmol), DMAP (174 mg, 1.43 mmol), **S10** (203 mg, 0.712 mmol) and anhydrous MeCN (3 mL) was stirred at rt for 2 h, protected by a nitrogen atmosphere. The solvent was removed *in vacuo*, CH_2Cl_2 (30 mL) was added, and the organic layer was washed with H_2O (2 × 20 mL), dried over $MgSO_4$, filtered, and the solvent removed *in vacuo*. The residue was purified by chromatography (petrol with 0→100% CH_2Cl_2), to yield azide (*S*)-**3c** as a yellow oil (140 mg, 75%, 99.6% *ee*); ^{1}H NMR (400 MHz, $CDCl_3$, 298 K) δ, 7.29 (t, 1H, *J* = 1.6, H_b), 7.25-7.17 (m, 5H, H_h, H_j and H_i), 6.99 (d, 2H, *J* = 1.6, H_c), 6.44-7.6.23 (br t, 1H, *J* = 5.8, $H_{e,}$), 4.46 (dd, 1H, *J* = 14.6, 5.8, one of H_d), 4.29 (dd, 1H, *J* = 14.6, 5.8, one of H_d), 4.15 (dd, 1H, *J* = 7.9, 4.3, H_f), 3.31 (dd, 1H, *J* = 13.8, 4.3, one of H_g), 2.96 (dd, 1H, *J* = 13,8, 5.6, one of H_g), 1.24 (s, 18H, H_a); ^{13}C NMR (101 MHz, $CDCl_3$, 298 K) 168.4, 151.4, 136.4, 136.2, 129.5, 128.7, 127.2, 122.3, 121.9, 76.7, 65.8, 44.3, 38.8, 34.9, 31.5; LR-ESI-MS (+ve) *m/z* (%) = 393.5 [M+H] (100); HR-ESI-MS (+ve) *m/z* = 393.2644 [M+H] calc. 393.2649; Chiral SCFC (Lux C2, 250 × 4.6 mm, 5 μm, 40 °C, MeOH (0.2% v/v NH_3)/CO_2 = 1 : 9→1 1, 4 mL/min, λ = 210-400 nm); tR [(*S*)-**3c**] = 2.00, tR [(*R*)-**3c**] = 1.84.

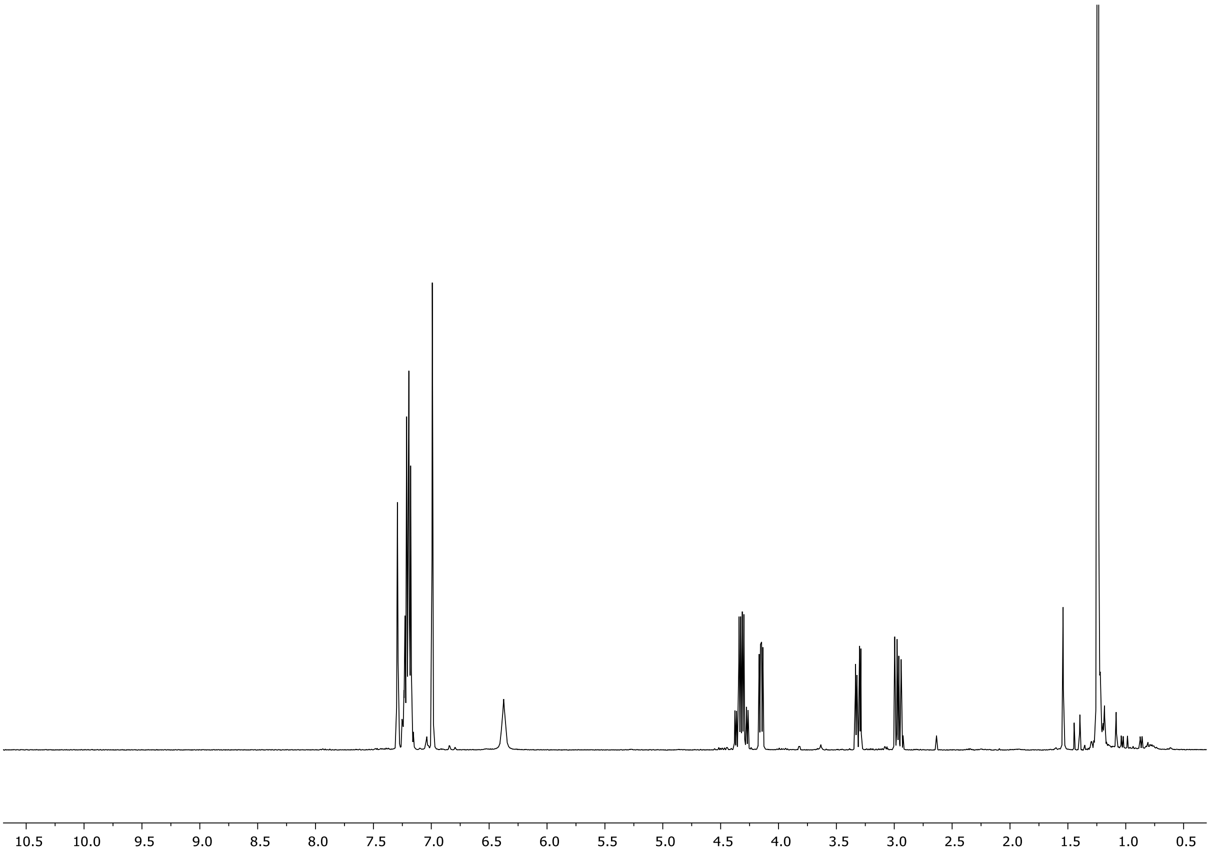

Figure S15. ^{1}H NMR (400 MHz, $CDCl_3$, 298 K) (*S*)-**3c**.

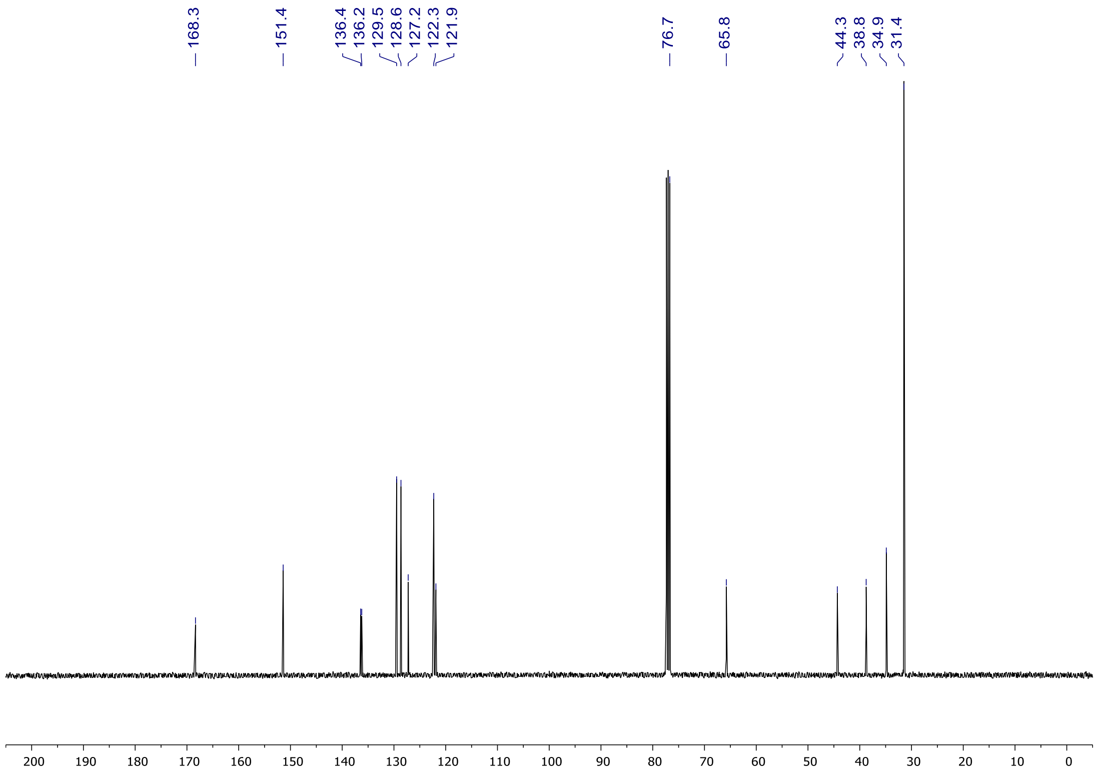

Figure S16. ^{13}C NMR (101 MHz, $CDCl_3$, 298 K) (*S*)-**3c**.

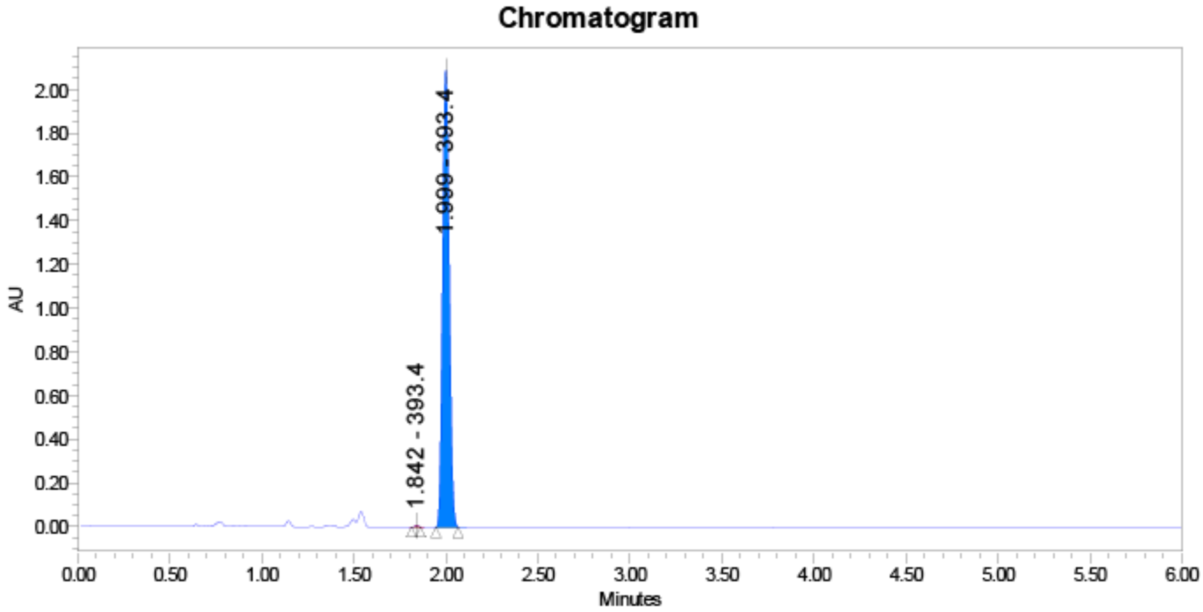

Peak Results

	Retention Time (min)	Area (μV*sec)	% Area	Width @ 50%
1	1.84	9428	0.2	0.02471
2	2.00	5383625	99.8	0.04102

Figure S17. Chiral SCFC chromatogram of (*S*)-**3c**.

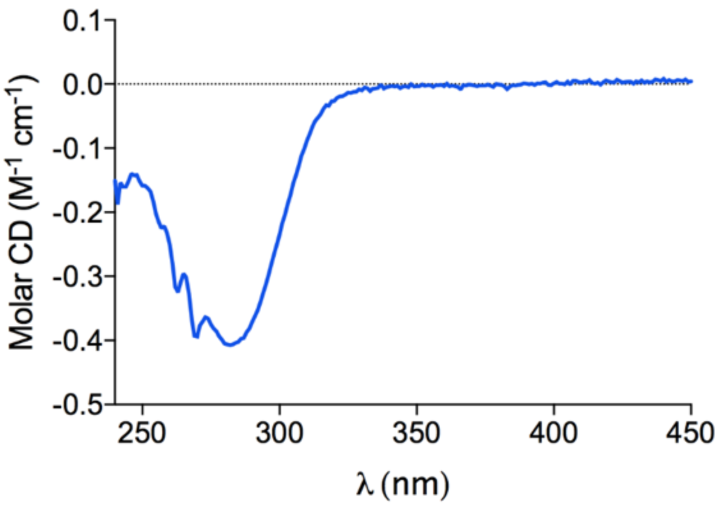

Figure S18. Circular dichroism spectrum of (*S*)-**3c** (66.2 mM in $CHCl_3$).

3,5-Di-*tert*-butylbenzyl (*S*)-2-azido-3-phenylpropanoate ((*S*)-**3d**)

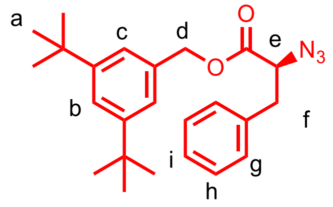

S5 (220 mg, 1.0 mmol), **S6** (128 mg, 0.63 mmol), *N*-(3-dimethylaminopropyl)-*N'*-ethylcarbodiimide hydrochloride (191 mg, 1.0 mmol), DMAP (8 mg, 0.063 mmol) and 1-hydroxybenzotriazole hydrate (135 mg, 1.0 mmol, assuming monohydrate) was dissolved in CH_2Cl_2 (5 mL) and the reaction mixture was stirred overnight at rt, protected by an argon atmosphere. CH_2Cl_2 (20 mL) was added and the organic layer was washed with 1M HCl (15 mL), H_2O (15 mL) and brine (20 mL). The combined organic layer were dried over $MgSO_4$, filtered, and had the solvent removed *in vacuo*. The residue was purified by chromatography (isocratic 25% CH_2Cl_2 in petrol), to yield azide (*S*)-**3d** as oil (144 mg, 58%); ^{1}H NMR (400 MHz, $CDCl_3$, 298 K) δ 7.49 (t, 1H, *J* = 1.8, H_b), 7.37-7.29 (m, 3H, H_h and H_i), 7.28-7.22 (m, 4H, H_g and H_c), 5.27 (d, 1H, *J* = 12.0, H_d), 5.22 (d, 1H, *J* = 12.0, H_d), 4.15 (dd, 1H, *J* = 8.6, 5.6, H_e), 3.25 (dd, 1H, *J* = 14.0, 5.5, one of H_f), 3.08 (dd, 1H, *J* = 14.0, 8.6, one of H_f), 1.39 (s, 18H, H_a); ^{13}C NMR (101 MHz, $CDCl_3$, 298 K) δ 170.0, 151.4, 136.0, 134.1, 129.3, 128.78, 127.4, 123.0, 122.8, 68.5, 63.4, 37.8, 35.0, 31.6.

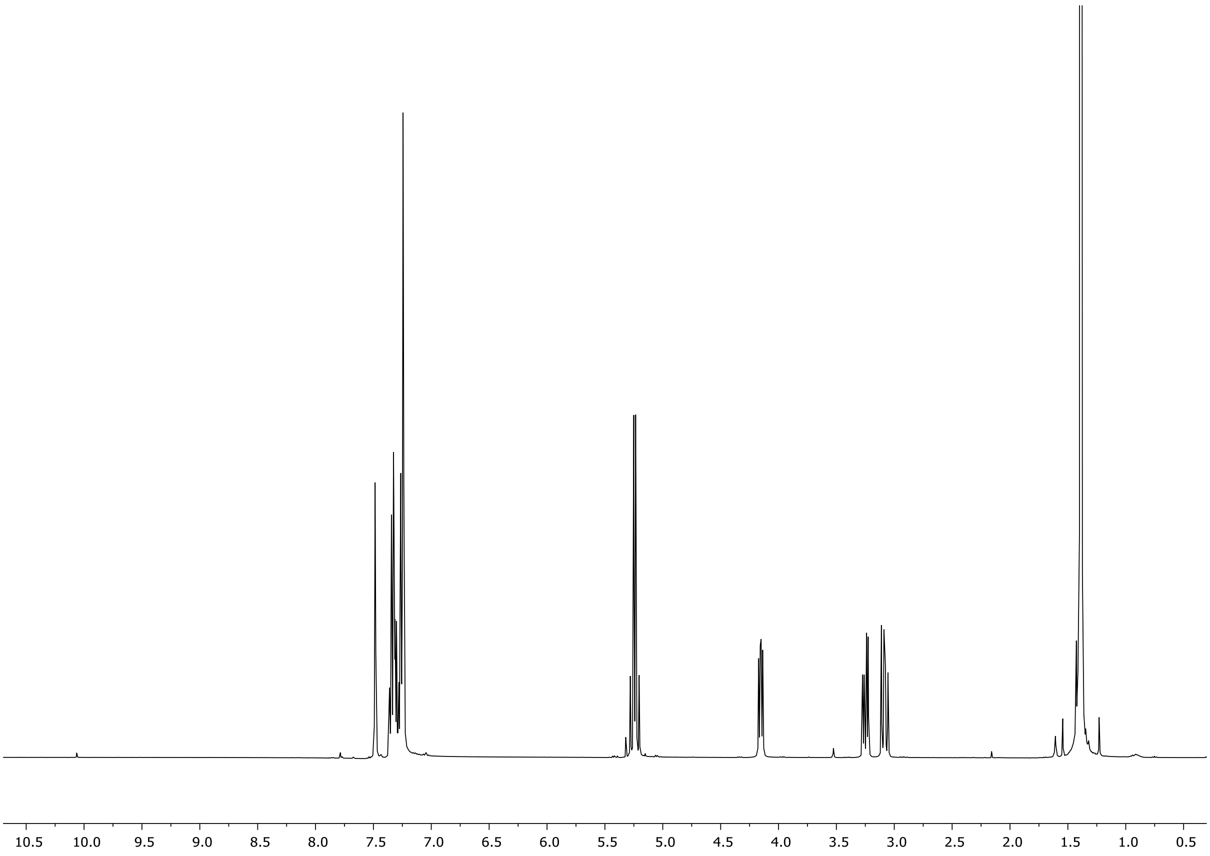

Figure S19. ^{1}H NMR (400 MHz, $CDCl_3$, 298 K) (*S*)-**3d**.

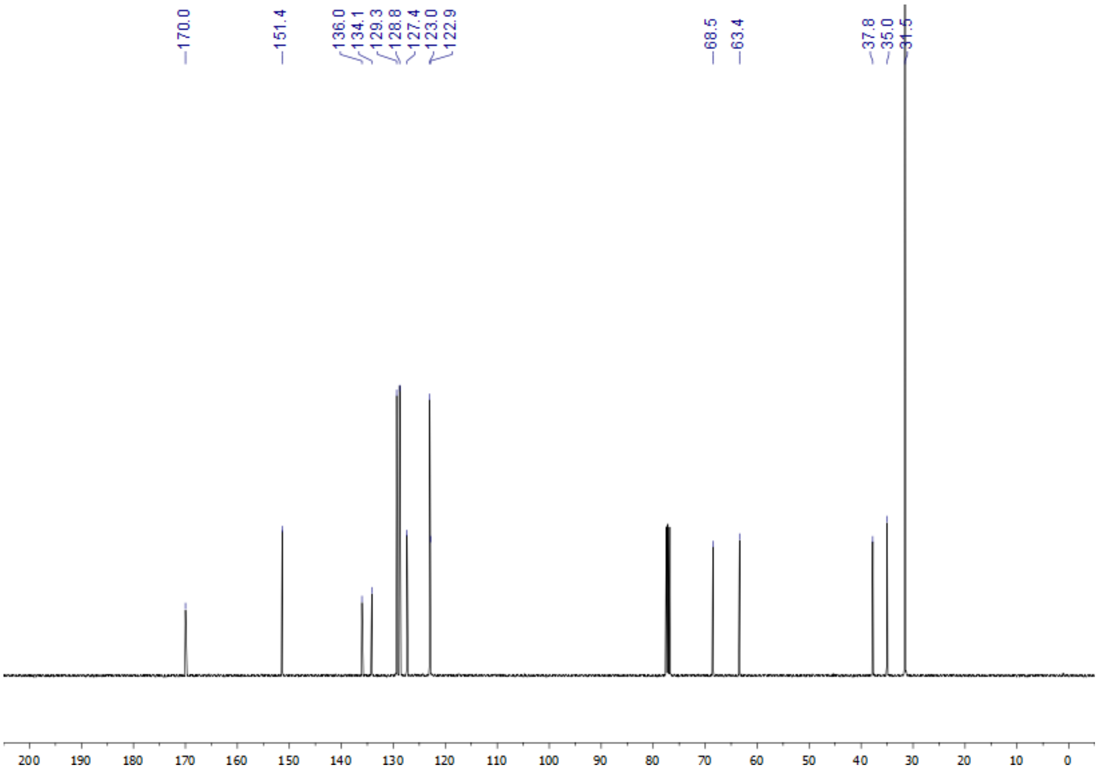

Figure S20. ^{13}C NMR (400 MHz, $CDCl_3$, 298 K) (*S*)-**3d**.

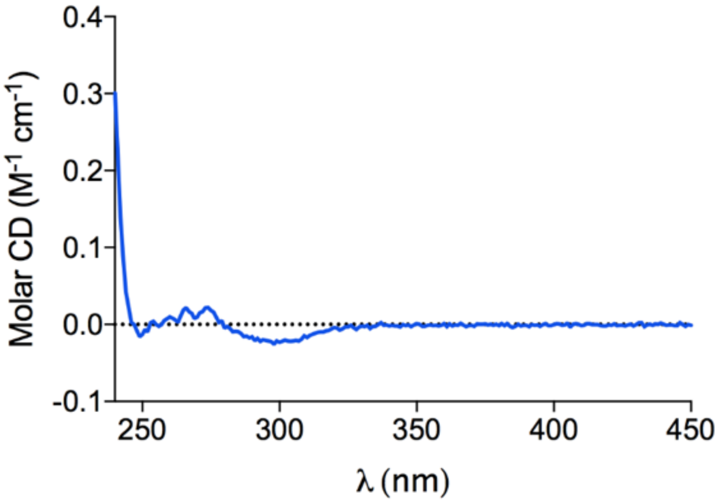

Figure S21. Circular dichroism spectrum of (*S*)-**3d** (86.0 mM in $CHCl_3$, 298 K).

Isopropyl (*S*)-2-azido-3-phenylpropanoate ((*S*)-**3e**)

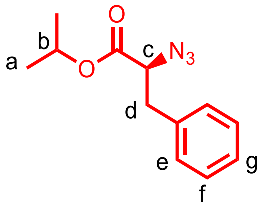

A dry 25 mL round bottom flask was charged with (*S*)-**S7** (605 mg, 2.92 mmol), DMAP (1.06 g, 8.73 mmol), **S10**(1.06 g, 3.71 mmol), and anhydrous MeCN (10 mL). The reaction mixture was stirred at rt for 2 h, protected by a nitrogen atmosphere. Saturated $NaHCO_3$ (30 mL) was added, and the aqueous layer was extracted with CH_2Cl_2 (3 × 100 mL). The combined organic extracts were dried over $MgSO_4$, filtered, and the solvent removed *in vacuo*. The residue was purified by chromatography (petrol with 0→100% CH_2Cl_2), to yield the product as a yellow oil (500 mg, 73%, >99.9% ee); ^{1}H NMR (400 MHz, $CDCl_3$, 298 K) δ 7.34-7.10 (m, 5H, H_e, H_f and H_g), 4.96 (sept, 1H, *J* = 6.3, H_b), 3.90 (dd, 1H, *J* = 8.6, 5.7, H_c), 3.05 (dd, 1H, *J* = 14.1, 5.7, one of H_d), 2.91 (dd, 1H, *J* = 14.1, 8.6, one of H_d), 1.16 (d, 3H, *J* = 6.3, three of H_a), 1.11 (d, 1H, *J* = 6.3, three of H_a); ^{13}C NMR (101 MHz, $CDCl_3$, 298 K) δ 169.5, 136.0, 129.3, 128.6, 127.2, 69.8, 63.2, 37.6, 21.7, 21.6; HR-EI-MS (+ve) *m/z* = 233.11605 [M^+] calc. 233.11588; Chiral SCFC (Chiralpak IG, 250 × 4.6 mm, 5 µm, 40 °C, MeOH (0.2% v/v NH_3)/CO_2 = 10%→50%, 4 mL/min, λ = 210-400 nm,): tR [(*R*/*S*)-**3e**] = 1.27 min, 1.61 min; tR [(*S*)-**3e**] = 1.61.

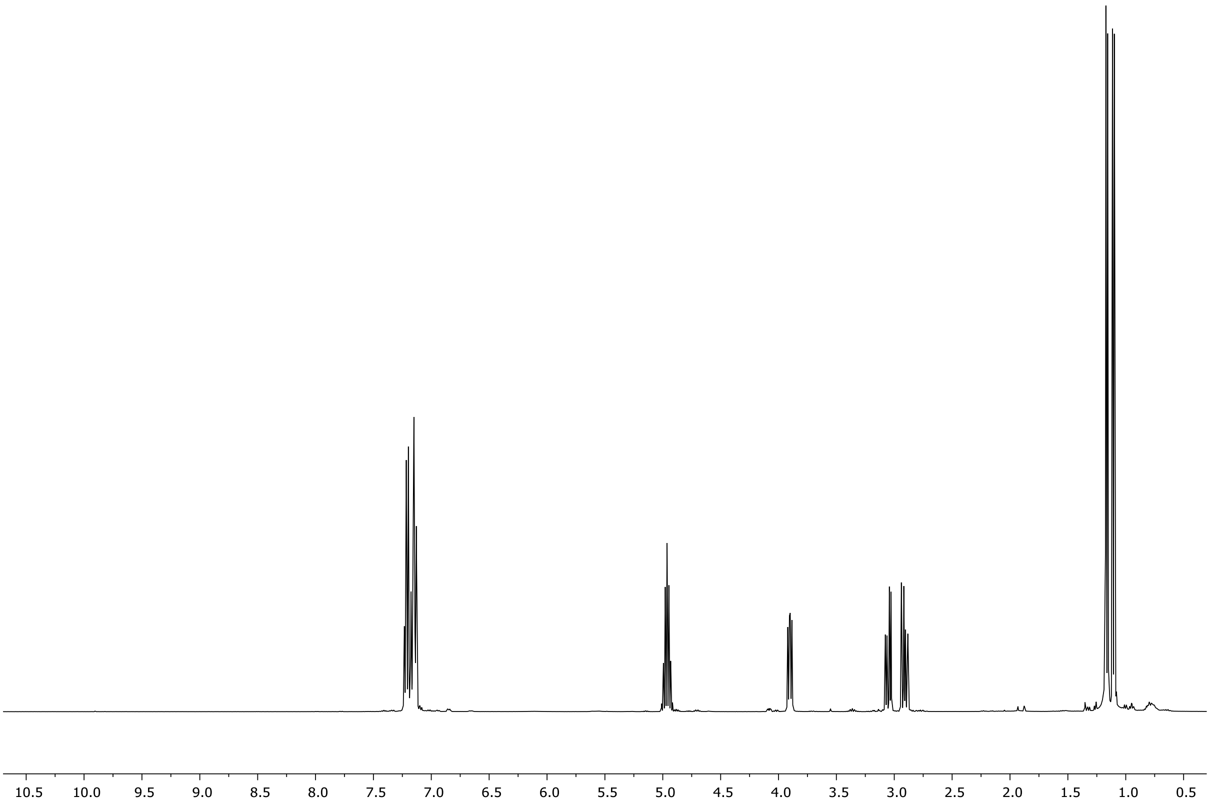

Figure S22. ^{1}H NMR (400 MHz, $CDCl_3$298 K) (*S*)-**3e**.

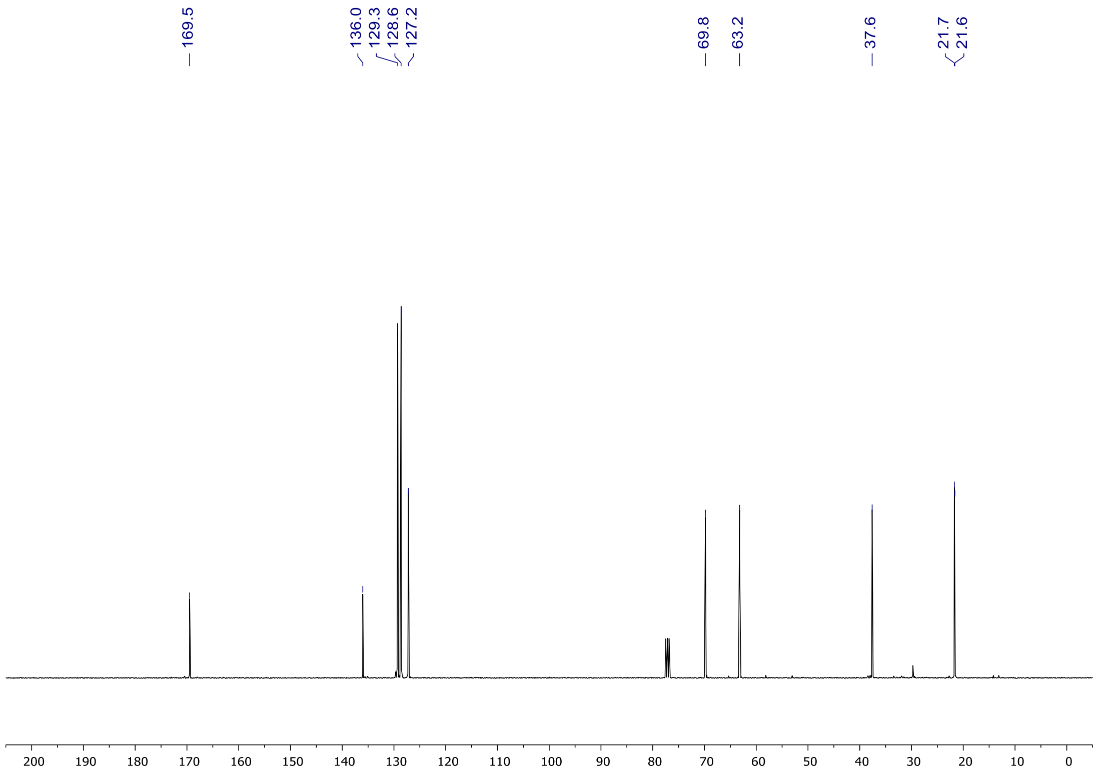

Figure S23. ^{13}C NMR (101 MHz, $CDCl_3$, 298 K) (*S*)-**3e**.

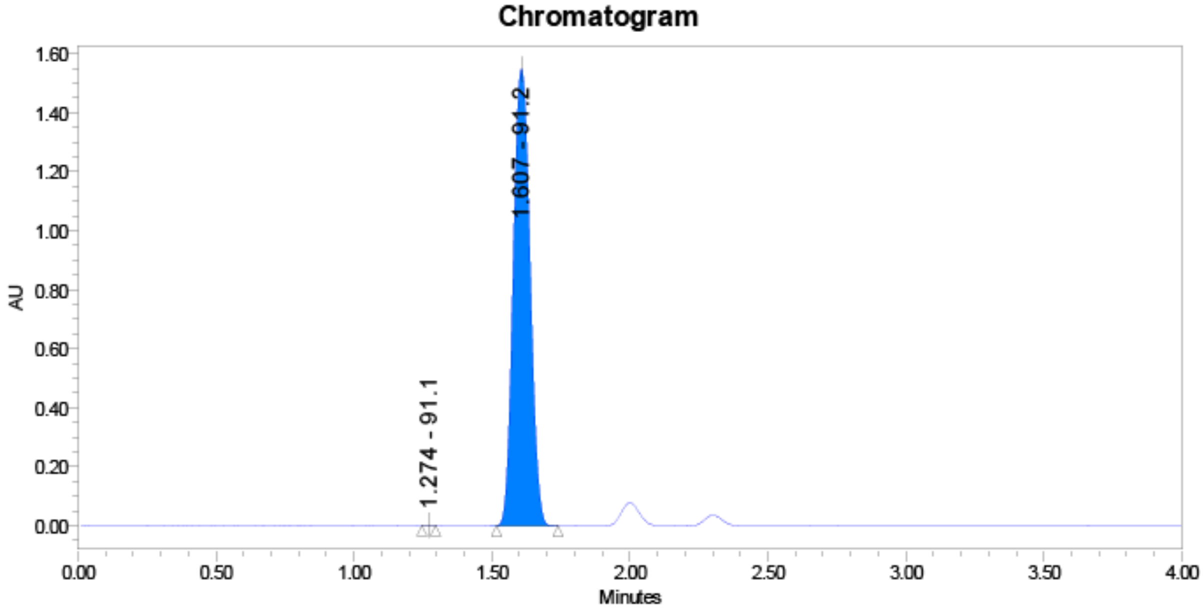

Peak Results

	Retention Time (min)	Area (µV*sec)	% Area	Width @ 50%
1	1.27	1140	0.0	0.02540
2	1.61	6505511	100.0	0.06701

Figure S24. Chiral SCFC chromatogram of (*S*)-**3e**.

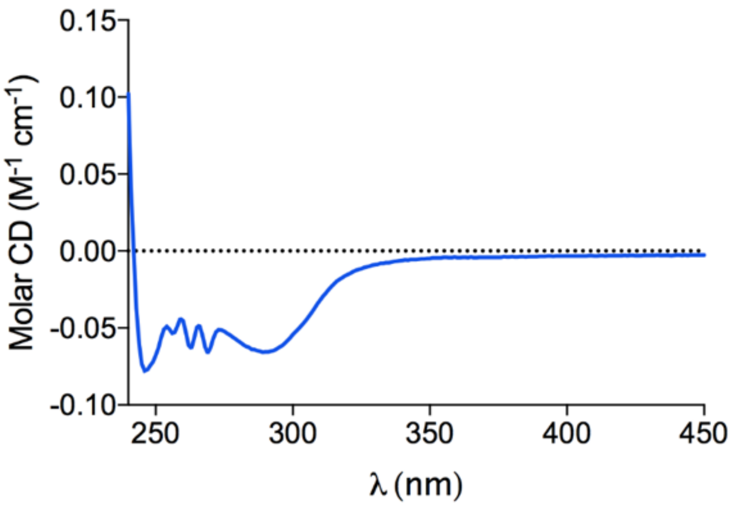

Figure S25. Circular dichroism spectrum of (*S*)-**3e** (1.67 mM in $CHCl_3$).

Isopropyl (*R*)-2-azido-3-phenylpropanoate ((*R*)-**3e**)

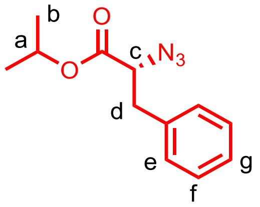

A dry 10 mL round bottom flask was charged with (*R*)-**S7** (277 mg, 1.34 mmol), DMAP (490 mg, 4.01 mmol), 2-azido-1,3-dimethylimidazolinium hexafluorophosphate (590 mg, 2.07 mmol) and anhydrous MeCN (5 mL). The reaction mixture was stirred at rt for 2 h, protected by a nitrogen atmosphere. Saturated $NaHCO_3$ (20 mL) was added, and the aqueous layer was extracted with CH_2Cl_2 (3 × 30 mL). The combined organic extracts were dried over $MgSO_4$, filtered, and the solvent removed *in vacuo*. The residue was purified by chromatography (petrol with 0→100% CH_2Cl_2), to yield (*R*)-**3e** as a yellow oil, with spectral data identical to (*S*)-**3e**, but opposite CD spectra (**Figure S27**) (196 mg, 63%, 99.6% ee); Chiral SCFC (Chiralpak IG, 250 × 4.6 mm, 5 µm, 40 °C, MeOH (0.2% v/v NH_3)/CO_2 = 10→50%, 4 mL/min, λ = 210-400 nm,): tR [racemate] = 1.26 min, 1.61 min; tR [(*R*)-**3e**] = 1.26.

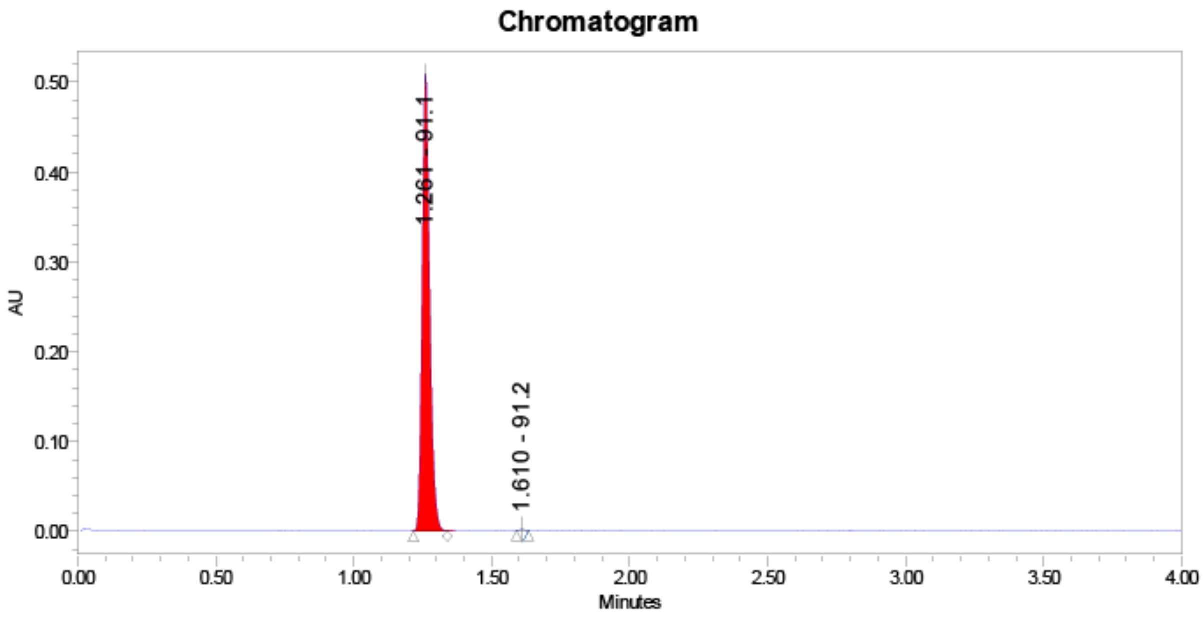

Peak Results

	Retention Time (min)	Area (μV*sec)	% Area	Width @ 50%
1	1.26	986755	99.8	0.02931
2	1.61	1764	0.2	0.02421

Figure S26. Chiral SCFC chromatogram of (*R*)-**3e**.

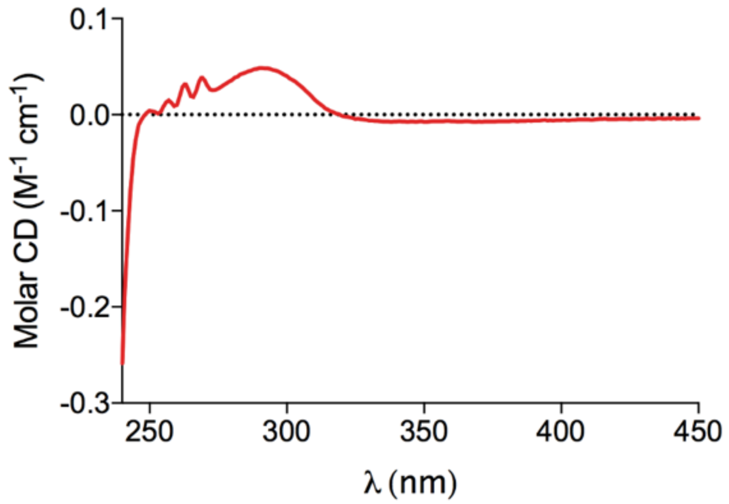

Figure S27. Circular dichroism spectrum of (*R*)-**3e** (0.789 mM in $CHCl_3$, 298 K).

Isopropyl (*R,S*)-2-azido-3-phenylpropanoate ((*R*/*S*)-**3e**)

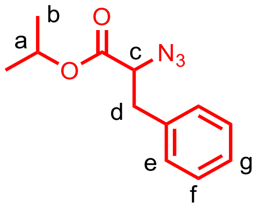

A dry 10 mL round bottom flask was charged with (*R*/*S*)-**S7** (279 mg, 1.35 mmol), DMAP (493 mg, 4.04 mmol), **S10** (395 mg, 1.39 mmol), and anhydrous MeCN (5 mL). The reaction mixture was stirred at rt for 2 h, protected by a nitrogen atmosphere. Saturated $NaHCO_3$ (20 mL) was added, and the aqueous layer was extracted with CH_2Cl_2 (3 × 50 mL). The combined organic extracts were dried over $MgSO_4$, filtered, and the solvent removed *in vacuo*. The yellow solid was purified by chromatography (petrol with 0→100% CH_2Cl_2), to yield (*R*/*S*)-**3e** as a yellow oil, with spectral data identical to **3e** and **3f** (92.0 mg, 29%, 0.0% ee); Chiral SCFC (Chiralpak IG, 250 × 4.6 mm, 5 µm, 40 °C, MeOH (0.2% v/v NH_3)/CO_2 = 10→50%, 4 mL/min, λ = 210-400 nm,): tR [(*R*/*S*)-**3e**] = 1.27 min [(*S*)-**3e**], 1.61 min [(*R*)-**3e**].

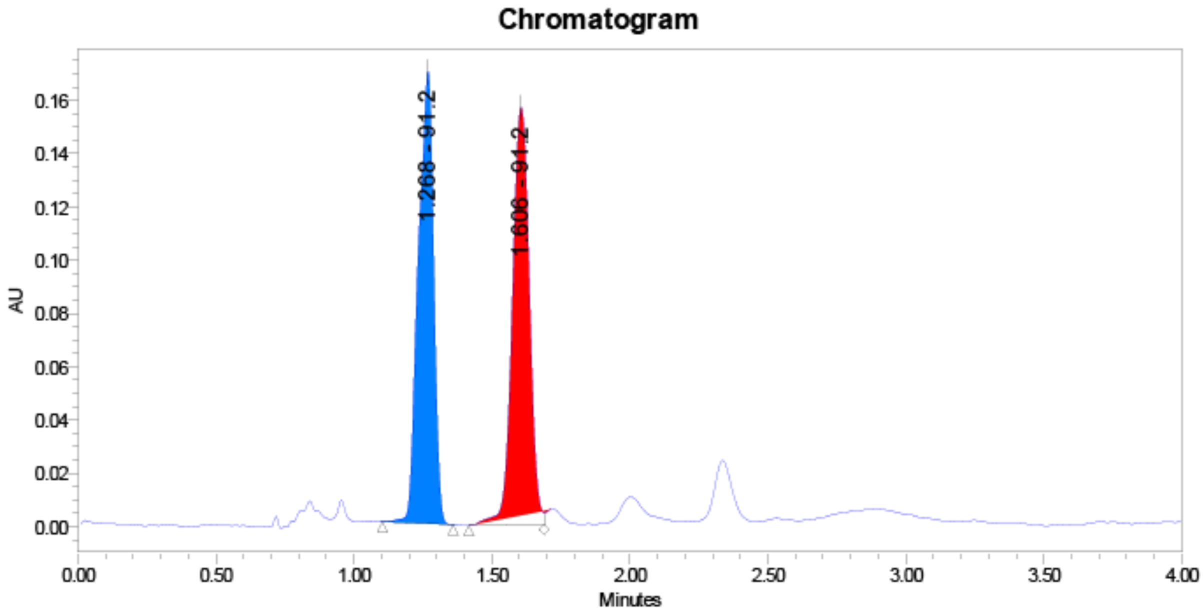

Peak Results

	Retention Time (min)	Area (µV*sec)	% Area	Width @ 50%
1	1.27	647881	49.0	0.06380
2	1.61	674090	51.0	0.06616

Figure S28. Chiral SCFC chromatogram of (*R*/*S*)-**3e**.

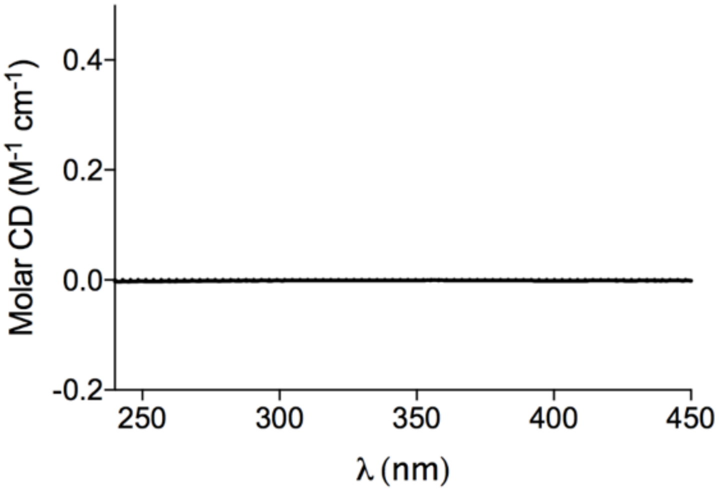

Figure S29. Circular dichroism spectrum of (*R*/*S*)-**3e** (3.93 mM in $CHCl_3$, 298 K).

3. Synthesis and characterisation of axles and rotaxanes (table 1)

Entry 1 – axle and rotaxanes derived from alkyne 2a and azide 3a

These have been reported previously. See ref 3.

Entry 2 – axle S12 and rotaxanes S13 derived from alkyne 2b and azide 3b

Axle (D)-**S12**

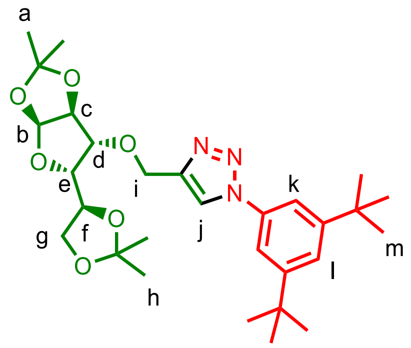

A dry sealed vessel was charged with (D)-**2b** (22.4 mg, 0.075 mmol), **3b** (17.4 mg, 0.075 mmol), $[Cu(MeCN)_4]PF_6$ (26.8 mg, 0.073 mmol) and anhydrous CH_2Cl_2 (1.25 mL). The reaction mixture was stirred at rt for 16 h, protected by an argon atmosphere. Saturated EDTA-NH_3 (10 mL) was added, and the aqueous layer was extracted with $CHCl_3$ (3 × 20 mL), dried over $MgSO_4$, filtered, and had the solvent removed *in vacuo*. The residue was purified by chromatography (75% CH_2Cl_2-MeCN), to yield axle (D)-**S12** as a yellow oil (37.7 mg, 95%); ^1H NMR (400 MHz, $CDCl_3$, 298 K) δ 8.00 (s, 1H, H_j), 7.53-7.50 (m, 1H, H_l), 7.49 (d, 2H, *J* = 1.7, H_k), 5.91 (d, 1H, *J* = 3.7, H_b), 4.93 (d, 1H, *J* = 12.6, one of H_i), 4.87 (d, 1H, *J* = 12.6, one of H_i), 4.67 (d, 1H, *J* = 3.7, H_c), 4.36 (dt, 1H, *J* = 8.6, 5.5, H_f), 4.15-4.07 (m, 3H, H_d, H_e and one of H_g), 4.01 (dd, 1H, *J* = 8.6, 5.4, one of H_g), 1.50 (s, 3H, three of H_a), 1.42-1.38 (m, 3H, three of H_h), 1.37 (s, 18H, H_m), 1.33 (s, 3H, three of H_h), 1.32 (s, 3H, three of H_a); ^{13}C NMR (126 MHz, $CDCl_3$, 298 K) δ 153.0, 145.3, 136.9, 123.2, 121.5, 115.8, 112.1, 109.2, 105.4, 82.8, 81.9, 81.3, 72.5, 67.7, 64.3, 35.3, 31.5, 27.0, 27.0, 26.4, 25.6.; HR-ESI-MS (+ve) m/z = 552.3043 $[M+Na]^+$ calc. 552.3044.

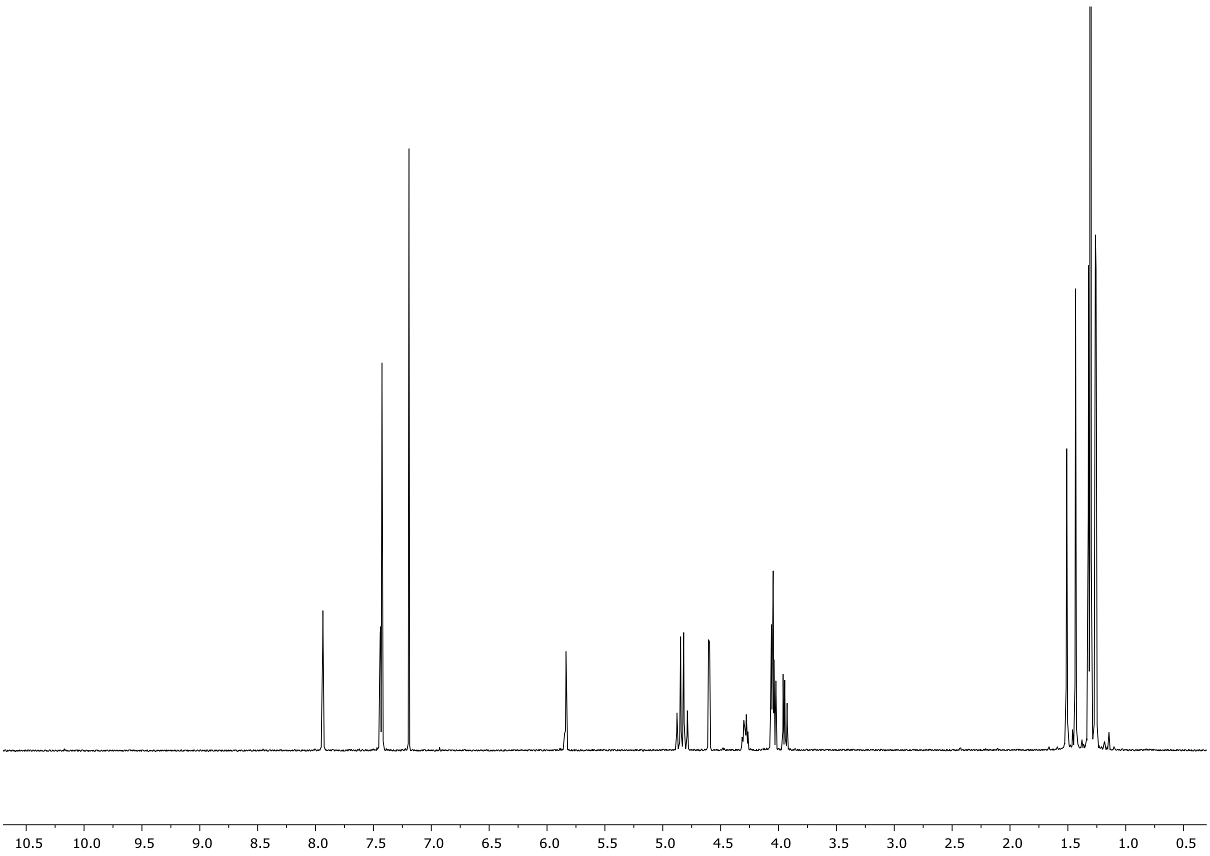

Figure S30. ^{1}H NMR (400 MHz, $CDCl_3$, 298 K) (D)-**S12**.

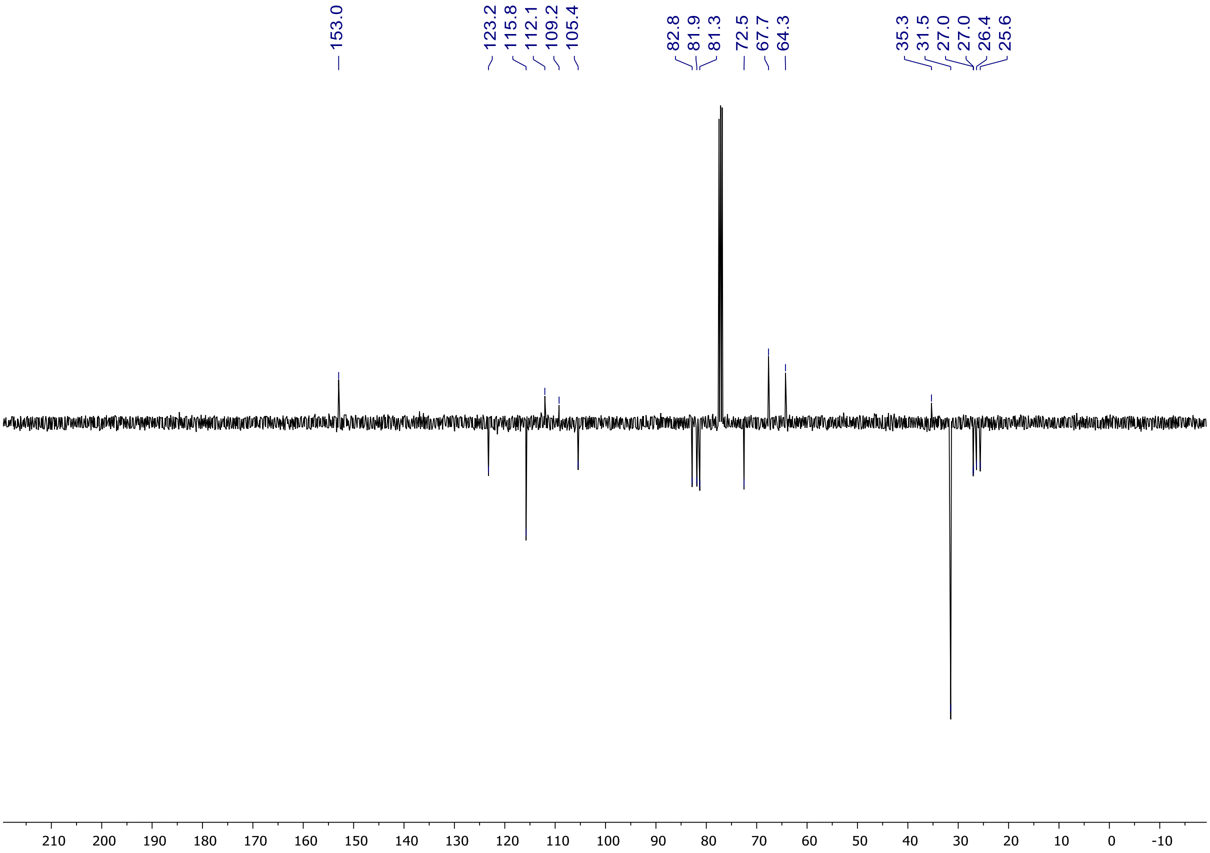

Figure S31. ^{13}C NMR (126 MHz, $CDCl_3$, 298 K) (D)-**S12**, following purification by chromatography.

Rotaxane (D,R_{mp}/S_{mp})-**S13**

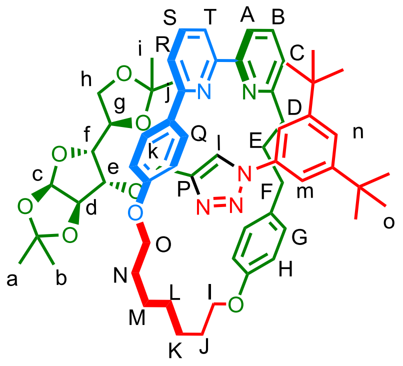

(D,S_{mp})-**S13**

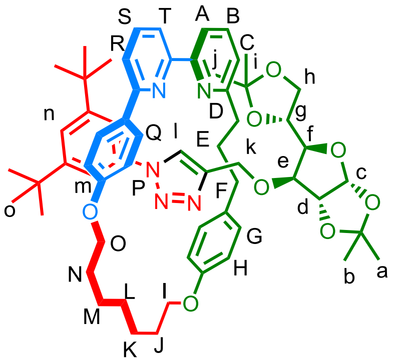

(D,R_{mp})-**S13**

A dry sealed vessel was charged with **2b** (11.2 mg, 0.0375 mmol), **3b** (8.7 mg, 0.0375 mmol), macrocycle **1** (12.0 mg, 0.025 mmol), [Cu(MeCN)$_4$]PF$_6$ (8.8 mg, 0.023 mmol), DIPEA (40 µL, 0.25 mmol), and anhydrous CH_2Cl_2 (0.63 mL). The reaction mixture was stirred at rt for 16 h, protected by an argon atmosphere. Saturated EDTA-NH_3 (10 mL) was added, and the aqueous layer was extracted with $CHCl_3$ (3 × 20 mL), dried over $MgSO_4$, filtered, and had the solvent removed *in vacuo*. The residue containing rotaxanes (D,R_{mp}/S_{mp})-**S13** (in a 0.50 : 0.50 diastereomeric ratio by ^{1}H NMR, **Figure S32**) was purified by chromatography (1 : 1 petrol CH_2Cl_2 with 0→15% EtOAc), to yield (D, R_{mp}/S_{mp})-**S13** as a yellow oil (15.1 mg, 60%, 0.50 : 0.50 diastereoisomeric ratio, **Figure S33**); ^{1}H NMR (400 MHz, CDCl$_3$, 298 K)* δ 9.99 (s, 1H, H_I), 9.94 (s, 1H, $H_{I'}$), 7.82-7.73 (m, 1H, H_B and $H_{B'}$), 7.71 (t, 1H, *J* = 7.8, H_S or $H_{S'}$), 7.70-7.60 (m, 2H, H_A, $H_{A'}$ and H_S or $H_{S'}$), 7.58-7.49 (m, 1H, H_T and $H_{T'}$), 7.50 (d, 2H, *J* = 1.7, $H_{m'}$), 7.38 (d, 1H, *J* = 1.7, H_m), 7.34 (d, 1H, *J* = 7.8, H_R or $H_{R'}$), 7.30-7.22 (m, 2H, H_C, $H_{C'}$ and H_R or $H_{R'}$), 7.17 (t, 1H, *J* = 1.7, $H_{n'}$), 7.13 (t, 1H, *J* = 1.8, H_n), 7.01-6.90 (m, 4H, H_Q and $H_{Q'}$), 6.52-6.43 (m, 1H, H_G and $H_{G'}$), 6.33-6.22 (m, 2H, H_P and $H_{P'}$), 6.11 (s, 2H, H_H and $H_{H'}$), 5.45 (d, 1H, *J* = 3.6, $H_{c'}$), 5.33 (d, 1H, *J* = 3.6, H_c), 4.52 (d, 1H, *J* = 11.1, one of H_k), 4.47 (d, 1H, *J* = 11.9, one of $H_{k'}$), 4.40-4.30 (m, 2H, H_d, $H_{d'}$ and one of H_k), 4.26 (d, 1H, *J* = 11.9, one of $H_{k'}$), 4.24-4.04 (m, 3H, H_g, $H_{g'}$, one of H_I, one of H_I, one of H_O and one of $H_{O'}$), 4.04-3.82 (m, 5H, H_e, $H_{e'}$, H_f, $H_{f'}$, one of H_I, one of $H_{I'}$, one of H_O, one of $H_{O'}$,one of H_h and one of $H_{h'}$), 3.82-3.71 (m, 1H, one of H_h and one of $H_{h'}$), 2.90-2.74 (m, 1H, one of H_D and one of $H_{D'}$), 2.73-2.59 (m, 1H, one of H_D and one of $H_{D'}$), 2.60-2.48 (m, 1H, one of H_F and one of $H_{F'}$), 2.48-2.33 (m, 1H, one of H_F and one of $H_{F'}$), 2.25-2.09 (m, 1H, one of H_L and one of $H_{L'}$), 2.07-1.64 (m, 7H, H_E, $H_{E'}$, H_J, $H_{J'}$, one of H_M, one of $H_{M'}$, H_N and $H_{N'}$), 1.64-1.46 (m, 2H, H_L, $H_{L'}$, one of H_M and one of $H_{M'}$), 1.42 (s, 3H, $H_{J'}$), 1.38 (m, 6H, H_b and $H_{b'}$), 1.33 (s, 3H, H_j), 1.24 (s, 3H, $H_{i'}$), 1.17 (s, 3H, $H_{a'}$), 1.14 (s, 18H, $H_{o'}$), 1.12 (s, 18H, H_o), 1.11 (s, 3H, H_i), 1.04 (s, 3H, H_a); ^{13}C NMR (126 MHz, CDCl$_3$, 298 K) δ 163.7, 163.6, 159.8, 159.8, 159.1, 159.1, 157.6, 157.6, 157.6, 157.3, 157.0, 156.9, 150.9, 150.8, 142.9, 142.7, 137.3, 137.0, 137.0, 136.9, 136.8, 131.7, 131.4, 131.2, 128.6, 128.6, 128.3, 128.0, 124.7, 124.4, 122.8, 122.6, 120.5, 120.4, 120.2, 120.2, 119.9, 119.7, 119.7, 119.6, 114.3, 114.1, 2 × 113.7, 113.6, 113.6, 111.7, 111.5, 109.0, 108.9, 105.3, 105.3, 83.0, 82.7, 82.1, 81.8, 2 × 81.5, 72.7, 72.4, 67.4, 67.3, 2 × 67.2, 2 × 65.5, 65.1, 64.7, 37.6, 37.6, 2 × 35.4, 35.0, 35.0, 33.4, 33.2, 31.4, 31.4, 29.6, 29.5, 29.1, 29.1, 2 × 28.2, 27.1, 27.1, 26.9, 26.9, 26.5, 26.2, 25.8, 25.7, 25.5, 25.2; LR-ESI-MS (+ve) *m/z* = (%) 1008.6 [M+H]$^+$, calc. 1009.0.

*The signals arising from the two diastereomers designated H_X and $H_{X'}$. Careful examination of 2D NMR data allowed the signals from each diastereomer to grouped accurately within the axle and macrocycle but not between them. Proton counts are provided for each signal and represent the expected integration of that environment. Where the signals of both diastereoisomers are coincident, no H_X/$H_{X'}$ label is provided and the proton count indicated refers the expected integration of that signal in each of the stereoisomers that contributes to the multiplet.

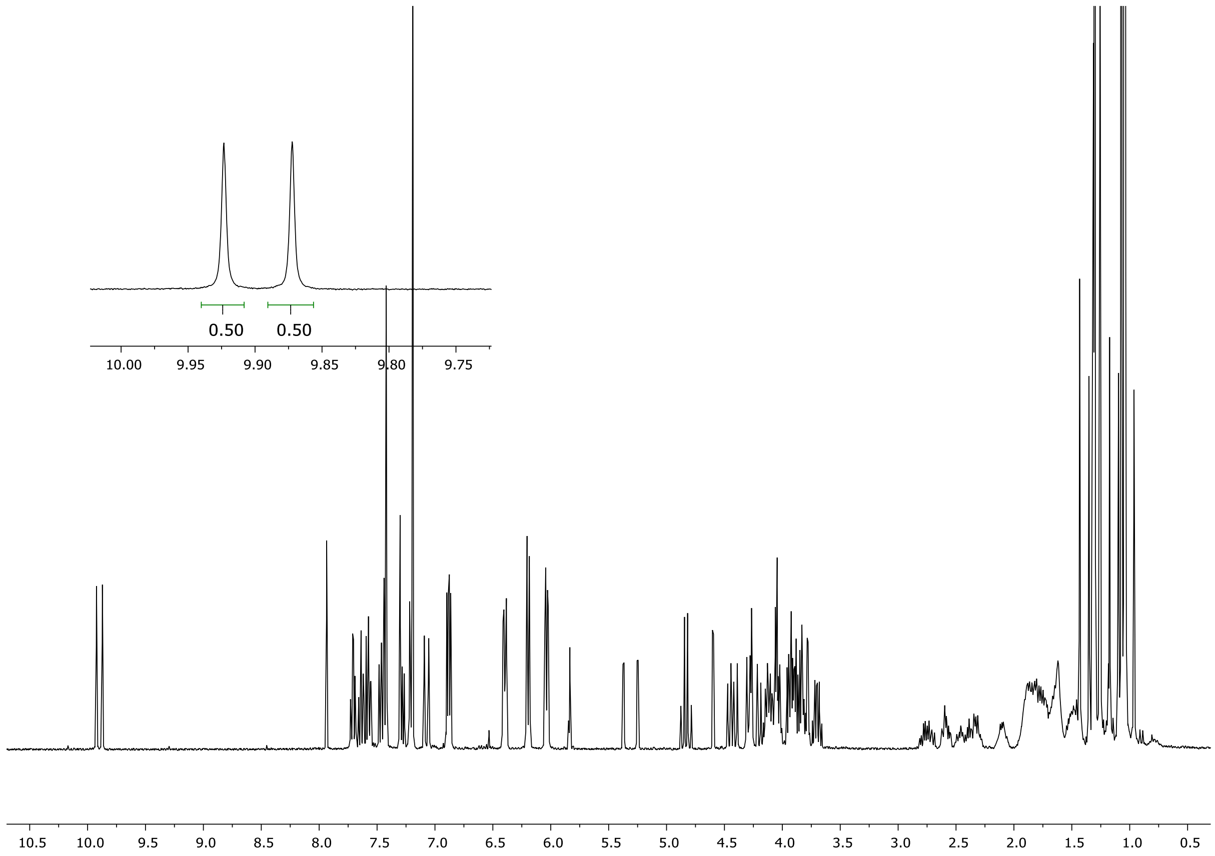

Figure S32. ^{1}H NMR (400 MHz, $CDCl_3$, 298 K) (D, R_{mp}/S_{mp})-**S13** prior to purification by chromatography.

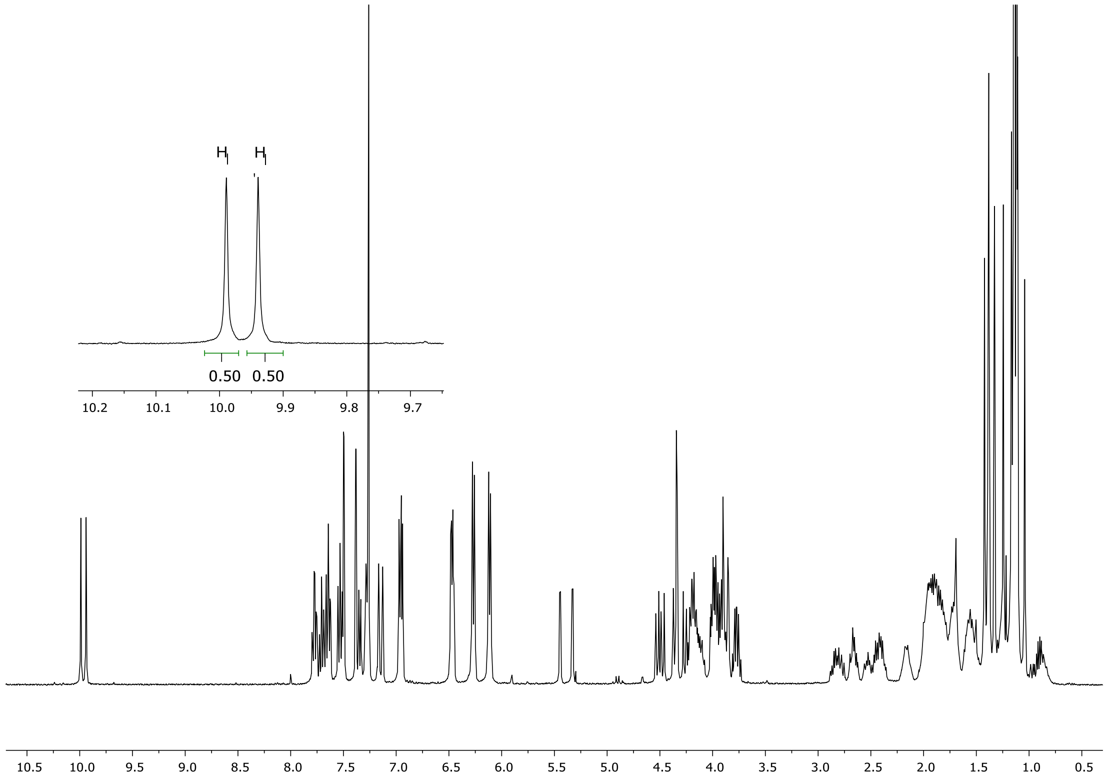

Figure S33. ^{1}H NMR (500 MHz, $CDCl_3$, 298 K) (D,R_{mp}/S_{mp})-**S13**, following purification by chromatography.

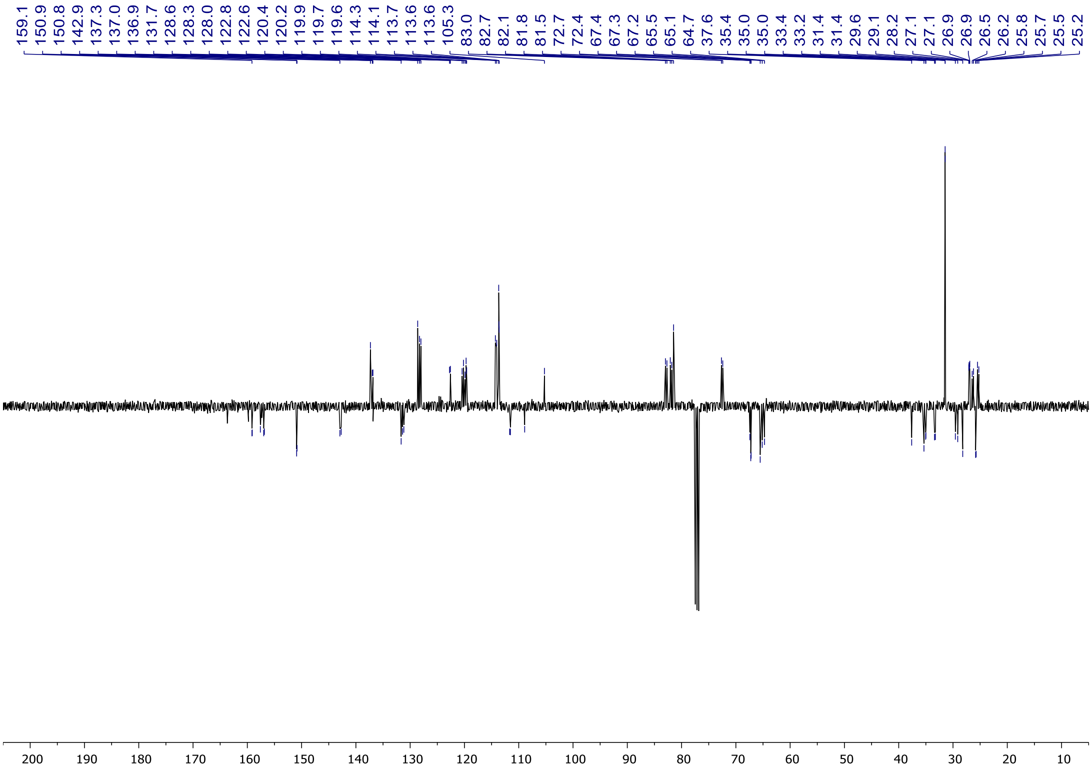

Figure S34. ^{13}C NMR (126 MHz, $CDCl_3$, 298 K) (D,R_{mp}/S_{mp})-**S13**, following purification by chromatography.

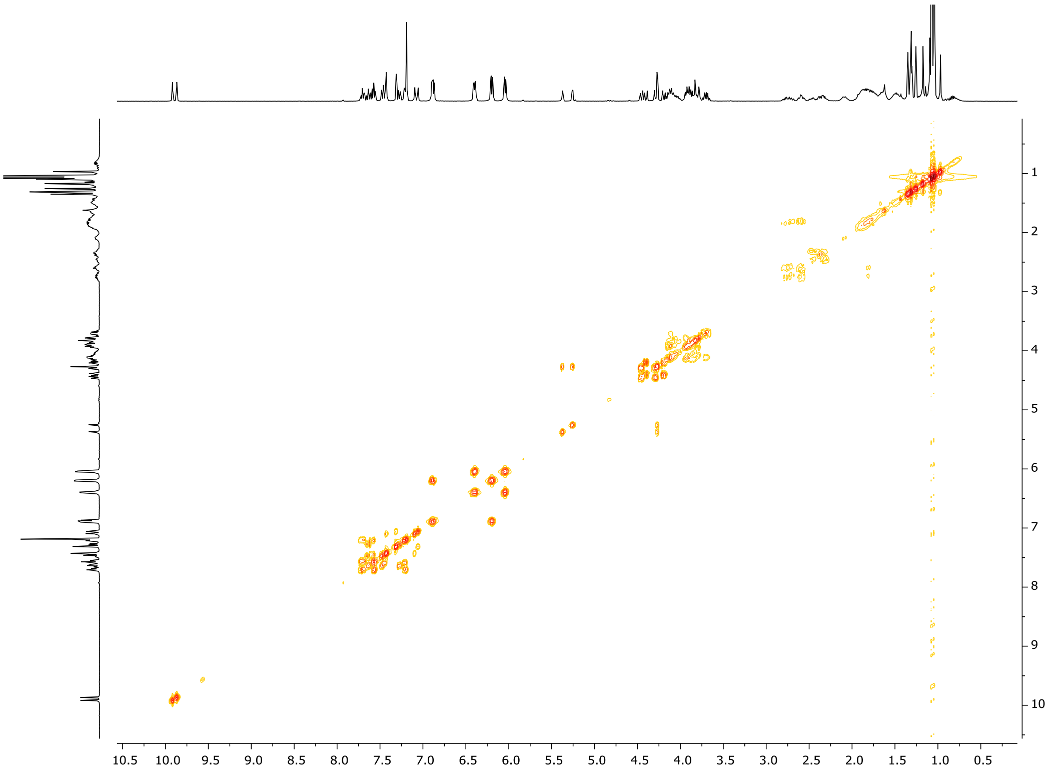

Figure S35. ^{1}H-^{1}H COSY NMR (126 MHz, $CDCl_3$, 298 K) (D,R_{mp}/S_{mp})-**S13**, following purification by chromatography.

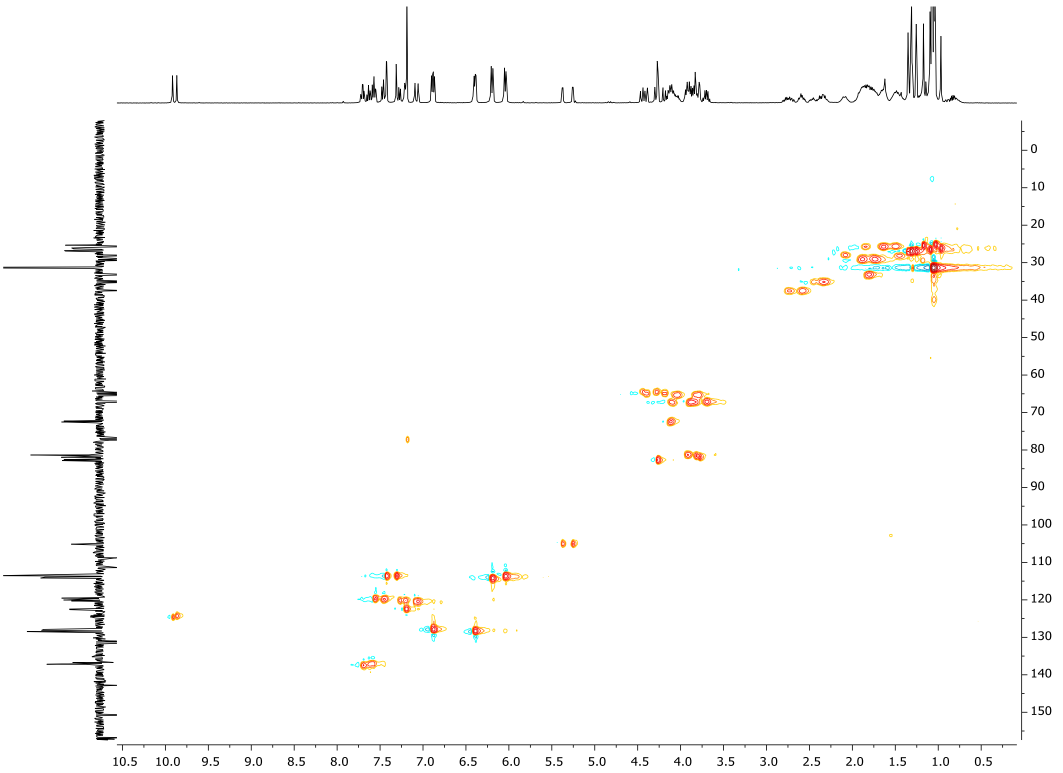

Figure S36. ^{1}H-^{13}C HSQC NMR (126 MHz, $CDCl_3$, 298 K) (D,R_m/S_{mp})-**S13**, following purification by chromatography.

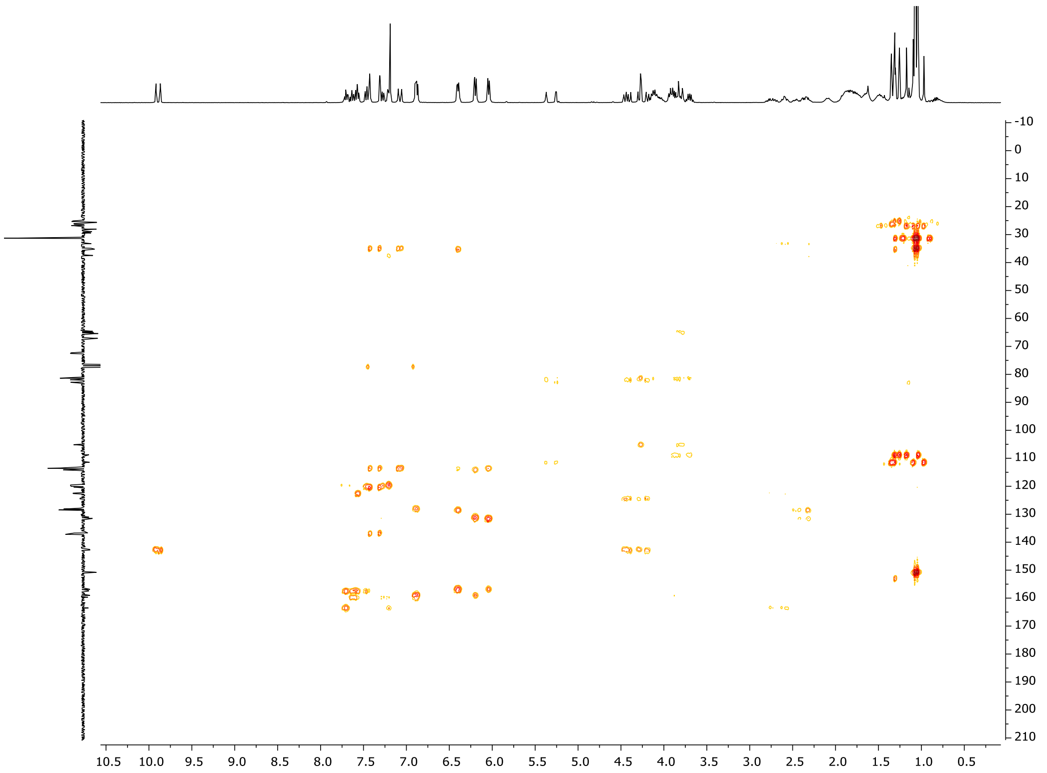

Figure S37. ^{1}H-^{13}C HMBC NMR (126 MHz, $CDCl_3$, 298 K) (D,R_{mp}/S_{mp})-**S13**, following purification by chromatography.

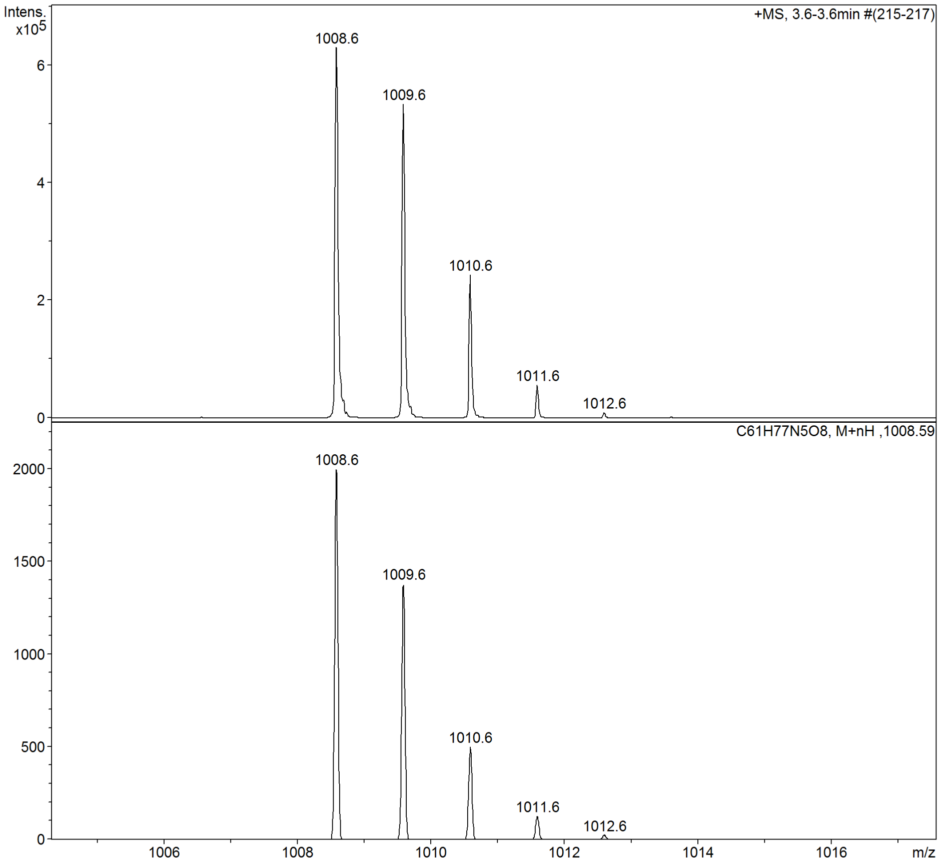

Figure S38. ESI-MS isotopic pattern of (D, R_{mp}/S_{mp})-**S13**; observed (top) and calculated (bottom).

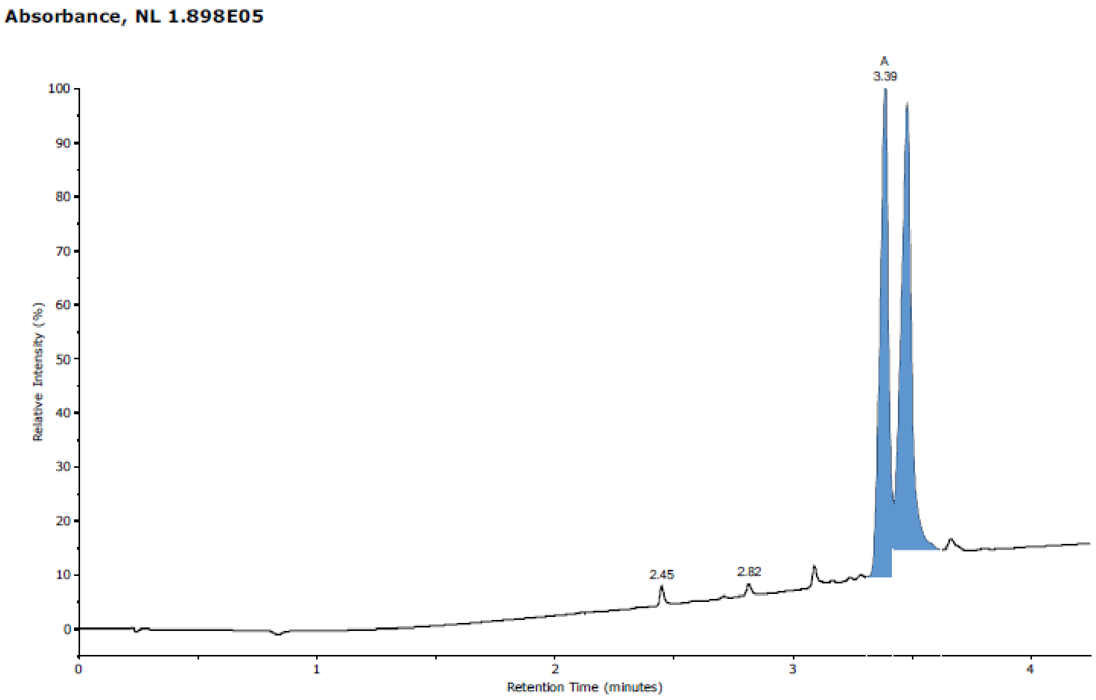

Figure S39. LCMS trace (C18 column, gradient 5 minutes (1 : 4 MeCN+0.2% formic acid-H_2O +0.2% formic acid →1 : 0 MeCN-H_2O +0.2% formic acid), UV 254 nm), of (D,R_{mp}/S_{mp})-**S13** following purification by chromatography.

Entry 3 – axle (*R*/*S*)-S14 and rotaxane (*R*/*S*)-S15 derived from alkyne 2c and azide 3b

Axle (*R*/*S*)-**S14**

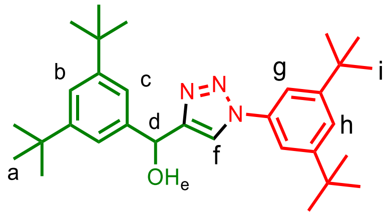

A 10 mL round bottom flask was charged with (*R*/*S*)-**2c** (24.4 mg, 0.1 mmol), **3b** (23.1 mg, 0.1 mmol), $CuSO_4.5H_2O$ (12.5 mg, 0.05 mmol) and sodium *L*-ascorbate (19.8 mg, 0.1 mmol), in DMF (2 mL). The reaction mixture was stirred at rt for 16 h. The reaction mixture was diluted with Et_2O (10 mL), washed with H_2O (2 × 10 mL), brine (5 mL), dried over $MgSO_4$, filtered, and the solvent removed *in vacuo*. The residue was purified by column chromatography (petrol with 0→50% Et_2O) to yield axle (*R*/*S*)-**S14** as white foam (37.0 mg, 78%); ^{1}H NMR (400 MHz, $CDCl_3$, 298 K) δ 7.74 (s, 1H, H_e), 7.48 (t, 1H, *J* = 1.7, H_h), 7.47 (d, 2H, *J* = 1.7, H_g) 7.40 (t, 1H, *J* = 1.7, H_b), 7.38 (d, 2H, *J* = 1.7, H_c), 6.11 (d, 1H, *J* = 3.7, H_d), 2.85 (d, 1H, *J* = 3.7, H_e), 1.35 (s, 18H, H_i), 1.33 (s, 18H, H_a); ^{13}C NMR (101 MHz, $CDCl_3$, 298 K); δ 152.9, 151.9, 151.3, 141.2, 136.9, 123.1, 122.4, 121.0, 120.0, 115.7, 70.3, 35.3, 35.1, 31.6, 31.6; HR-ESI-MS (+ve) *m/z* = 476.3624 $[M+H]^+$ calc. 476.3635.

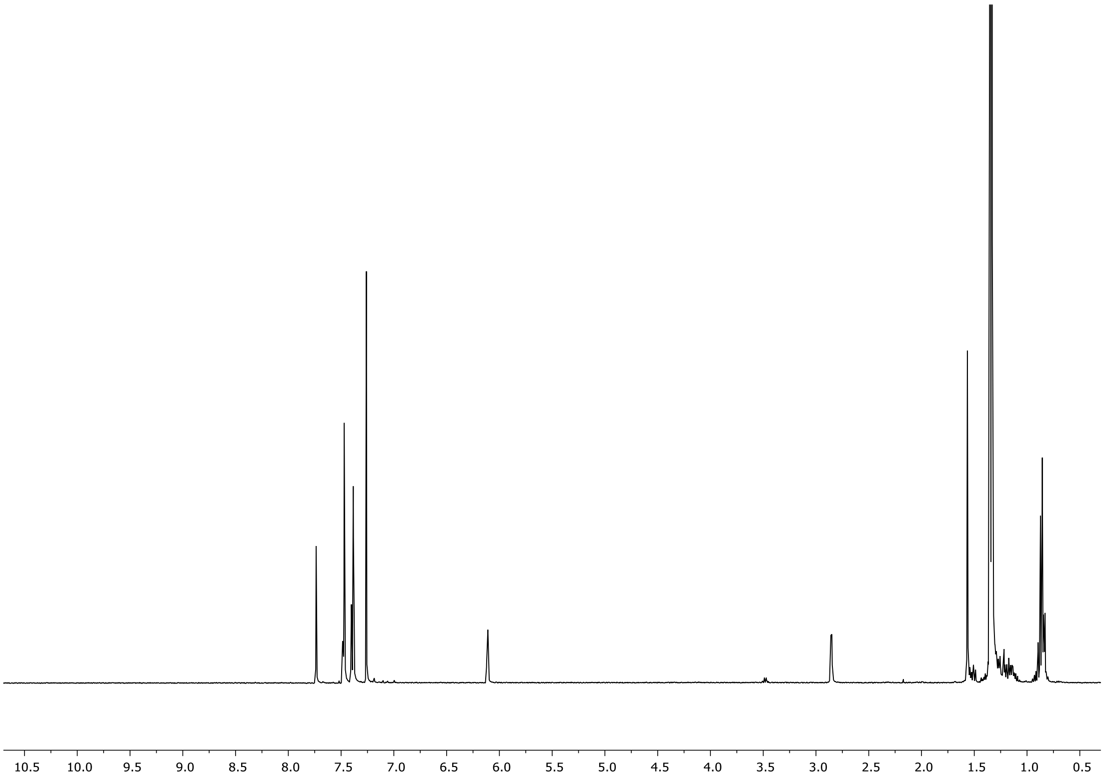

Figure S40. ^{1}H NMR (400 MHz, $CDCl_3$, 298 K) (*R*/*S*)-**S14**.

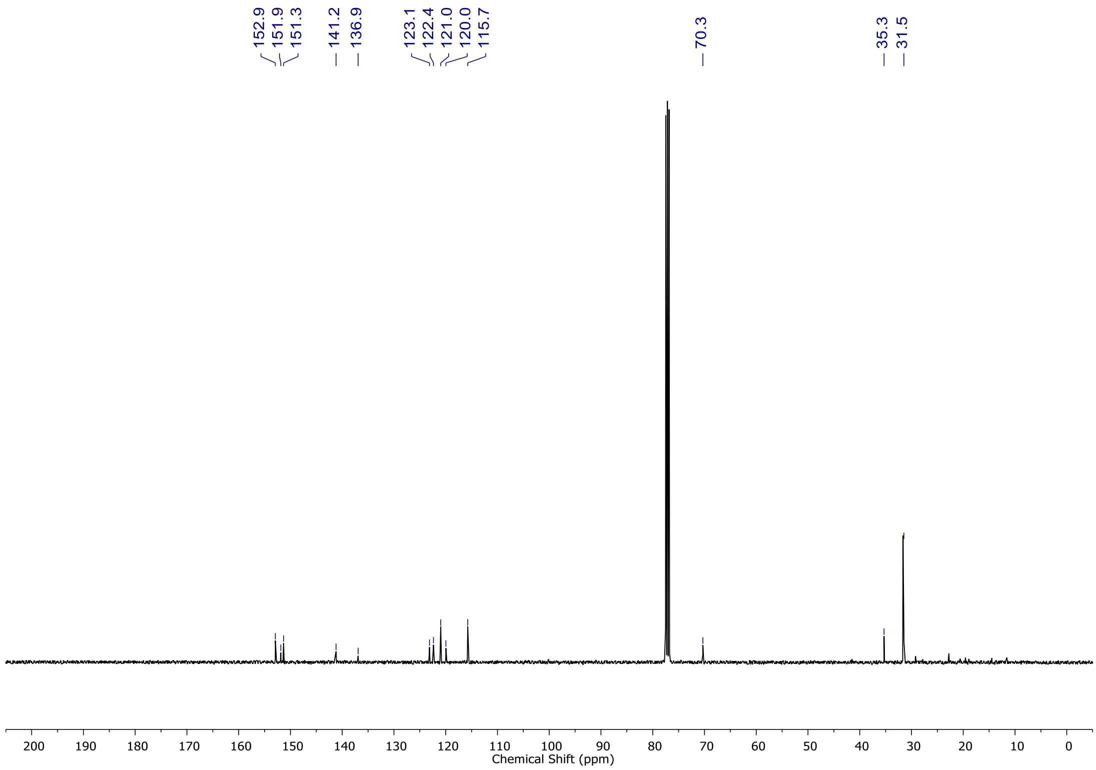

Figure S41. ^{13}C NMR (101 MHz, $CDCl_3$, 298 K) (*R*/*S*)-**S14**, following purification by chromatography.

Rotaxanes (R/S,R_{mp}/S_{mp})-**S15**

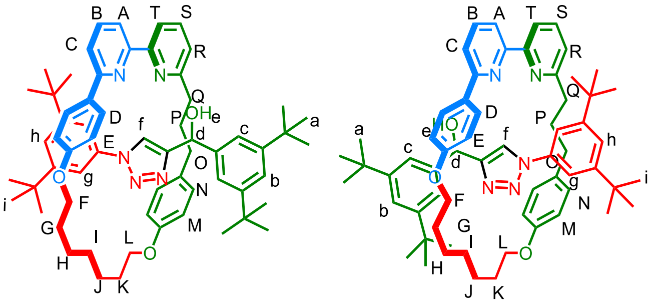

A dry sealed vessel was charged with (R/S)-**2c** (6.1 mg, 0.025 mmol), **3b** (5.8 mg, 0.025 mmol), macrocycle **1** (12.0 mg, 0.025 mmol), $[Cu(MeCN)_4]PF_6$ (8.9 mg, 0.024 mmol), DIPEA (9.0 µL, 0.05 mmol), and anhydrous CH_2Cl_2 (2 mL). The reaction mixture was stirred at rt for 16 h, protected by a nitrogen atmosphere. KCN (20.0 mg, 0.307 mmol) was added and the reaction mixture was stirred for an additional 16 h. Air was bubbled though the reaction to remove the solvent and the residue was re-dissolved in CH_2Cl_2 (20 mL) and had H_2O (10 mL) added. The aqueous layer was extracted with CH_2Cl_2 (2 × 10 mL), washed with brine (10 mL), dried over $MgSO_4$, filtered, and had the solvent removed *in vacuo*. The residue containing rotaxanes (R/S,R_{mp}/S_{mp})-**S15** (in a 0.65 : 0.35 diastereoisomeric ratio by ^{1}H NMR, **Figure S42**) was purified by chromatography (1 : 1 CH_2Cl_2-petrol with 0→20% MeCN), to yield rotaxanes (R/S,R_{mp}/S_{mp})-**S15** as a yellow foam (9.3 mg, 40%, 0.57 : 0.43 diastereoisomeric ratio **Figure S43**); ^{1}H NMR (400 MHz, $CDCl_3$, 298 K)* δ 9.88 (s, 1H, H_f(*major*)), 9.79 (s, 1H, H_f(*minor*)), 7.80 (app. q, 1H, J = 7.6, H_S), 7.74-7.67 (m, 1H, H_B), 7.65 (d, 1H, J = 7.8, H_T), 7.59-7.55 (m, 1H, H_A), 7.47 (d, 2H, J = 1.7, H_c (*major*)), 7.41 (d, 2H, J = 1.7, H_c (*minor*)), 7.36-7.21 (m, 5H, H_h (*major*), H_g, H_C, and H_R), 7.21 (t, 1H, J = 1.8, H_h (*minor*)), 7.15-7.13 (m, 1H, H_b), 6.79 (d, 2H, J = 8.7, H_E (*major*)), 6.61 (d, 2H, J = 8.7, H_E (*minor*)), 6.28 (d, 2H, J = 8.5, H_M (*minor*)), 6.16-6.09 (m, 4H, H_D and H_M (*major*)), 5.98-5.92 (m, 2H, H_N), 5.52 (br s, 1H, H_d (*minor*)), 5.42 (d, 1H, J = 3.1, H_d (*major*)), 4.23-4.07 (m, 2H, one of H_F and one of H_L) 3.99-3.90 (m, 1H, one of H_F or one of H_L), 3.81-3.71 (m, 1H, one of H_F or one of H_L), 3.10 (m, 1H, H_e (*minor*)), 2.92-2.46 (m, 3H, H_e (*major*) and H_Q) 2.45-2.18 (m, 3H, one of H_I and H_O), 2.08-1.15, (m, 11H, H_G, H_H, one of H_I, H_J, H_K and H_P), 1.18 (m, 18H, H_a or H_i), 1.14 (m, 18H, H_a (*minor*) or H_i (*minor*)), 1.13 (m, 18H, H_a (*major*) or H_i (*major*)); ^{13}C NMR (101 MHz, $CDCl_3$, 298 K) δ 2 × 163.7, 159.8, 159.7, 159.1, 159.0, 157.8, 2 × 157.7, 157.6, 2 × 156.8, 150.9, 150.8, 150.4, 150.2, 2 × 149.5, 148.9, 142.4, 2 × 137.3, 137.1, 2 × 137.0, 2 × 131.5, 131.3, 131.2, 128.7, 128.6, 128.1, 128.0, 122.8, 122.7, 122.0, 121.6, 120.6, 120.5, 120.4, 120.2, 120.1, 2 × 119.9, 2 × 119.8, 114.5, 114.4, 113.8, 113.6, 113.6, 113.5, 70.9, 70.3, 70.2 67.6, 65.6, 65.5, 37.7, 37.6, 35.2, 35.1, 35.0, 34.9, 33.4, 33.3, 31.6, 2 × 31.5, 29.9, 2 × 29.4, 29.2, 28.2, 25.8, 2 × 25.7; HR-ESI-MS (+ve) m/z = 954.6258 $[M+H]^+$ calc. 954.6256.

*As stereochemistry could not be unambiguously assigned, the signals are simply designated (*major*) or (*minor*). Proton counts are provided for each signal and represent the expected integration of that environment. Where the major and minor diastereoisomer signals are coincident, no (*major*)/(*minor*) label is provided and the proton count indicated refers the expected integration of that signal in each of the stereoisomers that contributes to the multiplet.

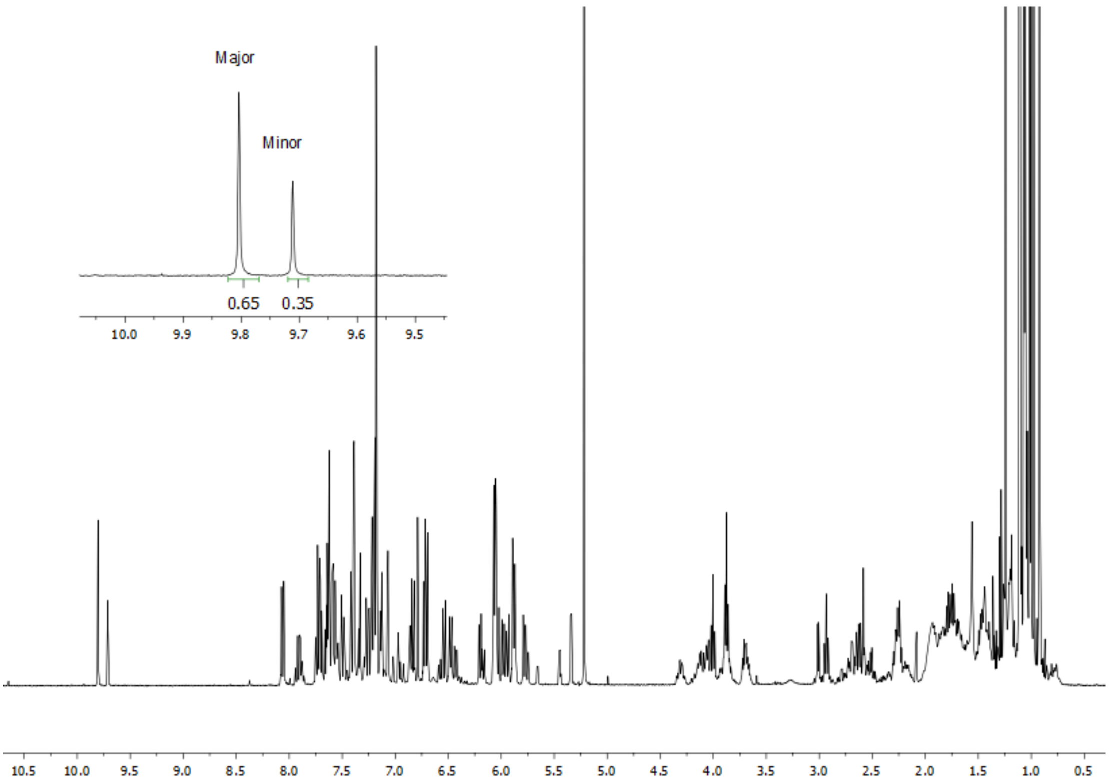

Figure S42. ^{1}H NMR (400 MHz, $CDCl_3$, 298 K) ($R/S,R_{mp}/S_{mp}$)-**S15** prior to purification by chromatography.

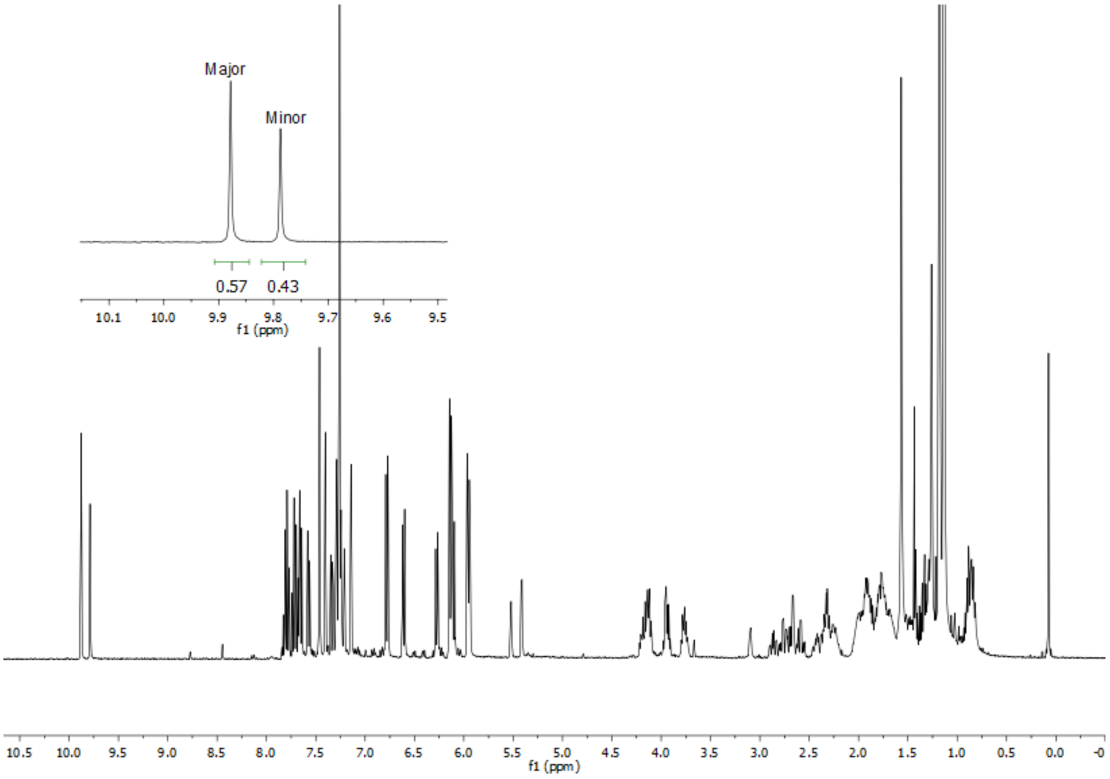

Figure S43. ^{1}H NMR (400 MHz, $CDCl_3$, 298 K) ($R/S,R_{mp}/S_{mp}$)-**S15,** following purification by chromatography.

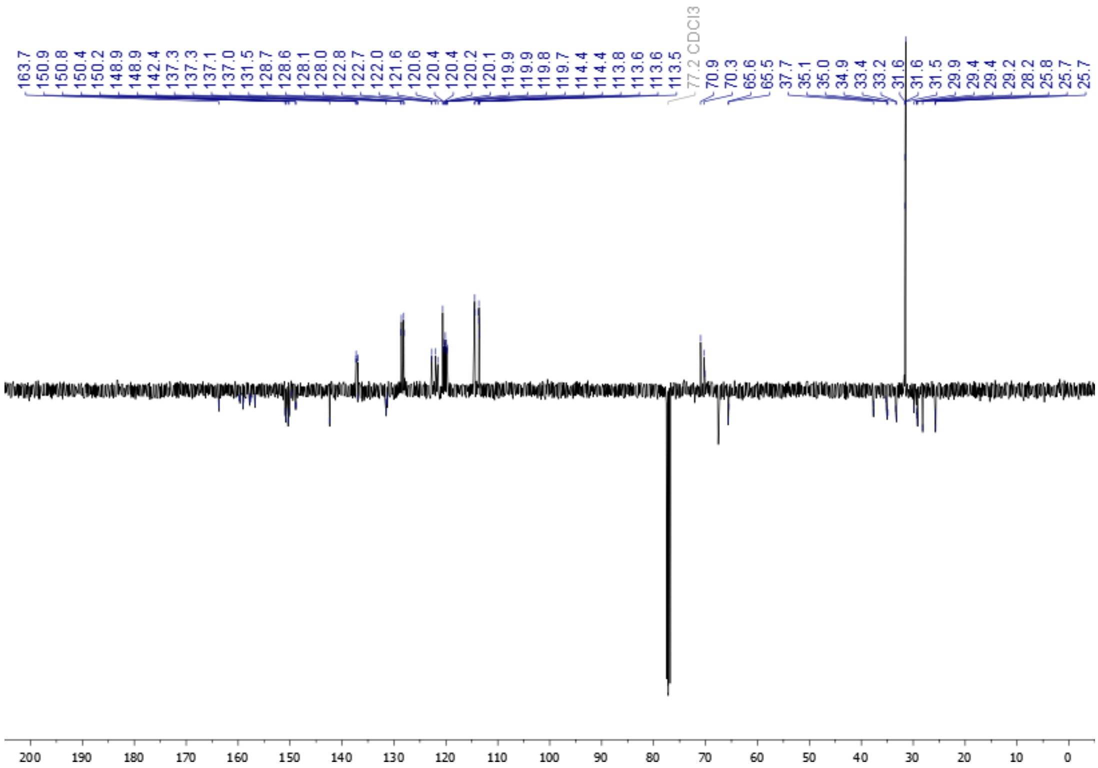

Figure S44. ^{13}C NMR (126 MHz, $CDCl_3$, 298 K) (*R*/*S*,*R*$_{mp}$/*S*$_{mp}$)-**S15**, following purification by chromatography.

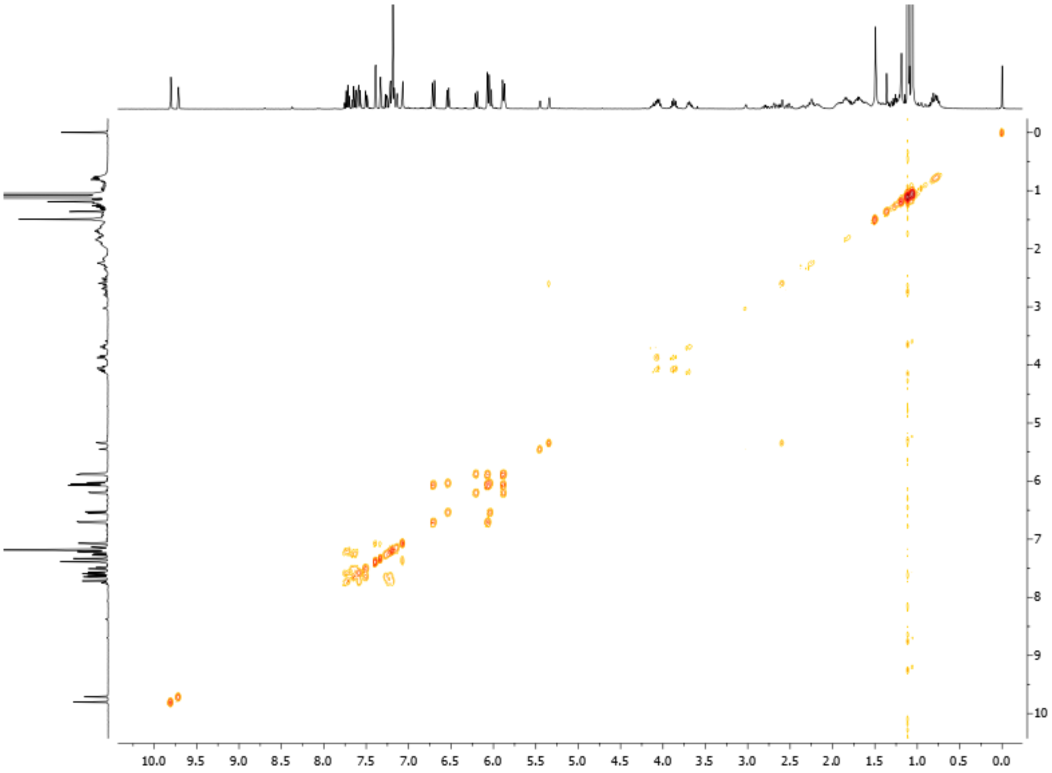

Figure S45. ^{1}H-^{1}H COSY NMR (500 MHz, $CDCl_3$, 298 K) (*R*/*S*,*R*$_{mp}$/*S*$_{mp}$)-**S15**, following purification by chromatography.

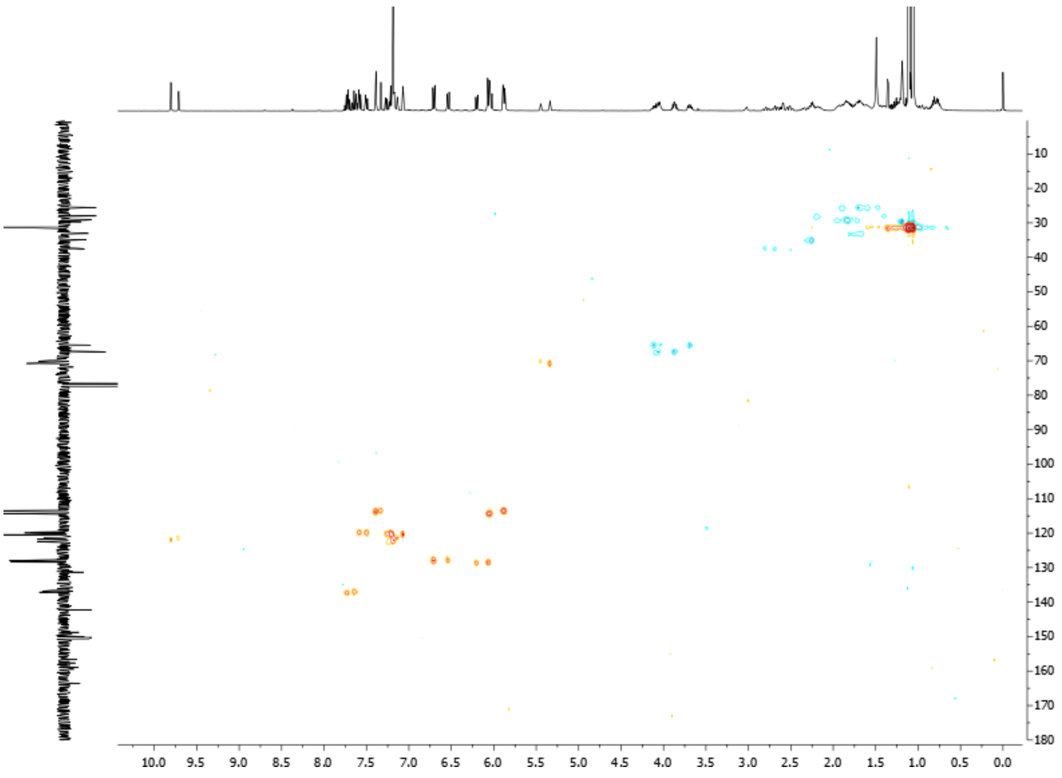

Figure S46. ^{1}H-^{13}C HSQC NMR (126 MHz, $CDCl_3$, 298 K) (*R*/*S*,*R*$_{mp}$/*S*$_{mp}$)-**S15**, following purification by chromatography.

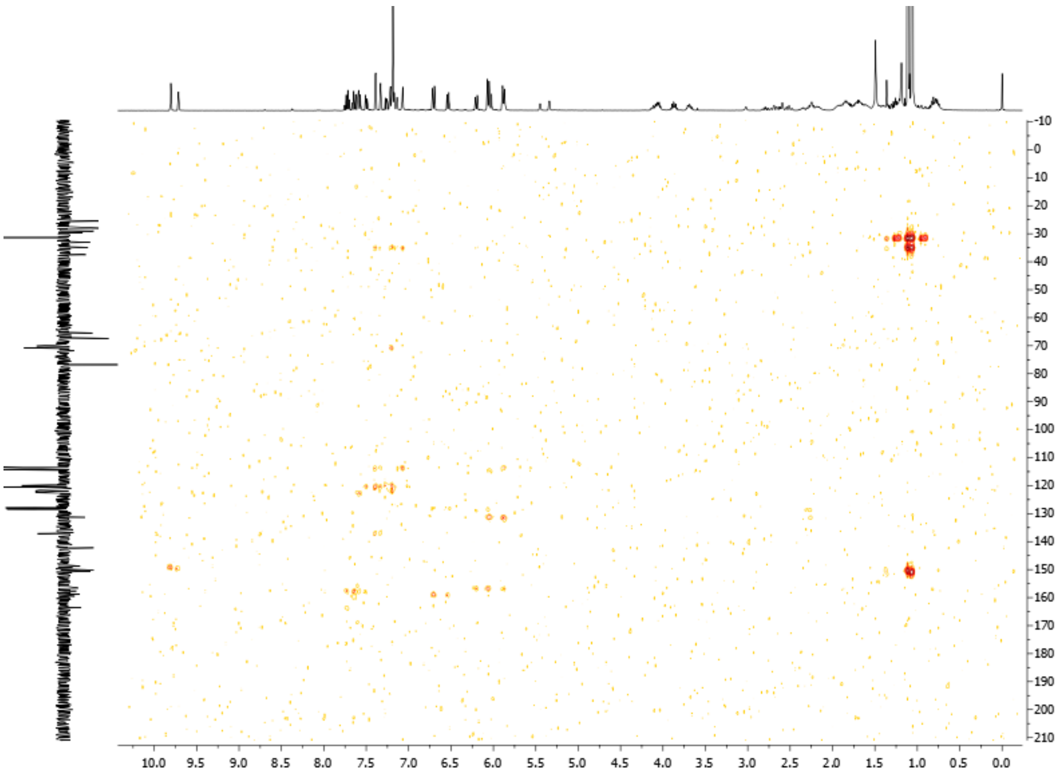

Figure S47. ^{1}H-^{13}C HMBC NMR (126 MHz, $CDCl_3$, 298 K) (*R*/*S*,*R*$_{mp}$/*S*$_{mp}$)-**S15**, following purification by chromatography.

Entry 4 – axle (*S*)-S16 and rotaxane (*S*,*R*$_{mp}$/*S*$_{mp}$)-S17 derived from alkyne 2a and azide 3c

Axle (*S*)-**S16**

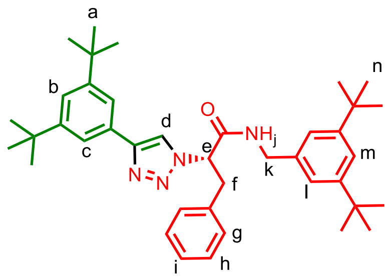

A 10 mL round bottom flask was charged with **2a** (7.6 mg, 0.035 mmol), (*S*)-**3c** (14 mg, 0.035 mmol), sodium *L*-ascorbate (11.1 mg, 0.056 mmol) and $CuSO_4.5H_2O$ (8.6 mg, 0.035 mmol), in DMF (2 mL). The reaction mixture was stirred at rt for 16 h. EtOAc (20 mL) was added and extracted with EDTA-NH_3 solution (15 mL) and 5% w/v LiCl solution (15 mL). The organic layer was dried over $MgSO_4$, filtered, and the solvent was removed *in vacuo*. The residue was purified by chromatography (ethyl acetate with 0→5% CH_2Cl_2), to yield (*S*)-**S16** as yellow foam (13.2 mg, 62%);^{1}H NMR (400 MHz, $CDCl_3$, 298 K) δ 7.82 (s, 1H, H_d), 7.61 (d, 2H, *J* = 1.8, H_c), 7.42 (t, 1H, *J* = 1.8, H_b), 7.32 (t, 1H, *J* = 1.8, H_m), 7.24-7.19 (m, 3H, H_h and H_i), 7.14-7.10 (m, 2H, H_g), 6.98 (d, 2H, *J* = 1.8, H_l), 6.58 (t, 1H, *J* = 5.5, H_j), 5.34-5.28 (dd, 1H, *J* = 9.1, 6.3, H_e), 4.45 (dd, 1H, *J* = 14.6, 5.6, one of H_k), 4.37 (dd, 1H, *J* = 14.6, 5.6, one of H_k), 3.73-3.65 (dd, 1H, *J* = 13.8, 6.3, one of H_f), 3.53 (dd, 1H, *J* = 13.8, 9.1, one of H_f), 1.36 (s, 18H, H_a or H_n), 1.27 (s, 18H, H_a or H_n); ^{13}C NMR (101 MHz, $CDCl_3$, 298 K) δ 167.4, 151.5, 151.5, 149.0, 136.2, 135.8, 129.4, 129.1, 129.0, 127.5, 122.8, 122.0, 122.0, 120.3, 120.2, 66.9, 44.6, 39.5, 35.1, 34.9, 31.6, 31.5. HR-ESI-MS (+ve) *m/z* = 607.4369 [M+H] calc. 607.4370.

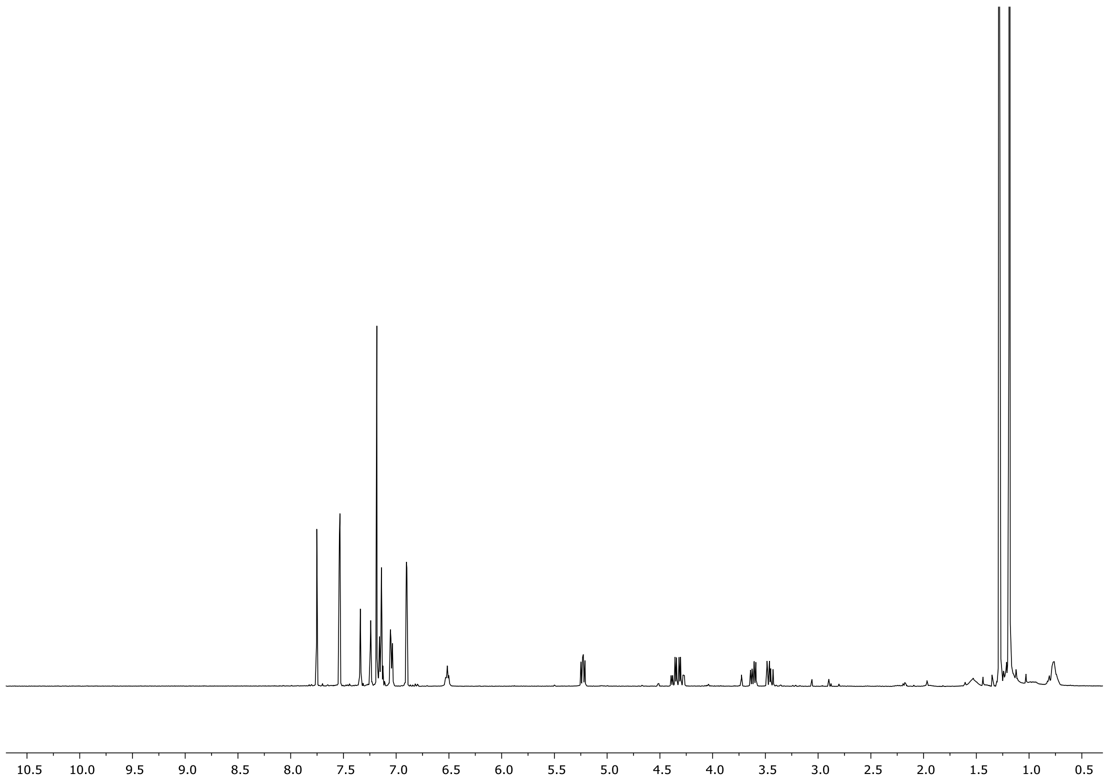

Figure S48. ^{1}H NMR (400 MHz, $CDCl_3$, 298 K) (*S*)-**S16**.

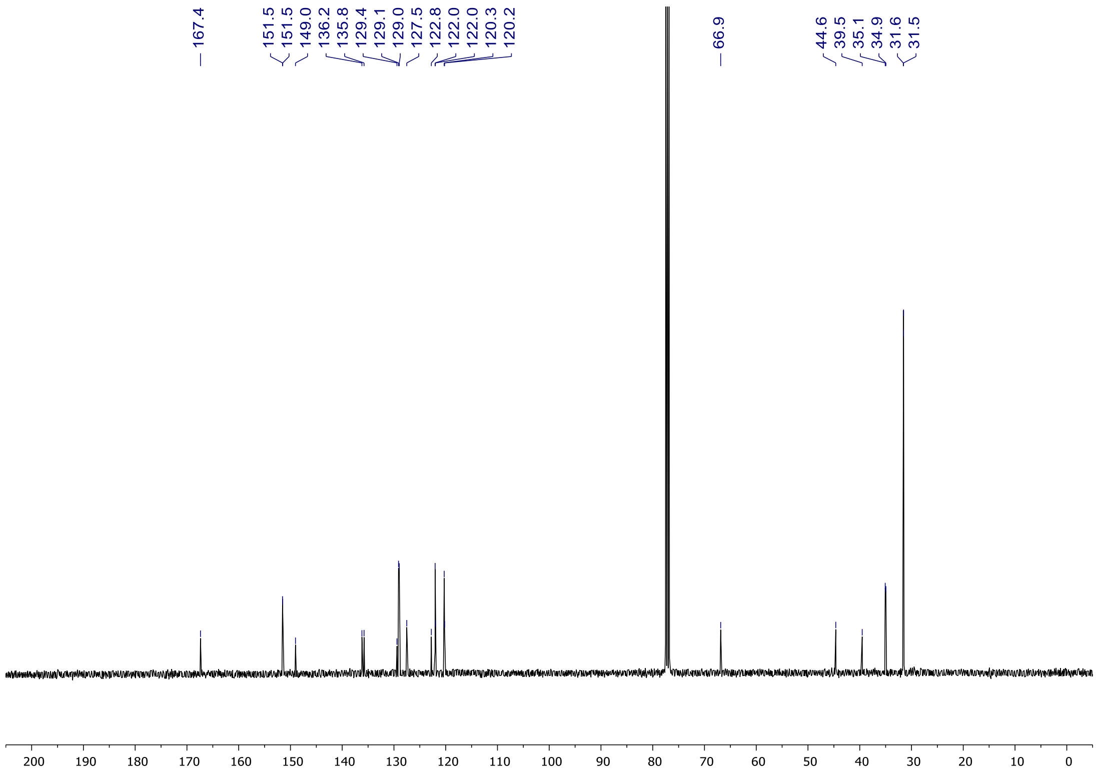

Figure S49. ^{13}C NMR (400 MHz, $CDCl_3$, 298 K) (*S*)-**S16**.

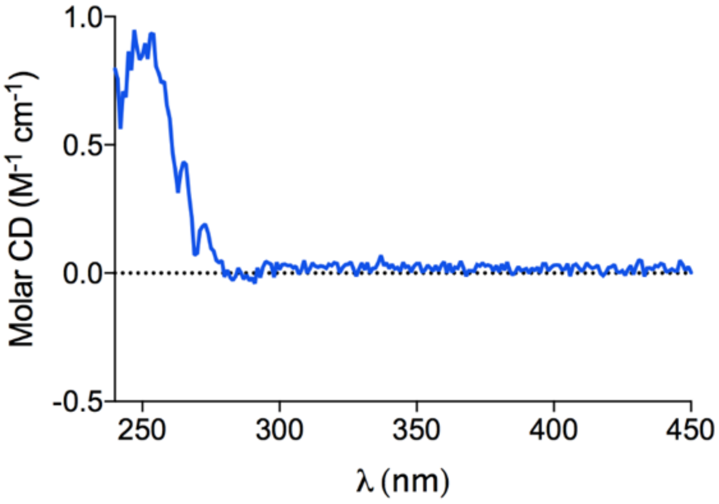

Figure S50. Circular dichroism spectrum of (*S*)-**S16** (72.5 µM in $CHCl_3$).

Rotaxane (*S*,*R*$_{mp}$/*S*$_{mp}$)-**S17**

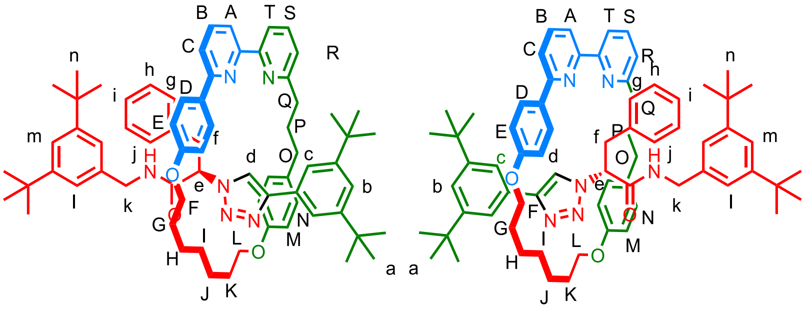

(*S*,*R*$_{mp}$)-**S17** (*S*,*S*$_{mp}$)-**S17**

A dry sealed vessel was charged with **2a** (19.7 mg, 0.092 mmol), (*S*)-**3c** (36.1 mg, 0.092 mmol), macrocycle **1** (22.3 mg, 0.046 mmol), [Cu(MeCN)$_4$]PF$_6$ (16.4 mg, 0.044 mmol), DIPEA (32 µL, 0.184 mmol), in anhydrous CH_2Cl_2 (1.1 mL). The reaction mixture was stirred at rt for 16 h, protected by an argon atmosphere. TFA (0.10 mL, 1.30 mmol) was added and the reaction mixture was stirred for an additional 16 h. Saturated EDTA-NH_3 solution (10 mL) was added, and the aqueous layer was extracted with $CHCl_3$ (3 × 30 mL), dried over $MgSO_4$, filtered, and had the solvent removed *in vacuo*. The residue containing rotaxanes (*S*,*R*$_{mp}$/*S*$_{mp}$)-**S17** (in a 0.15 : 0.85 diastereoisomeric ratio by ^{1}H NMR, **Figure S51**) was purified *via* preparative TLC (98 : 2 CH_2Cl_2-EtOH), to yield (*S*,*R*$_{mp}$/*S*$_{mp}$)-**S17** as a white foam (35.5 mg, with 6% of free macrocycle **1**, 84%, in a 0.15 : 0.85 diastereoisomeric ratio by ^{1}H NMR, **Figure S52**); ^{1}H NMR (400 MHz, $CDCl_3$, 298 K)* δ 10.15 (s, 1H, H_d (*minor*)), 10.06 (s, 1H, H_d (*major*)), 7.82-7.71 (m, 2H, H_B and H_S), 7.69 (d, 2H, *J* = 1.8, H_c), 7.66-7.60 (m, 1H, H_T), 7.58 (d, 1H, *J* = 7.7, H_A), 7.49 (d, 2H, *J* = 7.8, H_C (*minor*)), 7.43 (d, 1H, *J* = 7.8, H_C (*major*)), 7.33-7.28 (m, 2H, H_R and H_m), 7.23-7.18 (br s, 1H, H_b), 7.14-7.08 (m, 5H, H_h (major), H_i and H_D), 6.95 (d, 2H, *J* = 1.8, H_l (*minor*)), 6.93 (d, 2H, *J* = 1.9, H_l (*major*)), 6.88-6.79 (m, 2H, H_g), 6.70-6.65 (m, 2H, H_h (*minor*)), 6.42 (d, 2H, *J* = 8.1, H_N), 6.39-6.33 (m, 1H, H_j), 6.28-6.16 (m, 4H, H_E and H_M (*major*)), 6.09 (d, 2H, *J* = 8.2, H_M (*minor*)), 4.65 (dd, 1H, *J* = 9.3, 4.7, H_e (*minor*)), 4.45 (dd, 1H, *J* = 10.2, 4.3, H_e (*major*)), 4.33-4.11 (m, 3H, H_k and one of H_L (m*ajor*)), 4.05-3.90 (m, 2H, one of H_L and one of H_F), 3.21 (dd, 1H, *J* = 13.6, 10.2, one of H_f), 3.01 (t, 1H, *J* = 7.0, one of H_L (m*inor*)), 2.93 (dd, 1H, *J* = 13.5, 4.3, one of H_f (*major*)), 2.90-2.84 (m, 1H, one of H_f (*minor*)), 2.73 (dd, 2H, *J* = 10.2, 7.7, H_Q), 2.46 (t, 2H, *J* = 6.4, H_O), 2.35-1.42 (m, 12H, H_K, H_P, H_J, H_I, H_H and H_G), 1.28 (s, 18H, H_a or H_n), 1.20 (s, 18H, H_a or H_n); ^{13}C NMR (101 MHz, $CDCl_3$, 298 K) δ 166.6, 163.7, 2 × 159.1, 157.9, 157.6, 157.2, 151.2, 150.1, 150.0, 147.1, 2 × 137.2, 136.9, 136.8, 131.7, 131.4, 131.3, 129.5, 129.2, 128.6, 2 × 128.4, 128.2, 126.8, 124.6, 122.8, 122.6, 122.4, 121.7, 120.5, 120.4, 120.2, 120.0, 119.9, 114.8, 114.7, 114.1, 114.0, 67.9, 65.8, 65.2, 64.9, 44.5, 38.3, 37.6, 35.3, 2 × 34.9, 32.4, 3 × 31.6, 29.8, 29.2, 29.0, 28.3, 25.6, 25.5. LR-ESI-MS (+ve) *m/z* = 1085.7 [M+H], calc. 1085.7.

*As stereochemistry could not be unambiguously assigned, the signals are simply designated (*major*) or (*minor*). Proton counts are provided for each signal and represent the expected integration of that environment. Where the major and minor diastereoisomer signals are coincident, no (*major*)/(*minor*) label is provided and the proton count indicated refers the expected integration of that signal in each of the stereoisomers that contributes to the multiplet.

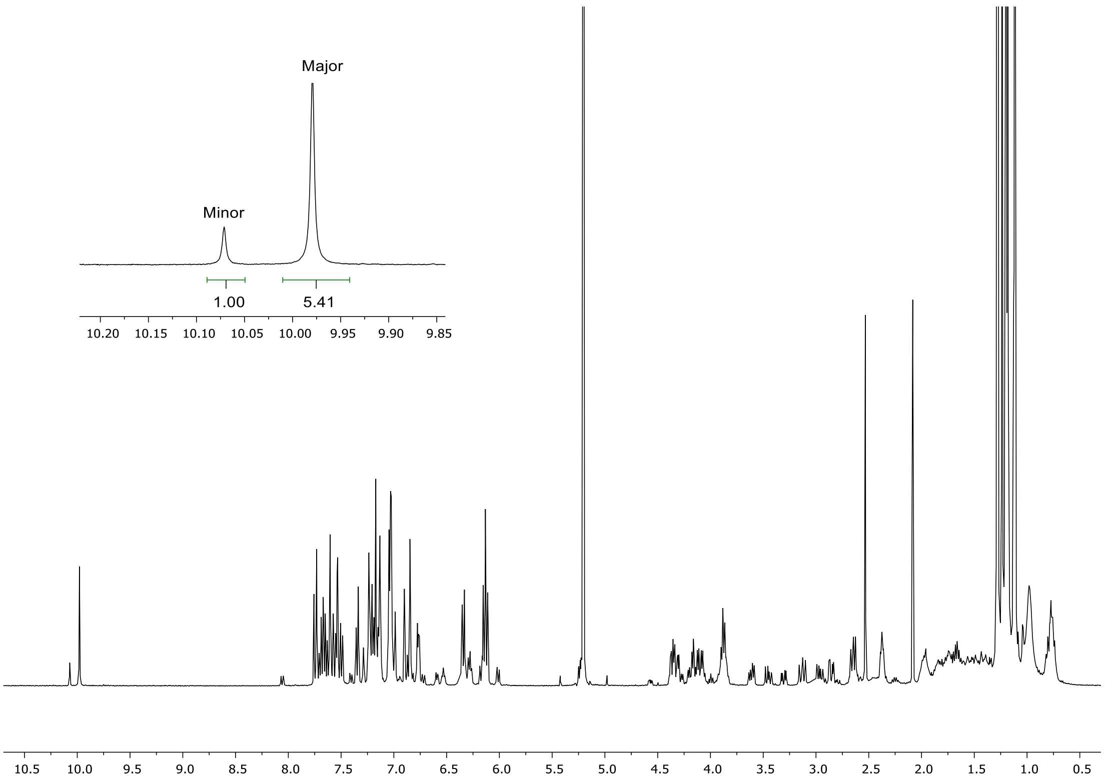

Figure S51. ^{1}H NMR (400 MHz, $CDCl_3$, 298 K) (S,R_{mp}/S_{mp})-**S17** prior to purification by preparative TLC.

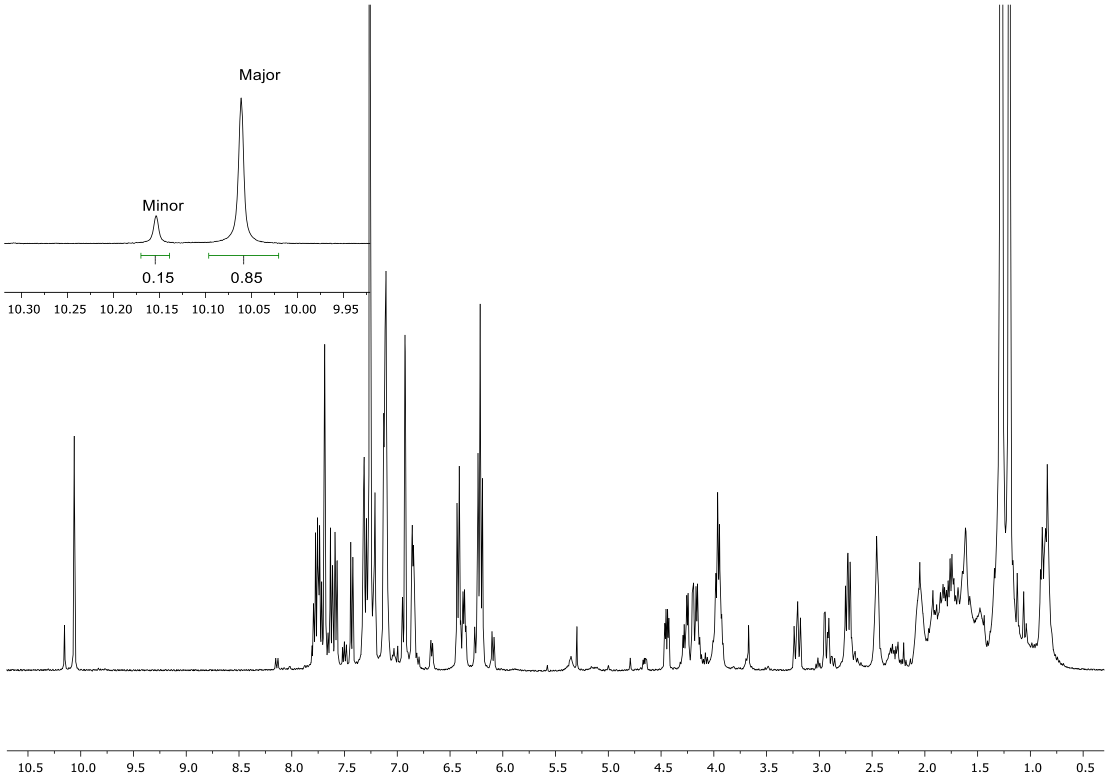

Figure S52. ^{1}H NMR (400 MHz, $CDCl_3$, 298 K) (S,R_{mp}/S_{mp})-**S17**. The signals corresponding to free macrocycle **1** are not integrated for reasons of clarity.

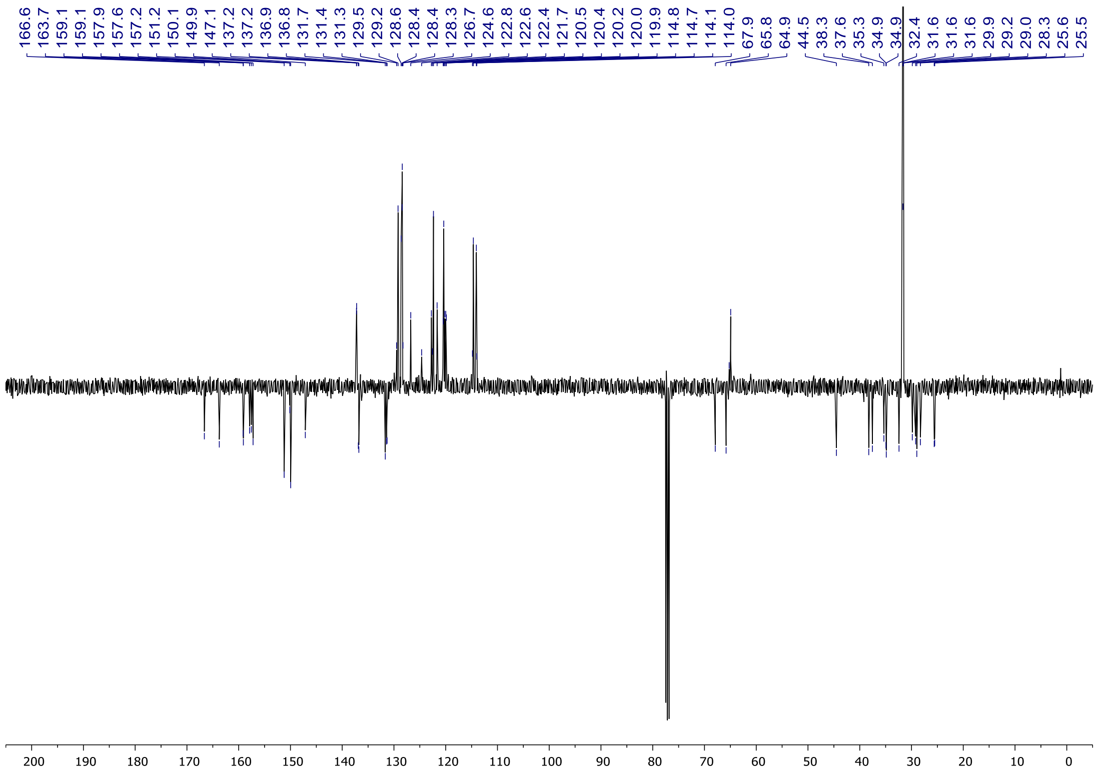

Figure S53. ^{13}C NMR (101 MHz, $CDCl_3$, 298 K) (S,R_{mp}/S_{mp})-**S17**.

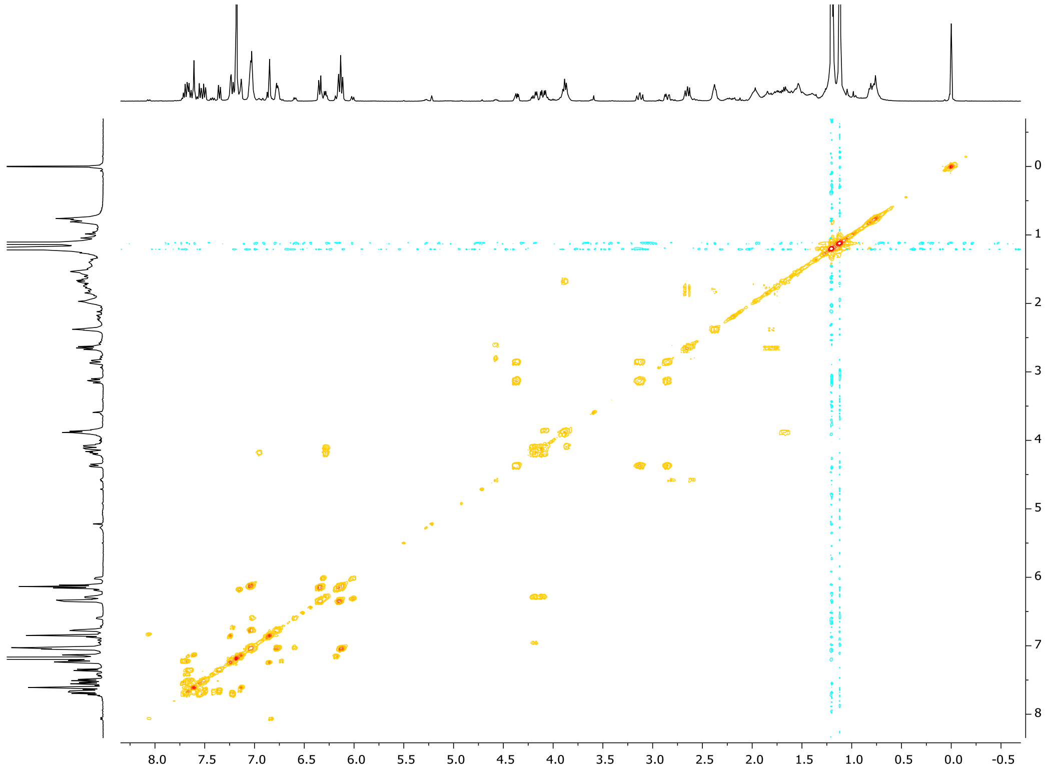

Figure S54. ^{1}H-^{1}H COSY NMR (400 MHz, $CDCl_3$, 298 K) (S,R_{mp}/S_{mp})-**S17**.

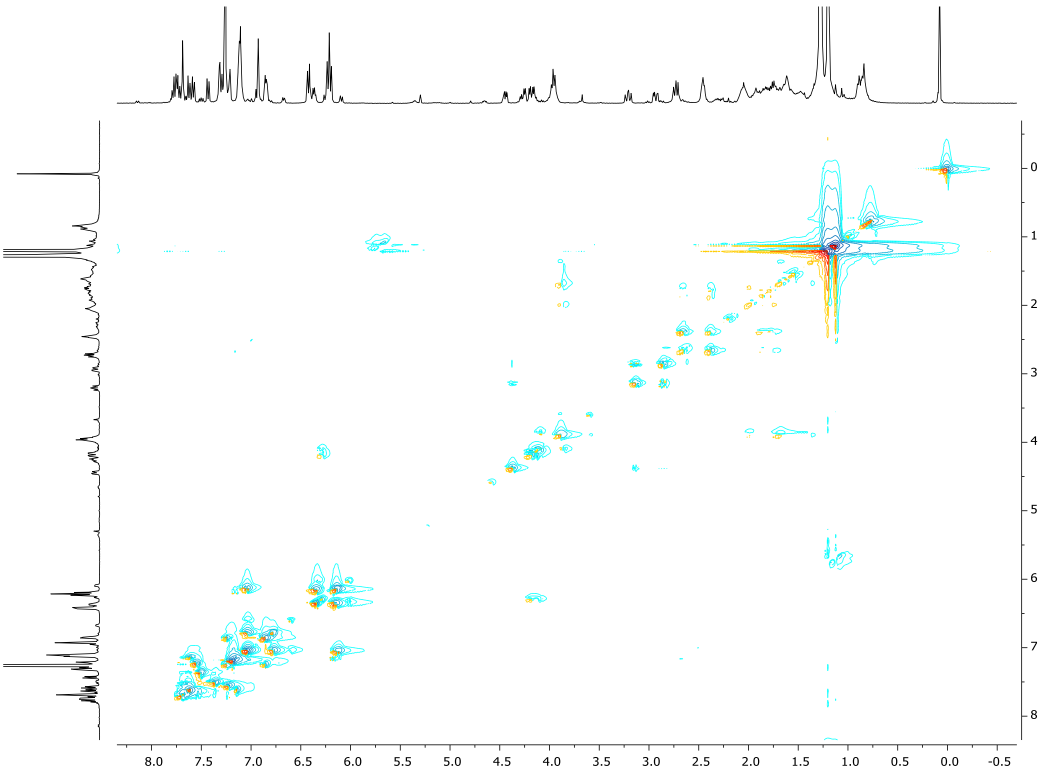

Figure S55. ^{1}H-^{1}H TOCSY NMR (400 MHz, $CDCl_3$, 298 K) (S,R_{mp}/S_{mp})-**S17.**

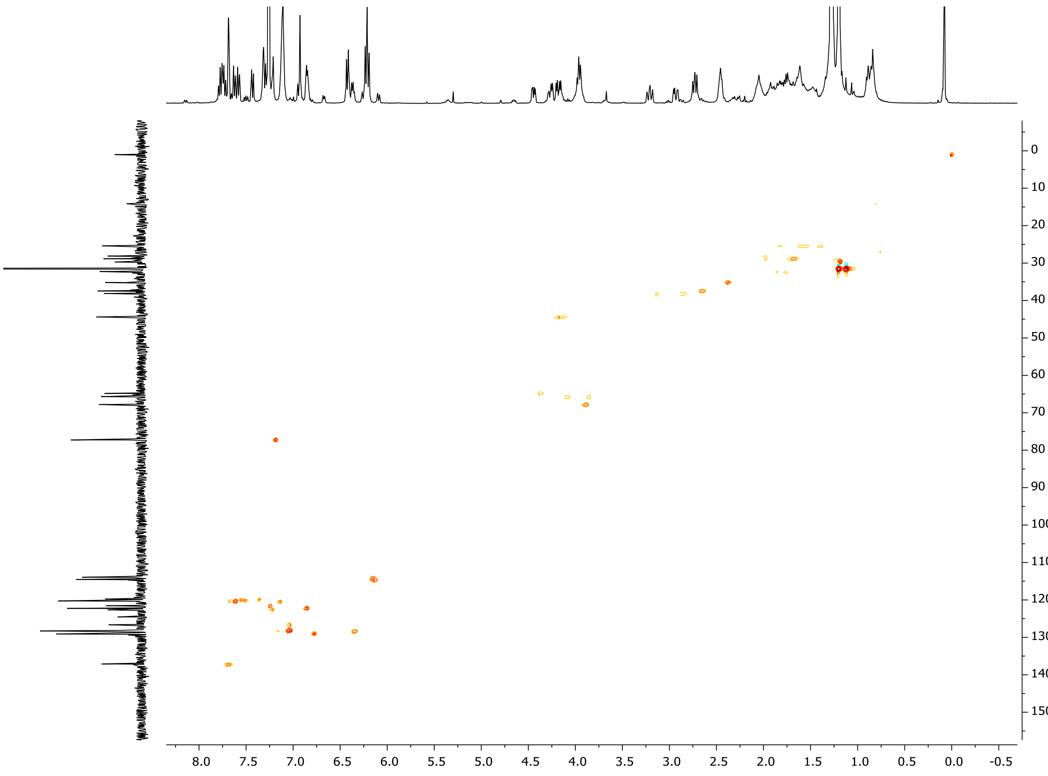

Figure S56. ^{1}H-^{13}C HSQC NMR (101 MHz, $CDCl_3$, 298 K) (S,R_{mp}/S_{mp})-**S17**.

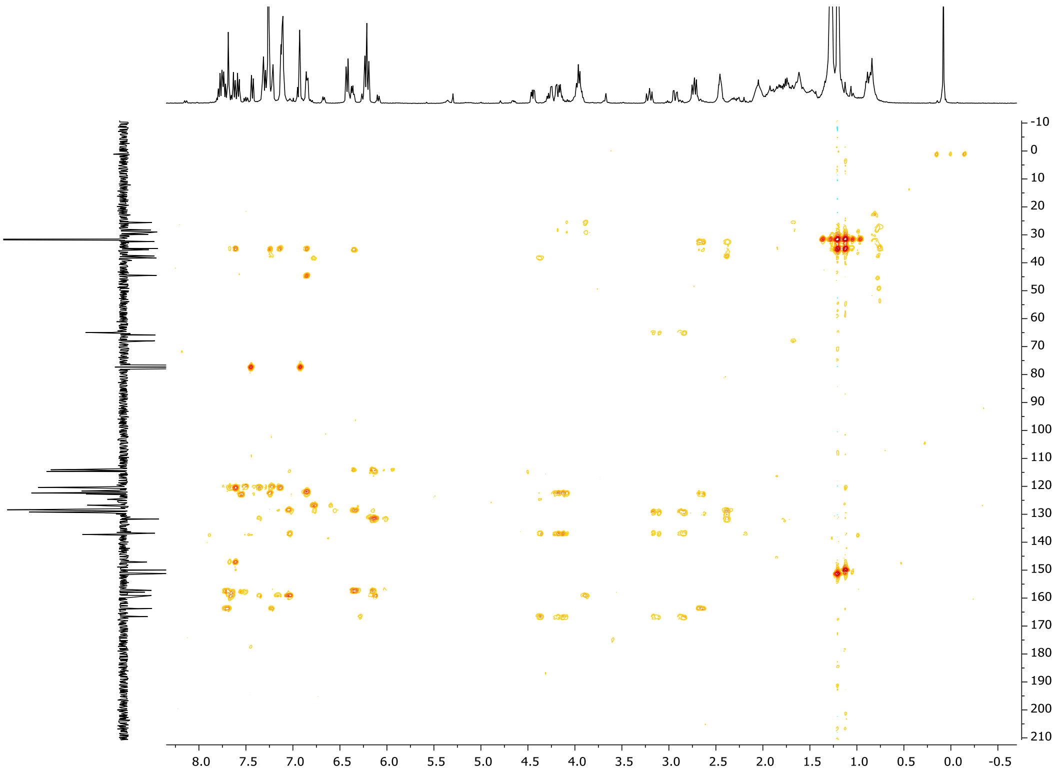

Figure S57. ^{1}H-^{13}C HMBC NMR (101 MHz, $CDCl_3$, 298 K) (S,R_{mp}/S_{mp})-**S17**.

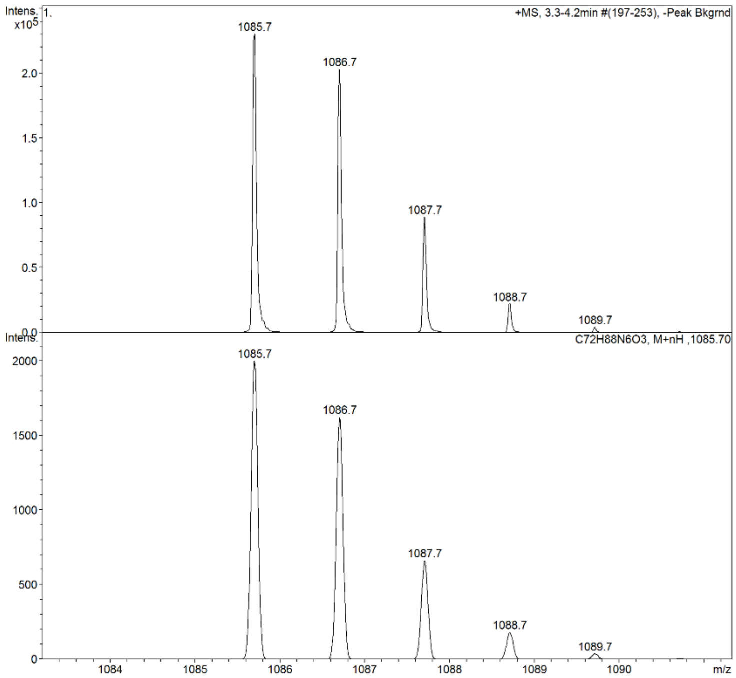

Figure S58. ESI-MS isotopic pattern of (D, R_{mp}/S_{mp})-**S17**; observed (top) and calculated (bottom).

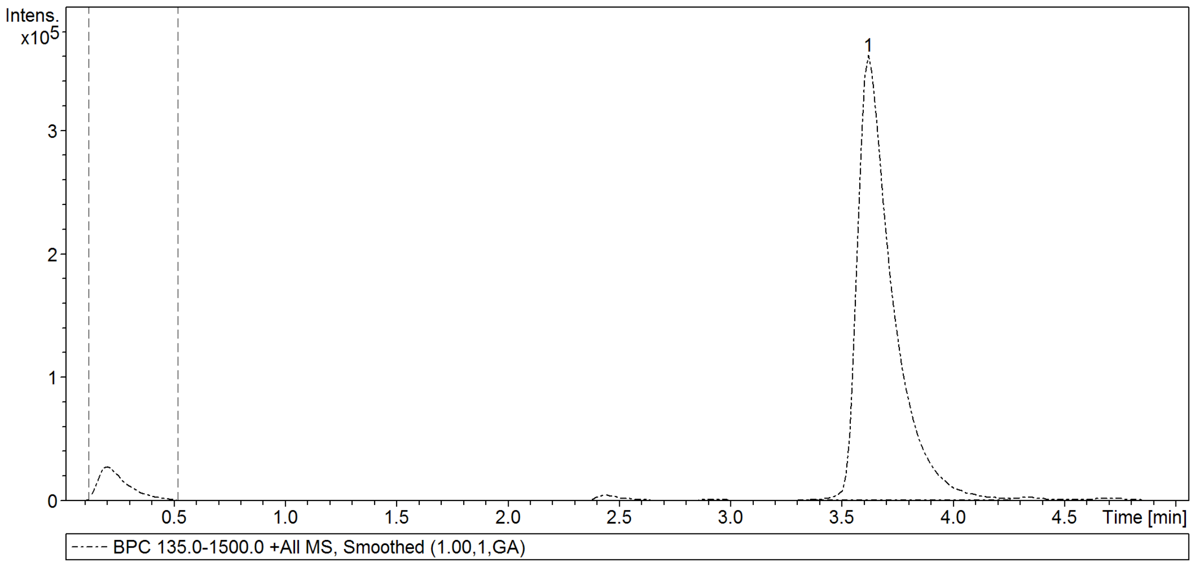

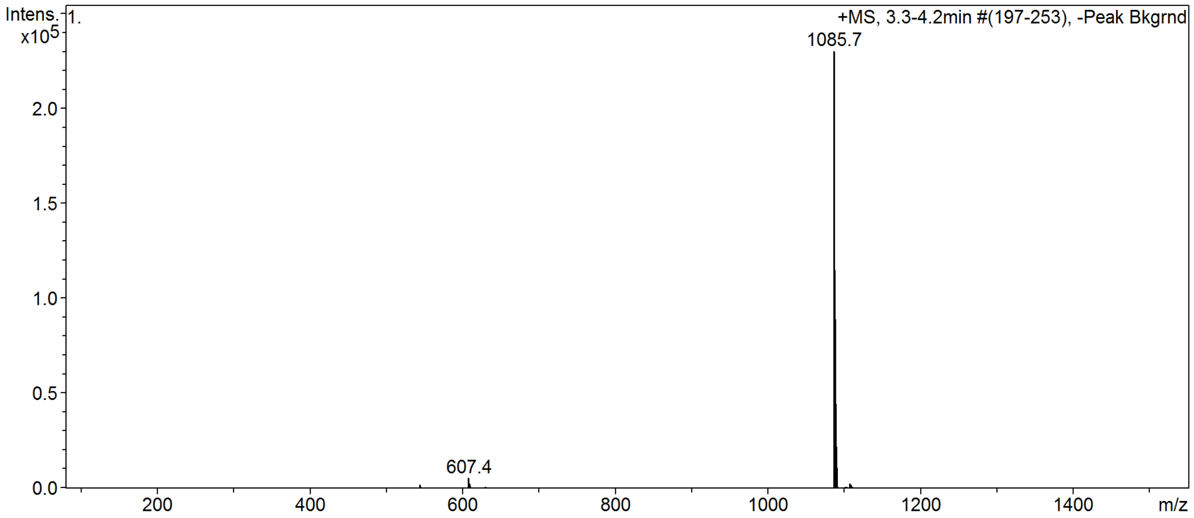

Figure S59. LCMS trace (C_{18} column, gradient 5 minutes (1 : 4 MeCN+0.2% formic acid-H_2O +0.2% formic acid →1 : 0 MeCN-H_2O +0.2% formic acid), UV 254 nm), of (S,R_{mp}/S_{mp})-**S17** following purification by chromatography (top), and HR-ESI-MS spectrum of (S,R_{mp}/S_{mp})-**S17** (bottom).

Entry 5 – axle (*S*)-S18 and rotaxanes (*S*,R_{mp}/S_{mp})-S19 derived from alkyne 2b and azide (*S*)-3b

Axle (*S*)-**S18**

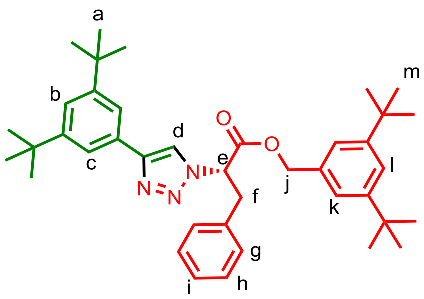

A 5 mL round bottomed flask was charged with **2a** (7.6 mg, 0.035 mmol), **3d** (14 mg, 0.035 mmol), sodium *L*-ascorbate (11.1 mg, 0.056 mmol) and $CuSO_4.5H_2O$ (8.6 mg, 0.035 mmol) and DMF (2 mL). The reaction mixture was stirred at rt for 16 h. EtOAc (20 mL) was added and the organic extract was washed with saturated EDTA-NH_3 solution (15 mL) and 5% w/v LiCl solution (3 × 15 mL). The organic layer was dried over $MgSO_4$, filtered, and the solvent was removed *in vacuo*. The residue was purified by chromatography 0→5% CH_2Cl_2-EtOAc), to yield axle (*S*)-**S18** as a yellow foam (13.2 mg, 62%);^{1}H NMR (400 MHz, $CDCl_3$, 298 K) δ 7.82 (s, 1H, H_d), 7.61 (d, 2H, *J* = 1.8, H_c), 7.42 (t, 1H, *J* = 1.8, H_l), 7.32 (t, 1H, *J* = 1.8, H_m), 7.24-7.19 (m, 3H, H_h and H_i), 7.14-7.10 (m, 2H, H_g), 6.98 (d, 2H, *J* = 1.8, H_k), 6.58 (t, 1H, *J* = 5.5, H_j), 5.34-5.28 (dd, 1H, *J* = 9.1, 6.3, H_e), 4.45 (dd, 1H, *J* = 14.6, 5.6, one of H_j), 4.37 (dd, 1H, *J* = 14.6, 5.6, one of H_j), 3.73-3.65 (dd, 1H, *J* = 13.8, 6.3, one of H_f), 3.53 (dd, 1H, *J* = 13.8, 9.1, one of H_f), 1.36 (s, 18H, H_a or H_m), 1.27 (s, 18H, H_a or H_m); ^{13}C NMR (101 MHz, $CDCl_3$, 298 K) δ 167.4, 151.5, 151.5, 149.0, 136.2, 135.8, 129.4, 129.1, 129.0, 127.5, 122.8, 122.0, 122.0, 120.3, 120.2, 66.9, 44.6, 39.5, 35.1, 34.9, 31.6, 31.5. HR-ESI-MS (+ve) *m/z* = 607.4369 [M+H] calc. 607.4370.

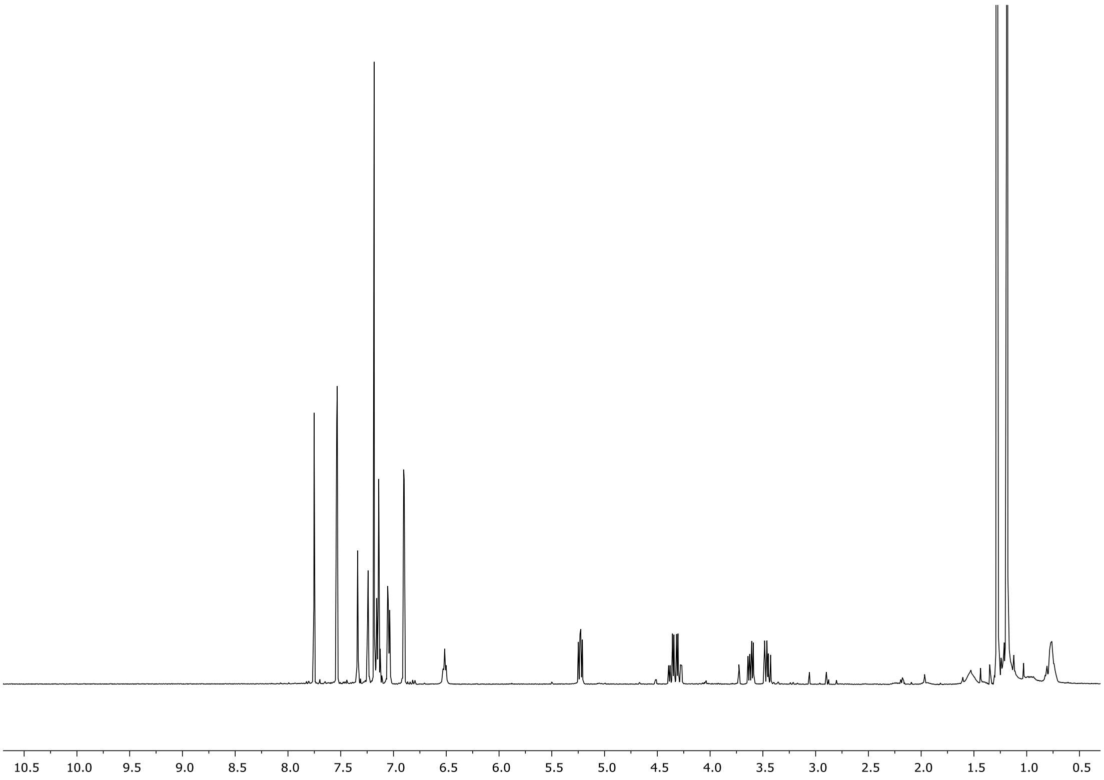

Figure S60. ^{1}H NMR (400 MHz, $CDCl_3$, 298 K) (*S*)-**S18**.

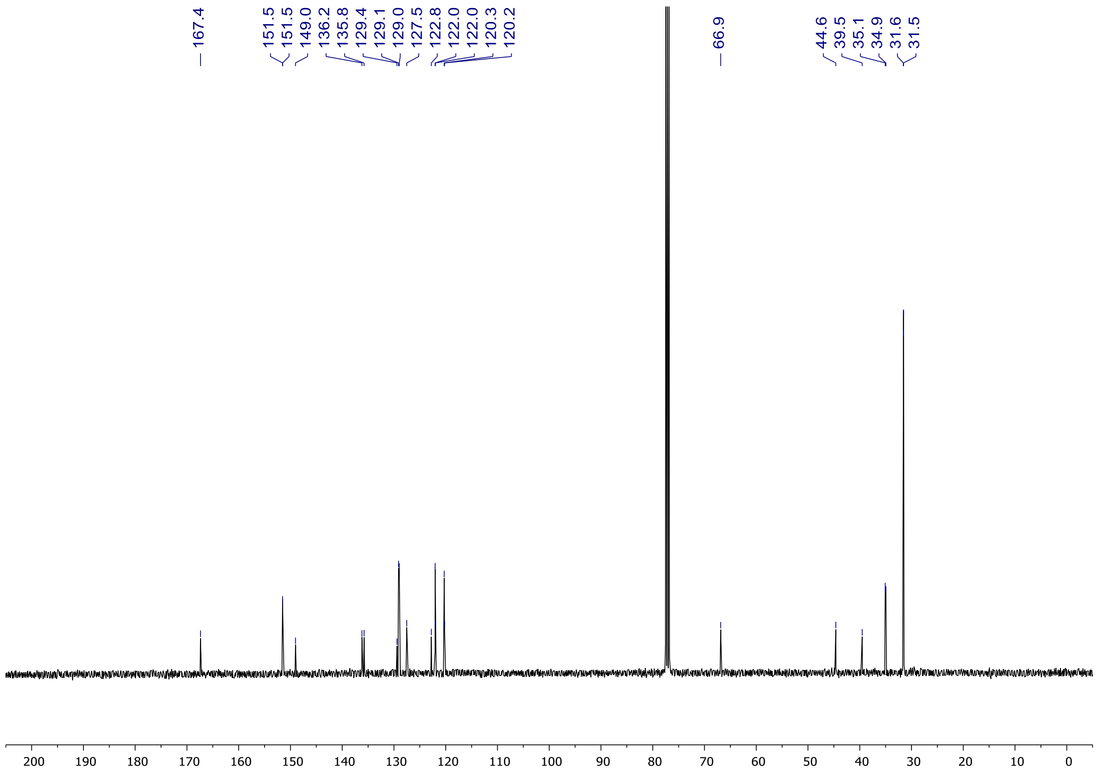

Figure S61. ^{13}C NMR (400 MHz, $CDCl_3$, 298 K) (*S*)-**S18**.

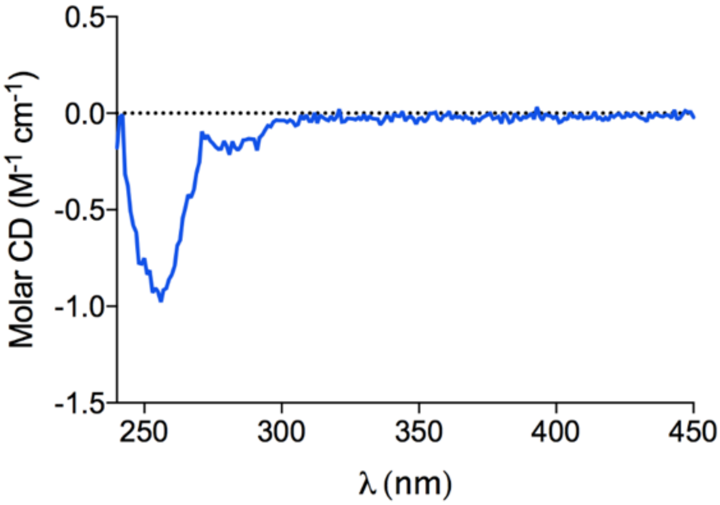

Figure S62. Circular dichroism spectrum of (*S*)-**S18** (66.0 μM in $CHCl_3$).

Rotaxanes (S,R_{mp}/S_{mp})-**S19**

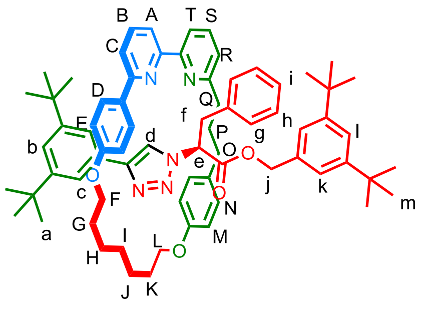

(S,R_{mp})-**S19**

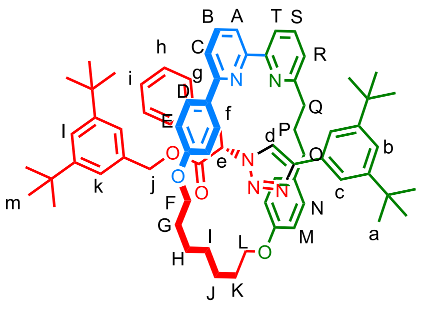

(S,S_{mp})-**S19**

A dry sealed vessel was charged with **2a** (21.9 mg, 0.102 mmol), **3d** (40.3 mg, 0.102 mmol), macrocycle **1** (24.5 mg, 0.051 mmol), $[Cu(MeCN)_4]PF_6$ (18.2 mg, 0.049 mmol), DIPEA (35.5 µL, 0.204 mmol), in anhydrous CH_2Cl_2 (1.2 mL). The reaction mixture was stirred at rt for 16 h, protected by an argon atmosphere. TFA (0.10 mL, 1.31 mmol) was added and the reaction mixture was stirred for an additional 16 h. Saturated EDTA-NH_3 solution (10 mL) was added, and the aqueous layer was extracted with $CHCl_3$ (3 × 30 mL), dried over $MgSO_4$, filtered, and had the solvent removed *in vacuo*. The residue containing (S,R_{mp}/S_{mp})-**S19** (in a 0.96 : 0.04 diastereoisomeric ratio by ^{1}H NMR, **Figure S63**) was purified by chromatography (CH_2Cl_2 with 0→5% EtOH), to yield (S,R_{mp}/S_{mp})-**S19** as a white foam (35.2 mg, 64% in a 0.95 : 0.05 diastereoisomeric ratio by ^{1}H NMR, **Figure S64**); ^{1}H NMR (400 MHz, $CDCl_3$, 298 K) δ 10.01 (s, 1H, H_d (*major*)), 9.92 (s, 1H, H_d (*minor*)),* 7.79-7.70 (m, 4H, H_S, H_B and H_c), 7.63 (d, 1H, J = 7.1, H_T), 7.59-7.55 (m, 1H, H_A), 7.46 (dd, 1H, J = 7.8, 0.7, H_C), 7.34 (t, 1H, J = 1.8, H_l), 7.29 (d, 1H, J = 7.0, H_R), 7.24-7.21 (m, 2H, H_D), 7.20 (t, 1H, J = 1.8, H_b), 7.09–7.02 (m, 3H, H_h and H_i), 6.93 (d, 2H, J = 1.8, H_k), 6.68 (dd, 2H, J = 7.7, 1.6, H_g), 6.44 (d, 2H, J = 8.5, H_N), 6.28-6.21 (m, 4H, H_E and H_M), 4.90 (d, 1H, J = 12.0, one of H_j), 4.83 (d, 1H, J = 12.0, one of H_j), 4.71 (dd, 1H, J = 11.9, 4.2, H_e), 4.24 (td, 1H, J = 8.5, 5.0, one of H_F), 4.00 (t, 2H, J = 7.0, H_L), 3.90 (dt, 1H, J = 8.6, 5.3, one of H_F), 2.94–2.86 (dd, 1H, J = 13.3, 11.9, one of H_f), 2.82–2.71 (m, 3H, one of H_f and H_Q), 2.53-2.38 (m, 2H, H_O), 2.18–2.03 (m, 2H, H_G), 2.03–1.89 (m, 2H, H_P), 1.87–1.43 (m, 8H, H_H, H_I, H_J and H_K), 1.26 (s, 18H, H_m), 1.19 (s, 18H, H_a); ^{13}C NMR (101 MHz, CD, 298 K) δ 168.1, 164.0, 159.3, 159.2, 158.0, 157.6, 157.2, 151.0, 149.8, 147.3, 137.1, 135.8, 134.1, 131.6, 131.2, 129.0, 128.6, 128.4, 126.9, 123.4, 123.0, 122.8, 122.5, 120.6, 120.3, 120.0, 119.9, 119.5, 114.9, 114.0, 68.2, 68.0, 65.7, 63.5, 37.7, 37.6, 35.5, 34.9, 34.9, 32.4, 31.6, 31.6, 29.2, 29.0, 28.3, 25.8, 25.6. LR-ESI-MS (+ve) m/z = 1086.7 $[M+H]^+$ calc. 1086.7.

*Given the high d.r. of rotaxane **S19**, only H_d of the minor diastereomer, the signal used to assess diastereoselectivity, was assigned.

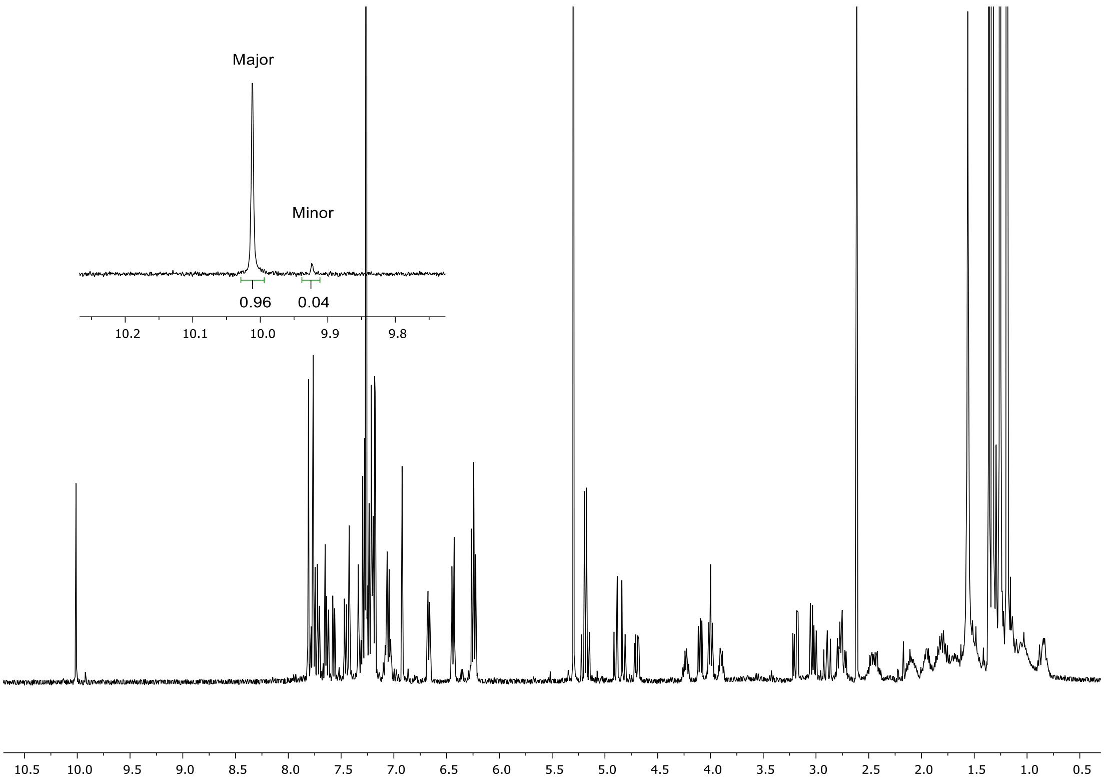

Figure S63. ^{1}H NMR (400 MHz, $CDCl_3$, 298 K) (S,R_{mp}/S_{mp})-**S19** prior to purification by chromatography.

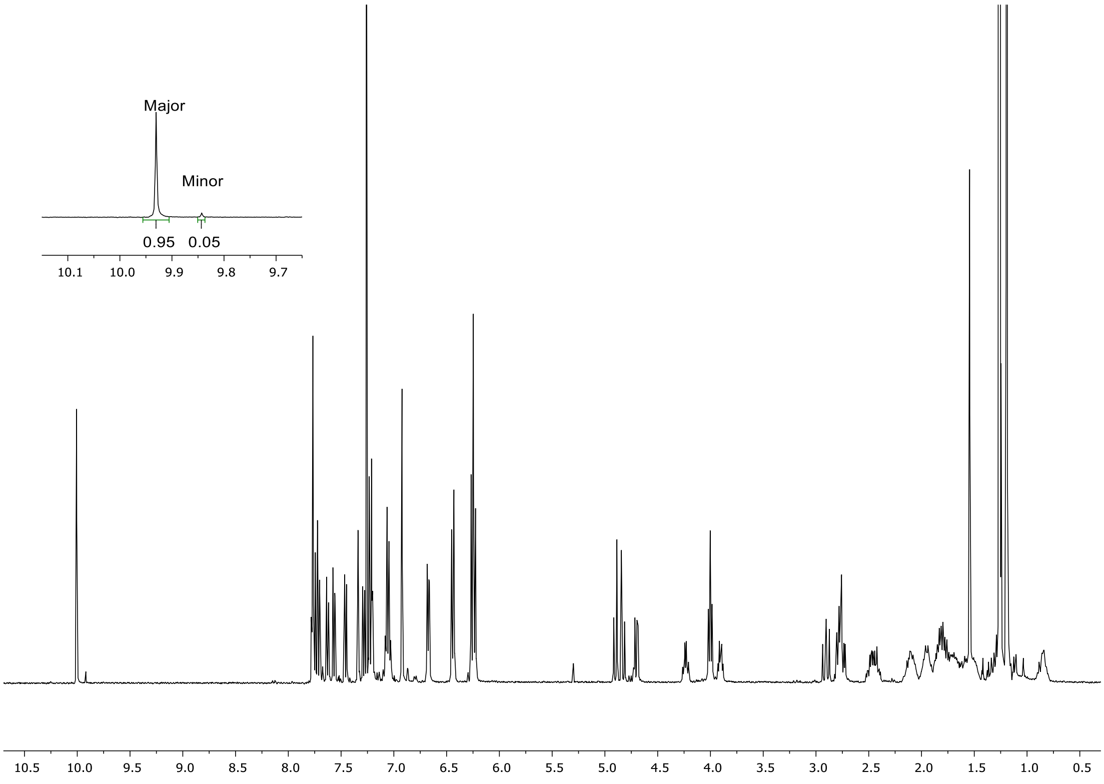

Figure S64. ^{1}H NMR (400 MHz, $CDCl_3$, 298 K) (S,R_{mp}/S_{mp})-**S19**, following purification by chromatography.

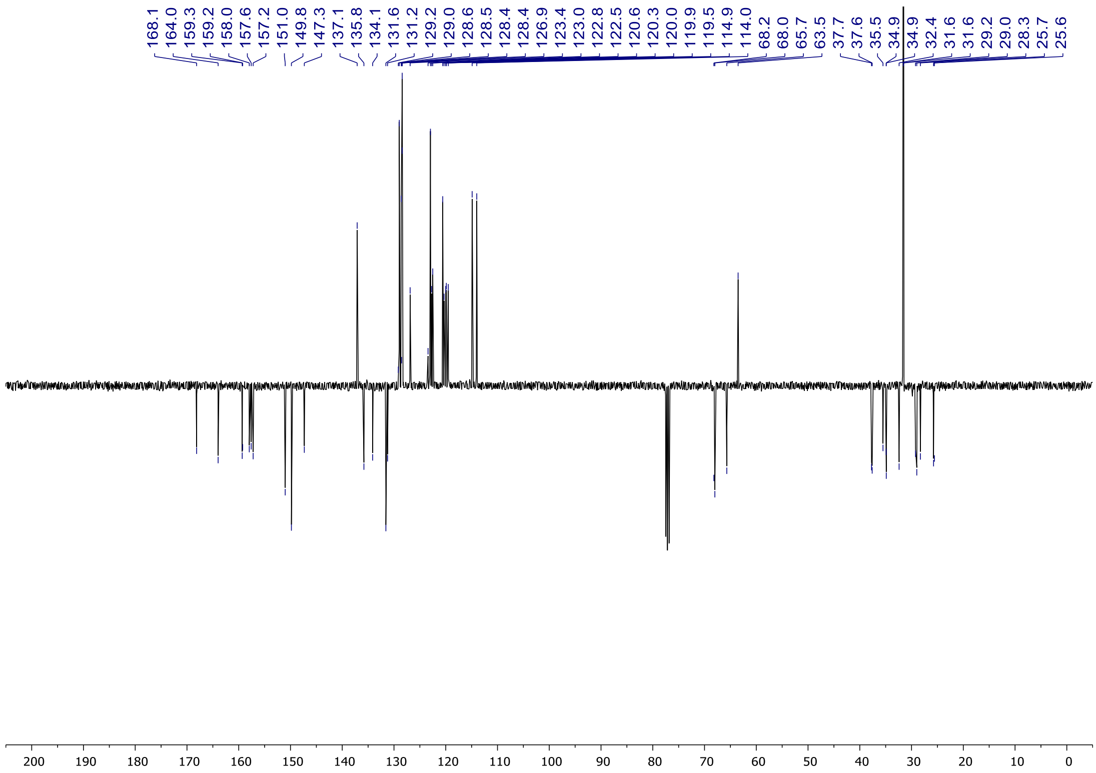

Figure S65. ^{13}C NMR (101 MHz, $CDCl_3$, 298 K) (*S*,R_{mp}/S_{mp})-**S19**, following purification by chromatography.

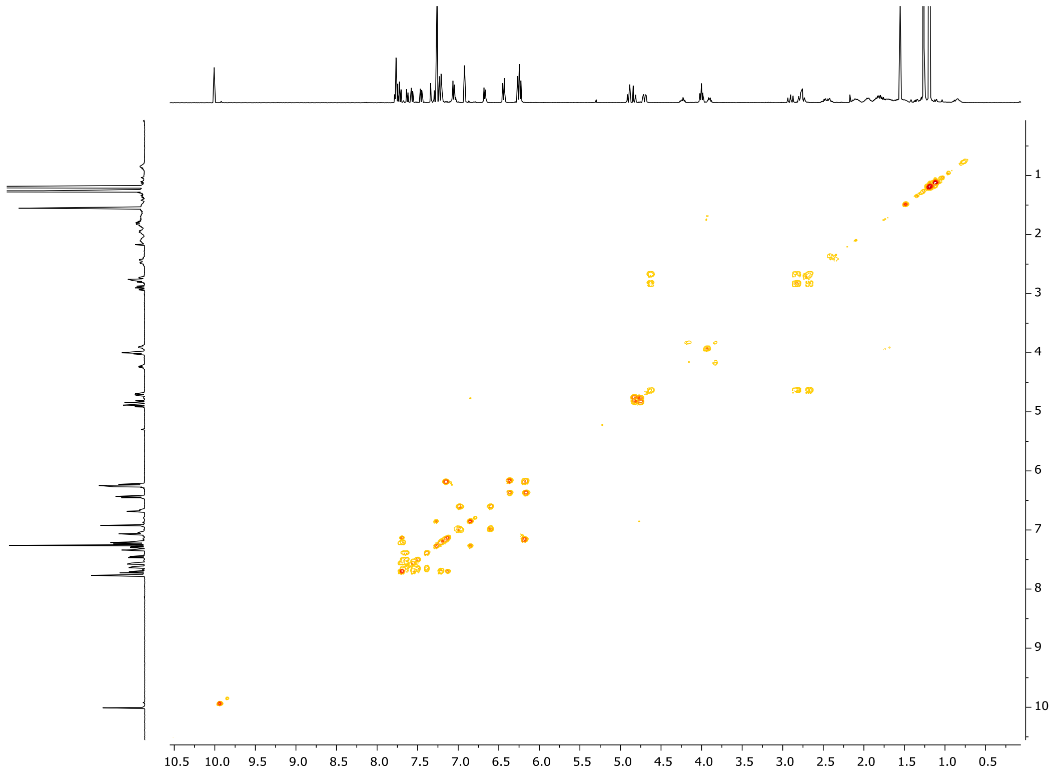

Figure S66. ^{1}H-^{1}H COSY NMR (400 MHz, $CDCl_3$, 298 K) (*S*,R_{mp}/S_{mp})-**S19**, following purification by chromatography.

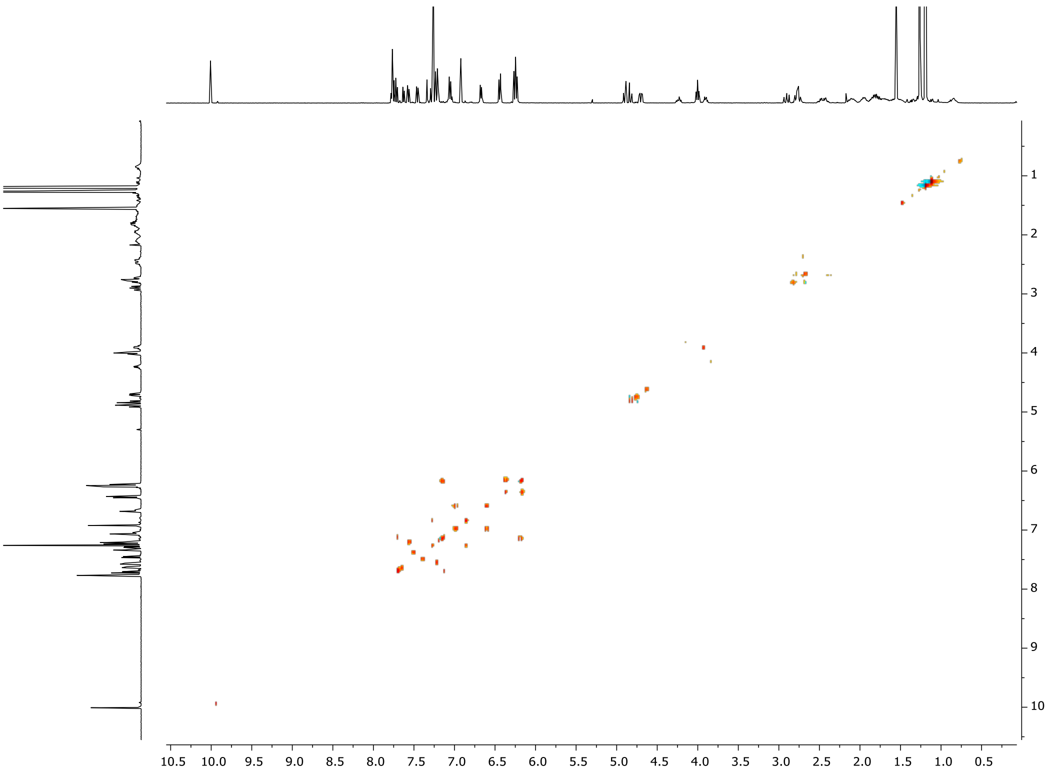

Figure S67. ^{1}H-^{1}H TOCSY NMR (400 MHz, $CDCl_3$, 298 K) (S,R_{mp}/S_{mp})-**S19**, following purification by chromatography.

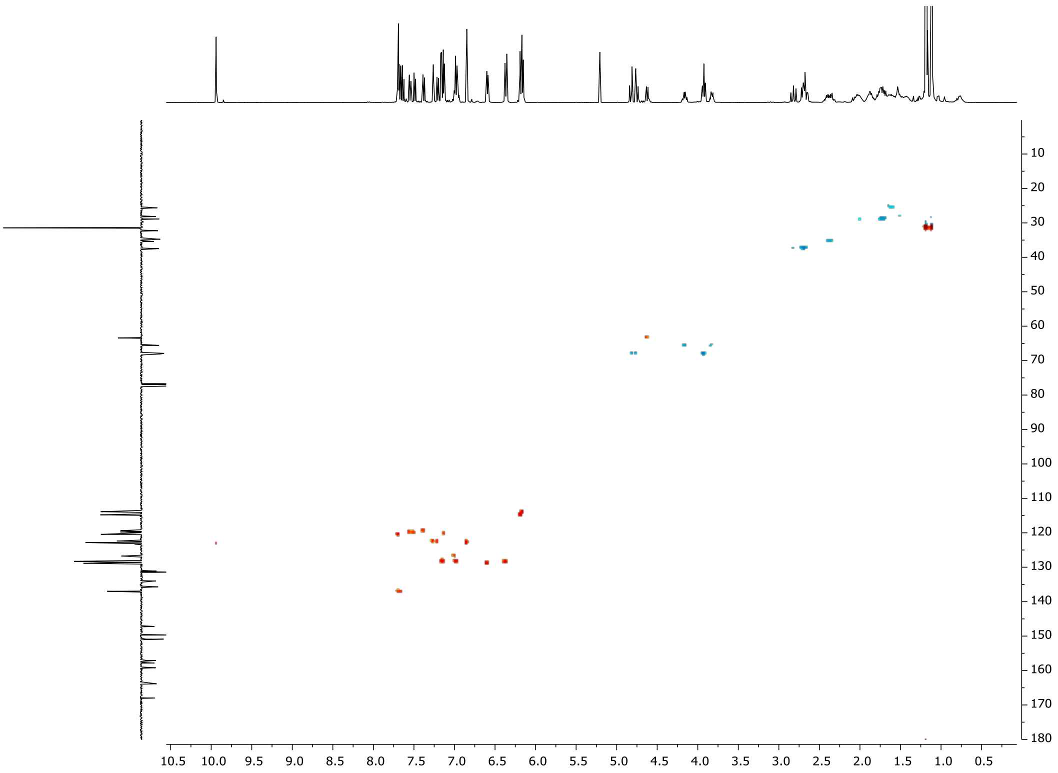

Figure S68. ^{1}H-^{13}C HSQC NMR (101 MHz, $CDCl_3$, 298 K) (S,R_{mp}/S_{mp})-**S19**, following purification by chromatography.

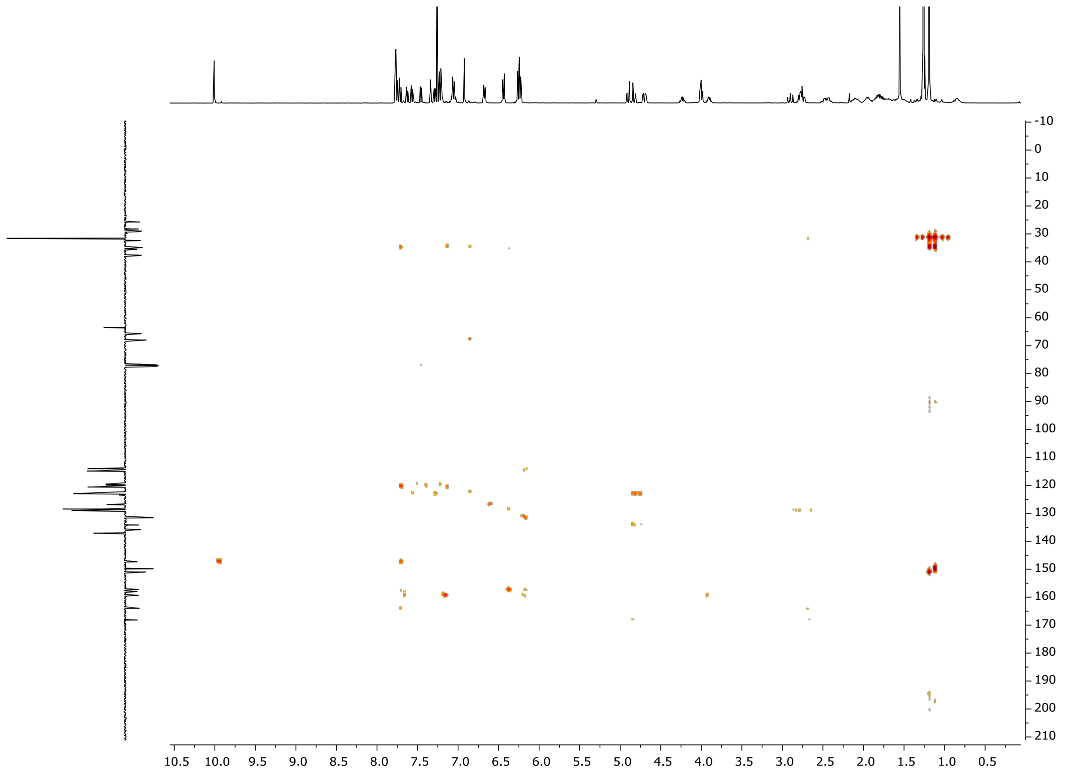

Figure S69. ^{1}H-^{13}C HMBC NMR (101 MHz, $CDCl_3$, 298 K) (*S*,*R*$_{mp}$/*S*$_{mp}$)-**S19**, following purification by chromatography.

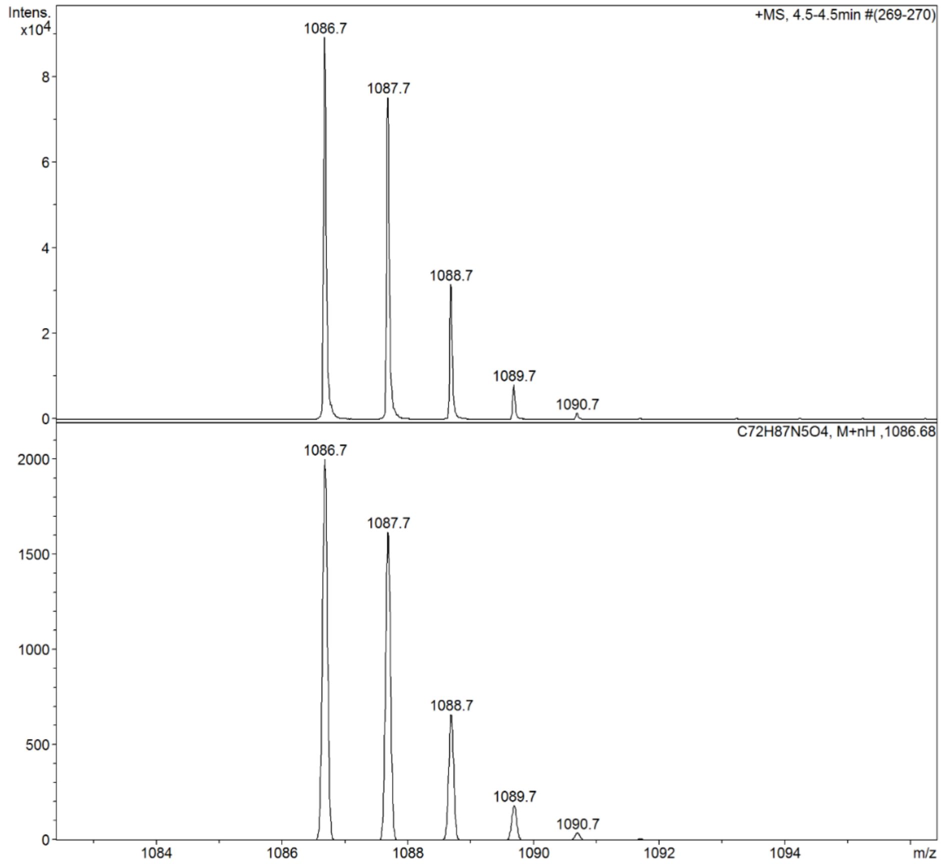

Figure S70. ESI-MS isotopic pattern of (*S*,*R*$_{mp}$/*S*$_{mp}$)-**S19**; observed (top) and calculated (bottom).

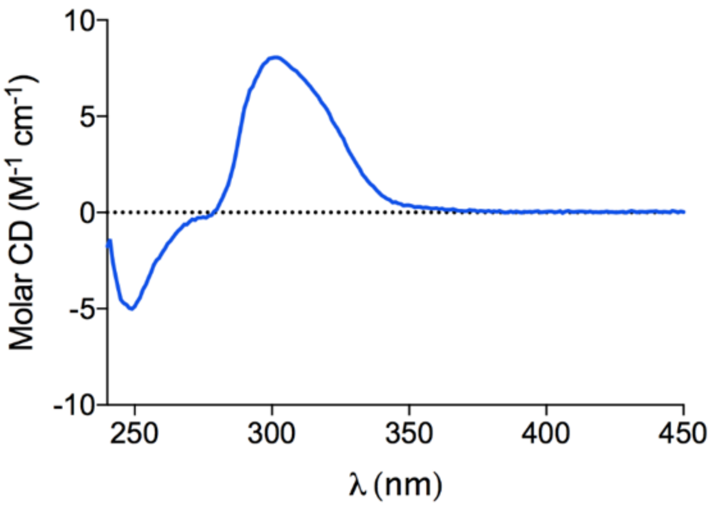

Figure S71. Circular dichroism spectrum of (S,R_{mp}/S_{mp})-**S19** (44.2 μM in $CHCl_3$).

Entry 6 – axle S20 and rotaxane 4 derived from alkyne 2a and azide 3e

Axle (*S*)-**S20**

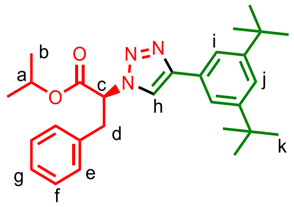

A 5 mL round bottom flask was charged with **2a** (44.1 mg, 0.206 mmol), (*S*)-**3e** (48.0 mg, 0.206 mmol), $CuSO_4.5H_2O$ (51.3 mg, 0.206 mmol), sodium *L*-ascorbate (43.6 mg, 0.220 mmol), in DMF (3 mL). The reaction mixture was stirred at rt for 16 h. Saturated EDTA-NH_3 solution (50 mL) was added, and the aqueous layer was extracted with EtOAc (3 × 30 mL). The combined organic extracts were washed with 5% w/v LiCl (5 × 30 mL), brine (30 mL), were dried over $MgSO_4$, filtered, and had the solvent removed *in vacuo*, and the residue was purified by chromatography ($CH_2Cl_2$with 0→10% EtOH), to yield axle (*S*)-**S20** as a yellow solid (77.0 mg, 84%, 95.6% ee); ^1H NMR (400 MHz, $CDCl_3$, 298 K) δ 7.79 (s, 1H, H_h), 7.58 (d, 2H, *J* = 1.6, H_i), 7.34 (t, 1H, *J* = 1.6, H_j), 7.20-7.14 (m, 3H, H_f and H_g), 7.07-7.01 (m, 2H, H_e), 5.53 (dd, 1H, *J* =7.8, 7.4, H_c), 4.98 (sept, 1H, *J* = 6.2, H_b), 3.44 (app. d, 1H, *J* = 7.8, one of H_d), 1.30 (s, 18H, H_k), 1.15 (d, 3H, *J* = 6.2, three of H_a), 1.09 (d, 3H, *J* = 6.2, three of H_a); ^{13}C NMR (101 MHz, $CDCl_3$, 298 K) δ 168.1, 151.5, 148.7, 135.0, 129.9, 129.3, 128.9, 127.7, 122.6, 119.5, 70.6, 64.3, 39.4, 35.1, 31.6, 31.4, 21.7, 21.6; LR-ESI-MS (+ve) *m/z* (%) = 366.7 $[M+H]^+$ (100); HR-ESI-MS (+ve) *m/z* = 448.2969 $[M+H]^+$ calc. 448.2959; Chiral SCFC (Chiralpak IG, 250 × 4.6 mm, 5 µm, 40 °C, EtOH (0.2% v/v NH_3)/CO_2 = 15%, 4 mL/min, λ = 210-400 nm): tR [(*R*/*S*)-**S20**] = 1.69 min, 2.02 min; tR [(*S*)-**S20**] = 1.70.

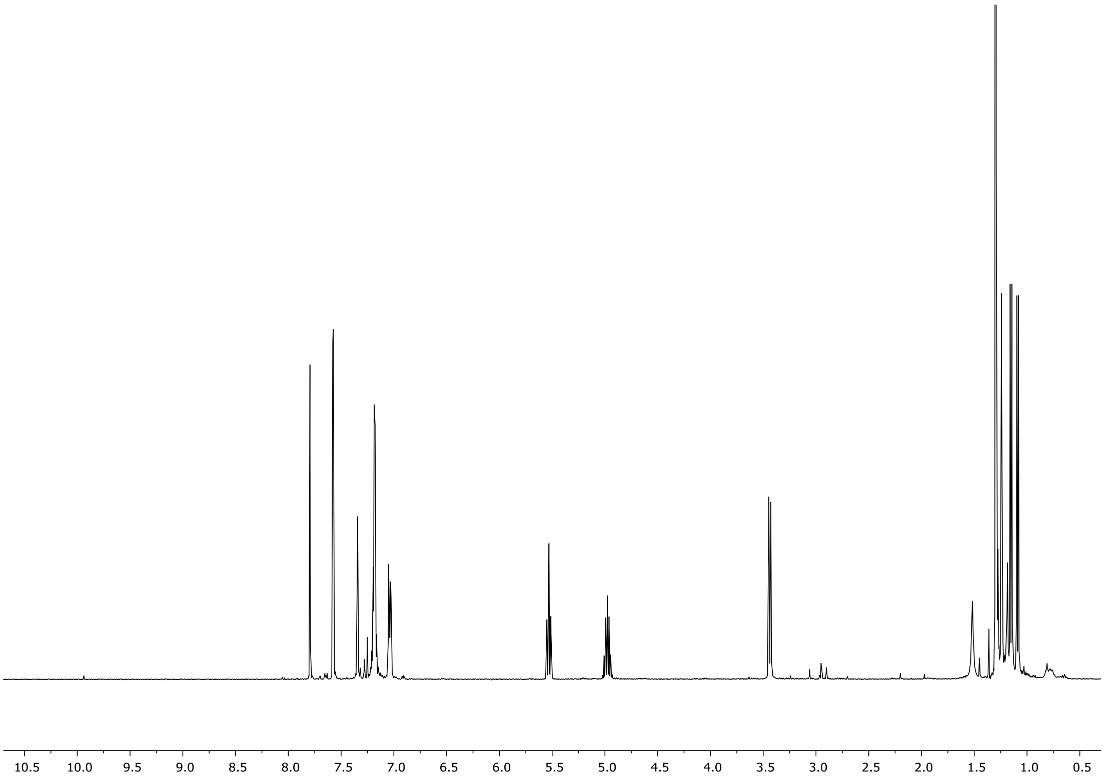

Figure S72. ^1H NMR (400 MHz, $CDCl_3$ 298 K) (*S*)-**S20**.

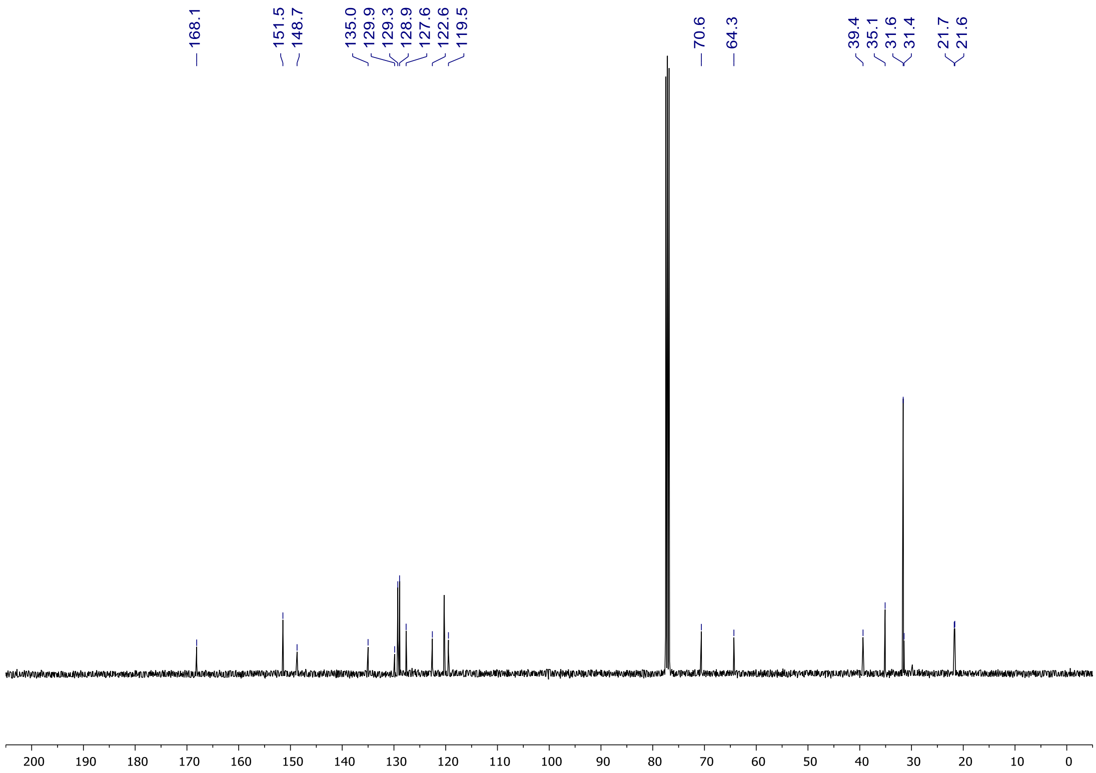

Figure S73. ^{13}C NMR (101 MHz, $CDCl_3$ 298 K) (*S*)-**S20**.

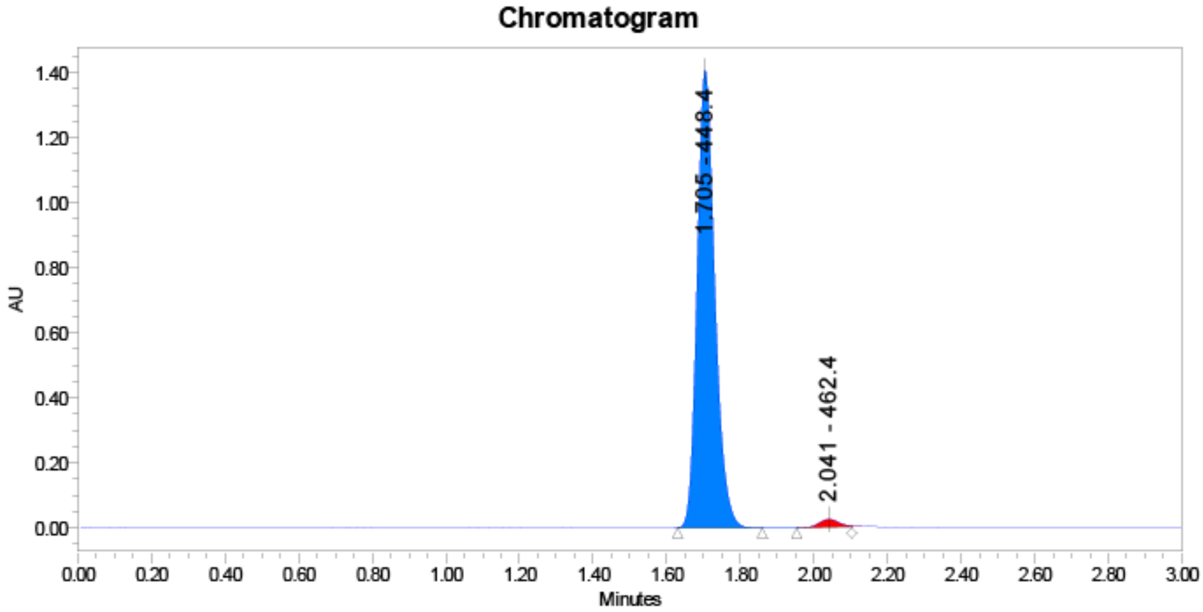

Peak Results

	Retention Time (min)	Area (µV*sec)	% Area	Width @ 50%
1	1.70	4675680	97.8	0.05062
2	2.04	106648	2.2	0.06229

Figure S74. Chiral SCFC chromatogram of (*S*)-**S20**.

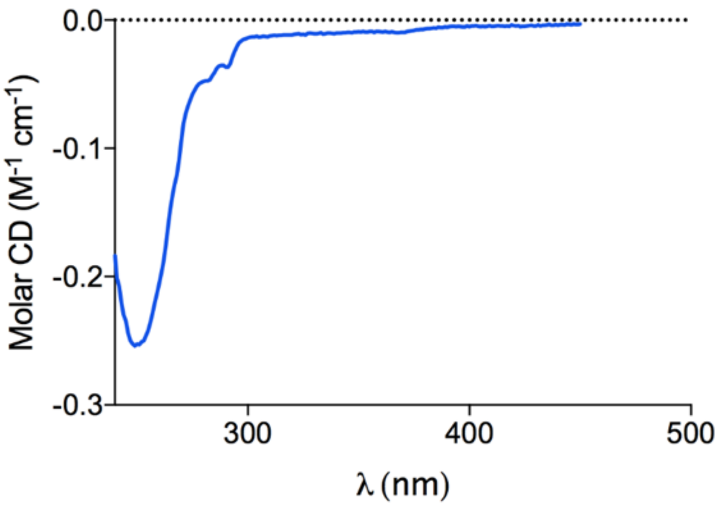

Figure S75. Circular dichroism spectrum of (*S*)-**S20** (62.5 μM in $CHCl_3$).

Axle (R)-**S20**

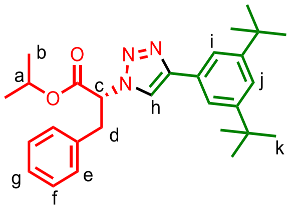

A 5 mL round bottom flask was charged with **2a** (13.5 mg, 0.0630 mmol), (R)-**3e** (14.6 mg, 0.0630 mmol), $CuSO_4.5H_2O$ (15.6 mg, 0.0630 mmol), sodium ascorbate (20.0 mg, 0.100 mmol), in DMF (3 mL). The reaction mixture was stirred at rt for 16 h. Saturated EDTA-NH_3 solution (50 mL) was added, and the aqueous layer was extracted with EtOAc (3 × 30 mL). The combined organic extracts were washed with 5% w/v LiCl (5 × 30 mL), brine (30 mL), were dried over $MgSO_4$, filtered, and had the solvent removed *in vacuo*. The residue was purified by chromatography (CH_2Cl_2 with 0→10% EtOH), to yield axle (R)-**S20** as a yellow solid, with identical spectra to (S)-**S20**, with the exception of circular dichroism spectra (**Figure S76**) (8.7 mg, 31%);

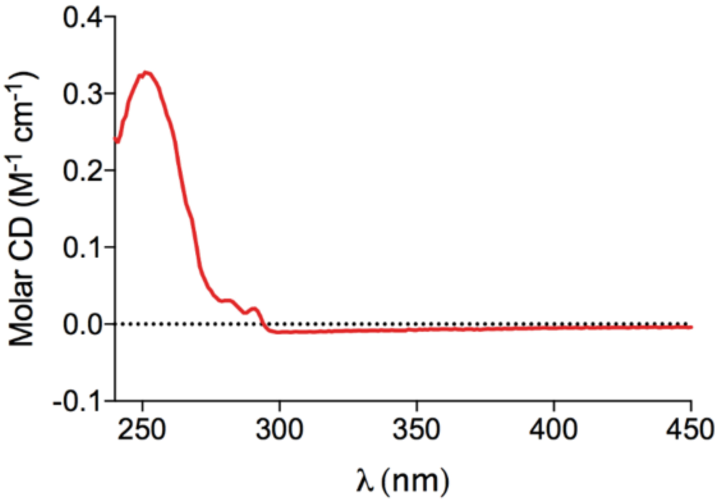

Figure S76. Circular dichroism spectrum of (R)-**S20** (53.6 μM in $CHCl_3$).

Axle (*R*/*S*)-**S20**

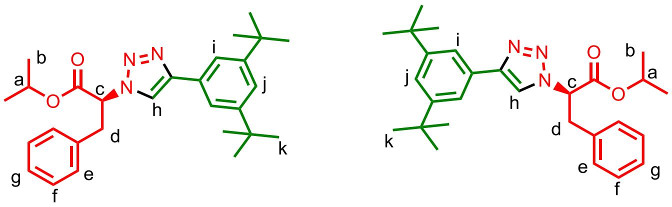

A 5 mL round bottom flask was charged with **2a** (16.4 mg, 0.0.765 mmol), **3g** copper sulfate pentahydrate (11.3 mg, 0.0454 mmol), sodium *L*-ascorbate (12.1 mg, 0.0611 mmol), and DMF (3 mL). The reaction mixture was stirred at rt for 16 h. Saturated EDTA-NH_3 solution (50 mL) was added, and the aqueous layer was extracted with EtOAc (3 × 30 mL). The combined organic extracts were washed with 5% w/v LiCl (5 × 30 mL), brine (30 mL), were dried over $MgSO_4$, filtered, and had the solvent removed *in vacuo*, to yield the product as a yellow oil, which solidified upon standing, with identical spectra to **S20** (29.1 mg, 94%, 0.0% ee); Chiral SCFC (Chiralpak IG, 250 × 4.6 mm, 5 µm, 40 °C, EtOH (0.2% v/v NH_3)/CO_2 = 15%, 4 mL/min, λ = 210-400 nm): tR [(*R*/*S*)-**S20**] = 1.69 min [(*S*)-**S20**], 2.02 min [(*R*)-**S20**].

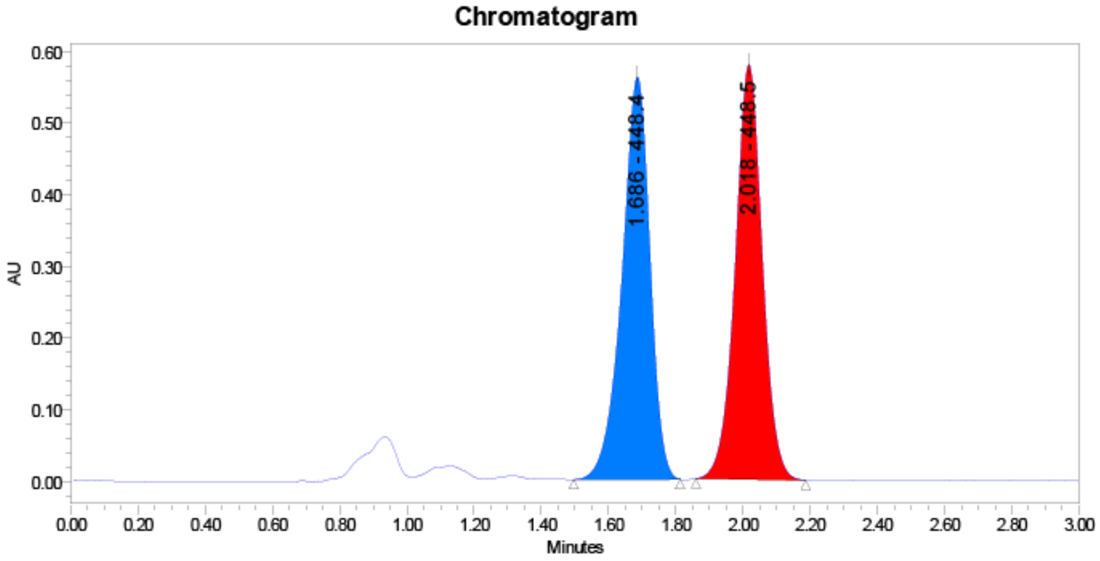

Peak Results

	Retention Time (min)	Area (µV*sec)	% Area	Width @ 50%
1	1.69	3234804	50.0	0.08670
2	2.02	3238594	50.0	0.08445

Figure S77. Chiral SCFC chromatogram of (*R*/*S*)-**S20**

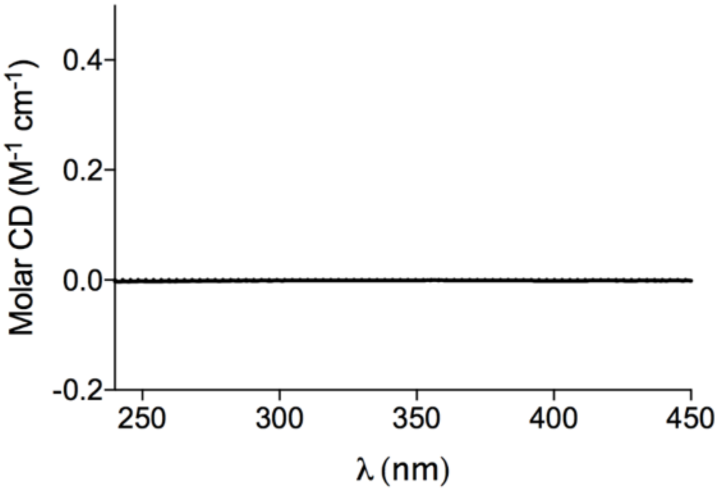

Figure S87. Circular dichroism spectrum of (*R*/*S*)-**S20** (92.7 μM in $CHCl_3$).

Rotaxane (*S,S$_{mp}$*)-**4**

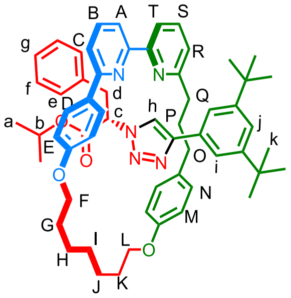

A dry sealed vessel was charged with **2a** (27.6 mg, 0.129 mmol), (*S*)-**3e** (30.0 mg, 0.129 mmol), macrocycle **1** (18.7 mg, 0.0400 mmol), [Cu(MeCN)$_4$]PF$_6$ (14.3 mg, 0.129 mmol), DIPEA (27 µL, 0.160 mmol), and anhydrous CH_2Cl_2 (1 mL). The reaction mixture was stirred at rt for 16 h, protected by an argon atmosphere. TFA (0.20 mL, 2.61 mmol) was added and the reaction mixture was stirred for an additional 16 h. Saturated EDTA-NH_3 solution (10 mL) was added, and the aqueous layer was extracted with $CHCl_3$ (3 × 30 mL), dried over $MgSO_4$, filtered, and had the solvent removed *in vacuo*. The residue containing (*S,S$_{mp}$*)-**4** (in a 0.98 : 0.02 diastereoisomeric ratio by ^{1}H NMR, **Figure S79**) was purified by chromatography (petrol with 0→100% CH_2Cl_2, followed by 0→10% EtOH), to yield (*S,S$_{mp}$*)-**4** as a white foam (32.8 mg, 88%, 0.98 : 0.02 diastereoisomeric ratio **Figure S80**, 99.4% ee); ^{1}H NMR (500 MHz, $CDCl_3$, 298 K) δ 9.99 (s, 1H, H_h *(major)*), 9.92 (s, 1H, H_h *(minor)*),* 7.80-7.71 (m, 4H, $H_{i,}$ H_S and H_B), 7.63 (d, 1H, *J* = 7.7, H_A), 7.58 (d, 1H, *J* = 7.6, H_T), 7.50 (d, 1H, *J* = 7.7, H_C), 7.29 (d, 1H, *J* = 7.6, H_R), 7.26 (d, 2H, *J* = 8.8, H_D), 7.20 (t, 1H, *J* = 1.6, H_j), 7.15-7.09 (m, 3H, H_f and H_g), 6.75-6.67 (m, 2H, H_e), 6.46 (d, 2H, *J* = 8.6, H_N), 6.31 (d, 2H, *J* = 8.8, H_E), 6.24 (d, 2H, *J* = 8.6, H_M), 4.82 (sept, 1H, *J* = 6.1, H_b), 4.55 (dd, 1H, *J* = 12.1, 4.6, H_c), 4.29-4.22 (m,1H, one of H_F), 4.02 (t, 2H, *J* = 6.7, H_Q), 3.94-3.85 (m, 1H, one of H_F), 2.88 (dd, 1H, *J* = 13.0, 12.1, one of H_d), 2.84-2.71 (m, 3H, one of H_d and H_O), 2.51-2.48 (m, 1H, one of H_L), 2.45-2.36 (m, 1H, one of H_L), 2.16-1.58 (m, 12H, H_G, H_H, H_I, H_J, H_K and H_P), 1.19 (s, 18H, H_k), 1.14 (d, 3H, *J* = 6.1, three of H_a), 0.78 (d, 3H, *J* = 6.1, three of H_a); ^{13}C NMR (126 MHz, $CDCl_3$, 298 K) δ 167.8, 164.0, 159.3, 159.1, 158.0, 157.6, 157.1, 149.8, 147.1, 137.1, 137.10, 135.8, 131.6, 131.5, 131.1, 129.2, 128.6, 2 × 128.4, 126.9, 123.4, 122.8, 120.5, 120.3, 120.0, 119.9, 119.5, 114.9, 113.9, 69.0, 68.2, 65.7, 63.5, 37.8, 37.9, 35.5, 34.9, 32.5, 31.6, 29.2, 29.0, 28.3, 25.7, 25.5, 21.7, 21.4.; LR-ESI-MS (+ve) *m/z* (%) = 926.1 [M+H]$^+$ (100); HR-ESI-MS (+ve) *m/z* = 926.5587 [M+H]$^+$ calc. 926.5579; Chiral SCFC (LUX C1, 250 × 4.6 mm, 5 µm, 40 °C, MeOH (0.2% v/v NH_3)/CO_2 = 10→50%, 4 mL/min, λ = 210-400 nm): tR [(*R*,R*$_{mp}$*)-**4**] =4 .60 min, 4.98 min; tR [(*S,S$_{mp}$*)-**4**] = 4.67.

*Given the high d.r. of rotaxane **4**, only H_h of the minor diastereomer, the signal used to assess diastereoselectivity, was assigned.

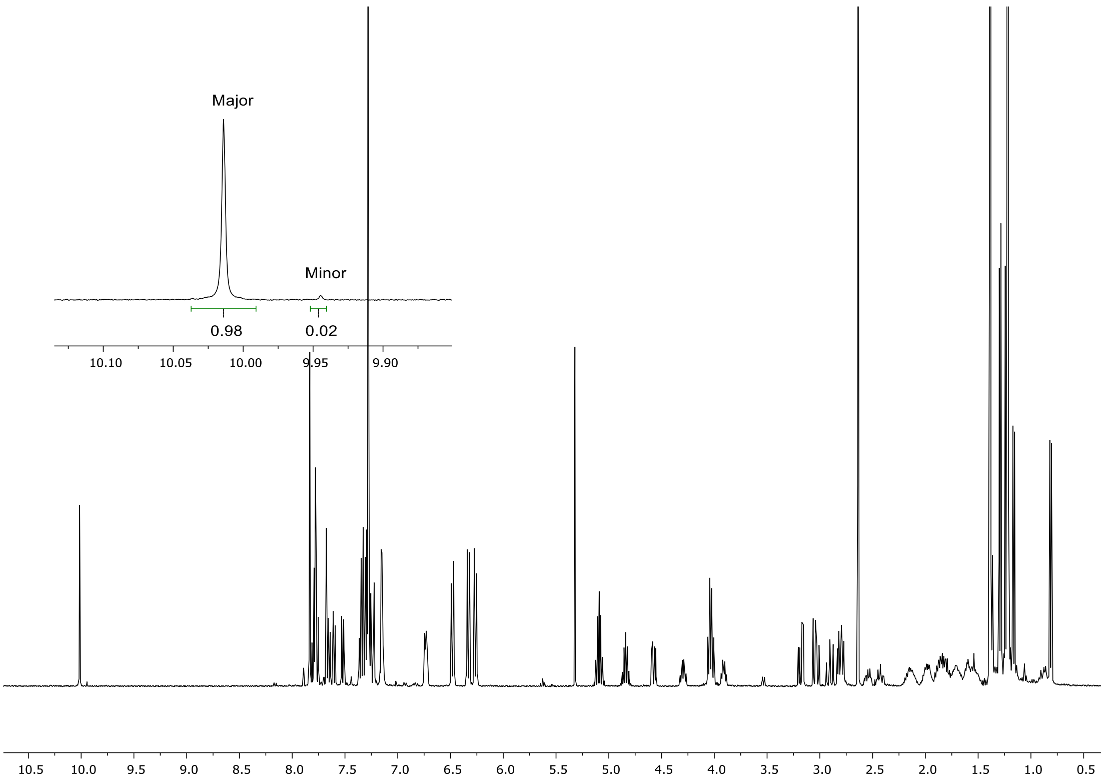

Figure S79. ^{1}H NMR (400 MHz, $CDCl_3$, 298 K) (*S,S$_{mp}$*)-**4** prior to purification by chromatography.

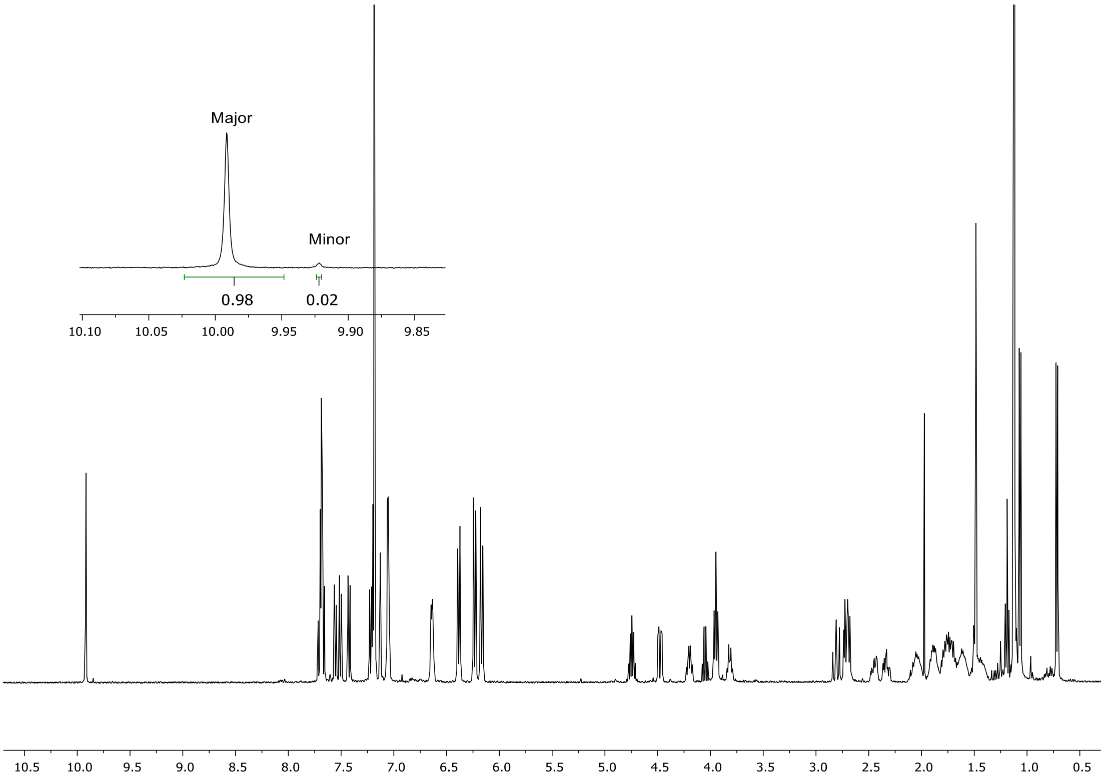

Figure S80. ^{1}H NMR (500 MHz, $CDCl_3$, 298 K) (*S,S$_{mp}$*)-**4**, following purification by chromatography.

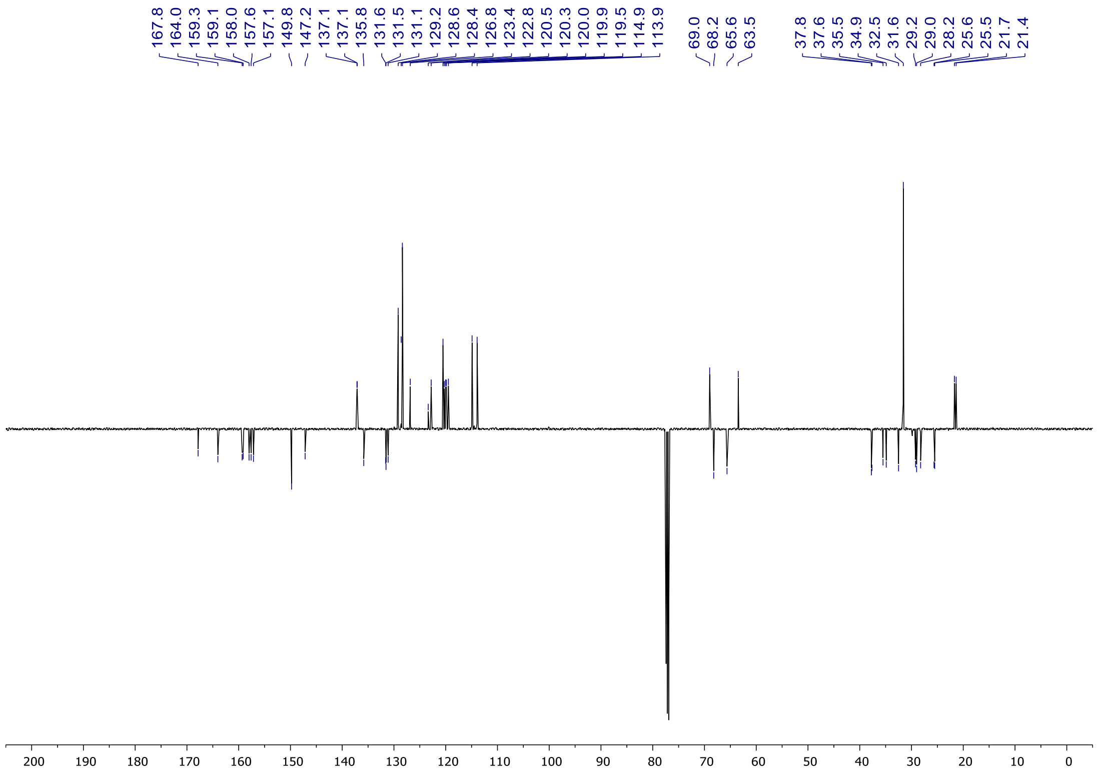

Figure S81. ^{13}C NMR (126 MHz, $CDCl_3$, 298 K) (*S*,*S*$_{mp}$)-**4**, following purification by chromatography.

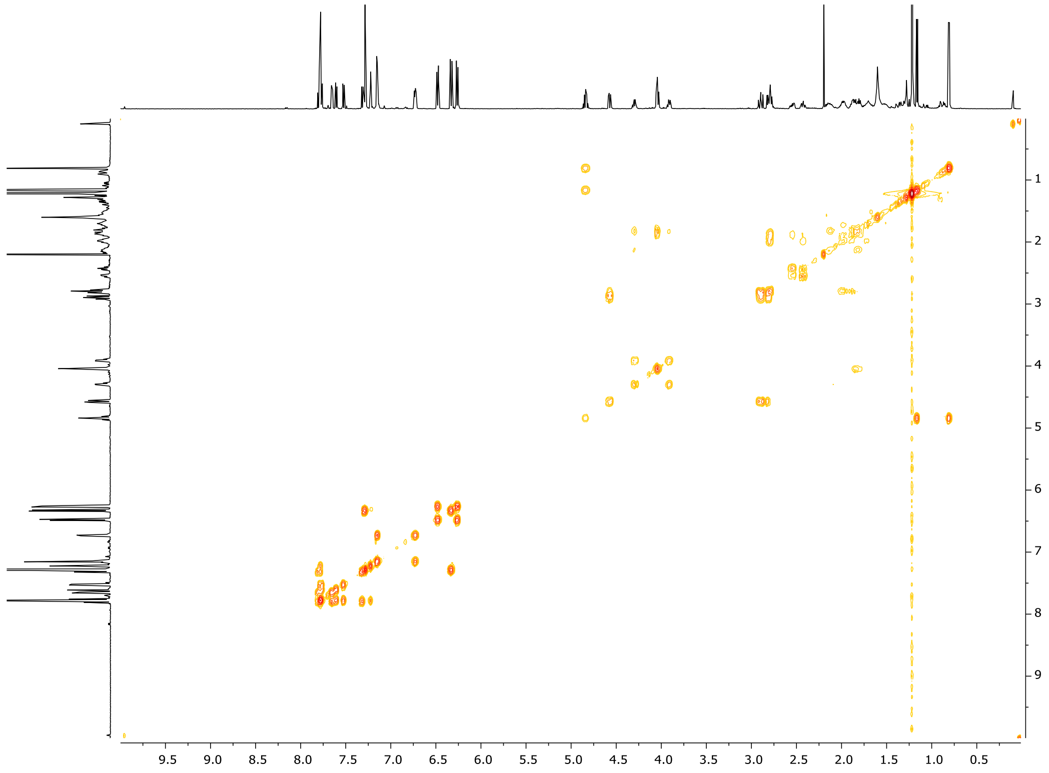

Figure S82. ^{1}H-^{1}H COSY NMR (500 MHz, $CDCl_3$, 298 K) (*S*,*S*$_{mp}$)-**4**, following purification by chromatography.

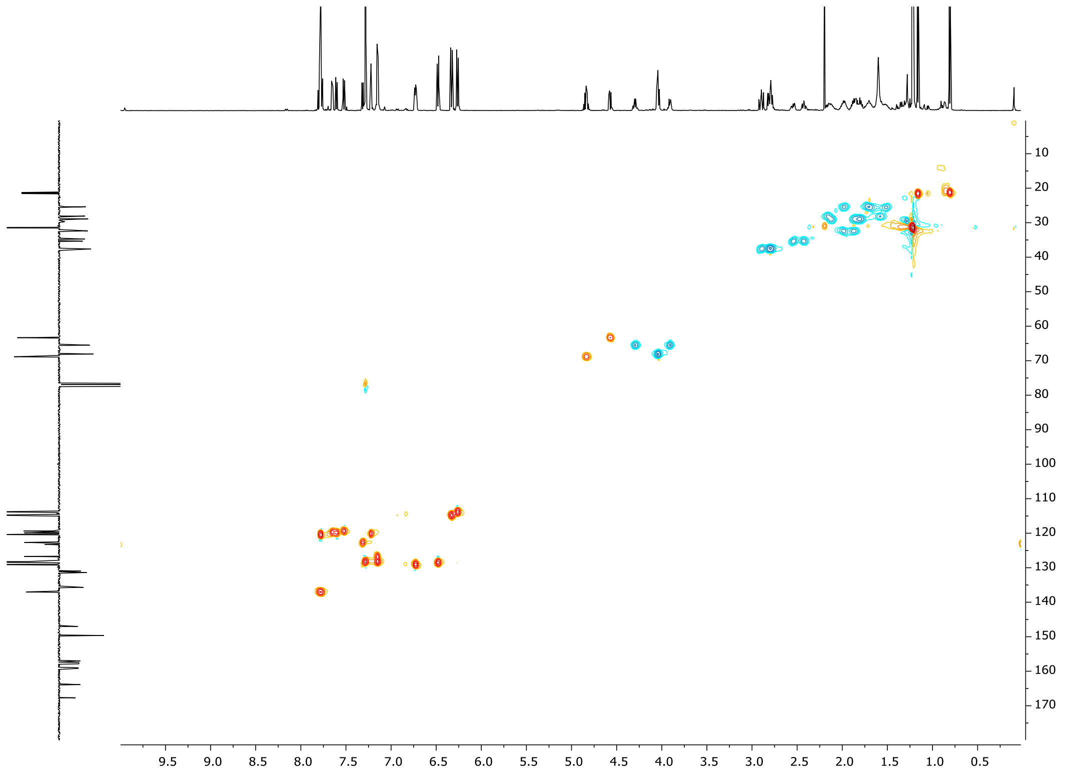

Figure S83. ^{1}H-^{13}C HSQC NMR (126MHz, $CDCl_3$, 298 K) (*S*,*S*$_{mp}$)-**4**, following purification by chromatography.

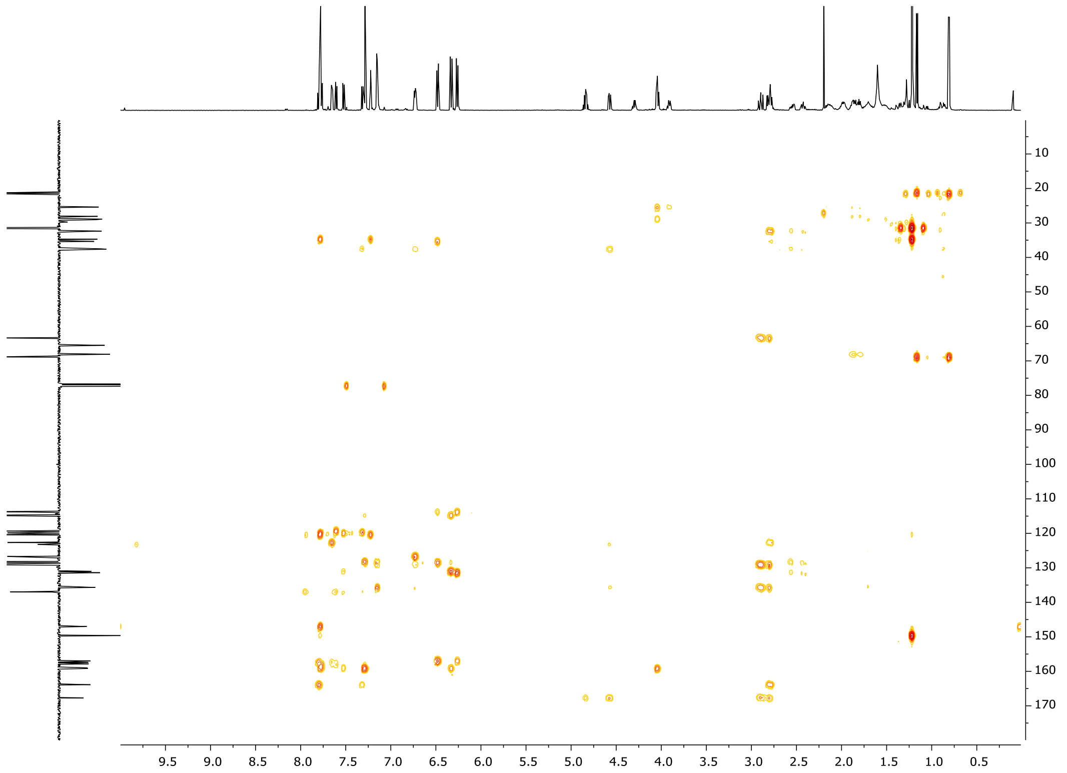

Figure S84. ^{1}H-^{13}C HMBC NMR (126MHz, $CDCl_3$, 298 K) (*S*,*S*$_{mp}$)-**4**, following purification by chromatography.

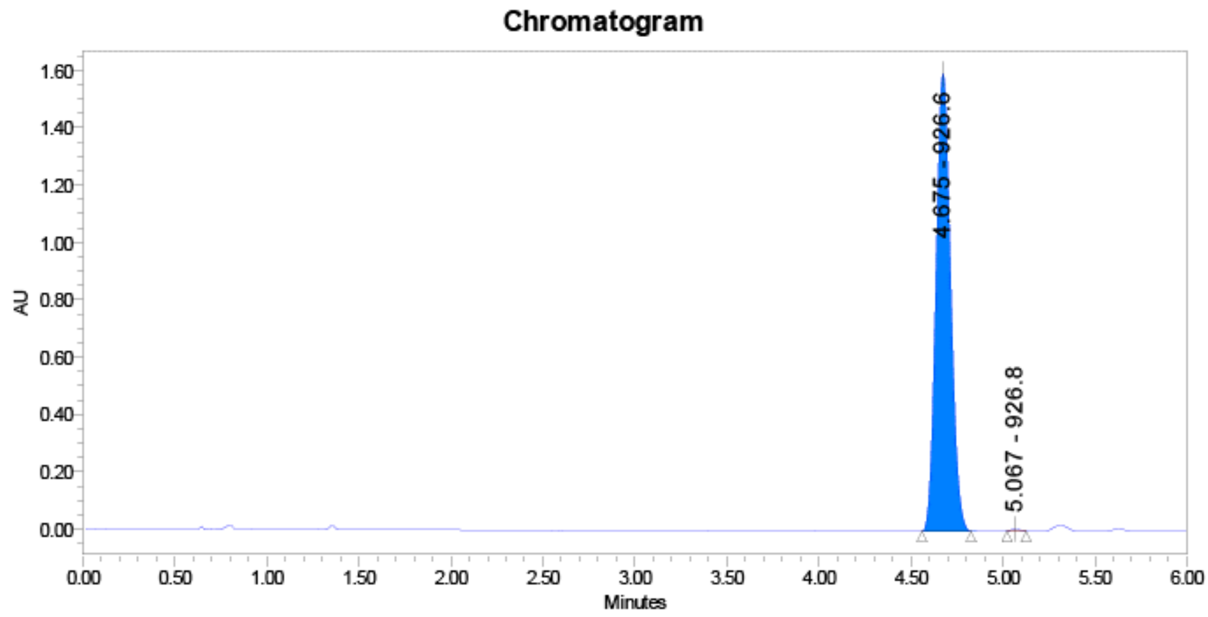

Peak Results

	Retention Time (min)	Area (μV*sec)	% Area	Width @ 50%
1	4.67	8822302	99.7	0.08660
2	5.07	26994	0.3	0.06064

Figure S85. Chiral SCFC chromatogram of (*S,S$_{mp}$*)-**4**, following purification by chromatography.

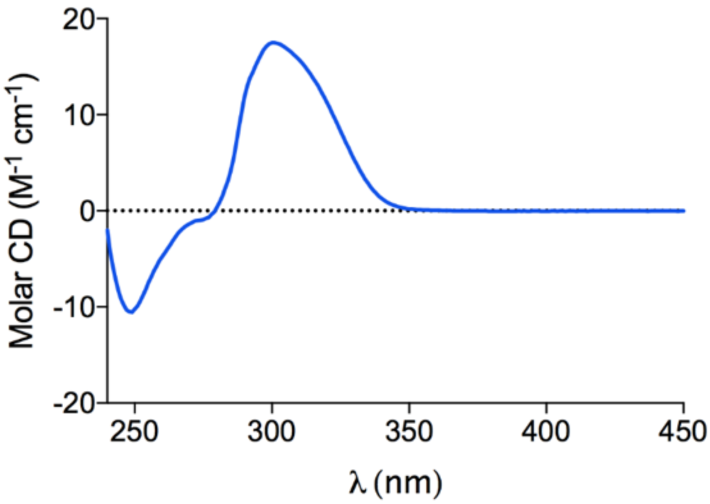

Figure S86. Circular dichroism spectrum of (*S,S$_{mp}$*)-**4** (39.7 μM in $CHCl_3$).

Rotaxane (R,R_{mp})-**4**

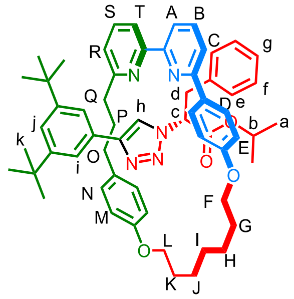

A dry sealed vessel was charged with **2a** (27.2 mg, 0.127 mmol), (R)-**3e** (29.2 mg, 0.125 mmol), macrocycle **1** (28.1 mg, 0.0587 mmol), $[Cu(MeCN)_4]PF_6$ (21.4 mg, 0.0574 mmol), DIPEA (40 µL, 0.225 mmol), and anhydrous CH_2Cl_2 (1.25 mL) was stirred at rt for 16 h, protected by an argon atmosphere. TFA (0.10 mL, 1.31 mmol) was added and the reaction mixture was stirred for an additional 16 h. Saturated EDTA-NH_3 solution (10 mL) was added, and the aqueous layer was extracted with CH_2Cl_2 (3 × 20 mL), dried over $MgSO_4$, filtered, and had the solvent. The residue containing (R,R_{mp})-**4** (in a 0.99 : 0.01 diastereoisomeric ratio by ^{1}H NMR, **Figure S87**) was purified by chromatography (petrol with 0→100% CH_2Cl_2, followed by 0→10% EtOH), to yield (R,R_{mp})-**4** as a white foam, with identical spectra to (S,S_{mp})-**4**, with the exception of circular dichroism spectra (**Figure S90**) (43.2 mg, 80%, 0.99 : 0.01 diastereoisomeric ratio **Figure S88**, 99.8% ee); Chiral SCFC (LUX C1, 250 × 4.6 mm, 5 µm, 40 °C, MeOH (0.2% v/v NH_3)/CO_2 = 10→50%, 4 mL/min, λ = 210-400 nm): tR [(R^*,R^*_{mp})-**4**] = 4.60 min, 4.98 min; tR [(R,R_{mp})-**4**] = 5.01.

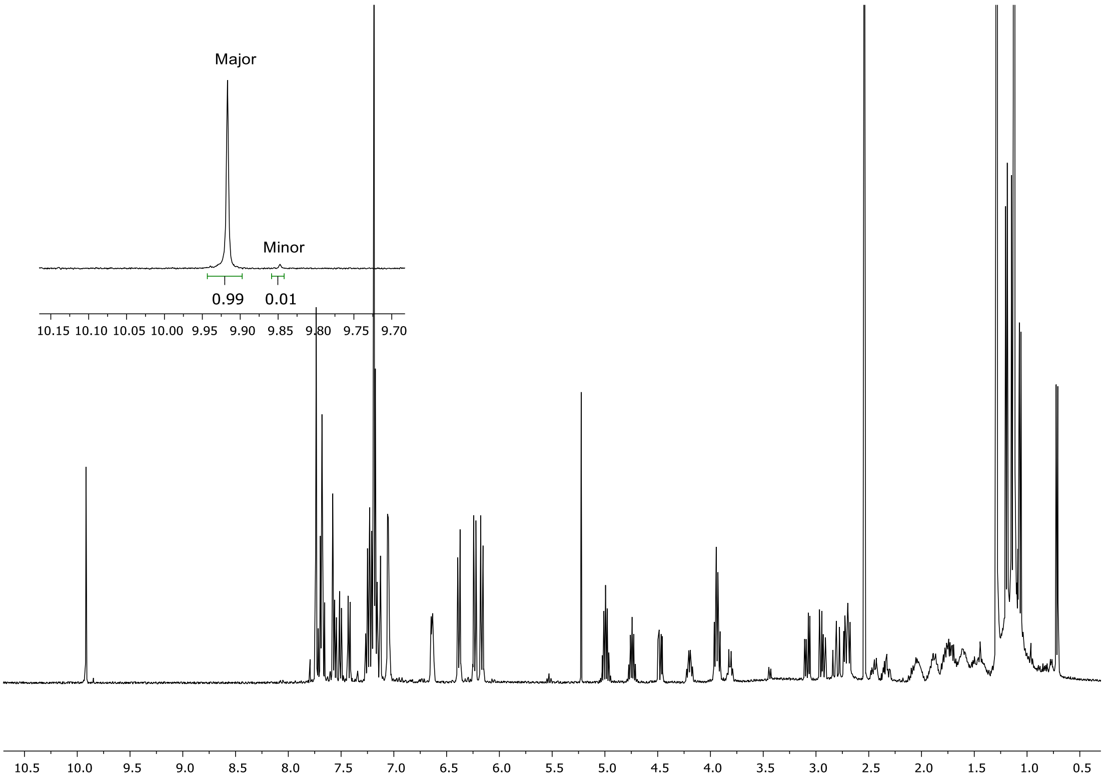

Figure S87. ^{1}H NMR (400 MHz, $CDCl_3$, 298 K) (R,R_{mp})-**4** prior to purification by chromatography.

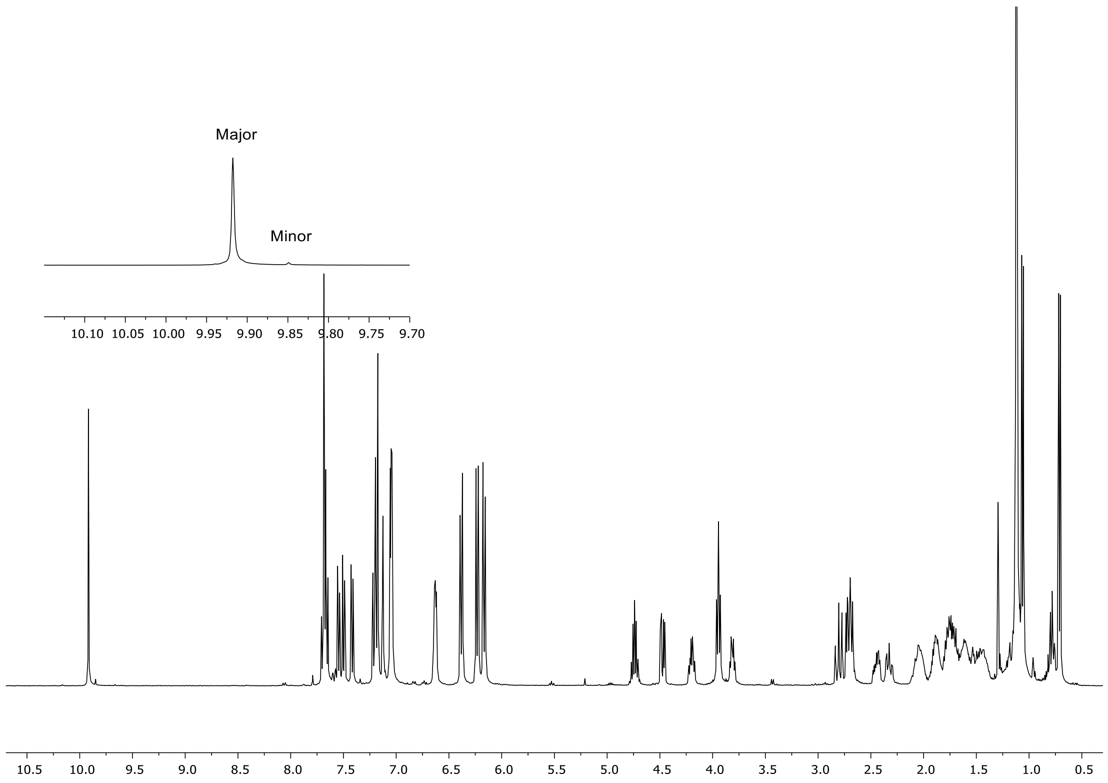

Figure S88. ^{1}H NMR (500 MHz, $CDCl_3$, 298 K) (*R*,*R*$_{mp}$)-**4**, following purification by chromatography.

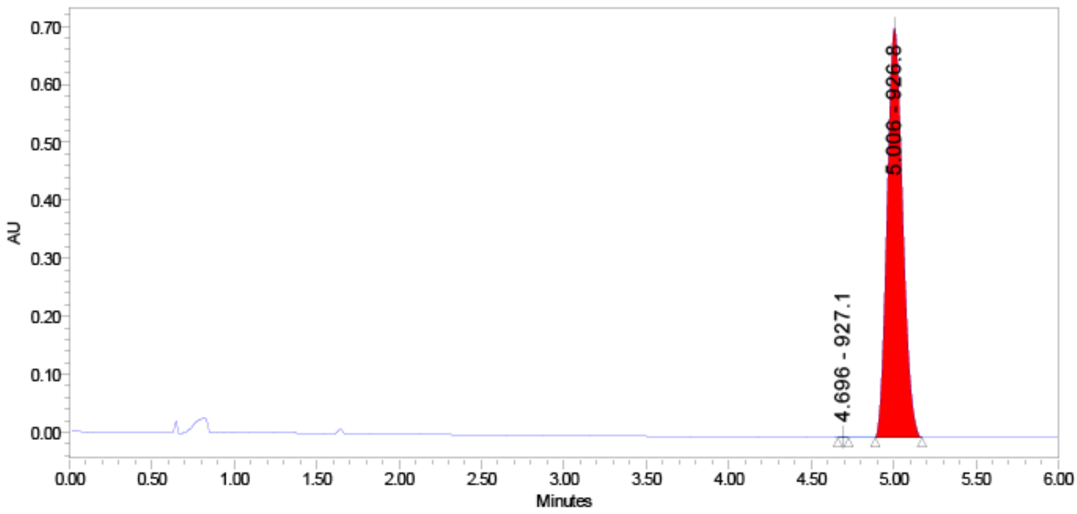

Peak Results

	Retention Time (min)	Area (μV*sec)	% Area	Width @ 50%
1	4.70	2440	0.1	0.03623
2	5.01	4656897	99.9	0.10400

Figure S89. Chiral SCFC chromatogram of (*R*,*R*$_{mp}$)-**4**, following purification by chromatography.

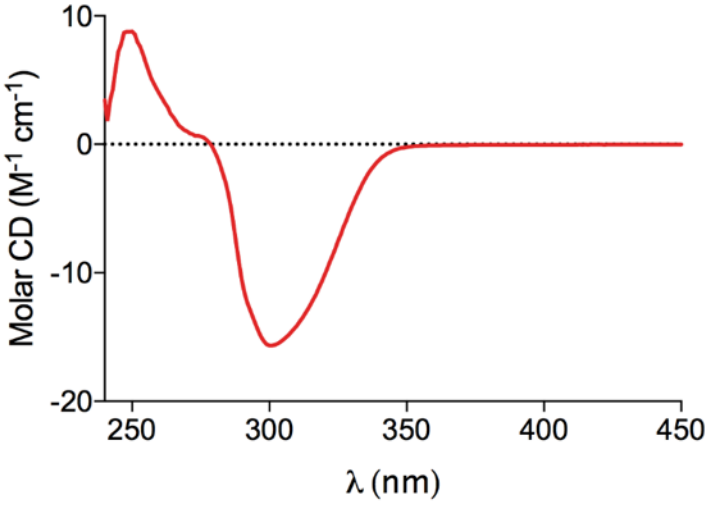

Figure S90. Circular dichroism spectrum of *(R,R$_{mp}$)*-**4** (90.7 µM in $CHCl_3$).

Rotaxanes (*R*,R**$_{mp}$)-**4**

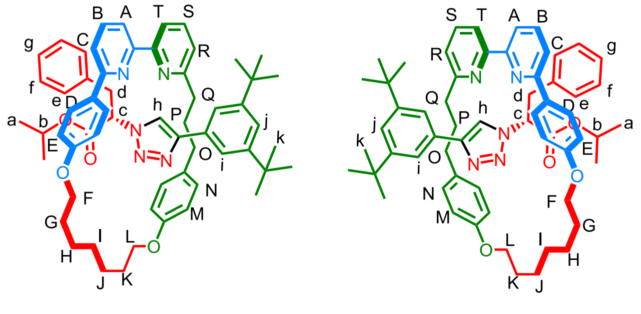

A dry sealed vessel was charged with **2a** (31.4 mg, 0.147 mmol), (*R*/*S*)-**3e** (34.2 mg, 0.147 mmol), macrocycle **1** (34.1 mg, 0.0710 mmol), $[Cu(MeCN)_4]PF_6$ (26.4 mg, 0.0708 mmol), DIPEA (48 µL, 0.285 mmol), and anhydrous CH_2Cl_2 (1.75 mL). The reaction mixture was stirred at rt for 16 h, protected by an argon atmosphere. TFA (0.15 mL, 1.96 mmol) was added and the reaction mixture was stirred for an additional 16 h. Saturated EDTA-NH_3 solution (30 mL) was added, and the aqueous layer was extracted with CH_2Cl_2 (3 × 20 mL), dried over $MgSO_4$, filtered, and had the solvent removed *in vacuo*. The residue containing (*R*,R**$_{mp}$)-**4** (in a 0.99 : 0.01 diastereoisomeric ratio by ^{1}H NMR, **Figure S91**) was purified by chromatography (petrol with 0→100% CH_2Cl_2, followed by 0→10% EtOH), to yield (*R*,R**$_{mp}$)-**4** as a white foam, with identical spectra to (*R,R*$_{mp}$)-**4** and (*S,S*$_{mp}$)-**4** (42 mg, 64%, 0.98 : 0.02 diastereoisomeric ratio, **Figure S92**, 0.0% ee); Chiral SCFC (LUX C1, 250 × 4.6 mm, 5 µm, 40 °C, MeOH (0.2% v/v NH_3)/CO_2 = 10→50%, 4 mL/min, λ = 210-400 nm): tR [*(R*,R**$_{mp}$)-**4**] = 4.60 min [(*S,S*$_{mp}$)-**4**], 4.98 min [(*R,R*$_{mp}$)-**4**].

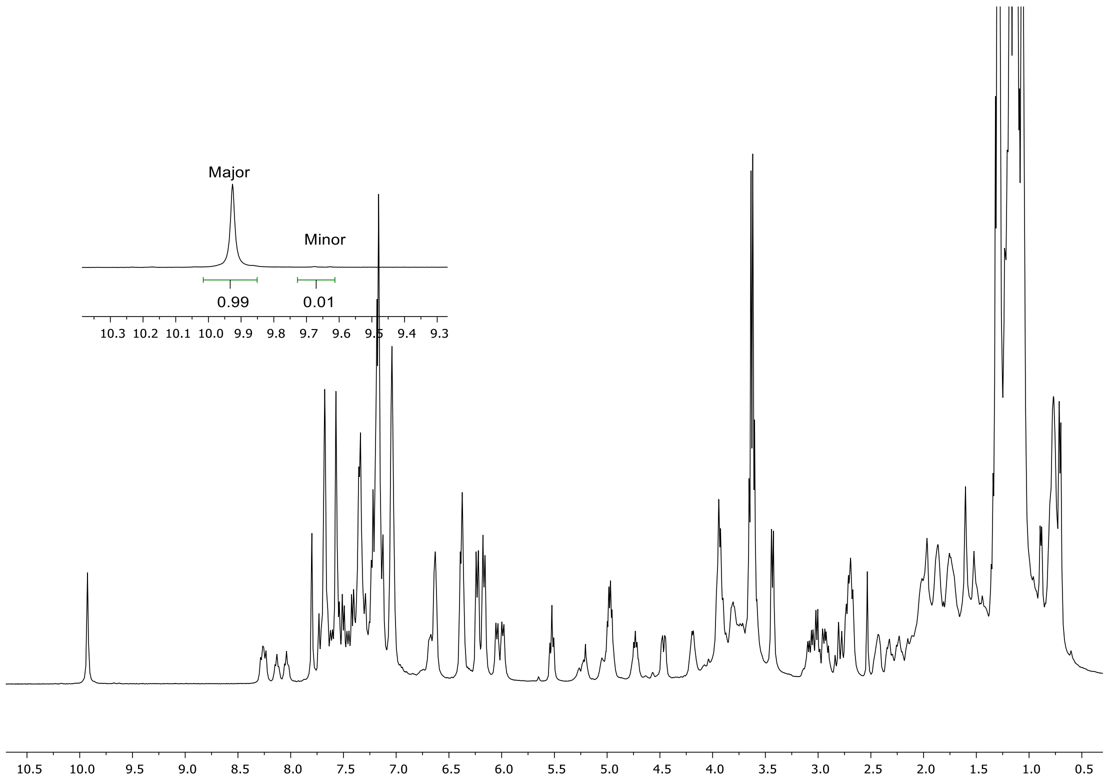

Figure S91. ^{1}H NMR (400 MHz, $CDCl_3$, 298 K) *(R*,R*$_{mp}$)*-**4** prior to purification by chromatography.

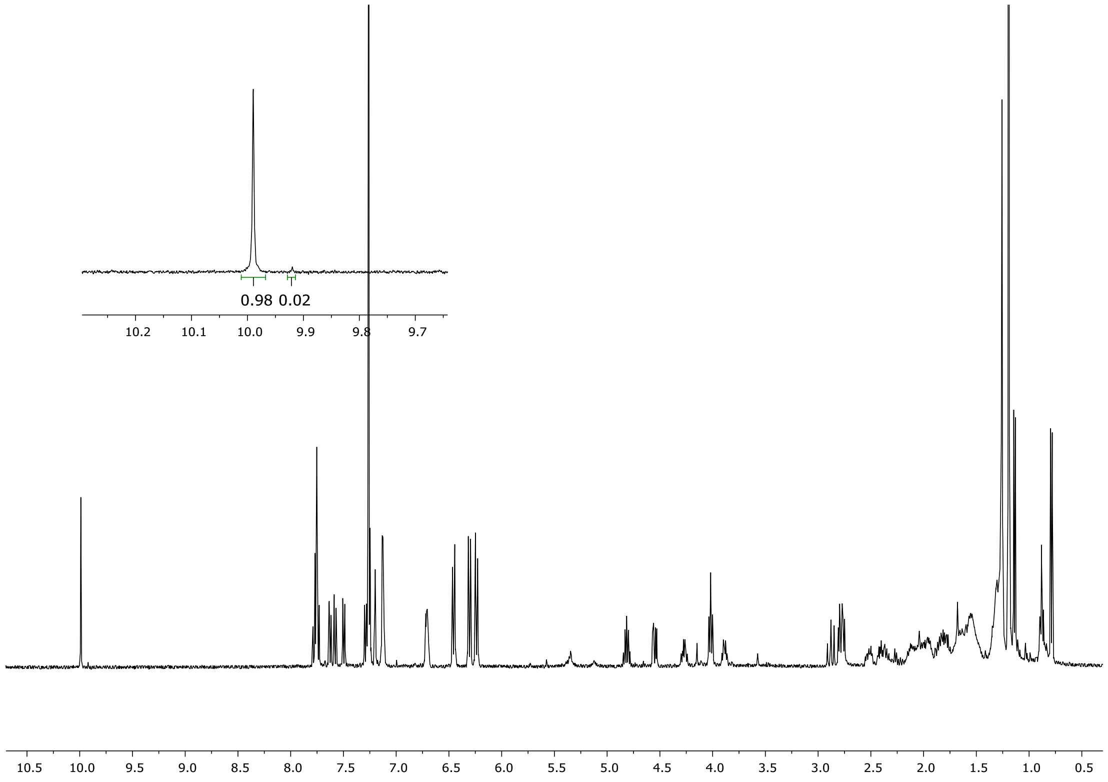

Figure S92. ^{13}C NMR (126 MHz, $CDCl_3$, 298 K) *(R*,R*$_{mp}$)*-**4**, following purification by chromatography.

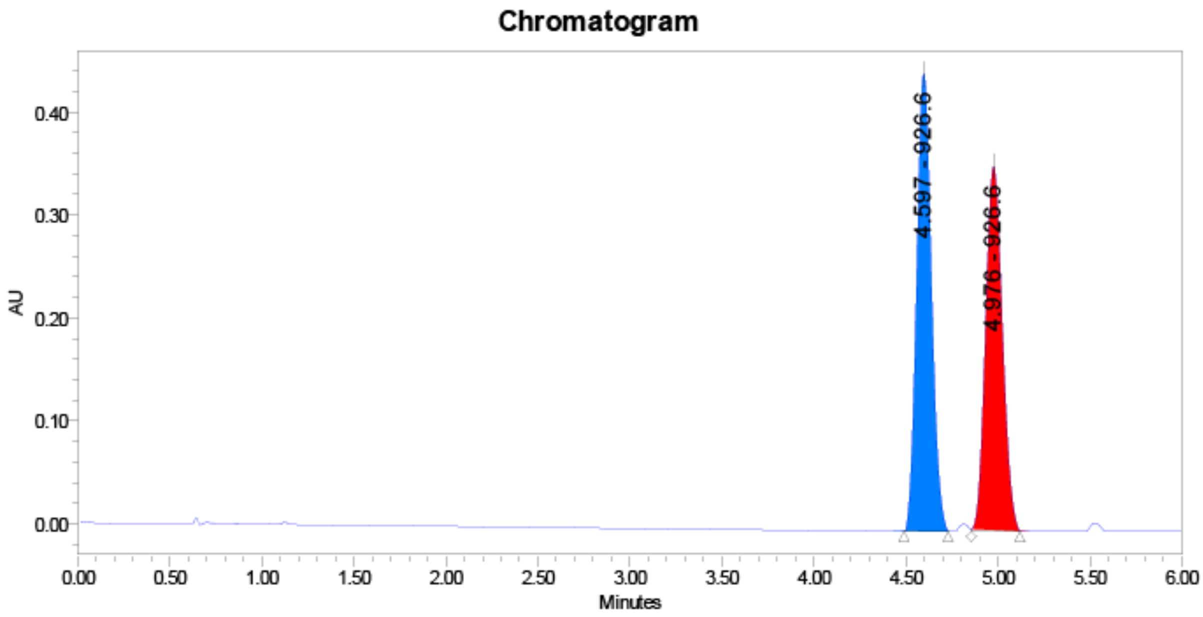

Peak Results

	Retention Time (min)	Area (µV*sec)	% Area	Width @ 50%
1	4.60	2439912	52.0	0.08551
2	4.98	2256298	48.0	0.10018

Figure S93. Chiral SCFC chromatogram of (R^*,R^*_{mp})-**4**, following purification by chromatography.

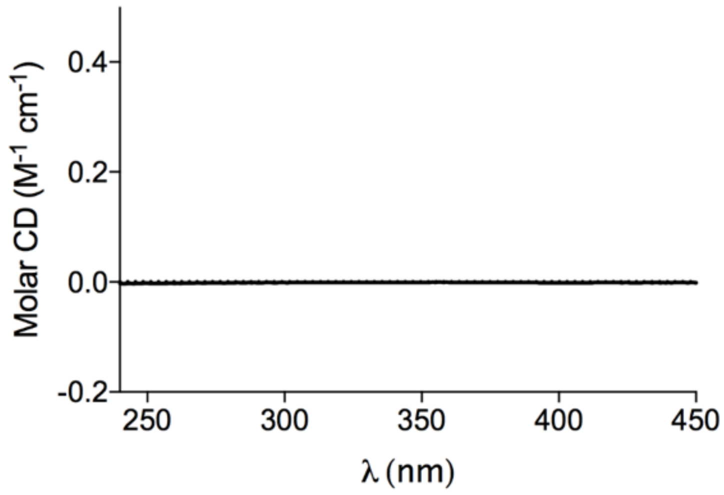

Figure S94. Circular dichroism spectrum of (R^*,R^*_{mp})-**4** (92.7 µM in $CHCl_3$).

In order to identify the signals corresponding to the minor diastereoisomer of **4**, the AT-CuAAC reaction was performed at 100 °C.

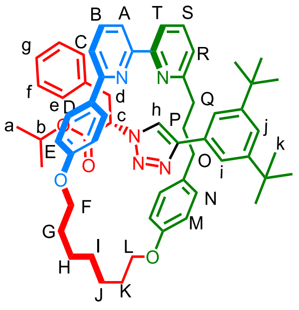

(*S*,*S*$_{mp}$)-**4**

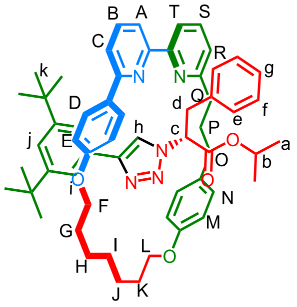

(*S*,*R*$_{mp}$)-**4**

A sealed vessel was charged with **2a** (17.3 mg, 0.081 mmol), (*S*)-**3e** (18.9 mg, 0.081 mmol), macrocycle **1** (19.4 mg, 0.040 mmol), $[Cu(MeCN)_4]PF_6$ (14.5 mg, 0.039 mmol), DIPEA (28.2 μL, 0.16 mmol), and EtOH (1 mL) and stirred at 100 ºC for 2 h, protected by an argon atmosphere in a microwave (70 W). The solvent was removed *in vacuo*, and the residue was re-dissolved in CH_2Cl_2 (1 mL) and TFA (0.10 mL, 1.305 mmol) was added and the reaction mixture was stirred for an additional 16 h. Saturated EDTA-NH_3 (10 mL) was added, and the aqueous layer was extracted with $CHCl_3$ (3 × 30 mL), dried over $MgSO_4$, filtered, and had the solvent removed *in vacuo* to give a yellow solid residue containing (*S*,R$_{mp}$/*S*$_{mp}$)-**4** as a mixture of diastereomers (58 : 42 ratio by ^{1}H NMR, **Figure S95**).

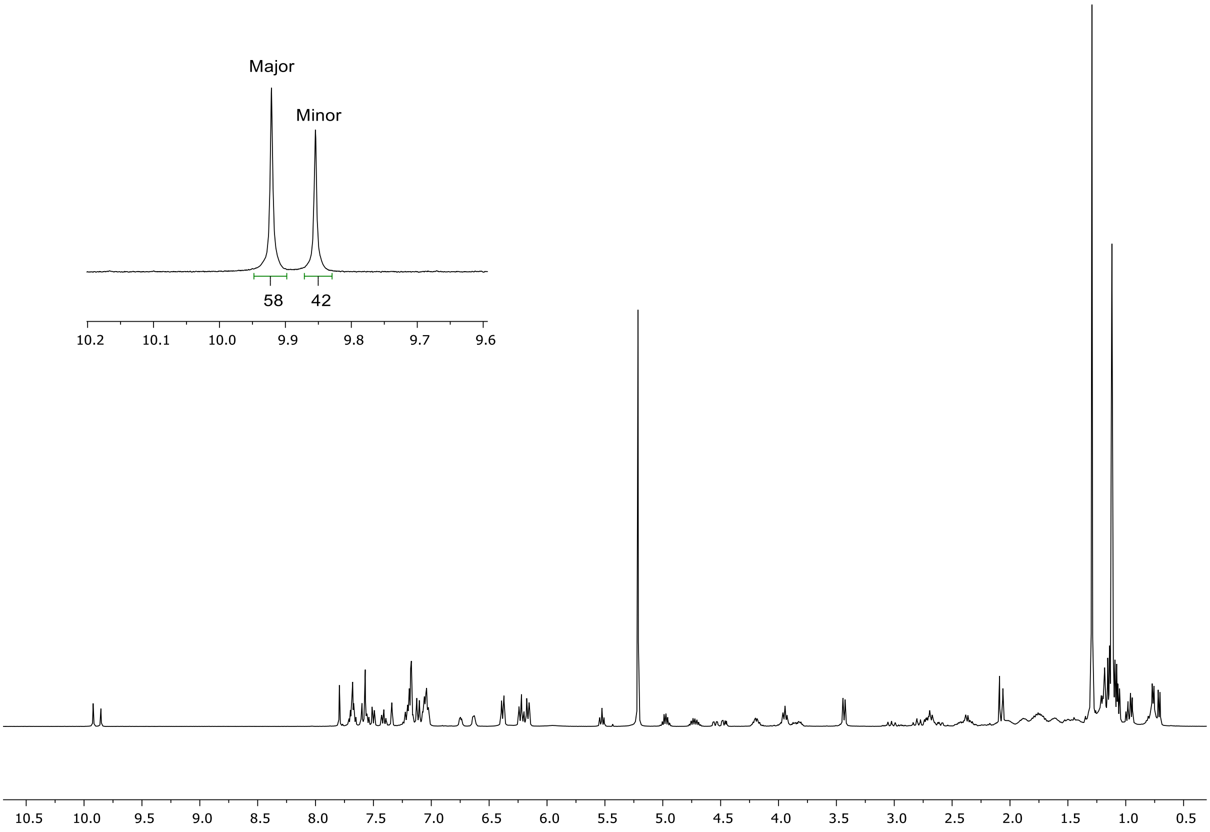

Figure S95. ^{1}H NMR (400 MHz, $CDCl_3$, 298 K) (*S*,R$_{mp}$/*S*$_{mp}$)-**4** (unpurified) when the reaction was performed at 100 °C.

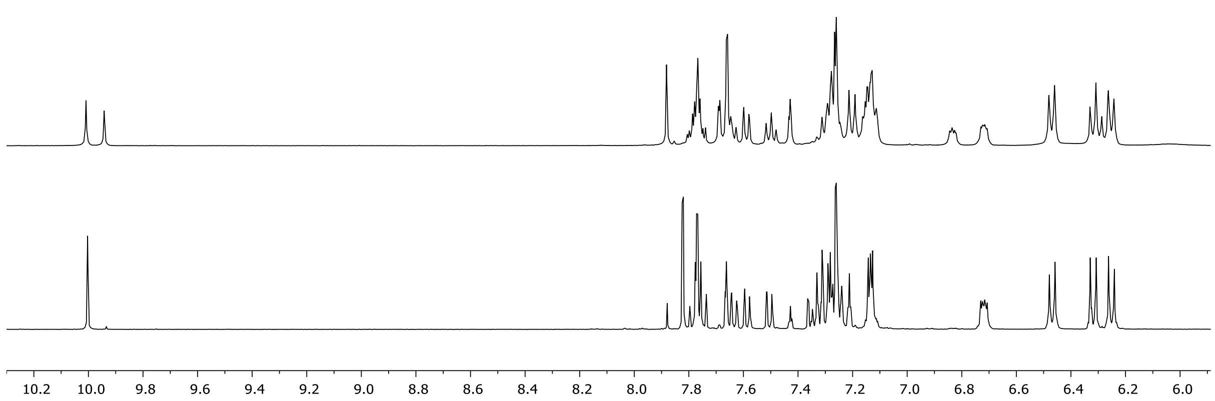

Figure S96. ^{1}H NMR (400 MHz, $CDCl_3$, 298 K) (unpurified samples) a) (S,R$_{mp}$/S_{mp})-**4** (reaction at 100 °C) and b) (S,S_{mp})-**4** (reaction at rt).

Entry 7 – axle (*S*)-S21 and rotaxane S22 derived from alkyne 2d and azide (*S*)-3e

Axle (*S*)-**S21**

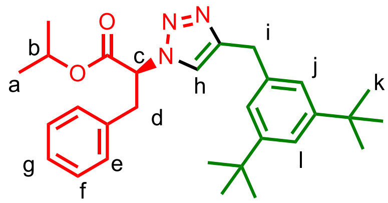

A 5 mL round bottom flask was charged with **2d** (15.4 mg, 0.0692 mmol), (*S*)-**3e** (16.9 mg, 0.0724 mmol), $CuSO_4.5H_2O$ (17.2 mg, 0.0691 mmol), sodium *L*-ascorbate (17.8 mg, 0.0898 mmol), and DMF (3 mL). The reaction mixture was stirred at rt for 16 h. Saturated EDTA-NH_3 solution (20 mL) was added, and the aqueous layer was extracted with EtOAc (3 × 20 mL). The combined organic extracts were washed with 5% w/v LiCl (5 × 20 mL), brine (20 mL), were dried over $MgSO_4$, filtered, and the solvent removed *in vacuo*. The residue was purified by chromatography (CH_2Cl_2 with 0→10% MeOH), to yield (*S*)-**S21** as a yellow oil (17.3 mg, 54%); ^{1}H NMR (400 MHz, $CDCl_3$, 298 K) δ 7.30 (s, 1H, H_h), 7.26 (t, 1H, *J* = 1.5, H_l), 7.22-7.18 (m, 3H, H_f and H_g), 7.07 (d, 2H, *J* = 1.5, H_j), 7.05-7.01 (m, 2H, H_e), 5.54 (dd, 1H, *J* = 7.4, 8.1, H_c), 4.99 (sept, 1H, *J* = 6.2, H_b), 4.09 (d, 1H, *J* = 16, 1H, one of H_i), 4.04 (d, 1H, *J* = 16, one of H_i), 3.44 (dd, 1H, *J* = 14.0, 7.4, one of H_d), 3.38 (dd, 1H, *J* = 14.0, 8.1, one of H_d), 1.30 (s, 18H, H_l), 1.18 (d, 3H, *J* = 6.2, three of H_a), 1.11 (d, 3H, *J* = 6.2, three of H_a); ^{13}C NMR (101 MHz, $CDCl_3$, 298 K) δ 168.0, 151.1, 148.1, 138.1, 135.0, 129.1, 128.8, 127.5, 123.0, 121.5, 120.5, 70.4, 64.2, 39.1, 34.9, 32.9, 31.6, 21.6; LR-ESI-MS (+ve) *m/z* (%) = 462.6 $[M+H]^+$; HR-ESI-MS (+ve) *m/z* = 462.3114 $[M+H]^+$ calc. 462.3115.

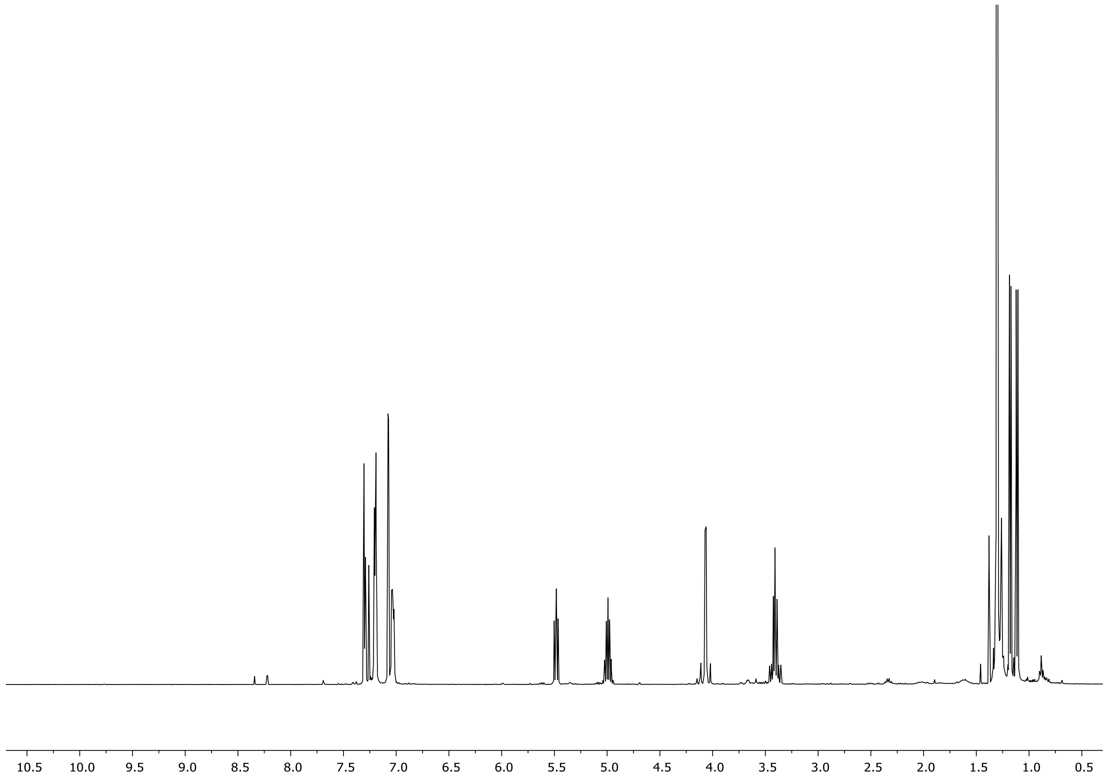

Figure S97. ^{1}H NMR (400 MHz, $CDCl_3$, 298 K) (*S*)-**S21**.

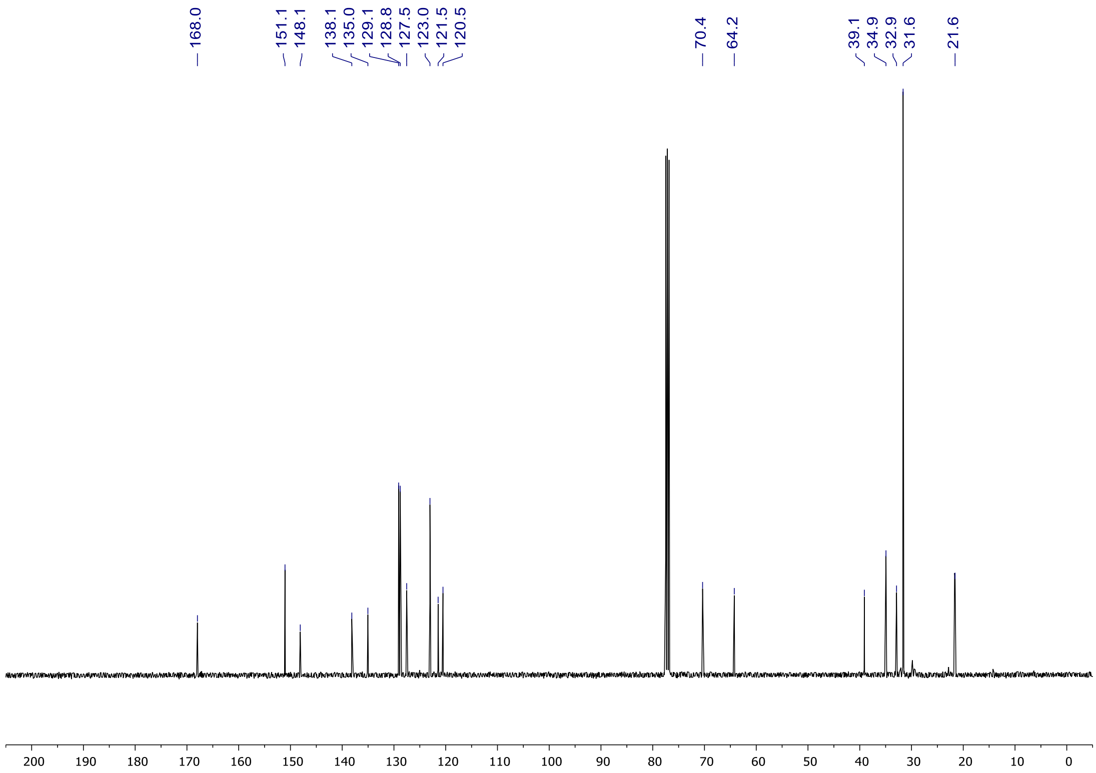

Figure S98. ^{13}C NMR (101 MHz, $CDCl_3$, 298 K) (*S*)-**S21**.

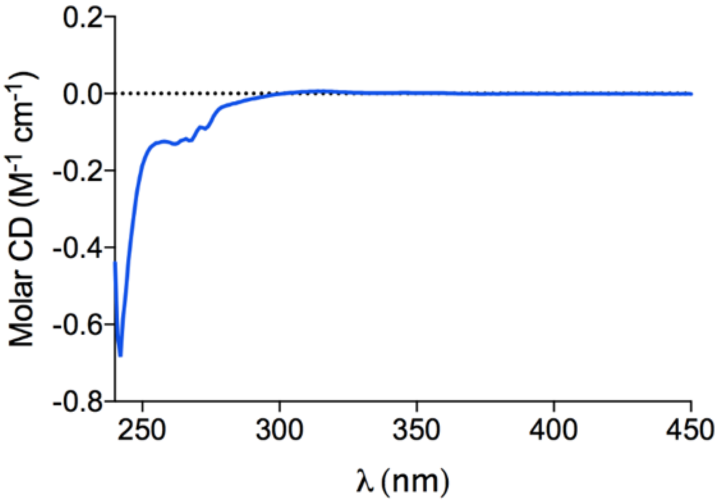

Figure S99. Circular dichroism spectrum of (*S*)-**S21** (1.12 mM in $CHCl_3$).

Rotaxanes (S,R$_{mp}$/S_{mp})-**S22**

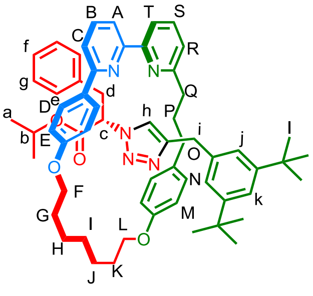

(S,S_{mp})-**S22**

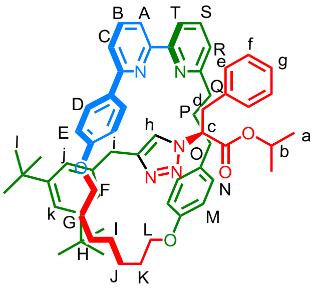

(S,R_{mp})-**S22**

A dry sealed vessel was charged with **2d** (16.5 mg, 0.0722 mmol), (S)-**3e** (16.9 mg, 0.0722 mmol), macrocycle **1** (14.1 mg, 0.0295 mmol), [Cu(MeCN)$_4$]PF$_6$ (10.7 mg, 0.0287 mmol), DIPEA (20 µL, 0.118 mmol), and anhydrous CH_2Cl_2 (1 mL). The reaction mixture was stirred at rt for 16 h, protected by an argon atmosphere. TFA (0.10 mL, 1.31 mmol) was added and the reaction mixture was stirred for an additional 16 h. Saturated EDTA-NH_3 solution (10 mL) was added, and the aqueous layer was extracted with $CHCl_3$ (3 × 20 mL), dried over $MgSO_4$, filtered, and had the solvent removed *in vacuo*. The residue containing rotaxanes (S,R$_{mp}$/S_{mp})-**S22** (in a 0.14 : 0.86 diastereoisomeric ratio by ^{1}H NMR, **Figure S100**) was purified by chromatography (petrol with 0→100% CH_2Cl_2, followed by 0→10.5% EtOH), to yield rotaxanes (S,R$_{mp}$/S_{mp})-**S22** as a yellow oil (22.2 mg, 80%, 0.40 : 0.60 diastereoisomeric ratio, **Figure S101**); ^{1}H NMR (500 MHz, $CDCl_3$, 298 K)* δ 9.66 (s, 1H, H_h (*minor*)), 9.46 (s, 1H, H_h (*major*)), 7.83-7.75 (m, 2H, H_B and H_S), 7.64 (m, 1H, H_T), 7.61-7.55 (m, 2H, H_A and H_C), 7.33 (dd, 1H, J = 3.0, 0.6, H_R (*major*)), 7.31 (dd, 1H, J = 3.0, 0.4, H_R (*minor*)), 7.14 (d, 2H, J = 1.2, 2H, H_j (*minor*)), 7.11-7.04 (m, 6H, H_f, H_g, H_j (*major*) and H_k), 7.03 (d, 1H, J = 8.8, H_D (*major*)) 6.76 (d, 2H, J = 8.8, H_N (*major*)), 6.74-6.71 (m, 1H, H_e (*minor*)), 6.69 (d, 2H, J = 8.5, H_N (*minor*)), 6.58-6.54 (m, 2H, H_e (*major*)), 7.54-7.49 (m, 2H, H_M), 6.15 (d, 2H, J = 8.9, H_E (*major*)), 6.11 (d, 2H, J = 8.9, H_E (*minor*)), 4.74-4.69 (m, 1H, H_b (*major*)), 4.60-4.54 (m, 1H, H_b (*minor*)), 4.44-4.38 (m, 2H, H_c (*minor*) and one of H_L (*minor*)), 4.11-4.05 (m, 1H, one of H_L (*major*)), 4.03-3.85 (m, 4H, H_F, one of H_L (*major*) and H_L (*minor*)), 3.72 (dd, 1H, J = 12.1, 4.4, H_c (*major*)), 3.57 (d, 1H, J = 14.4, one of H_i (*major*)), 3.54 (d, 1H, J = 14.4, one of H_i (*minor*)), 3.38 (d, 1H, J = 14.4, one of H_i (*major*)), 3.29-3.23 (m, 2H, one of H_d (*major*) and one of H_i (*minor*)), 3.22-3.14 (m, 1H, one of H_d (*minor*)), 3.01-2.78 (m, 3H, one of H_d (*major*) and H_Q), 2.73-2.52 (m, 2H, H_O), 2.37 (dd, 1H, J = 13.6, 4.6, one of H_d (*minor*)), 2.10-1.15 (m, 10H, H_G, H_H, H_I, H_J and H_K), 1.07 (s, 18H, H_l (*major*)), 1.06 (s, 18H, H_l (*minor*)), 1.04 (d, 3H, J = 6.3, three of H_a (*major*)), 0.74 (d, 3H, J = 6.3, three of H_a (*minor*)), 0.70 (d, 3H, J = 6.3, three of H_a (*major*)), 0.54 (d, 3H, J = 6.3, three of H_a (*minor*)); ^{13}C NMR (126 MHz, $CDCl_3$, 298 K) δ 168.4, 167.7, 163.3, 163.3, 159.1, 158.8, 158.4, 158.1, 157.5, 157.5, 157.4, 157.4, 157.4, 157.1, 150.4, 150.3, 144.7, 144.7, 139.3, 139.2, 137.0, 136.9, 136.9, 136.9, 136.0, 135.6, 131.5, 131.3, 130.4, 130.0, 129.1, 129.0, 128.9, 128.9, 128.2, 128.0, 128.0, 127.9, 126.4, 126.3, 125.5, 123.7, 2 × 123.0, 122.3, 122.1, 120.3, 2 × 120.1, 120.0, 119.8, 119.7, 119.1, 118.9, 115.1, 114.4, 114.2, 113.9, 68.5, 68.4, 68.3, 67.8, 65.9, 65.8, 63.2, 62.3, 37.2, 37.2, 37.1, 36.5, 2 × 35.1, 2 × 34.4, 33.5, 33.3, 2 × 32.7, 31.2, 31.1, 28.9, 28.7, 28.5, 28.3, 28.3, 25.6, 25.5, 25.1, 25.0, 21.4, 21.1, 21.0, 20.8.; LR-MS-ESI m/z = (%) 940.2 (100); HR-MS-ESI m/z = 940.5757 [M H+] calc. 940.5735.

*As stereochemistry could not be unambiguously assigned, the signals are simply designated (*major*) or (*minor*). Proton counts are provided for each signal and represent the expected integration of that environment. Where the major and minor diastereoisomer signals are coincident, no (*major*)/(*minor*) label is provided and the proton count indicated refers the expected integration of that signal in each of the stereoisomers that contributes to the multiplet.

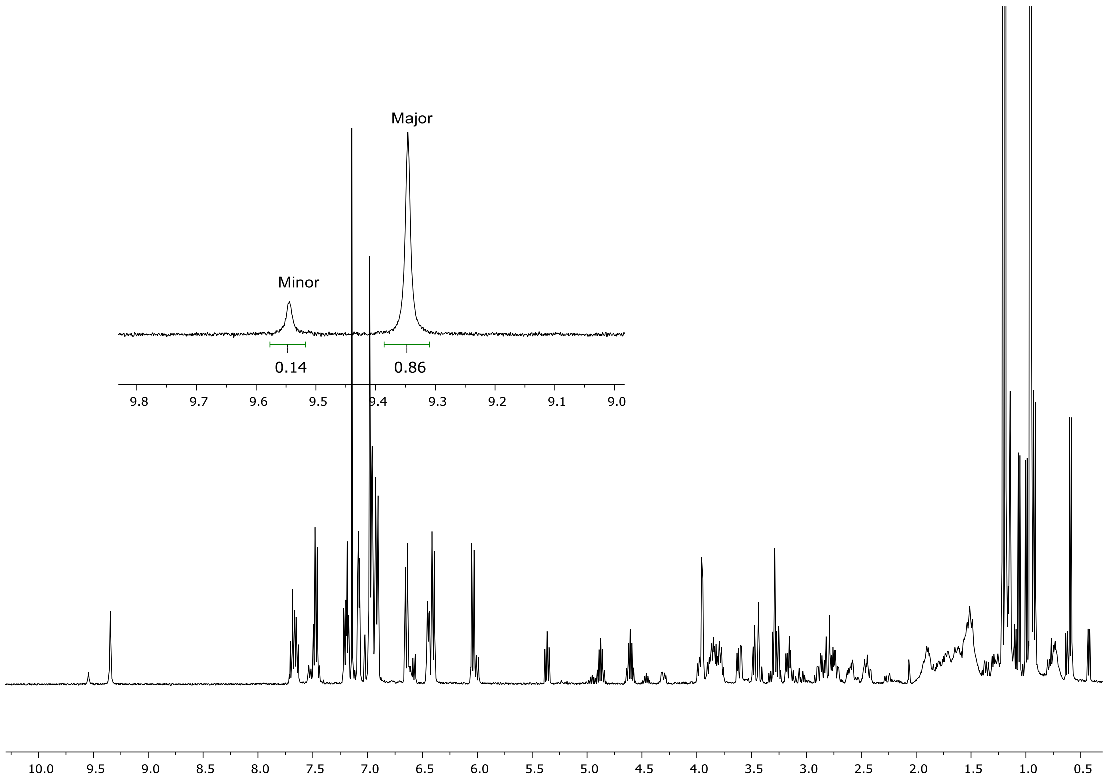

Figure S100. ^{1}H NMR (400 MHz, $CDCl_3$, 298 K) (S,R$_{mp}$/S_{mp})-**S22** prior to purification by chromatography.

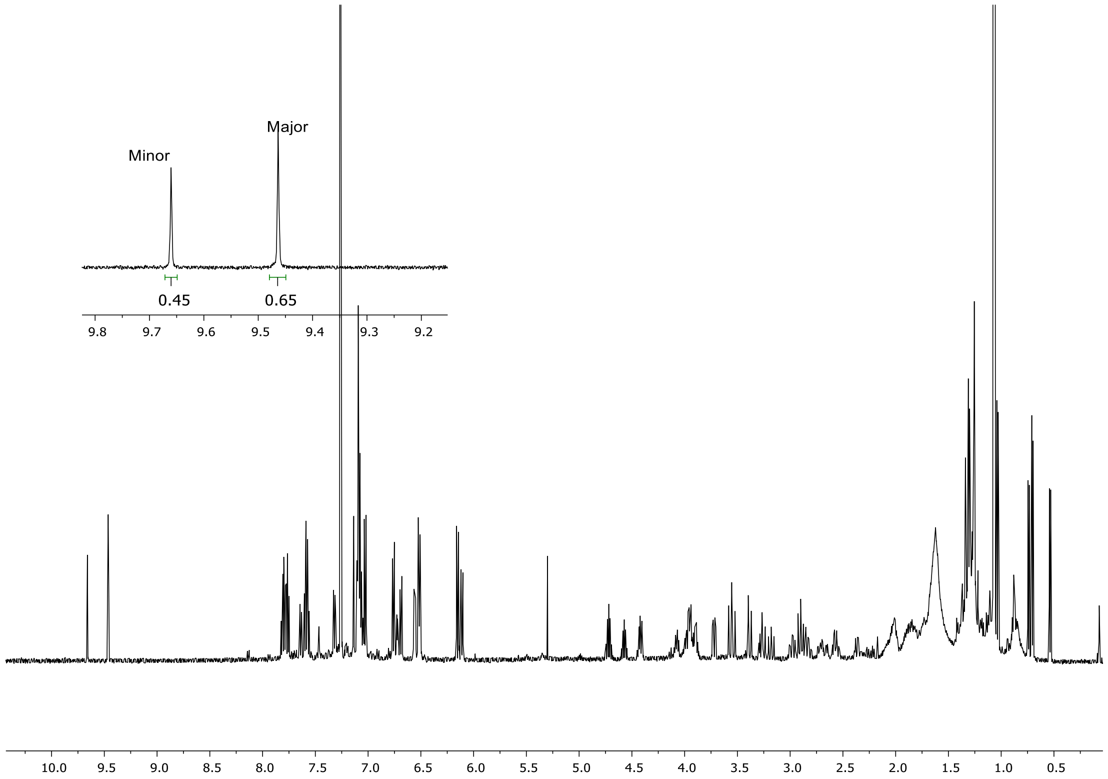

Figure S101. ^{1}H NMR (500 MHz, $CDCl_3$, 298 K) (S,R$_{mp}$/S_{mp})-**S22**, following purification by chromatography.

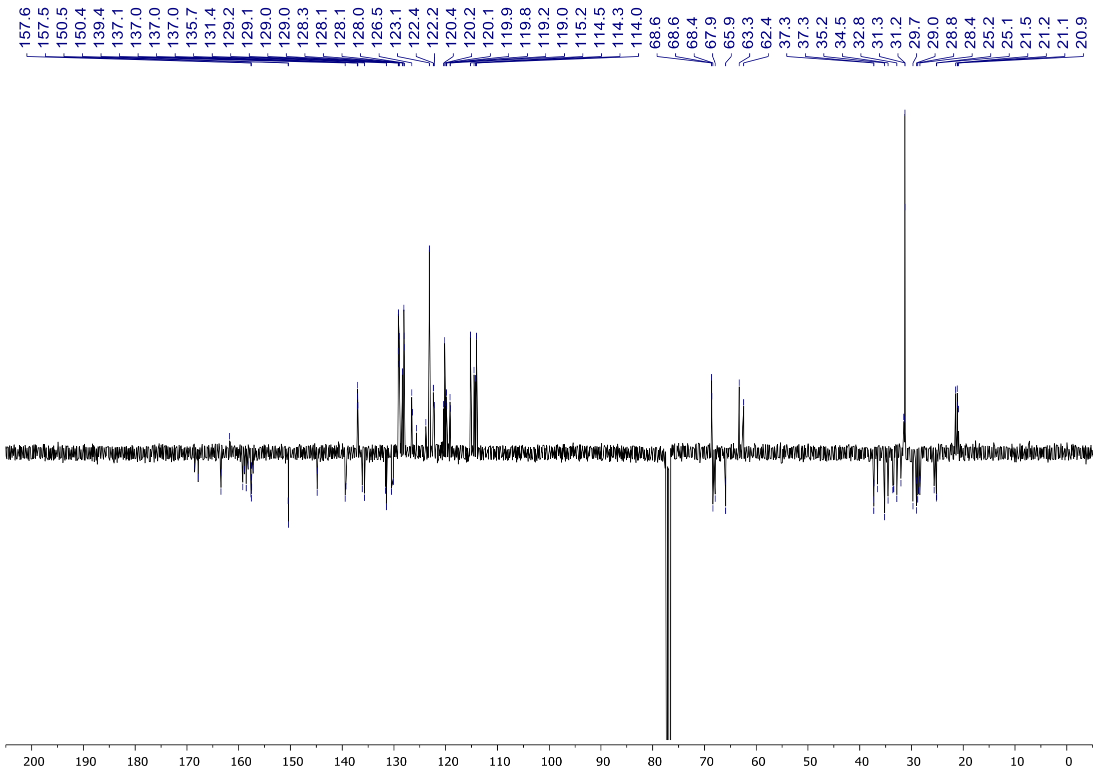

Figure S102. ^{13}C NMR (126 MHz, $CDCl_3$, 298 K) (*S*,R$_{mp}$/*S*$_{mp}$)-**S22**, following purification by chromatography.

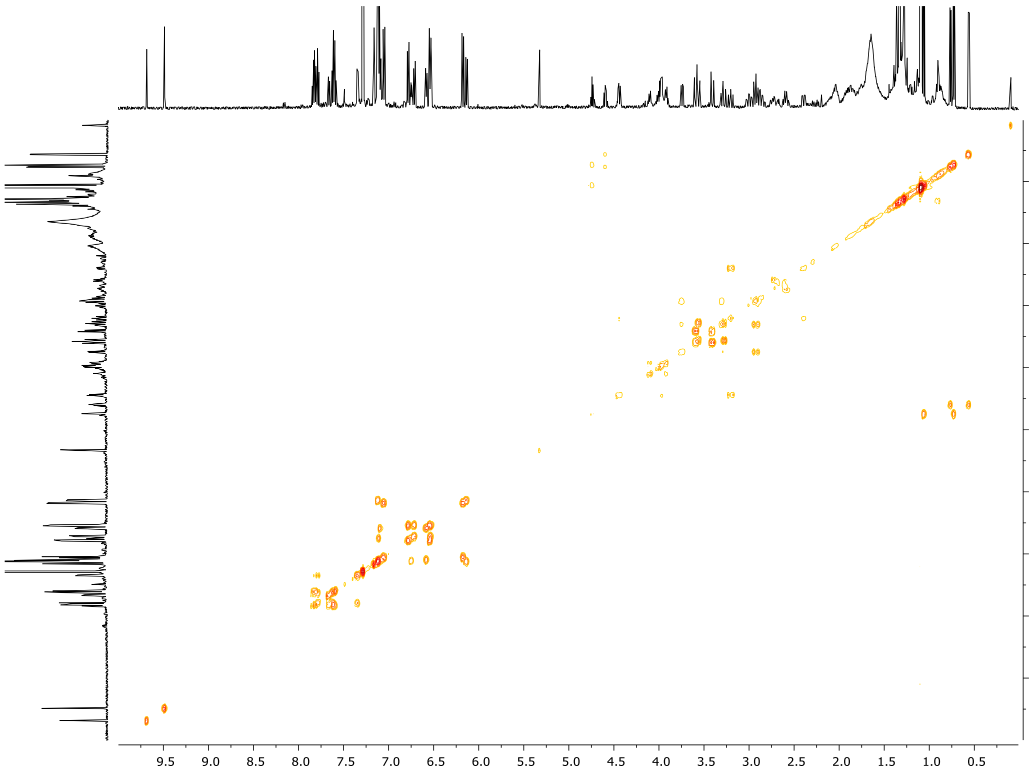

Figure S103. ^{1}H-^{1}H COSY NMR (126 MHz, $CDCl_3$, 298 K) (*S*,R$_{mp}$/*S*$_{mp}$)-**S22**, following purification by chromatography.

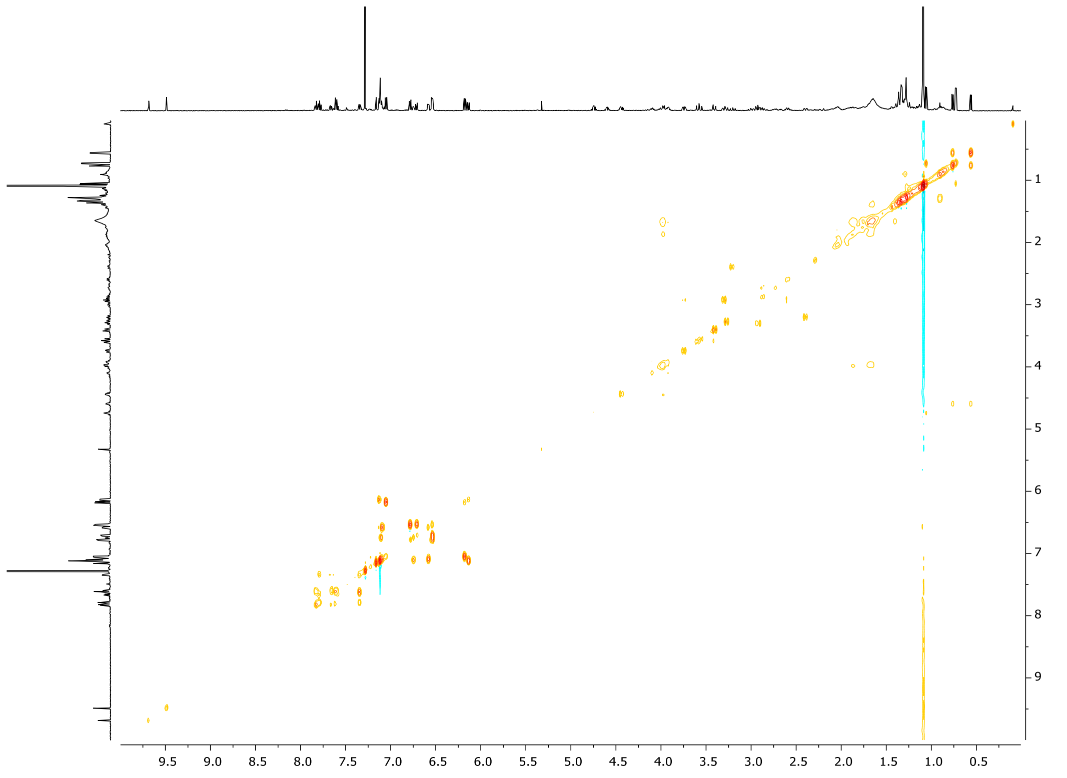

Figure S104. ^{1}H-^{1}H TOCSY NMR (126 MHz, $CDCl_3$, 298 K) (S,R_{mp}/S_{mp})-**S22**, following purification by chromatography.

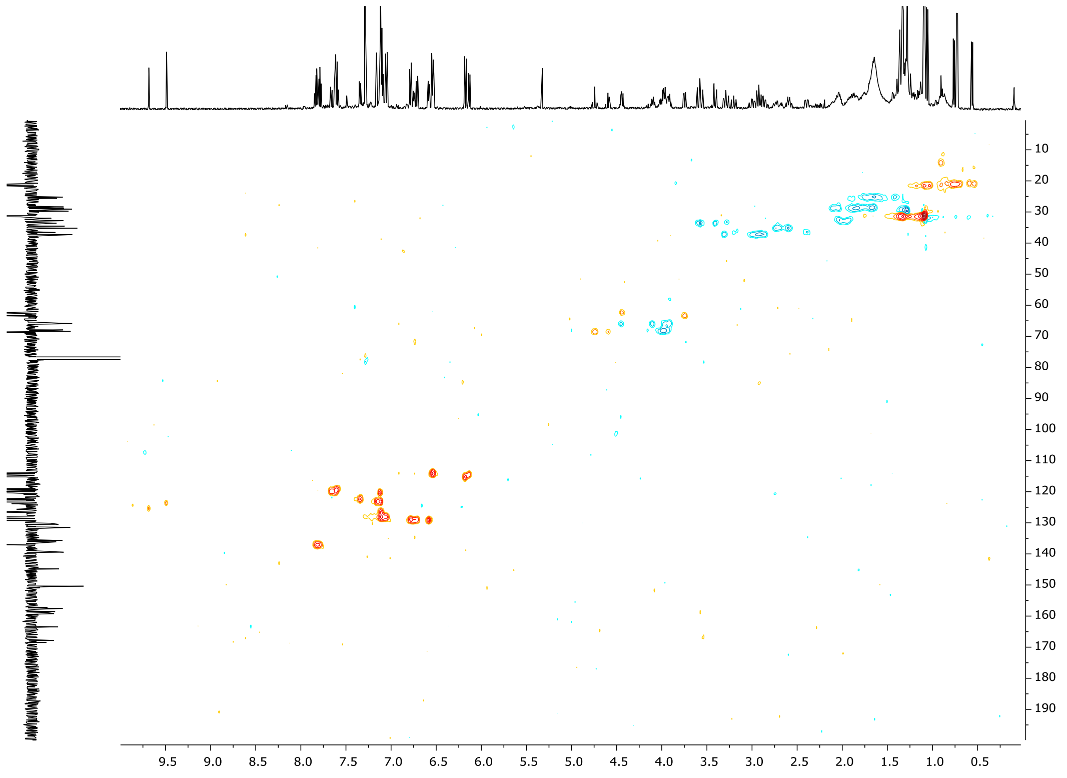

Figure S105. ^{1}H-^{13}C HSQC NMR (126 MHz, $CDCl_3$, 298 K) (S,R_{mp}/S_{mp})-**S22**, following purification by chromatography.

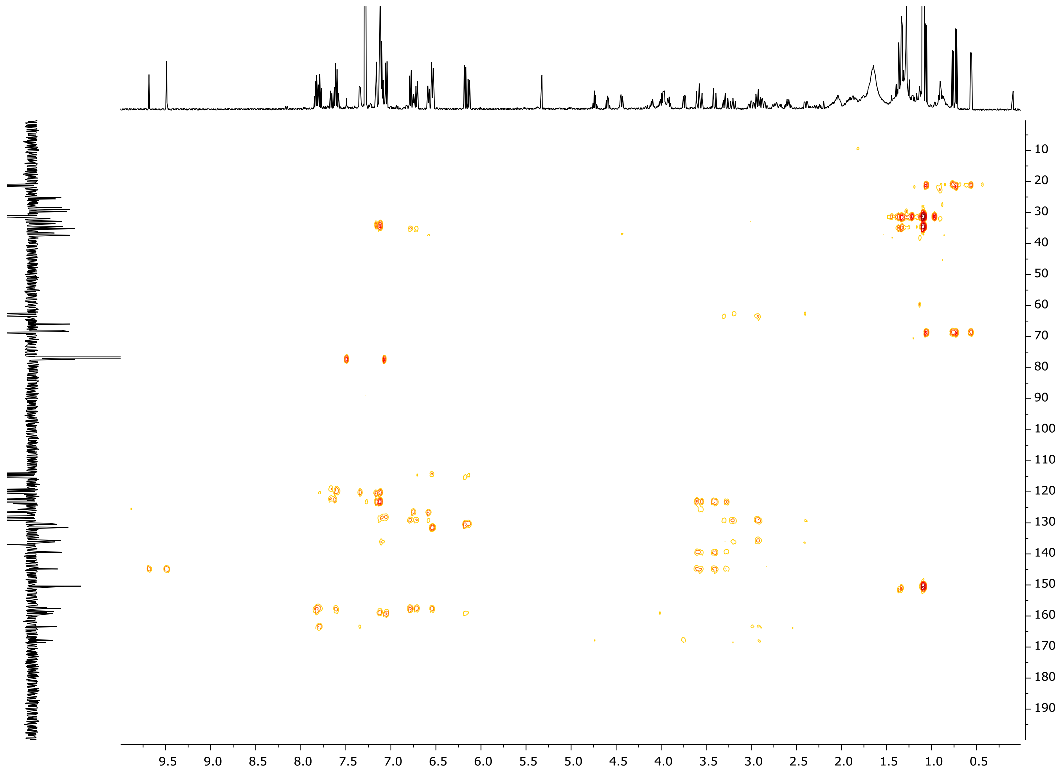

Figure S106. ^1H-^{13}C HMBC NMR (126 MHz, $CDCl_3$, 298 K) (*S*,R_{mp}/S_{mp})-**S22**, following purification by chromatography.

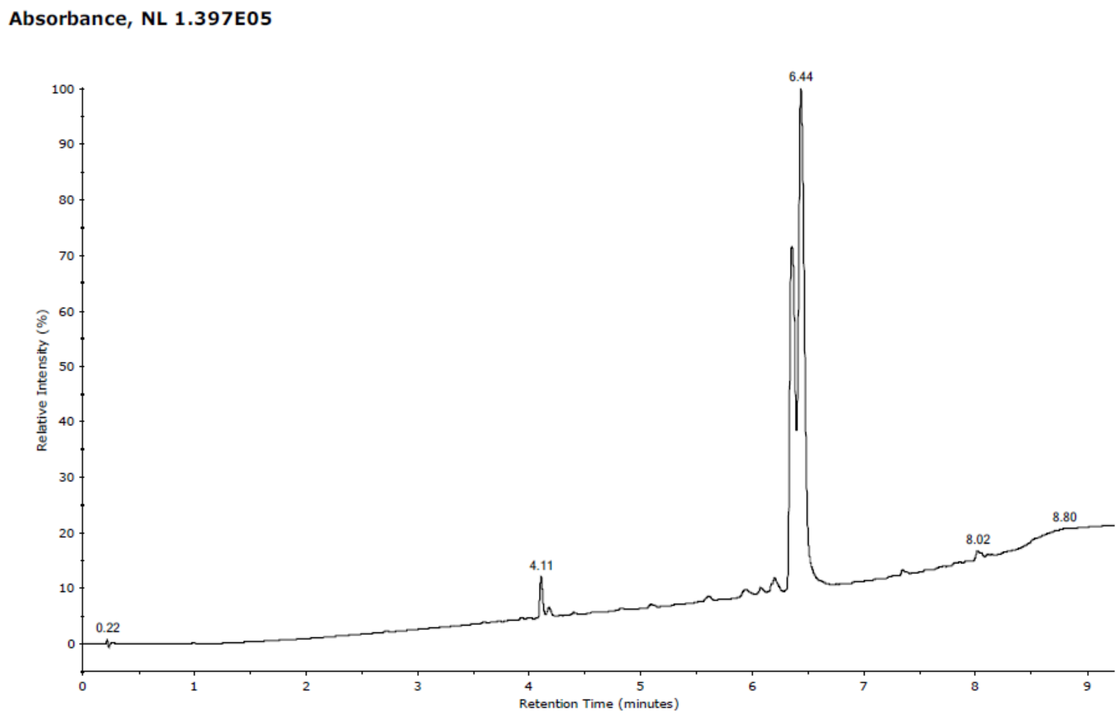

Figure S107. LCMS trace (C_{18} column, gradient 10 minutes (1 : 4 MeCN+0.2% formic acid-H_2O +0.2% formic acid →1 : 0 MeCN-H_2O +0.2% formic acid), UV 254 nm), of (*S*,R_{mp}/S_{mp})-**S22** following purification by chromatography.

Entry 8 – axle (*S*)-S23 and rotaxane (*S*,*R*$_{mp}$/*S*$_{mp}$)-S24 derived from alkyne 2e and azide (*S*)-3e

Axle (*S*)-**S23**

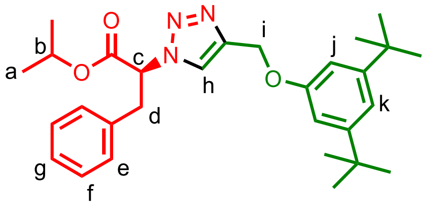

A 10 mL round bottom flask was charged with **2e** (16.9 mg, 0.0692 mmol), (*S*)-**3e** (14.7 mg, 0.0630 mmol), $CuSO_4.5H_2O$ (15.7 mg, 0.0.630 mmol), sodium *L*-ascorbate (14.3 mg, 0.0720 mmol), in DMF (5 mL). The reaction mixture was stirred at rt for 16 h. Saturated EDTA-NH_3 solution (20 mL) was added, and the aqueous layer was extracted with EtOAc (3 × 20 mL). The combined organic extracts were washed with 5% w/v LiCl (5 × 20 mL), brine (20 mL), were dried over $MgSO_4$, filtered, and the solvent removed *in vacuo*. The residue was purified by chromatography (CH_2Cl_2 with 0→10% MeOH), to yield axle (*S*)-**S23** as a yellow solid (20.0 mg, 66%); ^{1}H NMR (400 MHz, $CDCl_3$, 298 K) δ 7.77 (s, 1H, H_h), 7.20-7.14 (m, 3H, H_f and H_g), 7.08-7.01 (m, 3H, H_e and H_k), 7.85 (d, 2H, *J* = 1.8, H_j), 5.54 (dd, 1H, *J* = 7.4, 7.4, H_c), 5.21 (s, 2H, H_i), 5.02 (sept, 1H, *J* = 6.0, H_b), 3.47 (dd, 1H, *J* = 13.6, 7.1, one of H_d), 3.43 (dd, 1H, *J* = 13.6, 7.1, one of H_d), 1.31 (s, 18H, H_l), 1.20 (d, 3H, *J* = 6.0, three of H_a), 1.12 (d, 3H, *J* = 6.0, three of H_a); ^{13}C NMR (101 MHz, $CDCl_3$, 298 K) δ 167.8, 158.0, 152.4, 144.8, 134.8, 129.2, 128.9, 127.6, 122.7, 115.5, 109.3, 70.6, 64.4, 62.2, 39.3, 35.2, 31.6, 21.7, 21.6; LR-ESI-MS (+ve) *m/z* (%) = 478.6 [M+H]$^+$ (100); HR-ESI-MS (+ve) *m/z* = 478.3074 [M+H]$^+$ calc. 478.3064.

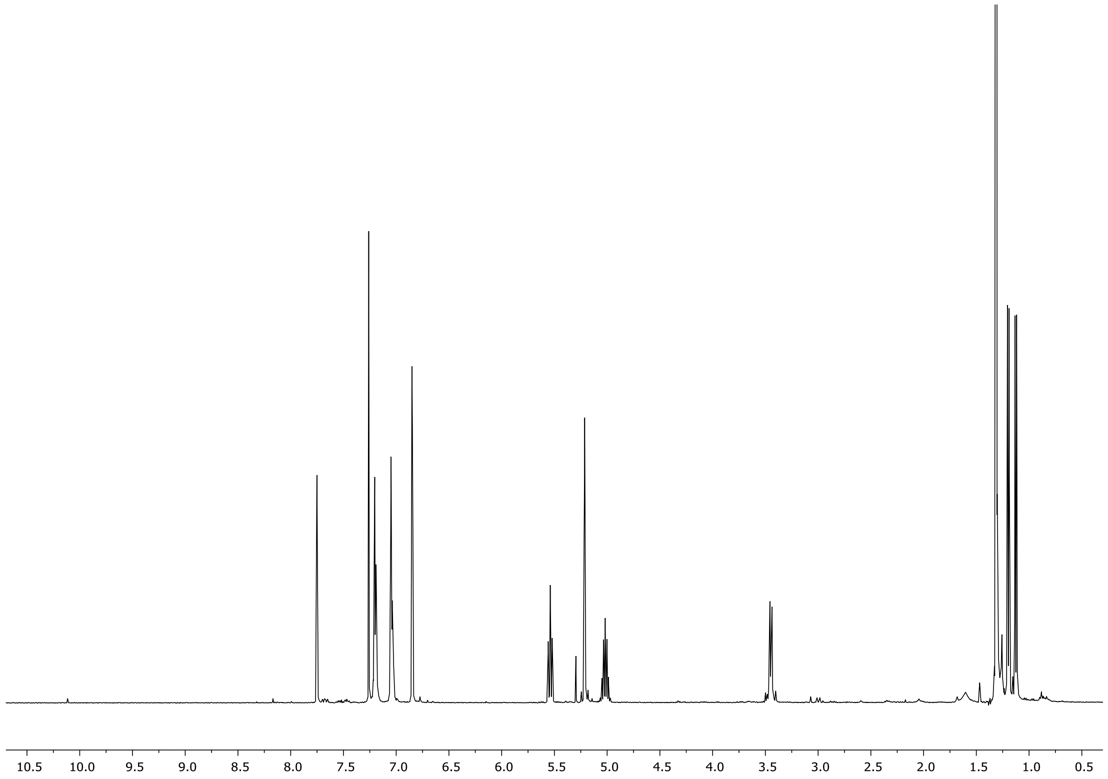

Figure S108. ^{1}H NMR (400 MHz, $CDCl_3$, 298 K) (*S*)-**S23**.

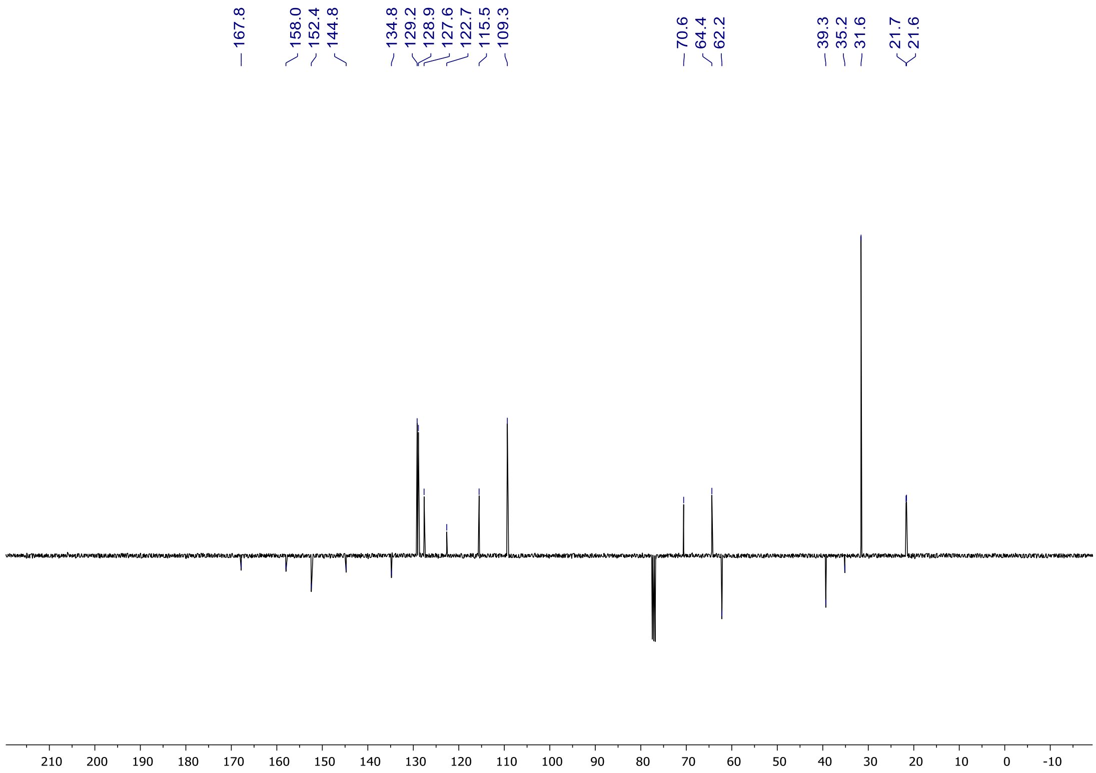

Figure S109. ^{13}C NMR (101 MHz, $CDCl_3$, 298 K) (*S*)-**S23**.

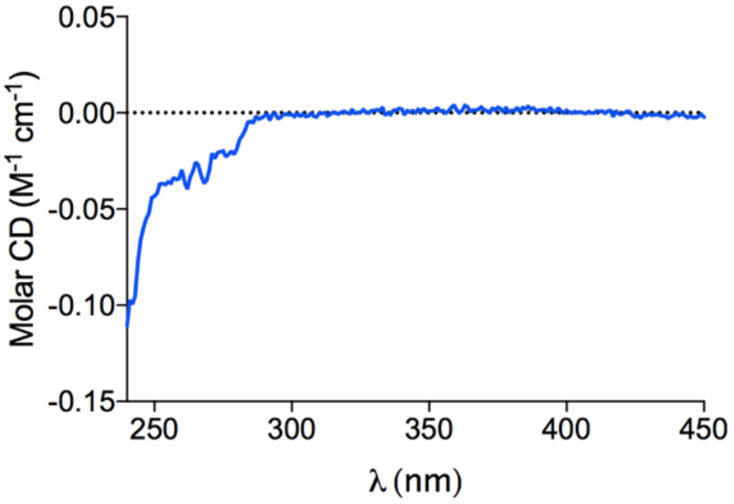

Figure S110. Circular dichroism spectrum of (*S*)-**S23** (0.536 mM in $CHCl_3$).

Rotaxanes (S,R_{mp}/S_{mp})-**S24**

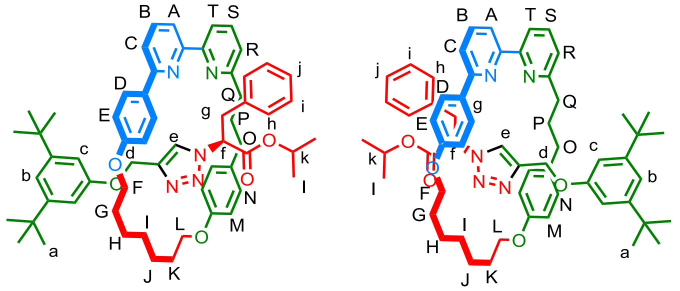

(S,R_{mp})-**S24** (S,S_{mp})-**S24**

A dry sealed vessel was charged with **2e** (19.0 mg, 0.077 mmol), (S)-**3e** (18.2 mg, 0.077 mmol), macrocycle **1** (18.6 mg, 0.039 mmol), $[Cu(MeCN)_4]PF_6$ (13.9 mg, 0.037 mmol), DIPEA (27.1 μL, 0.155 mmol), and anhydrous CH_2Cl_2 (0.95 mL). The reaction mixture was stirred at rt for 16 h, protected by an argon atmosphere. TFA (0.10 mL, 1.305 mmol) was added and the reaction mixture was stirred for an additional 16 h. Saturated EDTA-NH_3 solution (10 mL) was added, and the aqueous layer was extracted with $CHCl_3$ (3 × 30 mL), dried over $MgSO_4$, filtered, and had the solvent removed *in vacuo*. The residue containing rotaxanes (S,R_{mp}/S_{mp})-**S24** (in a 0.25 : 0.75 diastereoisomeric ratio by ^{1}H NMR, **Figure S111**) was purified by chromatography (CH_2Cl_2 with 0→5% EtOH), to yield rotaxanes (S,R_{mp}/S_{mp})-**S24** as a white foam (34.7 mg, 93%, in a 0.30 : 0.70 diastereoisomeric ratio by ^{1}H NMR, **Figure S112**); ^{1}H NMR (400 MHz, $CDCl_3$, 298 K)* δ 9.05 (s, 1H, H_e (*minor*)), 8.68 (s, 1H, H_e (*major*)), 7.83-7.67 (m, 2H, H_S and H_B), 7.60 (dd, 1H, J = 7.5, 2.4, H_C (*major*)), 7.54 (d, 1H, J = 7.8, H_T), 7.52-7.47 (m, 4H, H_A, H_C (*minor*) and H_D (*minor*)), 7.45 (d, 2H, J = 8.7, H_D (*major*)), 7.31-7.23 (m, 1H, H_R), 7.20-7.15 (m, 3H, H_i (*major*) and H_j (*major*)), 7.15-7.11 (m, 3H, H_i (*minor*) and H_j (*minor*)), 6.94 (t, 1H, J = 1.6, H_b (*minor*)), 6.92-6.86 (m, 3H, H_b (*major*) and H_h (*major*)), 6.79 (dd, 2H, J = 6.4, 3.0, H_h (*minor*)), 6.74-6.69 (m, 4H, H_c (*minor*) and H_N), 6.65 (d, 2H, J = 8.8, H_E (*minor*)), 6.62 (d, 2H J = 1.6, H_c (*major*)), 6.59 (d, 2H, J = 8.8, H_E (*major*)), 6.55 (d, 2H, J = 8.5, H_M (*minor*)), 6.48 (d, 2H, J = 8.5, H_M (*major*)), 4.89 (dd, 1H, J = 10.6, 5.7, H_f (*minor*)), 4.84-4.72 (m, 2H, H_f (*major*) and H_k), 4.70 (d, 1H, J = 11.2, one of H_d (*major*)), 4.50 (d, 1H, J = 11.1, one of H_d (*minor*)), 4.37 (dt, 1H, J = 7.2, 3,3, one of H_F (*minor*)), 4.30 (d, 1H, J = 11.1, one of H_d (*minor*)), 4.20 (td, 1H, J = 8.4, 4.5, one of H_F (*major*)), 4.15-3.96 (m, 3H, one of H_d (*major*) and H_L), 3.94-3.85 (m, 1H, one of H_F), 3.28 (dd, 1H, J = 13.7, 10.6, one of H_g (*minor*)), 3.10-3.04 (m, 1H one of H_g (*major*)), 2.87-2.71 (m, 3H, one of H_g (*minor*) and H_Q), 2.69-2.52 (m, 2H, H_O), 2.09-1.85 (m, 3H, H_P and one of H_G), 1.74-1.25 (m, 9H, one of H_G, H_K, H_H, H_I and H_J), 1.25 (s, 18H, H_a (*minor*)), 1.21 (s, 18H, H_a (*major*)), 1.07 (d, 3H, J = 6.3, three of H_l (*major*)), 0.91 (d, 3H, J = 6.3, three of H_l (*minor*)), 0.87 (d, 3H, J = 6.3, three of H_l (*major*)), 0.82 (d, 3H, J = 6.2, three of H_l (*minor*)); ^{13}C NMR (101 MHz, $CDCl_3$, 298 K) δ 168.1, 167.8, 163.5, 163.5, 158.9, 158.9, 158.7, 158.7, 158.7, 158.6, 158.0, 157.9, 157.7, 157.4, 157.4, 151.7, 151.4, 144.2, 143.1, 137.2, 137.1, 136.9, 136.8, 136.1, 135.7, 132.7, 132.7, 131.8, 131.3, 129.3, 129.3, 129.3, 129.2, 128.9, 128.8, 128.5, 128.4, 127.1, 126.9, 124.7, 122.1, 122.0, 120.1, 120.0, 120.0, 119.5, 119.4, 115.3, 115.2, 114.6, 114.2, 114.0, 109.3, 109.2, 69.4, 69.3, 67.9, 67.8, 66.5, 66.2, 63.7, 63.0, 61.5, 61.4, 38.0, 37.6, 37.6, 37.6, 35.4, 35.0, 35.0, 31.9, 31.9, 31.6, 31.6, 29.85, 29.2, 28.7, 28.6, 28.6, 28.4, 25.9, 25.8, 21.6, 21.4, 21.4. HR-ESI-MS (+ve) m/z = 956.5611 $[M+H]^+$ calc. 956.5689.

*As stereochemistry could not be unambiguously assigned, the signals are simply designated (*major*) or (*minor*). Proton counts are provided for each signal and represent the expected integration of that environment. Where the major and minor diastereoisomer signals are coincident, no (*major*)/(*minor*) label is provided and the proton count indicated refers the expected integration of that signal in each of the stereoisomers that contributes to the multiplet.

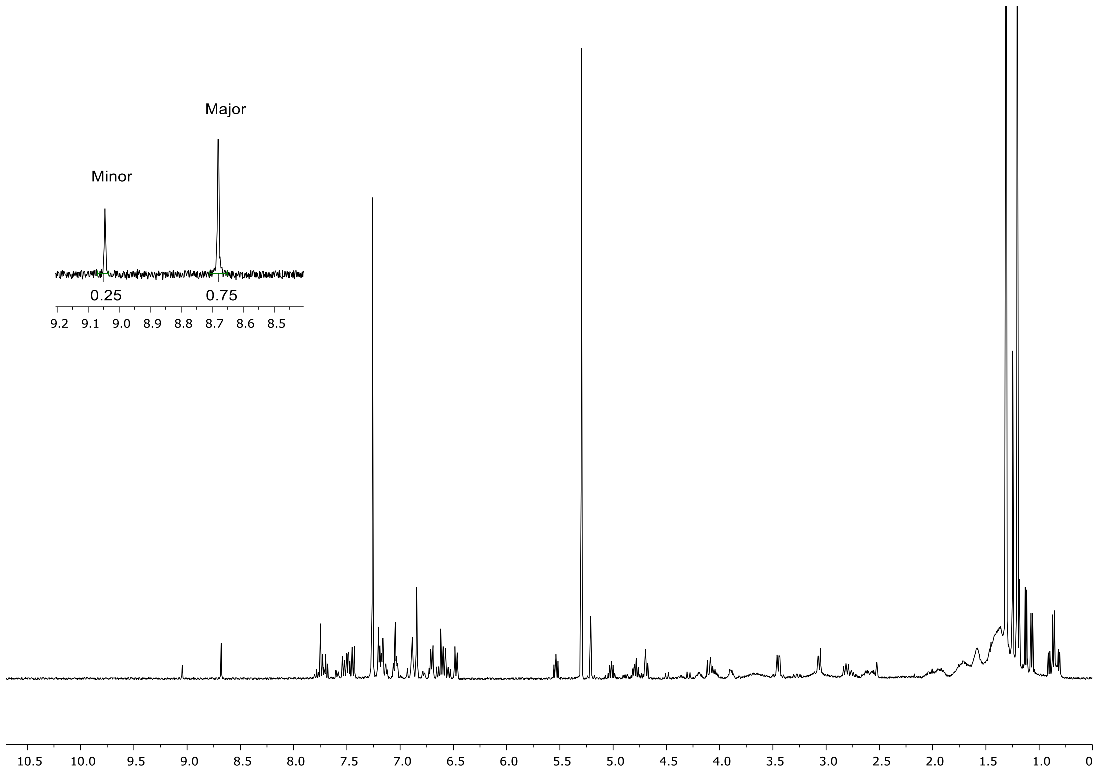

Figure S111. ^{1}H NMR (400 MHz, $CDCl_3$, 298 K) (S,R_{mp}/S_{mp})-**S24**, prior to purification by chromatography.

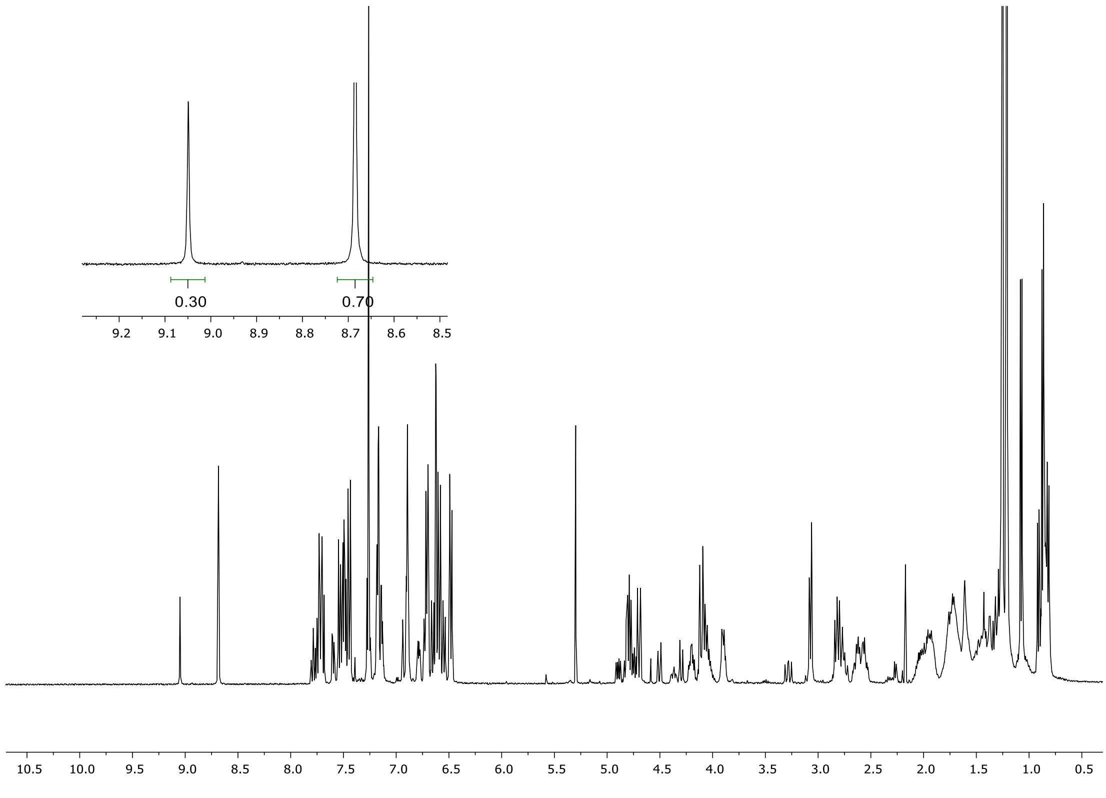

Figure S112. ^{1}H NMR (400 MHz, $CDCl_3$, 298 K) (S,R_{mp}/S_{mp})-**S24**, following purification by chromatography.

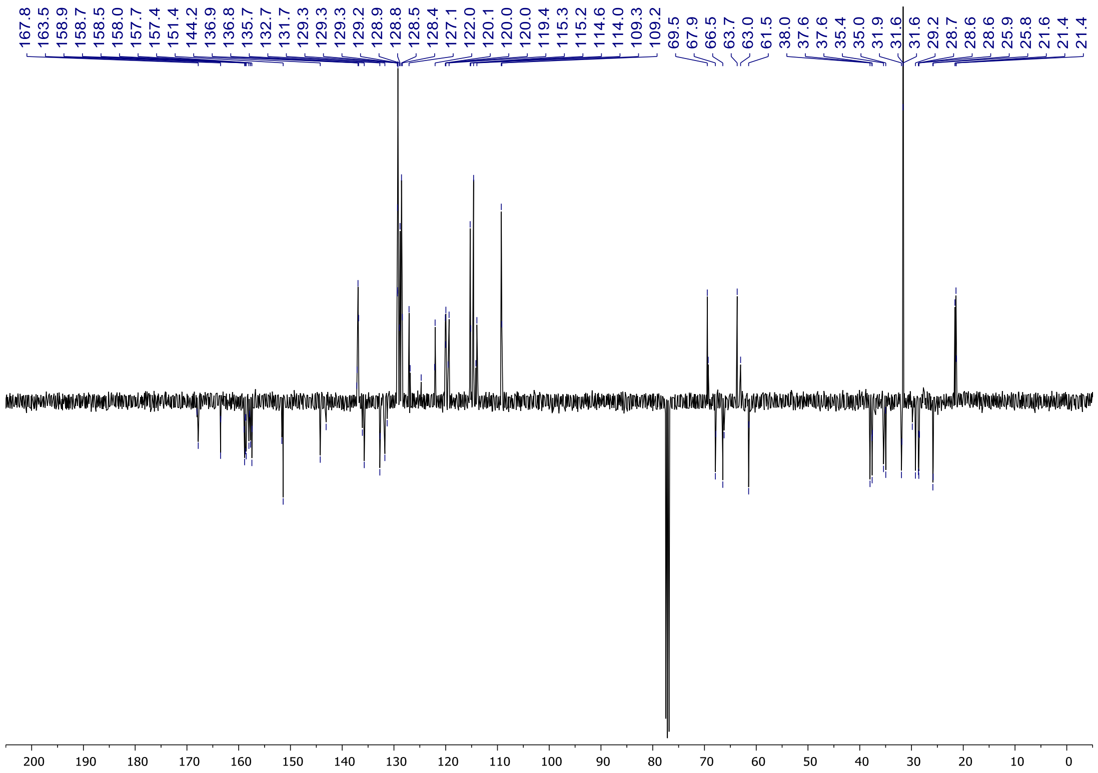

Figure S113. ^{13}C NMR (101 MHz, $CDCl_3$, 298 K) (*S*,R_{mp}/S_{mp})-**S24**, following purification by chromatography.

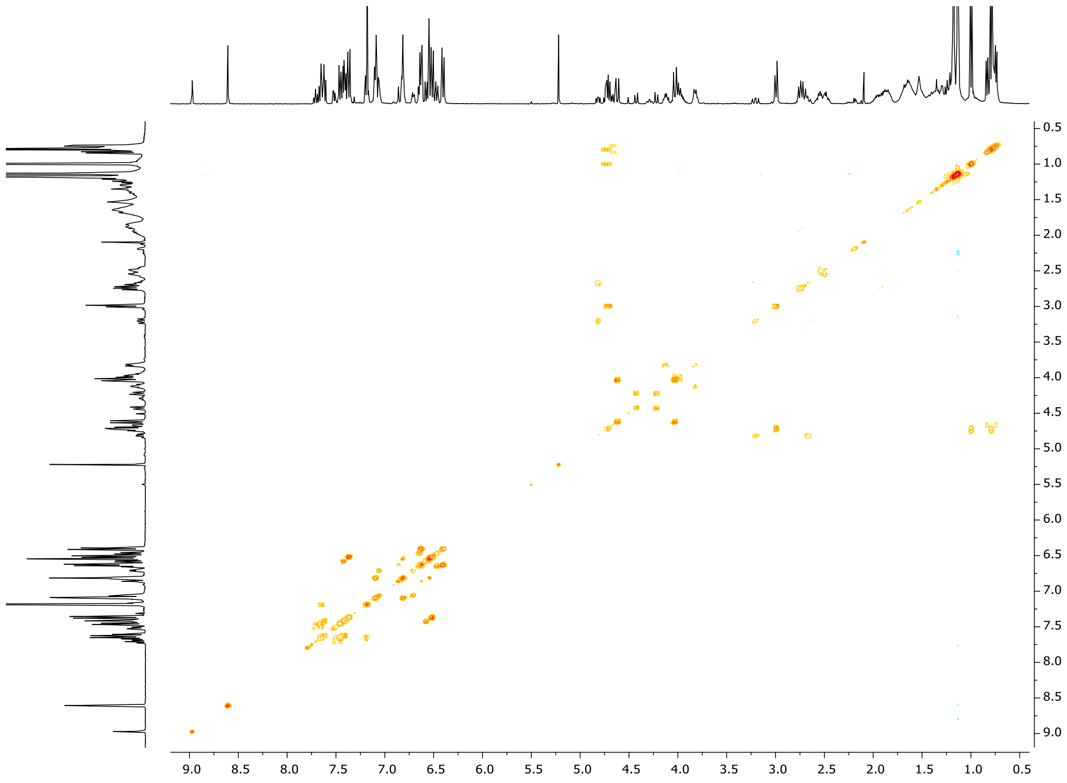

Figure S114. ^{1}H-^{1}H COSY NMR (400 MHz, $CDCl_3$, 298 K) (*S*,R_{mp}/S_{mp})-**S24**, following purification by chromatography.

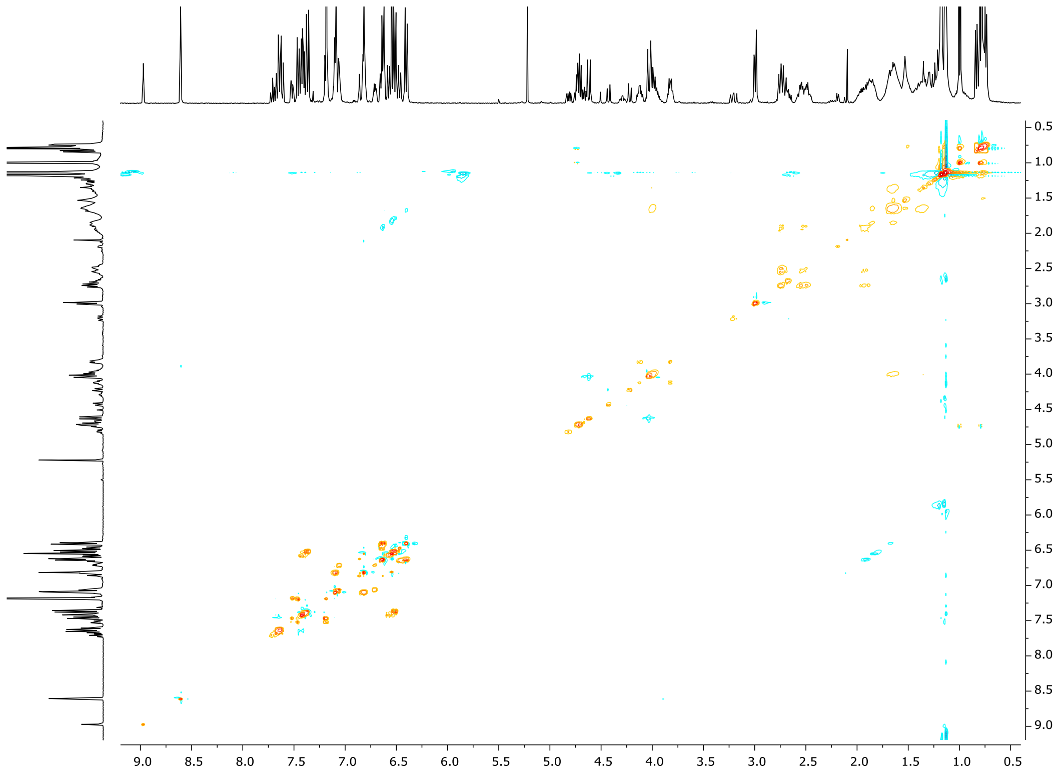

Figure S115. ^{1}H-^{1}H TOCSY NMR (400 MHz, $CDCl_3$, 298 K) (S,R_{mp}/S_{mp})-**S24**, following purification by chromatography.

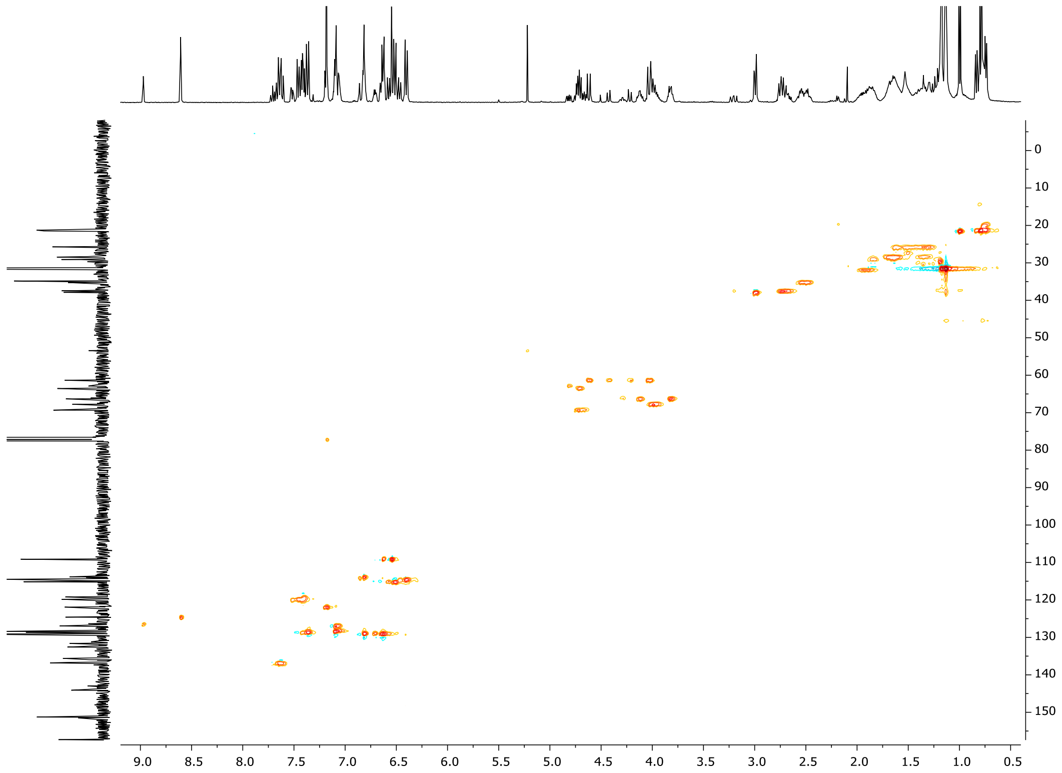

Figure S116. ^{1}H-^{13}C HSQC NMR (101 MHz, $CDCl_3$, 298 K) (S,R_{mp}/S_{mp})-**S24**, following purification by chromatography.

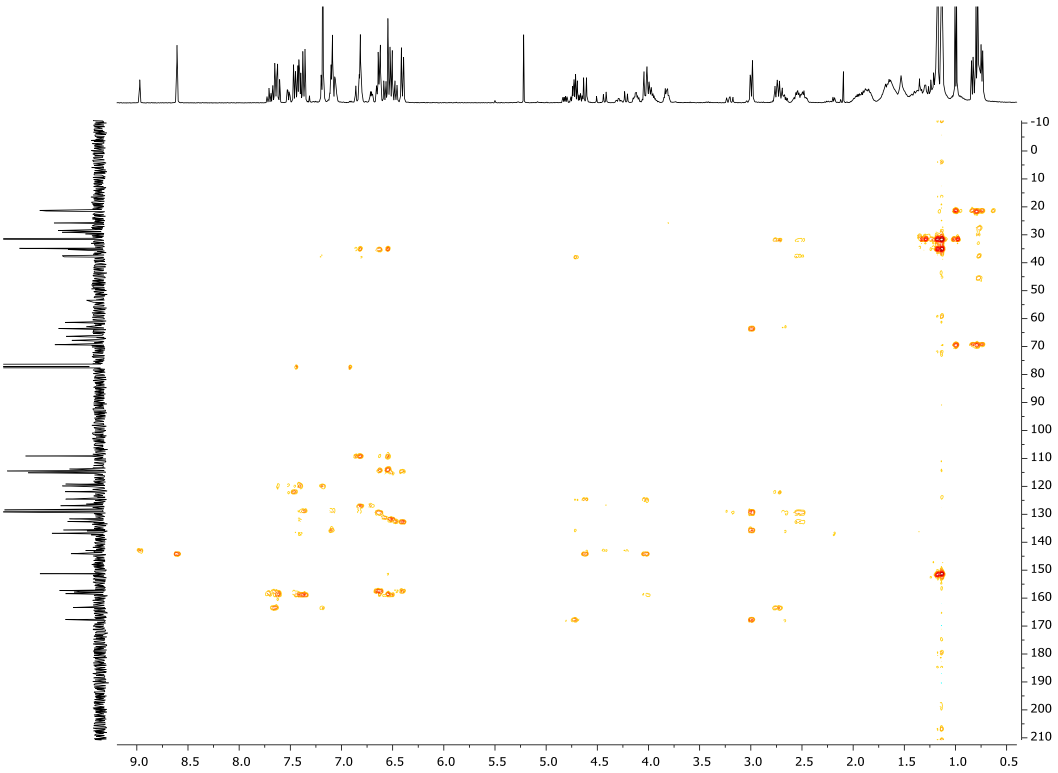

Figure S117. ^{1}H-^{13}C HMBC NMR (101 MHz, $CDCl_3$, 298 K) (S,R_{mp}/S_{mp})-**S24**, following purification by chromatography.

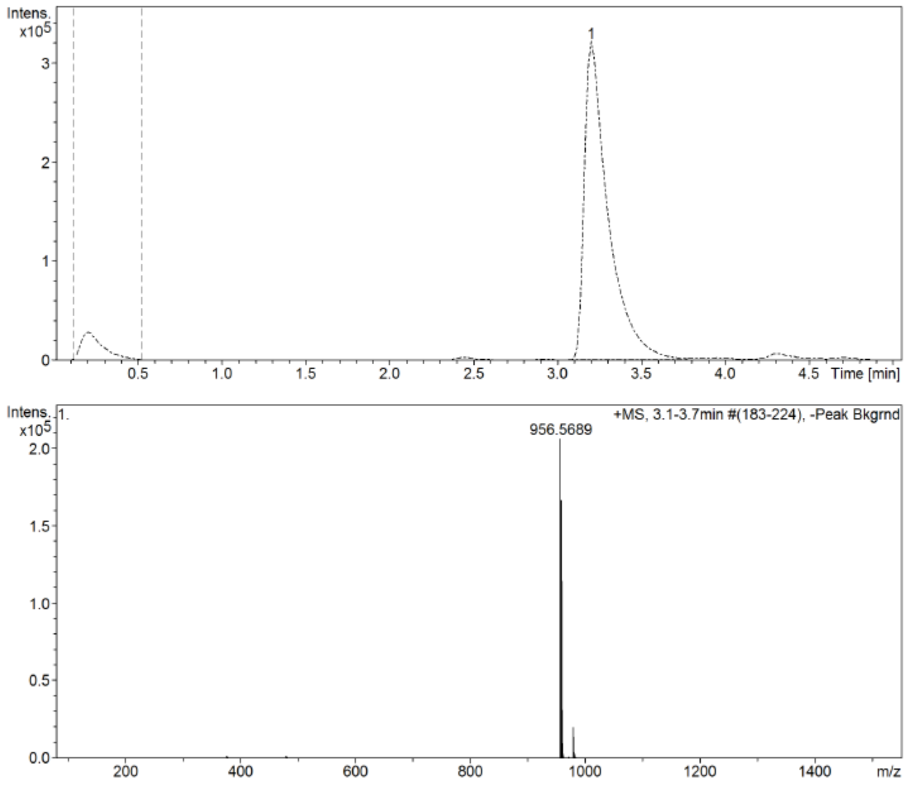

Figure S118. LCMS trace (C_{18} column, gradient 5 minutes (1 : 4 MeCN+0.2% formic acid-H_2O +0.2% formic acid →1 : 0 MeCN-H_2O +0.2% formic acid), UV 254 nm), of (S,R_{mp}/S_{mp})-**S24** following purification by chromatography (top), and HR-ESI-MS spectrum of (S,R_{mp}/S_{mp})-**S24** (bottom).

Entry 9 – axle (*S*)-S25 and rotaxane (*S*,*R*$_{mp}$/*S*$_{mp}$)-S26 derived from alkyne 2f and azide (*S*)-3e

Axle (*S*)-**S25**

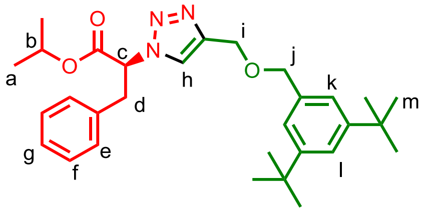

A 10 mL round bottomed flask was charged with **2f** (75.0 mg, 0.264 mmol), (*S*)-**3e** (28.1 mg, 0.120 mmol), $CuSO_4.5H_2O$ (29.29 mg, 0.120 mmol), sodium *L*-ascorbate (31.1 mg, 0.157 mmol), and DMF (3 mL). The reaction mixture was stirred at rt for 16 h. Saturated EDTA-NH_3 solution (20 mL) was added, and the aqueous layer was extracted with EtOAc (3 × 20 mL). The combined organic extracts were washed with 5% w/v LiCl (5 × 20 mL), brine (20 mL), were dried over $MgSO_4$, filtered, and had the solvent removed *in vacuo*. The residue was purified by chromatography (CH_2Cl_2 with 0→10% EtOH), to yield axle (*S*)-**S25** as a yellow oil (16.7 mg, 28%); ^{1}H NMR (400 MHz, $CDCl_3$, 298 K) δ 7.70 (s, 1H, H_h), 7.36 (t, 1H, *J* = 1.8, H_l), 7.26-7.20 (m, 3H, H_f and H_g), 7.18 (d, 2H, *J* = 1.8, H_k), 7.05-7.01 (m, 2H, H_e), 5.54 (dd, 1H, *J* = 7.4, 8.1, H_c), 5.02 (sept, 1H, *J* = 6.2, H_b), 4.69 (s, 2H, H_i), 4.53 (s, 2H, H_j), 4.09 (d, 1H, *J* = 16, one of H_i), 3.49 (dd, 1H, *J* = 14.0, 7.4, one of H_d), 3.42 (dd, 1H, *J* = 14.0, 8.1, one of H_d), 1.33 (s, 18H, H_m), 1.20 (d, 3H, *J* = 6.2, three of H_a), 1.13 (d, 3H, *J* = 6.2, three of H_a); ^{13}C NMR (101 MHz, $CDCl_3$, 298 K) δ 167.9, 151.0, 145.5, 137.0, 134.9, 129.2, 128.9, 127.6, 122.6, 122.4, 122.0, 73.2, 70.5, 64.3, 63.8, 39.3, 35.0, 31.6, 21.7, 21.6; LR-MS-ESI (ESI+) *m/z* (%) = 492.6 $[M+H]^+$ (100); HR-ESI-MS (+ve) *m/z* = 492.3230 $[M+H]^+$ calc. 492.3221.

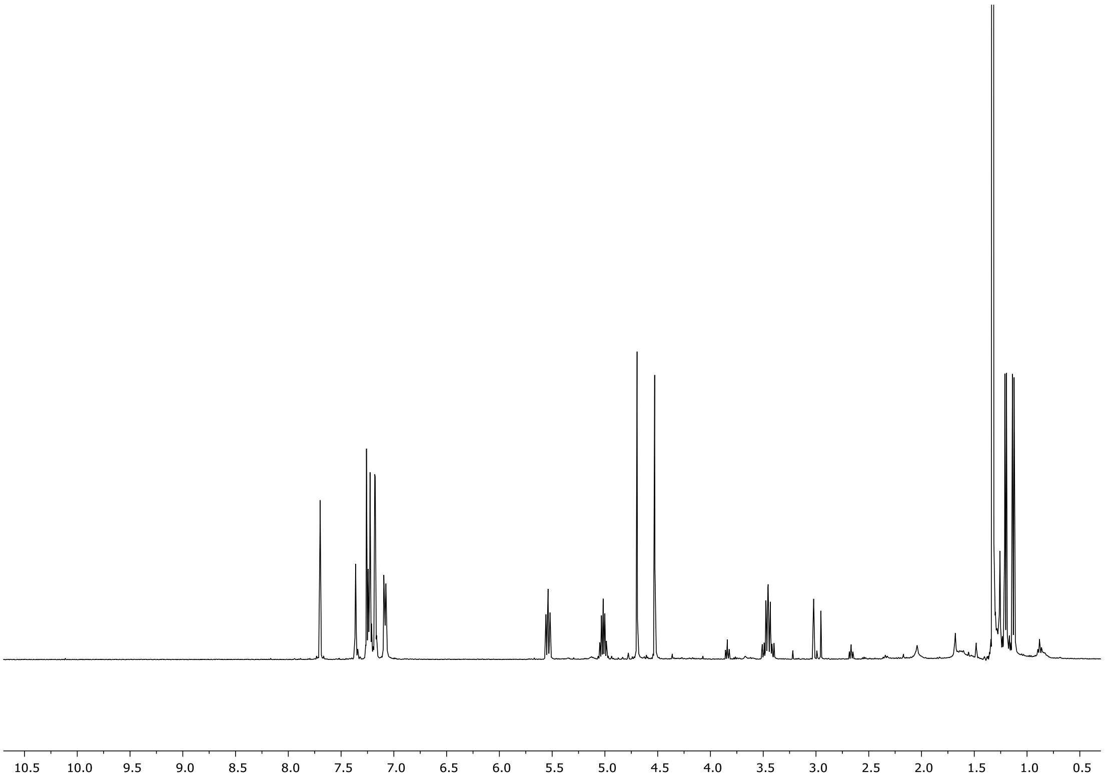

Figure S119. ^{1}H NMR (400 MHz, $CDCl_3$, 298 K) (*S*)-**S25**.

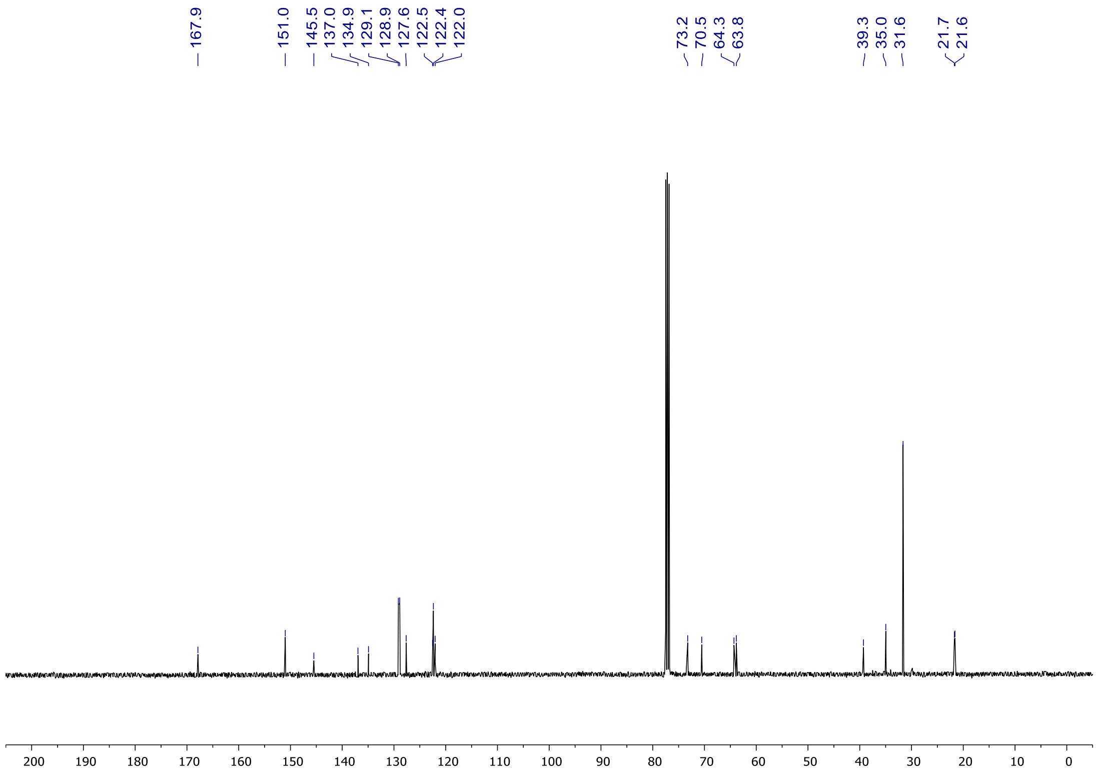

Figure S120. ^{13}C NMR (101 MHz, $CDCl_3$, 298 K) (*S*)-**S25**.

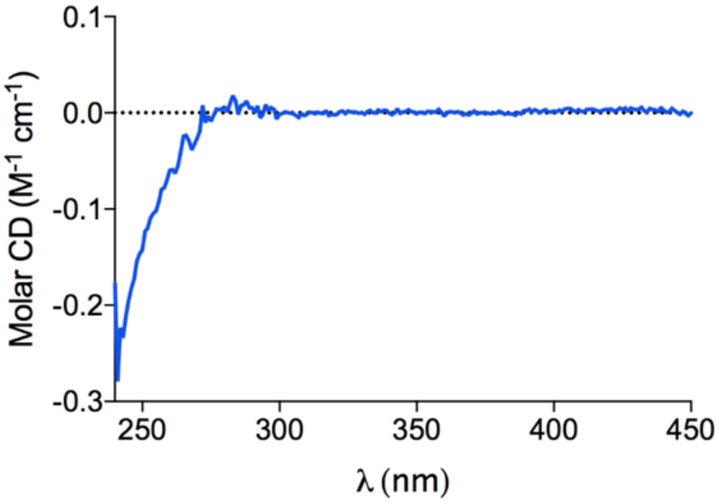

Figure S121. Circular dichroism spectrum of (*S*)-**S25** (0.342 mM in $CHCl_3$).

Rotaxane (S,R_{mp}/S_{mp})-**S26**

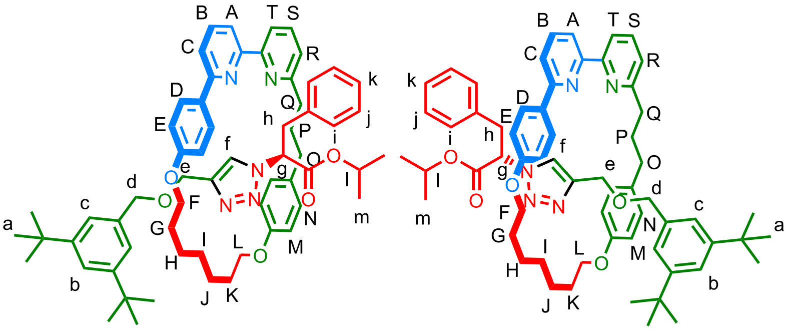

A dry sealed vessel was charged with **2f** (32.0 mg, 0.124 mmol), (S)-**3e** (29.0 mg, 0.124 mmol), macrocycle **1** (29.0 mg, 0.062 mmol), $[Cu(MeCN)_4]PF_6$ (22.0 mg, 0.059 mmol), DIPEA (43 µL, 0.248 mmol), and anhydrous CH_2Cl_2 (1.5 mL). The reaction mixture was stirred at rt for 16 h, protected by an argon atmosphere. TFA (0.15 mL, 1.95 mmol) was added and the reaction mixture was stirred for an additional 16 h. Saturated EDTA-NH_3 (10 mL) was added, and the aqueous layer was extracted with $CHCl_3$ (3 × 30 mL), dried over $MgSO_4$, filtered, and had the solvent removed *in vacuo*. The residue containing (S,R_{mp}/S_{mp})-**S26** (in a 0.28 : 0.72 diastereoisomeric ratio by ^{1}H NMR, **Figure S122**) was purified by chromatography (CH_2Cl_2 with 0→3% EtOH), to yield rotaxanes (S,R_{mp}/S_{mp})-**S26** as a white foam (38.2 mg, 64%, 0.29 : 0.71 diastereomeric ratio **Figure S123**, with 7% of free thread **S25**);^{1}H NMR (400 MHz, $CDCl_3$, 298 K)* δ 9.20 (s, 1H, H_f (*minor*)), 8.94 (s, 1H, H_f (*major*)), 7.87-7.72 (m, 2H, H_B and H_S) 7.70-7.53 (m, 4H, H_C, H_R, H_E), 7.35-7.22 (m, 3H, H_b, H_A, H_T), 7.19-7.07 (m, 5H, H_j, H_k and H_c), 6.87-6.80 (m, 2H, H_N or H_M), 6.80-6.73 (m, 2H, H_i), 6.72-6.66 (m, 2H, H_N or H_M), 6.67-6.59 (m, 2H, H_D), 4.82-4.64 (m, 2H, H_l and one of H_h (*minor*)), 4.56-4.48 (m, 1H, one of H_e), 4.38-4.26 (m, 3H, one of H_e (*minor*) and H_F), 4.21 (d, 1H, J = 11.0, one of H_e (*major*)), 4.16 (d, 1H, J = 12.0, one of H_d (*major*)), 4.10 (d, 1H, J = 11.7, one of H_d (*minor*)), 4.05-4.00 (m, 1H, one of H_d), 4.00 – 3.90 (m, 2H, H_L), 3.90-3.82 (m, 1H, H_h (*major*)), 3.24 (dd, 1H, J = 13.7, 10.9, H_g (*minor*)), 3.12-2.91 (m, 1H, H_g (*major*)), 2.90-2.75 (m, 2H, H_Q), 2.70-2.55 (m, 3H, H_O and one of H_h (*minor*)), 2.12-1.84 (m, 4H, H_P, H_G), 1.83-1.46 (m, 8H, H_H, H_I, H_J and H_K), 1.21 (s, 18H, H_a), 1.06 (d, 3H, J = 6.3, three of H_m (*major*)), 0.88 (d, 3H, J = 6.3, three of H_m (*minor*)), 0.81 (d, 3H, J = 6.2, three of H_m (*major*)), 0.78 (d, 3H, J = 6.3, three of H_m (*minor*)). ^{13}C NMR (101 MHz, $CDCl_3$, 298 K) δ 168.0, 167.8, 163.5, 163.4, 159.0, 158.9, 158.6, 158.4, 157.8, 157.6, 157.4, 150.4, 150.3, 144.1, 143.4, 138.0, 137.9, 137.3, 137.2, 137.0, 136.2, 135.7, 132.4, 132.4, 131.0, 130.7, 129.5, 129.4, 129.3, 129.2, 129.2, 129.1, 128.9, 128.8, 128.7, 128.6, 128.4, 128.2, 127.7, 126.9, 126.7, 125.4, 122.6, 122.2, 122.1, 121.5, 121.4, 120.2, 120.2, 120.1, 120.0, 119.4, 119.2, 115.5, 115.2, 114.7, 114.7, 73.8, 73.5, 69.2, 69.1, 67.9, 67.7, 66.6, 66.3, 64.4, 64.3, 63.6, 62.8, 58.6, 56.9, 37.9, 37.4, 35.4, 35.4, 34.8, 31.8, 31.7, 31.5, 31.5, 29.8, 29.2, 29.1, 28.5, 28.5, 28.4, 28.3, 25.8, 25.7, 25.7, 25.5, 21.6, 21.3, 21.3, 21.3, 18.6; HR-ESI-MS (+ve) m/z = 970.5768 $[M+H]^+$ calc. 970.5861.

*As stereochemistry could not be unambiguously assigned, the signals are simply designated (*major*) or (*minor*). Proton counts are provided for each signal and represent the expected integration of that environment. Where the major and minor diastereoisomer signals are coincident, no (*major*)/(*minor*) label is provided and the proton count indicated refers the expected integration of that signal in each of the stereoisomers that contributes to the multiplet.

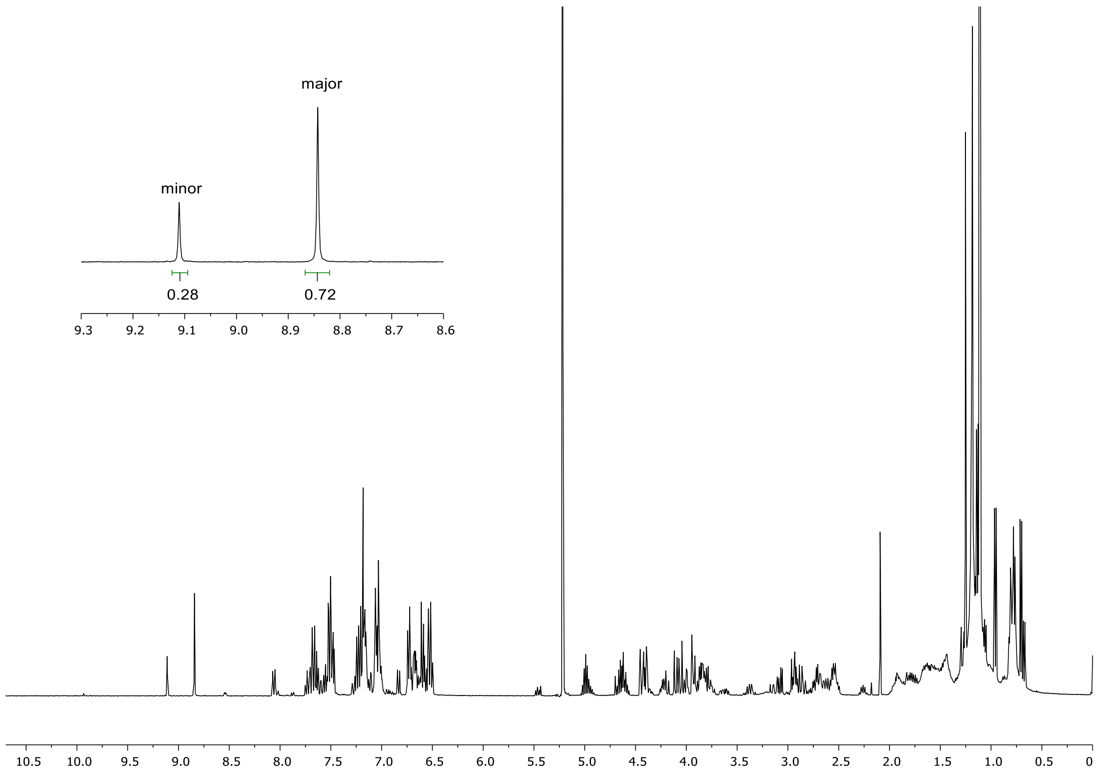

Figure S122. ^{1}H NMR (400 MHz, $CDCl_3$, 298 K) (*S*,R_{mp}/S_{mp})-**S26**, prior to purification by chromatography.

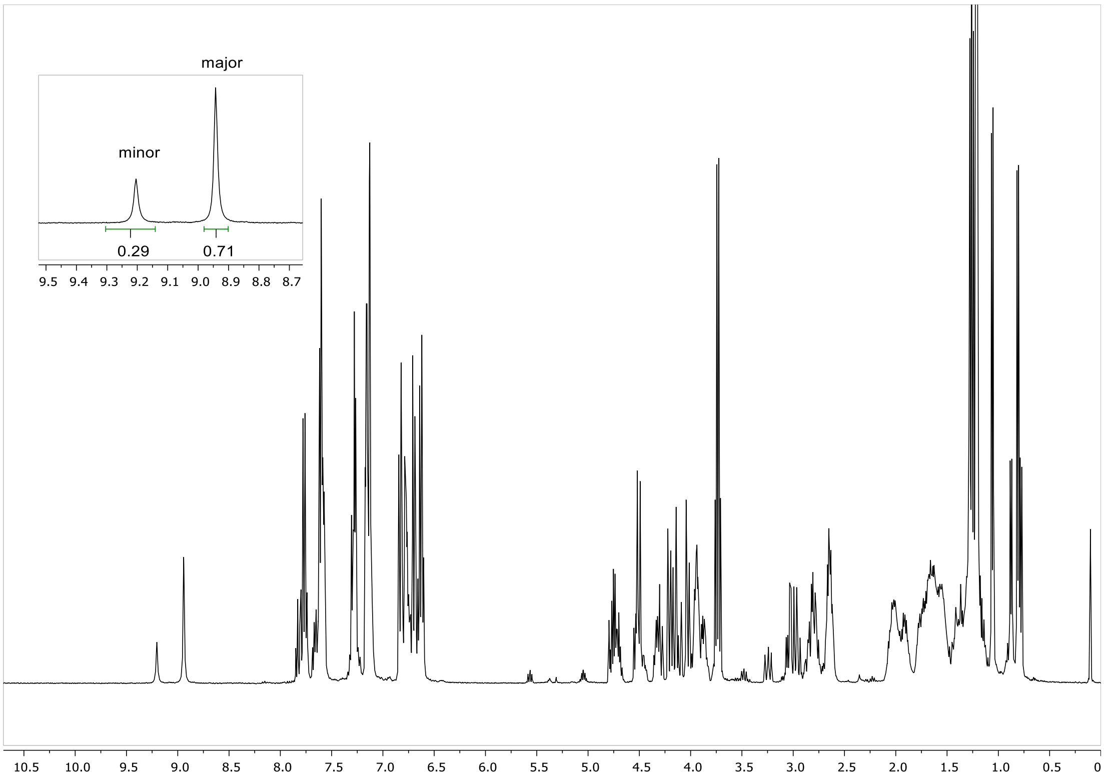

Figure S123. ^{1}H NMR (400 MHz, $CDCl_3$, 298 K) (*S*,R_{mp}/S_{mp})-**S26**, following purification by chromatography.

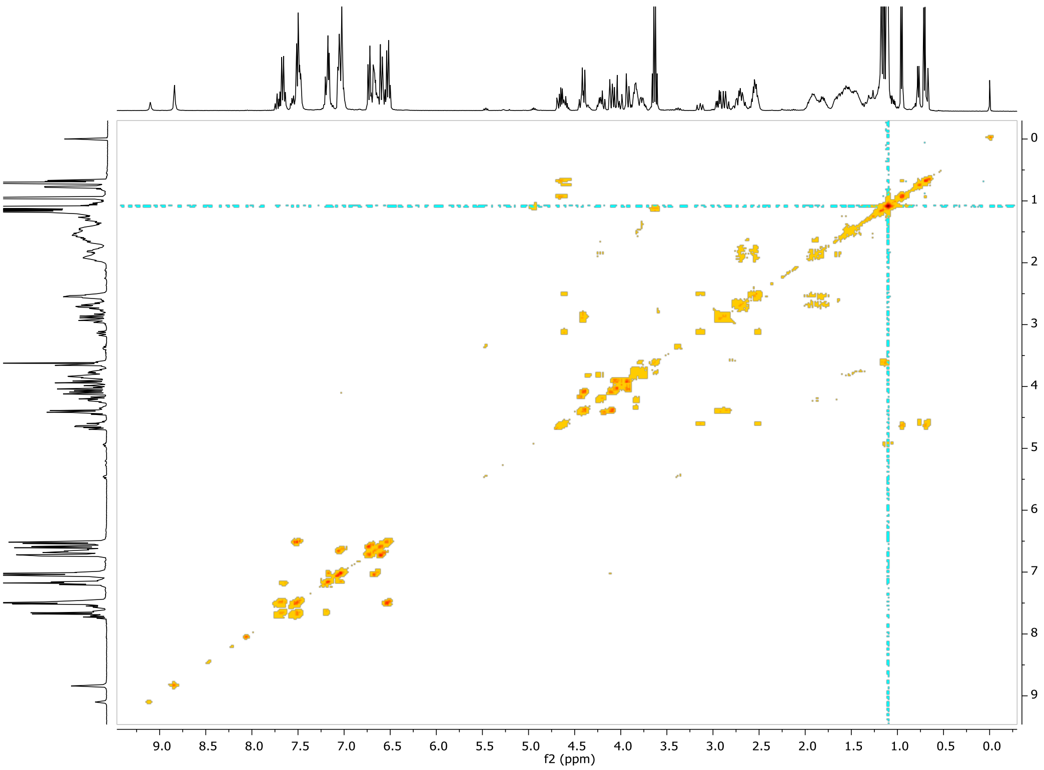

Figure S124. ^1H-^1H COSY NMR (400 MHz, $CDCl_3$, 298 K) (S,R_{mp}/S_{mp})-**S26**, following purification by chromatography.

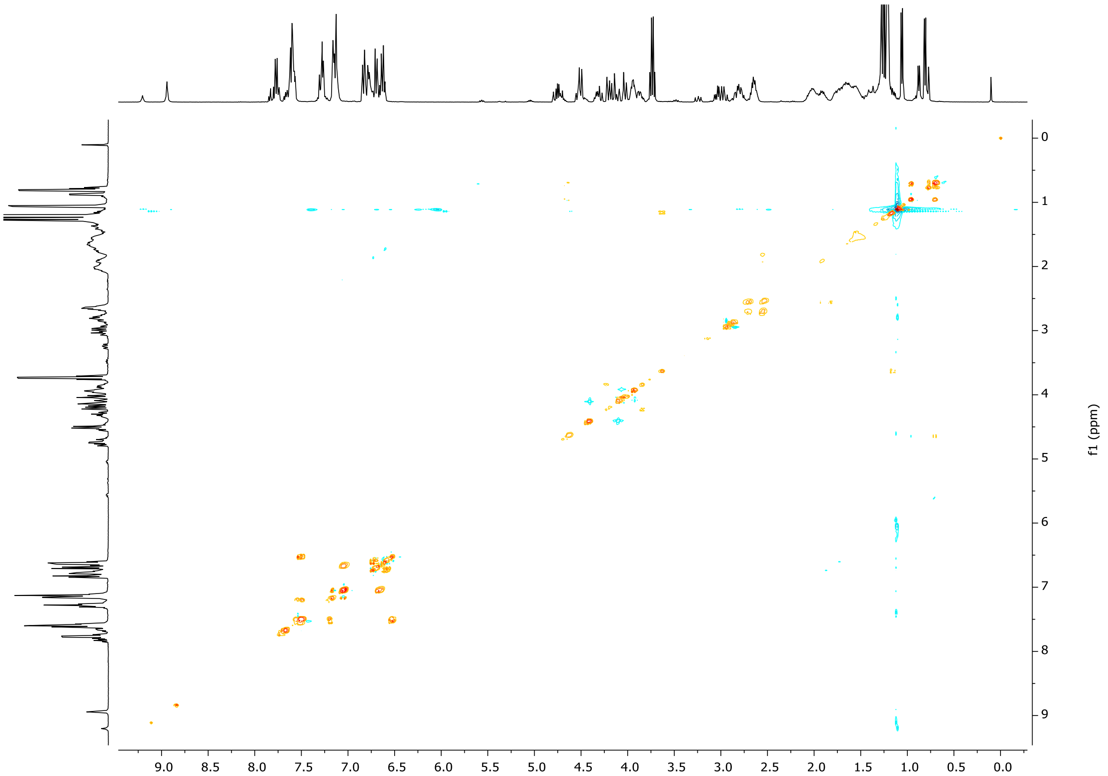

Figure S125. ^1H-^1H TOCSY NMR (400 MHz, $CDCl_3$, 298 K) (S,R_{mp}/S_{mp})-**S26**, following purification by chromatography.

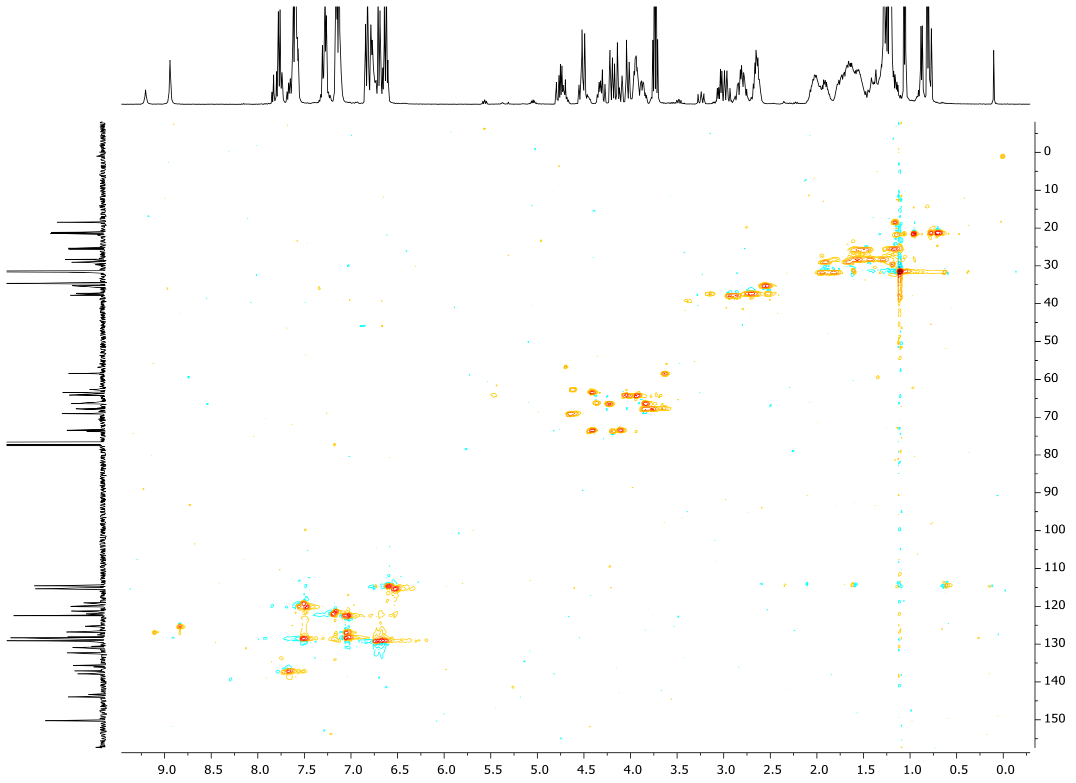

Figure S126. ^{1}H-^{13}C HSQC NMR (101 MHz, $CDCl_3$, 298 K) (S,R_{mp}/S_{mp})-**S26**, following purification by chromatography.

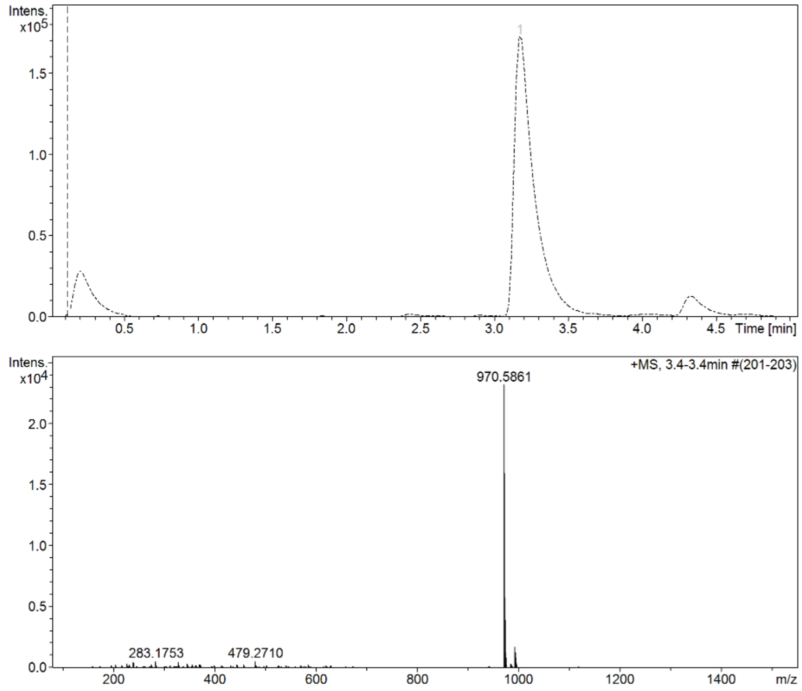

Figure S127. LCMS trace (C_{18} column, gradient 10 minutes (1 : 4 MeCN+0.2% formic acid-H_2O +0.2% formic acid →1 : 0 MeCN-H_2O +0.2% formic acid), UV 254 nm), of (S,R_{mp}/S_{mp})-**S26** following purification by chromatography (top), and HR-ESI-MS spectrum of (S,R_{mp}/S_{mp})-**S26** (bottom).

Entry 10 – axle (*S*)-S27 and rotaxanes (*S*,*R*$_{mp}$/*S*$_{mp}$)-S28 derived from alkyne 2g and azide (*S*)-3e

Axle (*S*)-**S27**

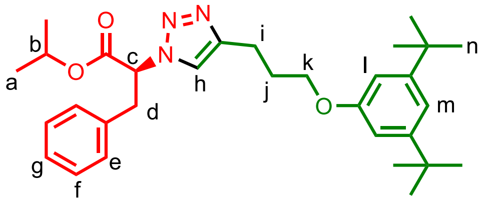

A 5 mL round bottomed flask was charged with **2g** (35.4 mg, 0.130 mmol), (*S*)-**3e** (26.4 mg, 0.113 mmol), $CuSO_4.5H_2O$ (28.1 mg, 0.113 mmol), sodium *L*-ascorbate (30.3 mg, 0.153 mmol), and DMF (3 mL). The reaction mixture was stirred at rt for 16 h. Saturated EDTA-NH_3 solution (20 mL) was added, and the aqueous layer was extracted with EtOAc (3 × 20 mL). The combined organic extracts were washed with 5% w/v LiCl (5 × 20 mL), brine (20 mL), were dried over $MgSO_4$, filtered, and had the solvent removed *in vacuo*. The residue was purified by chromatography (CH_2Cl_2 with 0→10% EtOH), to yield axle (*S*)-**S27** as a yellow oil (16.7 mg, 28%); ^{1}H NMR (400 MHz, $CDCl_3$, 298 K) δ 7.45 (s, 1H, H_h), 7.27-7.17 (m, 3H, H_f and H_g), 7.08-7.03 (m, 2H, H_e), 7.02 (t, 1H, *J* = 1.8, H_m), 6.76 (d, 2H, *J* = 1.8, H_l), 5.54 (dd, 1H, *J* = 7.4, 8.1, H_c), 5.01 (sept, 1H, *J* = 6.3, H_b), 4.05-3.93 (m, 2H, H_i), 3.46 (dd, 1H, *J* = 14.0, 7.4, one of H_d), 3.40 (dd, 1H, *J* = 14.0, 8.1, one of H_d), 2.95 (t, 2H, *J* = 7.5, H_k), 2.15 (app. quint., 2H, *J* = 7.5, H_j) 1.31 (s, 18H, H_n), 1.20 (d, 3H, *J* = 6.3, three of H_a), 1.13 (d, 3H, *J* = 6.3, three of H_a); ^{13}C NMR (101 MHz, $CDCl_3$, 298 K) δ 166.9, 157.5, 151.2, 146.3, 133.8, 128.0, 127.7, 126.4, 119.8, 113.9, 107.8, 69.3, 65.5, 63.0, 38.1, 34.0, 30.4, 28.1, 21.3, 20.6, 20.4; LR-ESI-MS (ESI+) *m/z* (%) = 506.7 [M+H]$^+$ (100); HR-ESI-MS (+ve) *m/z* = 506.3384 [M+H]$^+$ calc. 506.3377.

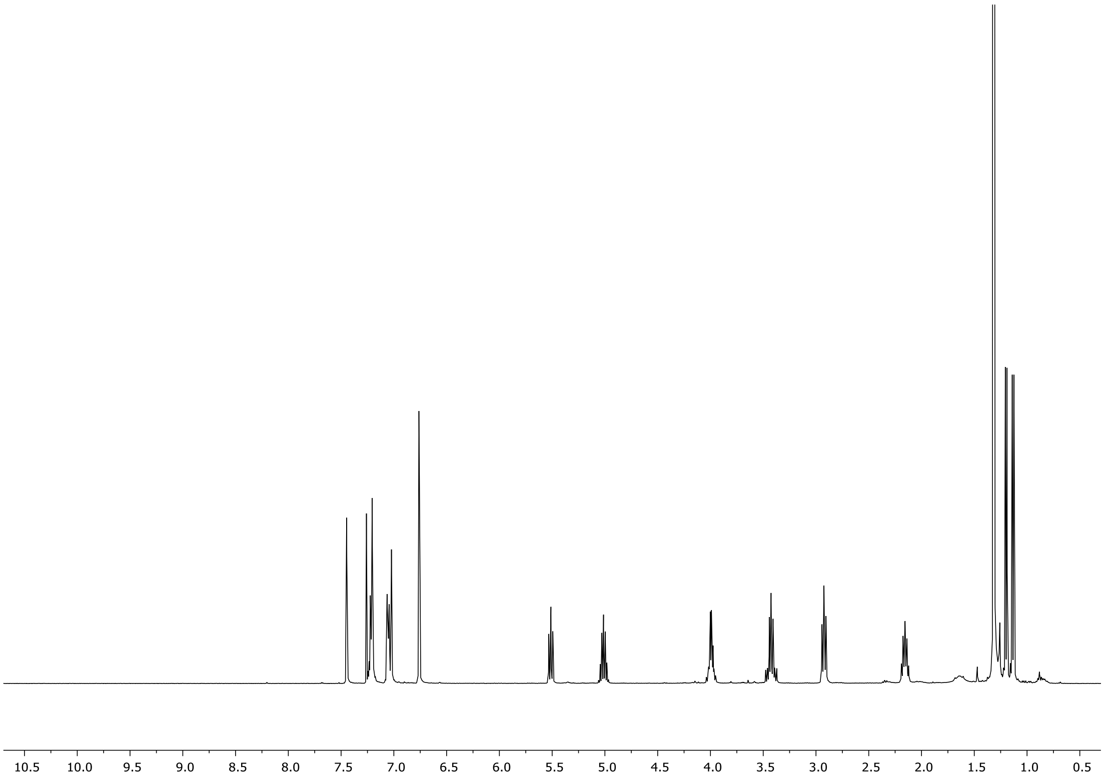

Figure S128. ^{1}H NMR (400 MHz, $CDCl_3$, 298 K) (*S*)-**S27**.

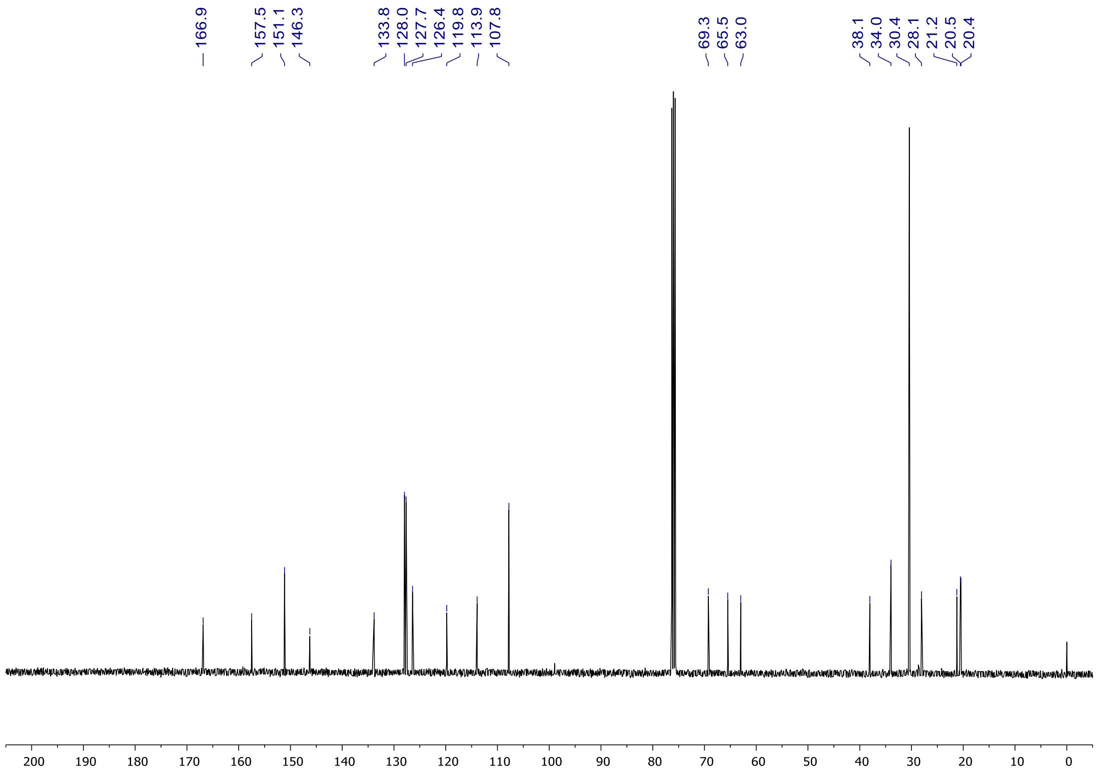

Figure S129. ^{13}C NMR (100 MHz, $CDCl_3$, 298 K) (*S*)-**S27**.

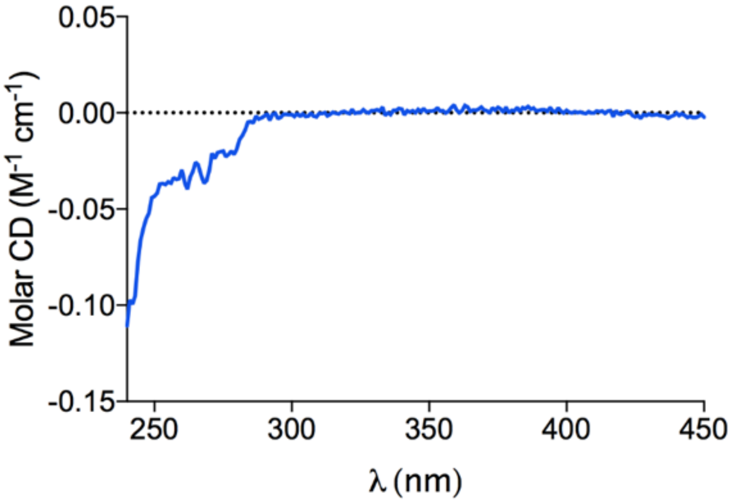

Figure S130. Circular dichroism spectrum of (*S*)-**S27** (1.12 mM in $CHCl_3$).

Rotaxane (S,R_{mp}/S_{mp})-**S28**

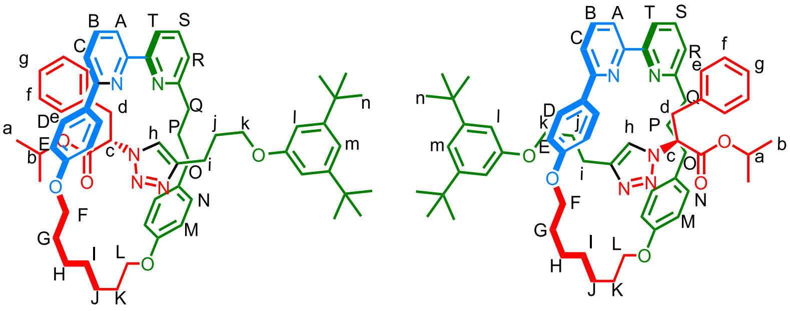

(S,S_{mp})-**S28** (S,R_{mp})-**S28**

A dry sealed vessel was charged with **2g** (22.7 mg, 0.0833 mmol), (S)-**3e** (20.0 mg, 0.0854 mmol), macrocycle **1** (19.2 mg, 0.0401 mmol), $[Cu(MeCN)_4]PF_6$ (14.6 mg, 0.0392 mmol), DIPEA (27 µL, 0.160 mmol), and anhydrous CH_2Cl_2 (1 mL). The reaction mixture was stirred at rt for 16 h, protected by an argon atmosphere. TFA (0.10 mL, 1.31 mmol) was added and the reaction mixture was stirred for an additional 16 h. Saturated EDTA-NH_3 solution (10 mL) was added, and the aqueous layer was extracted with $CHCl_3$ (3 × 20 mL), dried over $MgSO_4$, filtered, and had the solvent removed *in vacuo*. The residue containing rotaxanes (S,R_{mp}/S_{mp})-**S28** (in a 0.75 : 0.25 diastereoisomeric ratio by ^{1}H NMR, **Figure S131**) was purified by chromatography (petrol with 0→100% CH_2Cl_2, followed by 0→10% EtOH), to yield (S,R_{mp}/S_{mp})-**S28** as a yellow oil (26.4 mg, 67%, in a 0.71 : 0.29 diastereoisomeric ratio, by ^{1}H NMR, **Figure S132**); ^{1}H NMR (400 MHz, $CDCl_3$, 298 K)* δ 7.95 (s, 1H, H_h *(major)*), 7.93 (s, 1H, H_h *(minor)*), 7.76-7.62 (m, 2H, H_S and H_B), 7.56-7.46 (m, 2H H_A and H_T), 7.46-7.38 (m, 2H, H_C and H_R), 7.26 (d, 2H, J = 7.7, H_D *(major)*), 7.24 (d, 2H, J = 7.7, H_D *(minor)*), 7.25-7.17 (m, 3H, H_g and H_f), 6.97-6.92 (m, 2H, H_e), 6.91-6.89 (m, 1H, H_m), 6.79 (d, 2H, J = 8.4, H_E *(minor)*), 6.74 (d, 2H, J = 8.4, H_E *(major)*), 6.69 (d, 2H, J = 8.7, H_N *(major)*), 6.67-6.62 (m, 3H, H_l and H_N *(minor)*), 6.56 (d, 2H, J = 8.4, H_M *(minor)*), 6.51 (d, 2H, J = 8.4, H_M *(major)*), 4.93-4.83 (m, 2H, H_b and H_c), 4.18-4.05 (m, 3H, H_Q and one of H_d), 3.95-3.86 (m, 1H, one of H_F), 3.83-3.74 (m, 1H, one of H_F), 3.25-3.05 (m, 3H, H_k and one of H_d), 2.81-2.67 (m, 2H, H_L), 2.64-2.55 (m, 2H, H_i), 2.13-1.60 (m, 12H, H_G, H_H, H_I, H_J, H_K and H_P), 1.50-1.36 (m, 2H, H_j), 1.26-1.21 (m, 18H, H_n), 1.11-1.07 (m, 3H, three of H_a), 1.02 (d, 3H, J = 6.4, three of H_a *(minor)*) 0.97 (d, 3H, J = 6.4, three of H_a *(major)*); ^{13}C NMR (126 MHz, $CDCl_3$, 298 K) δ 167.9, 167.8, 163.2, 163.1, 159.4, 159.3, 159.0, 159.0, 158.6, 158.5, 158.0, 158.0, 157.8, 157.7, 157.4, 157.4, 151.4, 151.4, 148.2, 148.1, 136.7, 136.7, 136.7, 135.7, 135.6, 132.8, 132.8, 132.8, 129.4, 129.4, 129.1, 129.1, 128.9, 128.9, 128.5, 128.44, 127.1, 127.1, 122.3, 122.3, 122.2, 122.2, 122.0, 122.0, 120.3, 120.3, 120.2, 120.1, 119.9, 119.8, 115.5, 115.5, 114.4, 114.3, 113.6, 113.6, 109.0, 109.0, 69.6, 69.6, 67.8, 67.8, 67.3, 67.2, 66.2, 66.2, 63.6, 63.5, 38.0, 38.0, 37.9, 37.8, 37.7, 35.3, 35.3, 34.9, 32.5, 32.4, 31.5, 31.5, 29.7, 29.7, 29.5, 29.5, 28.8, 28.8, 28.5, 27.5, 27.4, 26.0, 25.9, 22.7, 22.1, 22.1, 21.5, 21.2, 21.4, 21.4; LR-MS-ESI m/z (%) = 984.9 (100); HR-ESI-MS (+ve) m/z = 984.6018 $[M+H]^+$ calc. 984.5997.

*As stereochemistry could not be unambiguously assigned, the signals are simply designated (*major*) or (*minor*). Proton counts are provided for each signal and represent the expected integration of that environment. Where the major and minor diastereoisomer signals are coincident, no (*major*)/(*minor*) label is provided and the proton count indicated refers the expected integration of that signal in each of the stereoisomers that contributes to the multiplet.

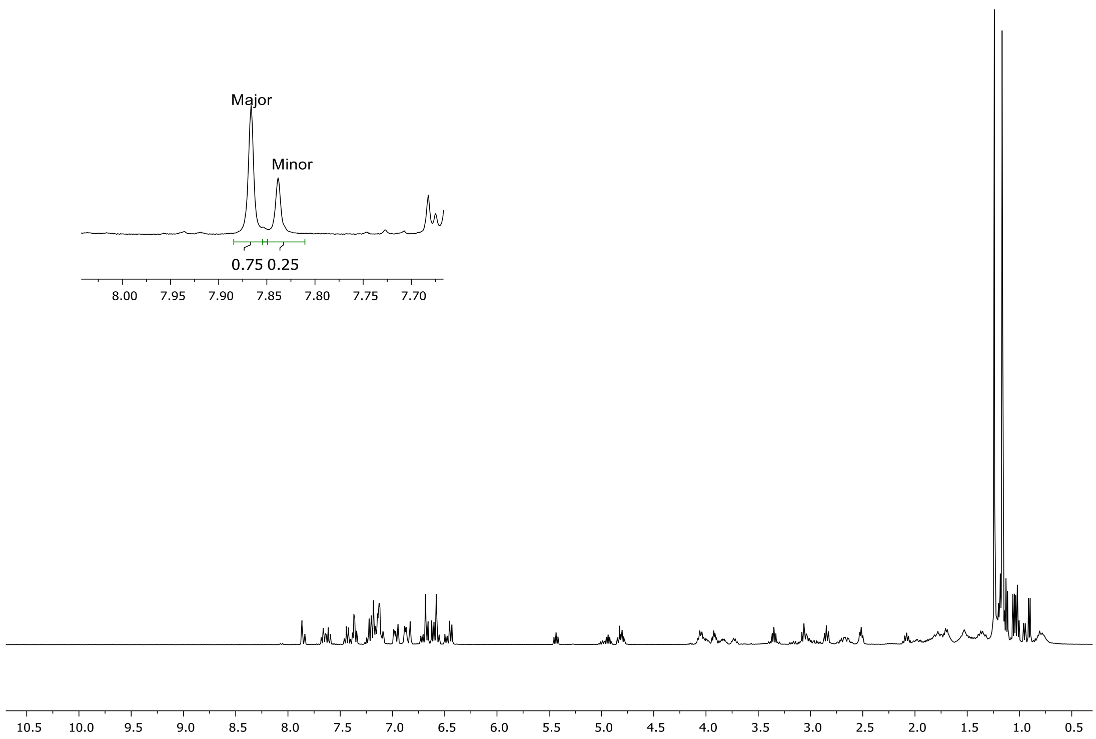

Figure S131. ^{1}H NMR (400 MHz, $CDCl_3$, 298 K) (S,R_{mp}/S_{mp})-**S28**, prior to purification by chromatography.

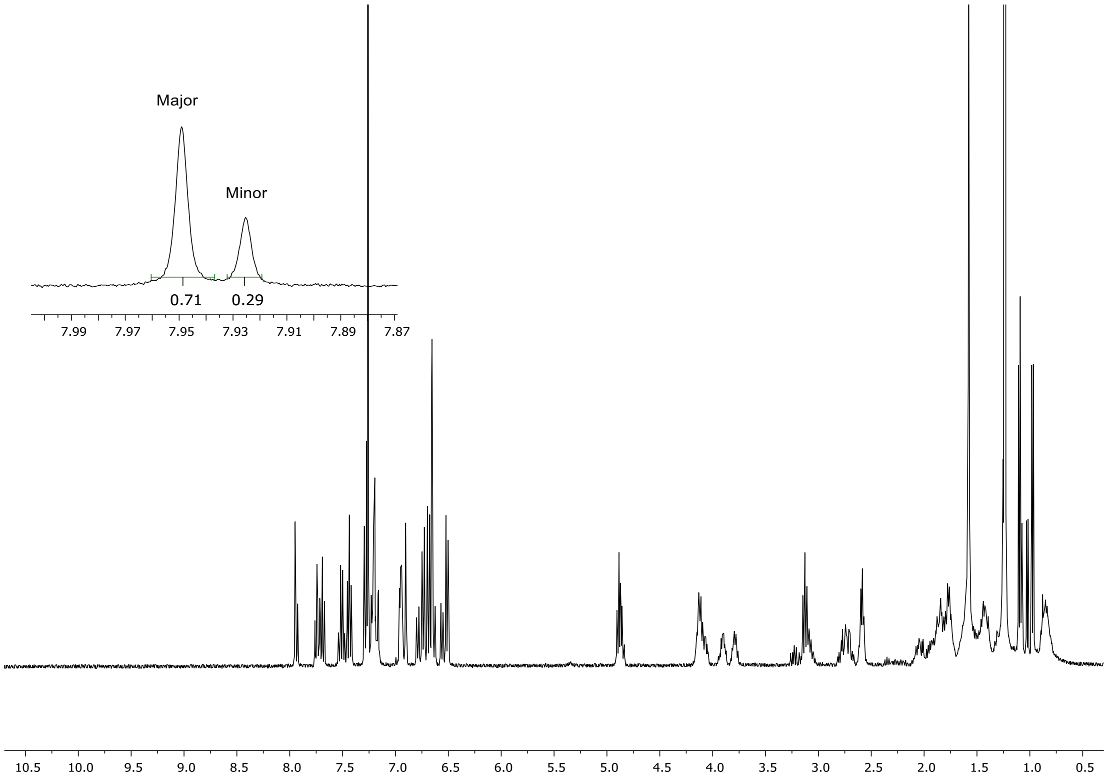

Figure S132. ^{1}H NMR (500 MHz, $CDCl_3$, 298 K) (S,R_{mp}/S_{mp})-**S28**, following purification by chromatography.

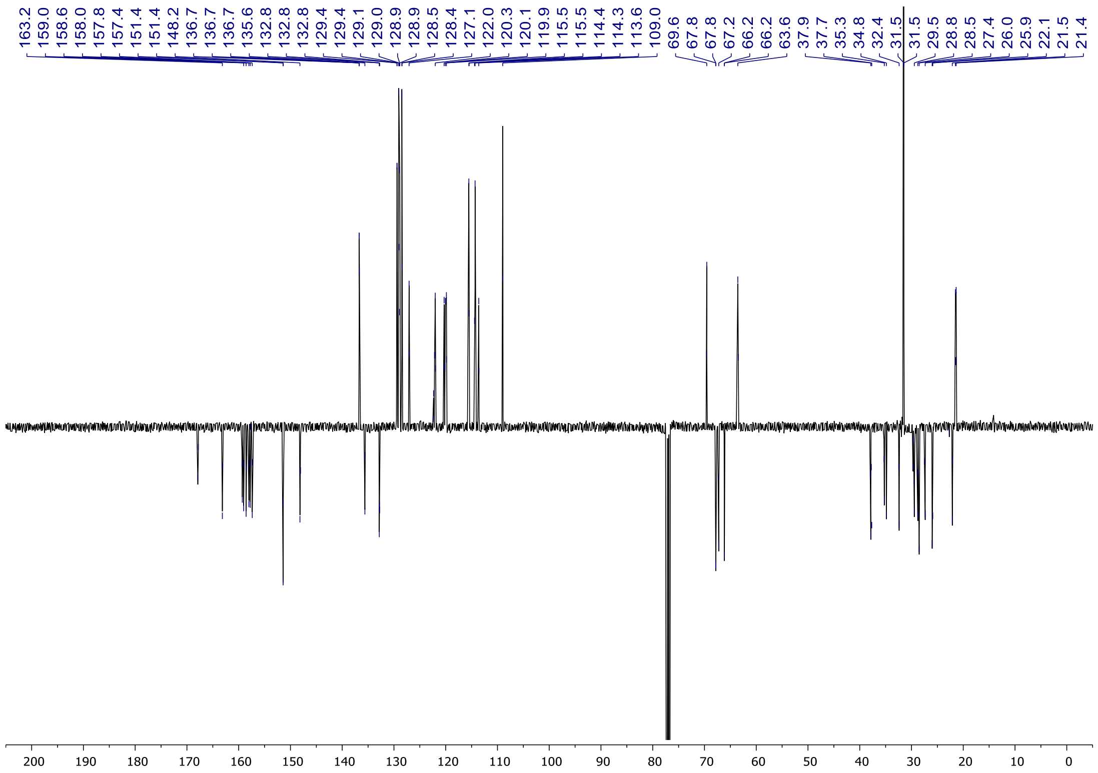

Figure S133. ^{13}C NMR (126 MHz, $CDCl_3$, 298 K) (*S*,*R*$_{mp}$/*S*$_{mp}$)-**S28**, following purification by chromatography.

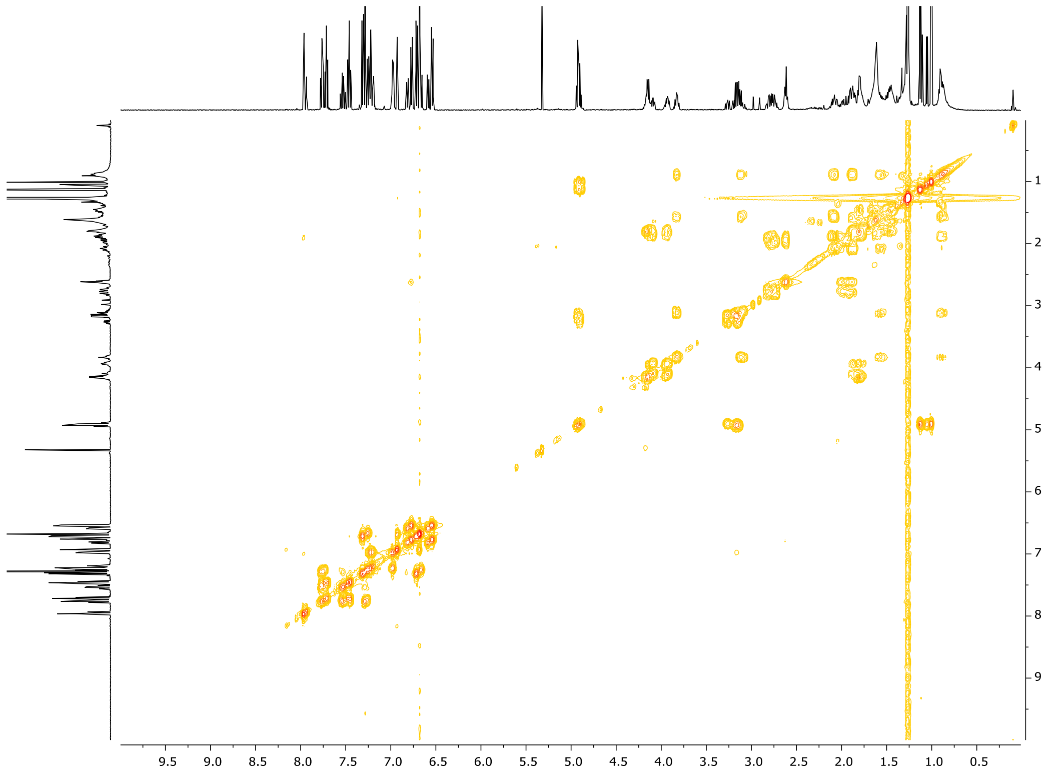

Figure S134. ^{1}H-^{1}H COSY NMR (126 MHz, $CDCl_3$, 298 K) (*S*,*R*$_{mp}$/*S*$_{mp}$)-**S28**, following purification by chromatography.

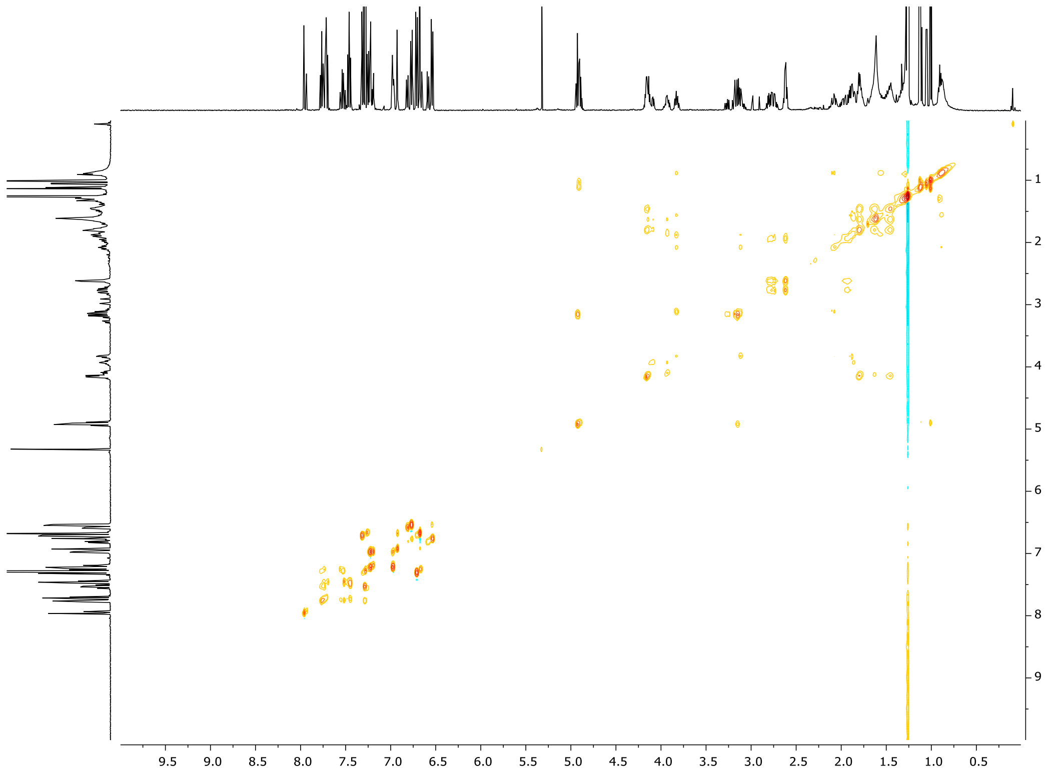

Figure S135. ^{1}H-^{1}H TOCSY NMR (126 MHz, $CDCl_3$, 298 K) (S,R_{mp}/S_{mp})-**S28**, following purification by chromatography.

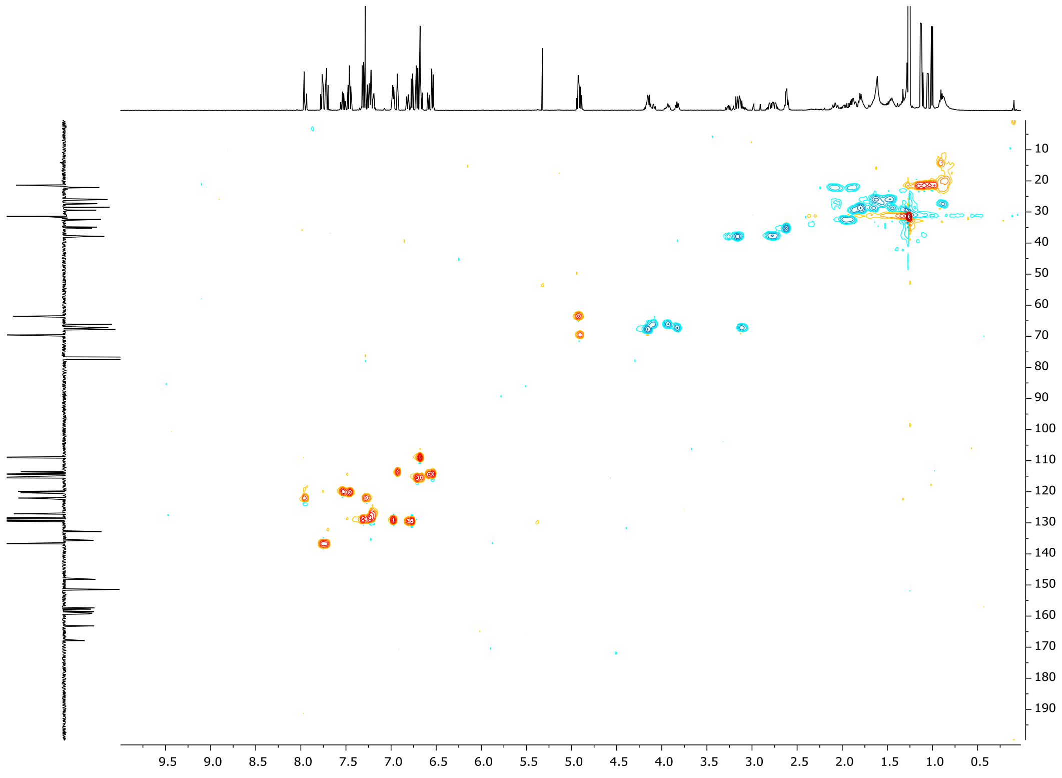

Figure S136. ^{1}H-^{13}C HSQC NMR (126 MHz, $CDCl_3$, 298 K) (S,R_{mp}/S_{mp})-**S28**, following purification by chromatography.

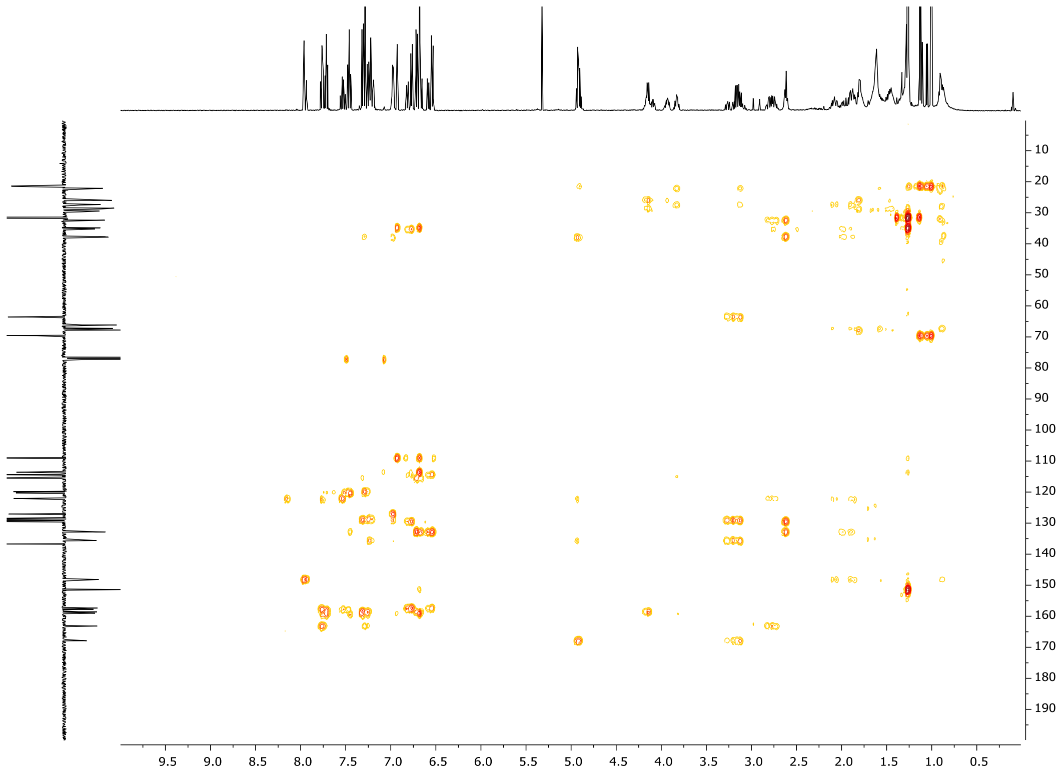

Figure S137. ^{1}H-^{13}C HMBC NMR (126 MHz, $CDCl_3$, 298 K) (S,R_{mp}/S_{mp})-**S28**, following purification by chromatography.

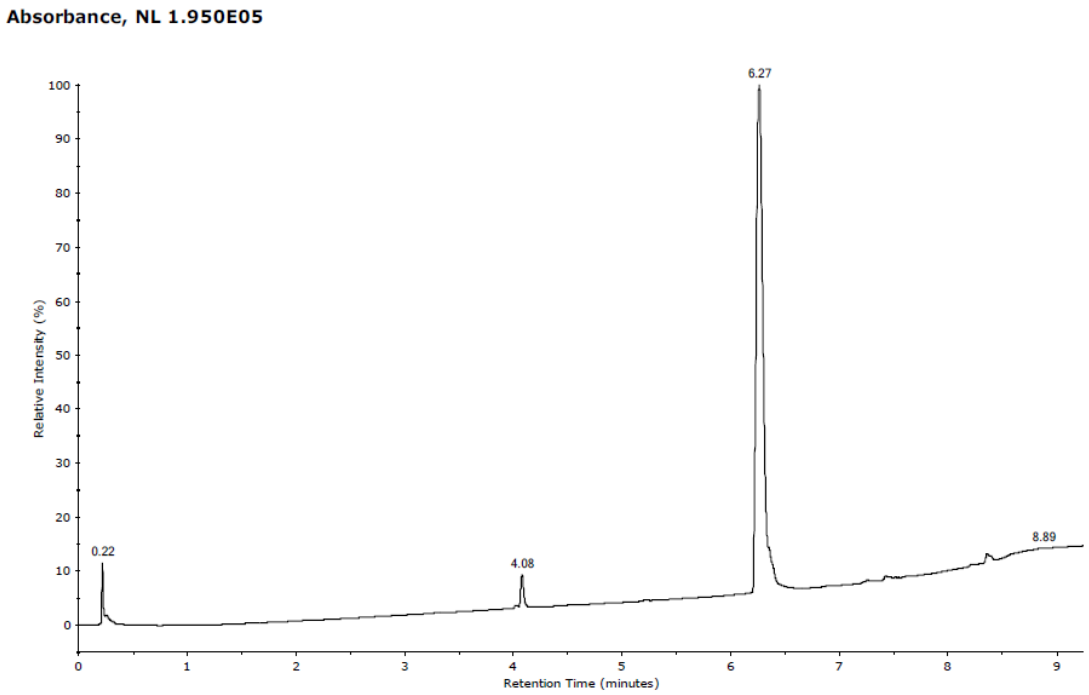

Figure S138. LCMS trace (C_{18} column, gradient 10 minutes (1 : 4 MeCN+0.2% formic acid-H_2O +0.2% formic acid →1 : 0 MeCN-H_2O +0.2% formic acid), UV 254 nm), of (S,R_{mp}/S_{mp})-**S28** following purification by chromatography

Entry 11 – axle (*S*)-S29 and rotaxane (*S*,*R*$_{mp}$/*S*$_{mp}$)-S30 derived from alkyne 2h and azide (*S*)-3e

Axle (*S*)-**S29**

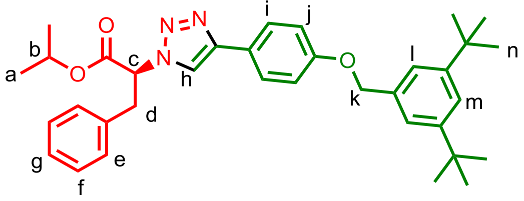

A 5 mL round bottomed flask was charged with **2h** (27.8 mg, 0.0867 mmol), (*S*)-**3e** (18.1 mg, 0.0776 mmol), $CuSO_4.5H_2O$ (19.3 mg, 0.0776 mmol), sodium *L*-ascorbate (21.4 mg, 0.108 mmol), and DMF (2 mL) was stirred at rt for 16 h. Saturated EDTA-NH_3 solution (15 mL) was added, and the aqueous layer was extracted with EtOAc (3 × 20 mL). The combined organic extracts were washed with 5% w/v LiCl (5 × 10 mL), dried over $MgSO_4$, filtered, and had the solvent removed *in vacuo*. The residue was purified by chromatography (CH_2Cl_2 with 0→10% EtOH), to yield axle (*S*)-**S29** as a yellow oil (27.6 mg, 67%); ^{1}H NMR (400 MHz, $CDCl_3$, 298 K) δ 7.7 (s, 1H, H_h), 7.67 (dt, 2H, *J* = 9.0, 2.2, H_i), 7.34 (t, 1H, *J* = 1.8, H_m), 7.22 (d, 2H, *J* = 1.8, H_l), 7.19-7.14 (m, 3H, H_f and H_g), 7.07-7.01 (m, 2H, H_e), 6.98 (dt, 2H, *J* = 9.0, 2.2, H_j), 5.54 (dd, 1H, *J* =7.4, 7.6, H_c), 5.02-4.90 (m, 3H, H_b and H_k), 3.44 (dd, 1H, *J* = 14.2, 7.4, one of H_d), 3.40 (dd, 1H, *J* = 14.2, 7.6, one of H_d), 1.27 (s, 18H, H_n), 1.15 (d, 3H, *J* = 6.3, three of H_a), 1.07 (d, 3H, *J* = 6.3, three of H_a); ^{13}C NMR (101 MHz, $CDCl_3$, 298 K) δ 168.0, 159.3, 151.2, 147.7, 135.9, 135.0, 129.2, 128.9, 127.7, 127.2, 123.6, 122.4, 122.3, 118.7, 115.3, 71.1, 70.6, 64.3, 39.4, 35.0, 31.6, 21.7, 21.6; LR-ESI-MS (+ve) *m/z* (100) = 554.6 $[M+H]^+$ (100); HR-ESI-MS (+ve) *m/z* = 554.3384 $[M+H]^+$ calc. 554.3377.

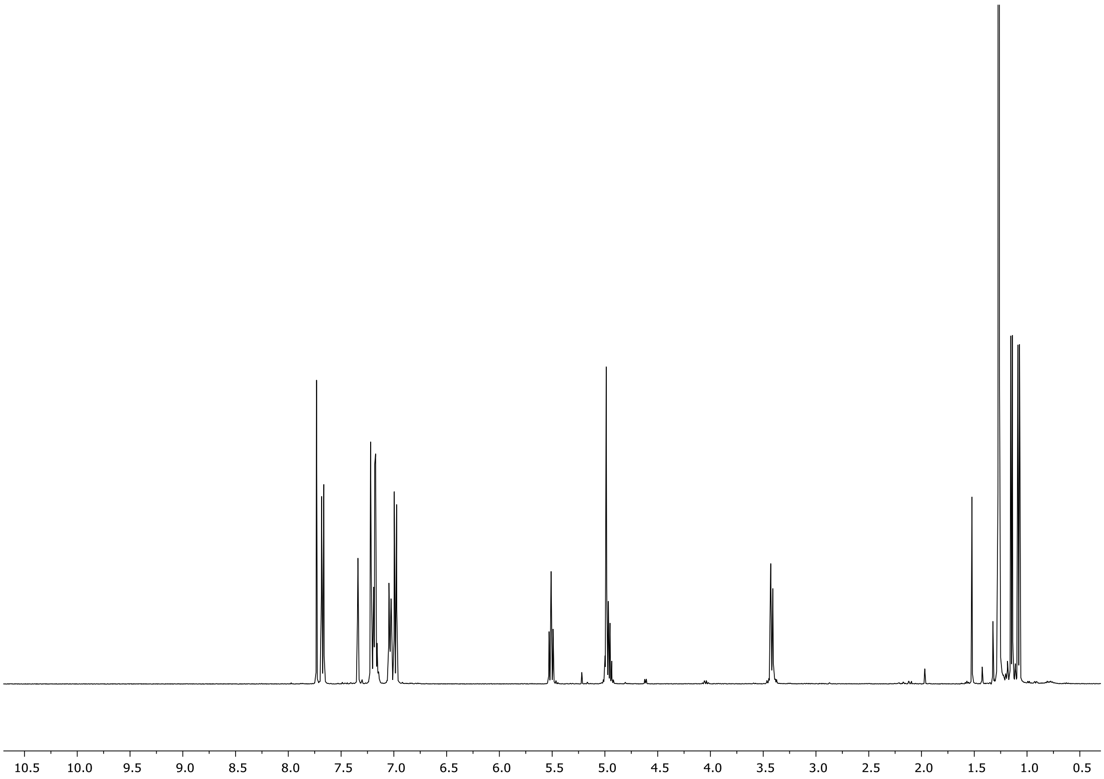

Figure S139. ^{1}H NMR (400 MHz, $CDCl_3$, 298 K) (*S*)-**S29**.

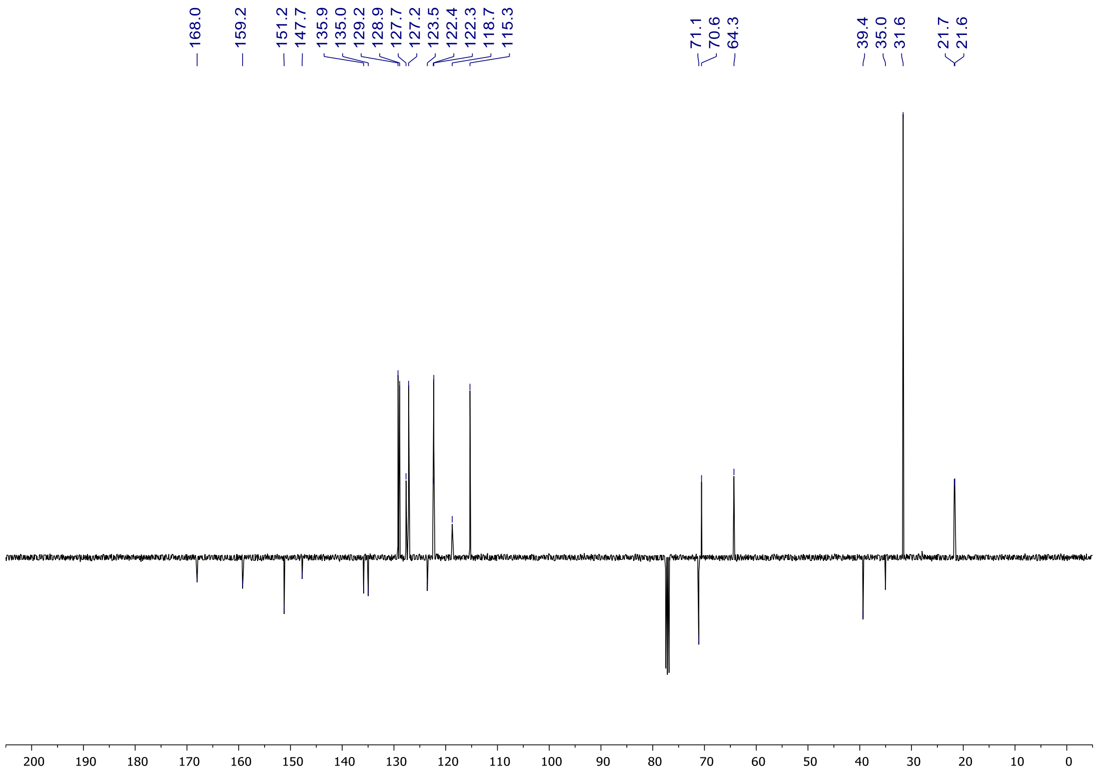

Figure S140. ^{13}C NMR (101 MHz, $CDCl_3$, 298 K) (*S*)-**S29**.

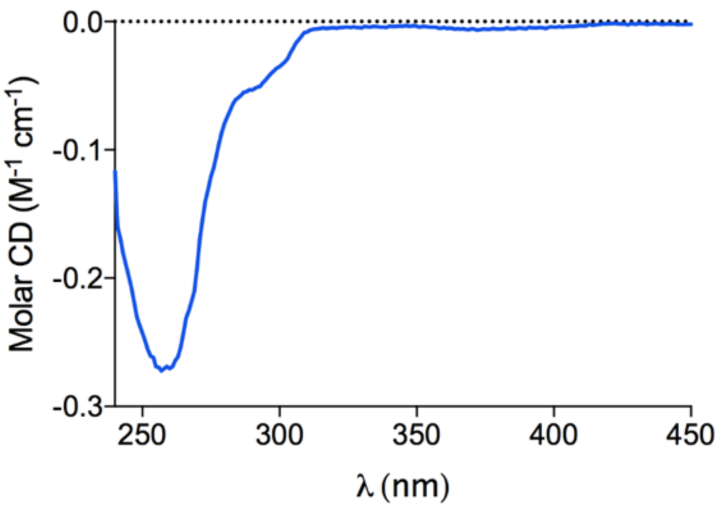

Figure S141. Circular dichroism spectrum of (*S*)-**S29** (57.1 μM in $CHCl_3$).

Rotaxanes (S,R_{mp}/S_{mp})-**S30**

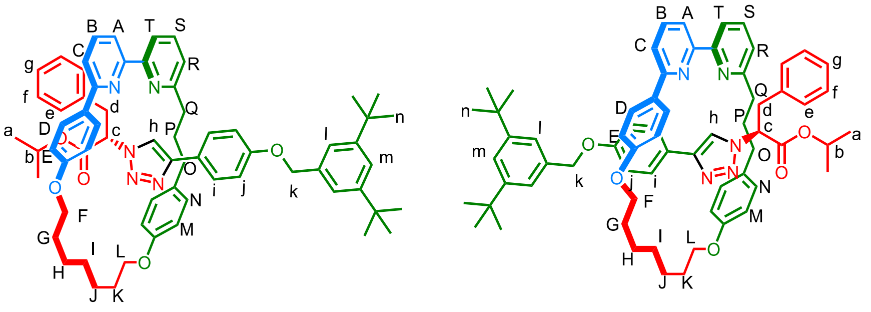

(S,S_{mp})-**S30** (S,R_{mp})-**S30**

A dry sealed vessel was charged with **2h** (55.7 mg, 0.174 mmol), (S)-**3e** (40.6 mg, 0.174 mmol), macrocycle **1** (41.4 mg, 0.0859 mmol), $[Cu(MeCN)_4]PF_6$ (30.9 mg, 0.829 mmol), DIPEA (58 µL, 0.344 mmol), and anhydrous CH_2Cl_2 (2 mL). The reaction mixture was stirred at rt for 16 h, protected by an argon atmosphere. TFA (0.10 mL, 1.31 mmol) was added and the reaction mixture was stirred for an additional 16 h. Saturated EDTA-NH_3 solution (20 mL) was added, and the aqueous layer was extracted with CH_2Cl_2 (3 × 30 mL), dried over $MgSO_4$, filtered, and had the solvent removed *in vacuo* The residue containing rotaxanes (S,R_{mp}/S_{mp})-**S30** (in a 0.84 : 0.16 diastereoisomeric ratio by ^{1}H NMR, **Figure S142**) was purified by chromatography(petrol with 0→100% Et_2O), to yield rotaxanes (S,R_{mp}/S_{mp})-**S30** as a yellow oil (72.0 mg, 76%, in a 0.83 : 0.17 diastereoisomeric ratio and 5% of macrocycle 1 by ^{1}H NMR, **Figure S143**); ^{1}H NMR (400 MHz, $CDCl_3$, 298 K)* δ 9.31 (s, 1H, H_h *(major)*), 9.21 (s, 1H, H_h *(minor)*), 7.83-7.68 (m, 4H, H_j, H_S and H_B), 7.62 (d, 1H, J = 7.8, H_A), 7.5l (d, 1H, J = 7.6, H_T), 7.46 (d, 1H, J = 7.8, H_R), 7.39-7.36 (m, 1H, H_m), 7.31 (d, 1H, J = 7.7, H_C), 7.25-7.15 (m, 8H, H_e, H_i, H_D and H_l), 6.88-6.62 (m, 3H, H_f and H_g), 6.49-6.42 (m, 2H, H_N), 6.34-6.29 (m, 2H, H_E), 6.22-6.14 (m, 2H, H_M), 5.06 (d, 1H, J = 11.0, one of H_k), 4.90-4.47 (m, 3H, H_b, H_c and one of H_k) 4.30-4.20 (m, 1H, one of H_F), 4.10-3.93 (m, 2H, H_Q), 3.84-3.76 (m, 1H, one of H_F), 2.91-2.71 (m, 4H, H_d and H_O), 2.58-2.48 (m, 1H, one of H_L), 2.47-2.38 (m, 1H, one of H_L), 2.16-1.58 (m, 12H, H_G, H_H, H_I, H_J, H_K and H_P), 1.35-1.31 (m, 18H, H_n), 1.12 (d, 3H, J = 6.4, three of H_a), 0.89 (d, 3H, J = 6.1, three of H_a); ^{13}C NMR (126 MHz, $CDCl_3$, 298 K) δ 167.9, 163.8, 159.1, 159.0, 158.3, 157.9, 157.7, 156.9, 150.9, 147.0, 137.1, 137.1, 136.6, 135.6, 131.9, 131.3, 129.3, 128.7, 128.5, 128.5, 127.2, 127.1, 124.7, 122.5, 122.3, 122.0, 121.7, 120.0, 120.0, 119.4, 114.9, 114.3, 114.1, 70.82 69.4, 67.8, 65.4, 63.6, 30.0, 37.5, 35.4, 35.0, 32.56, 31.7, 29.2, 28.9, 28.1, 25.9, 25.7, 21.7, 21.4; HR-ESI-MS (+ve) m/z = 1032.6 $[M+H]^+$ calc. 1032.0.

* As stereochemistry could not be unambiguously assigned, the signals are simply designated (*major*) or (*minor*). Proton counts are provided for each signal and represent the expected integration of that environment. Where the major and minor diastereoisomer signals are coincident, no (*major*)/(*minor*) label is provided and the proton count indicated refers the expected integration of that signal in each of the stereoisomers that contributes to the multiplet.

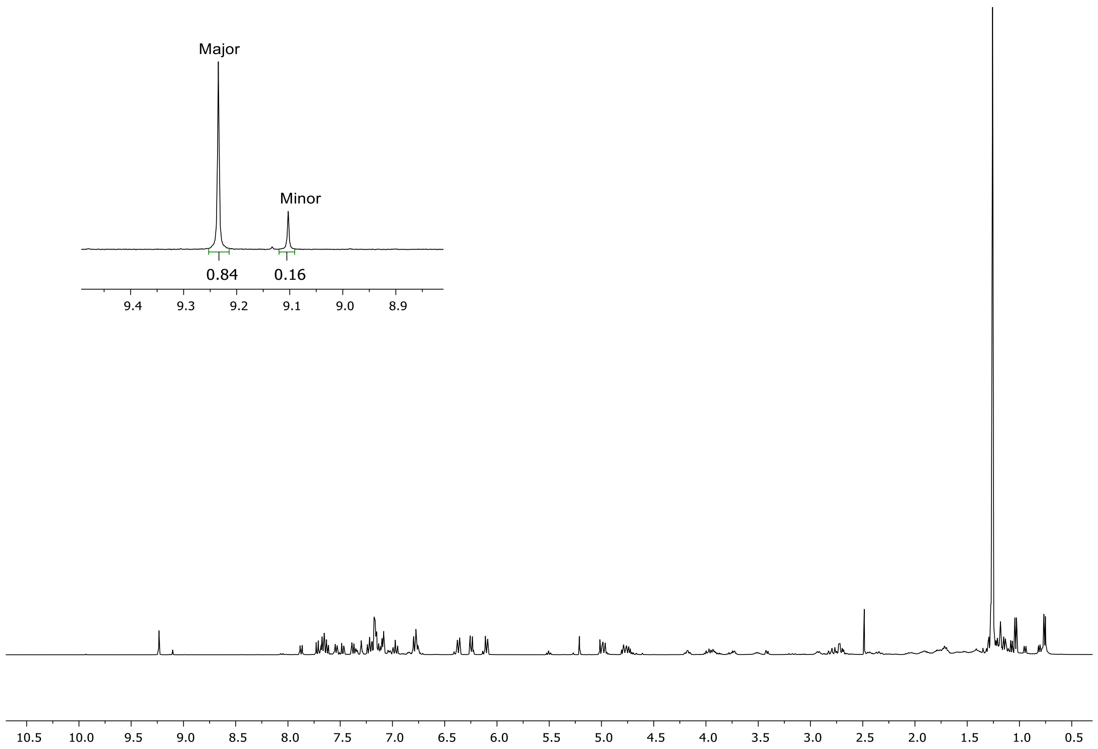

Figure S142. ^{1}H NMR (400 MHz, $CDCl_3$, 298 K) (*S*,R_{mp}/S_{mp})-**S30**, prior to purification by chromatography.

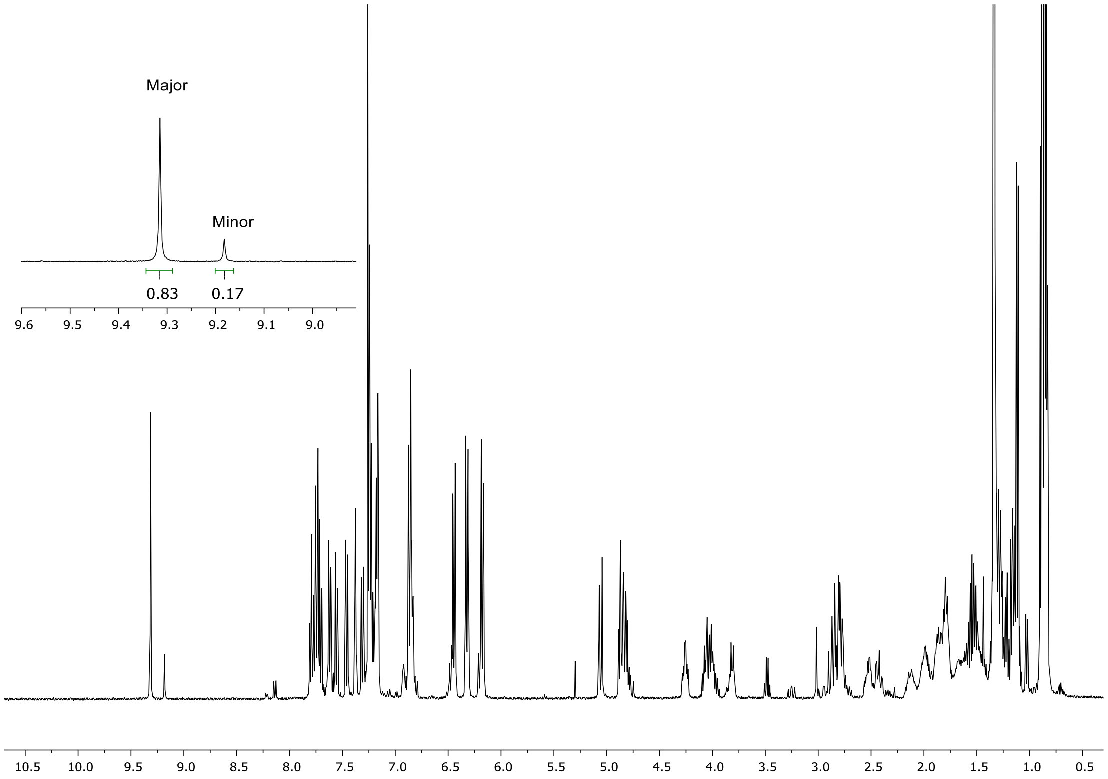

Figure S143. ^{1}H NMR (500 MHz, $CDCl_3$, 298 K) (*S*,R_{mp}/S_{mp})-**S30**, following purification by chromatography.

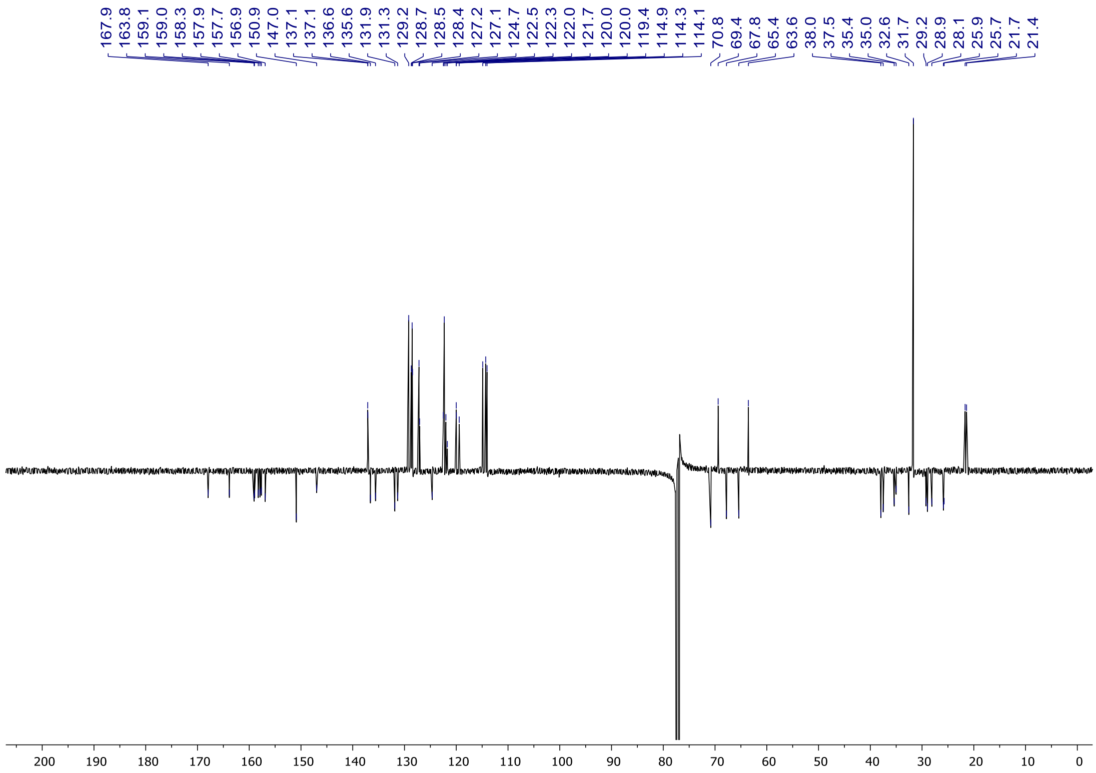

Figure S144. ^{13}C NMR (126 MHz, $CDCl_3$, 298 K) (S,R_{mp}/S_{mp})-**S30**, following purification by chromatography.

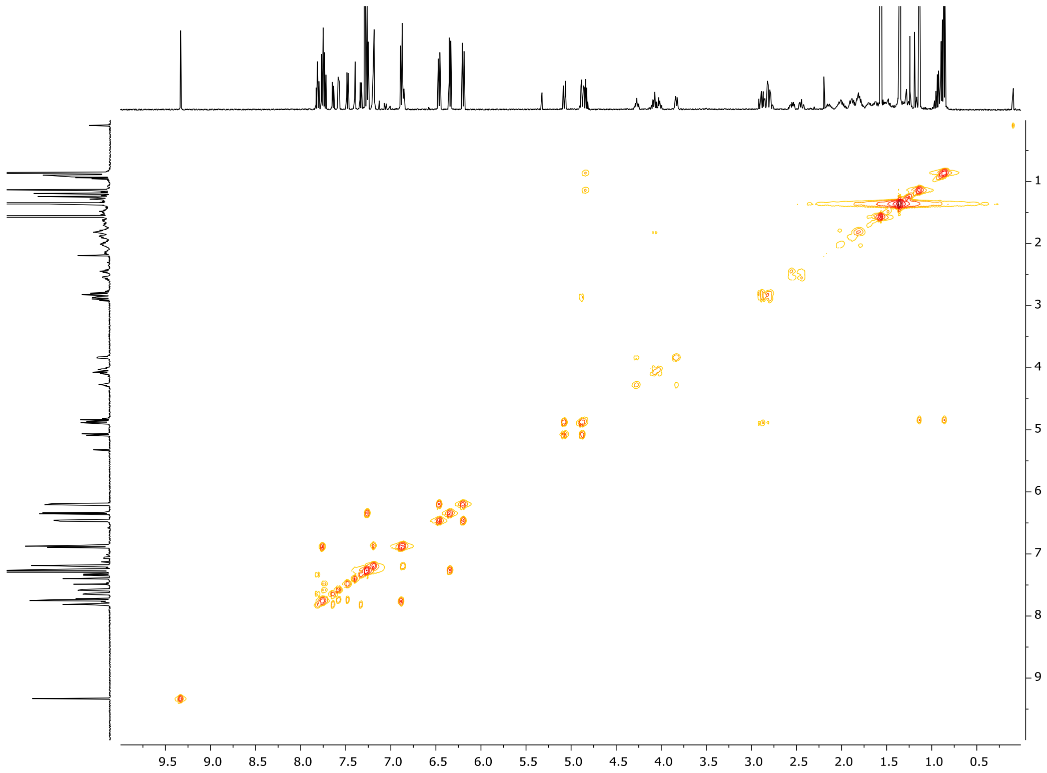

Figure S145. ^{1}H-^{1}H COSY (500 MHz, $CDCl_3$, 298 K) (S,R_{mp}/S_{mp})-**S30**, following purification by chromatography.

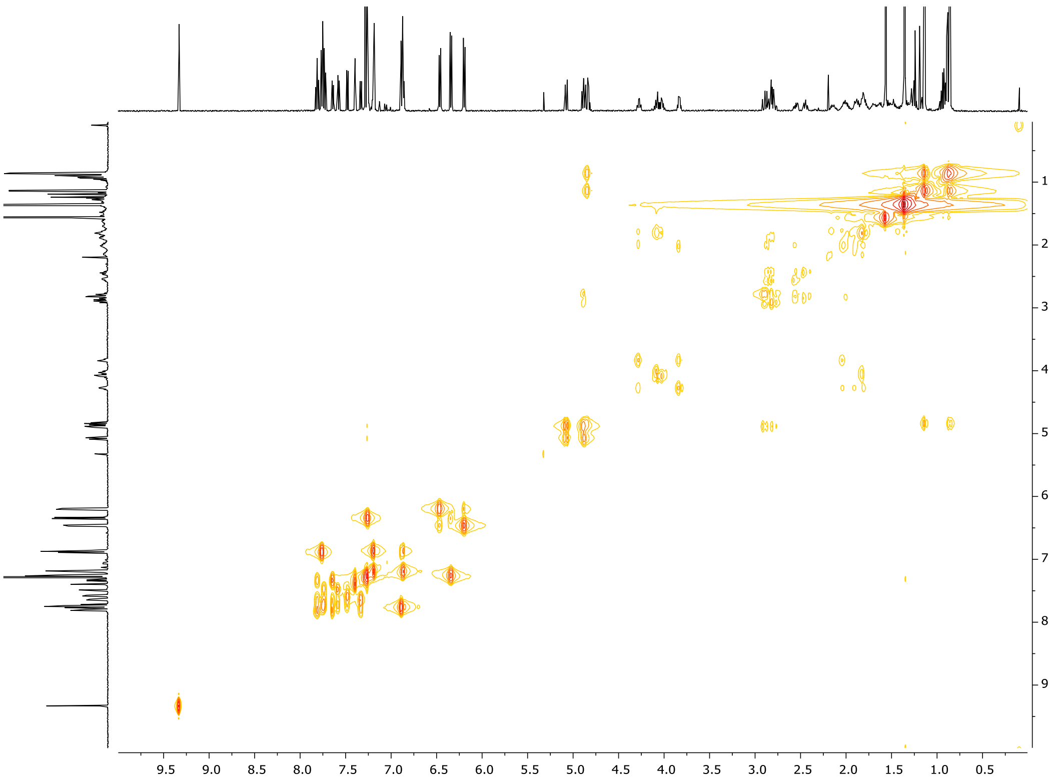

Figure S146. ^{1}H-^{1}H TOCSY (500 MHz, $CDCl_3$, 298 K) (S,R_{mp}/S_{mp})-**S30**, following purification by chromatography.

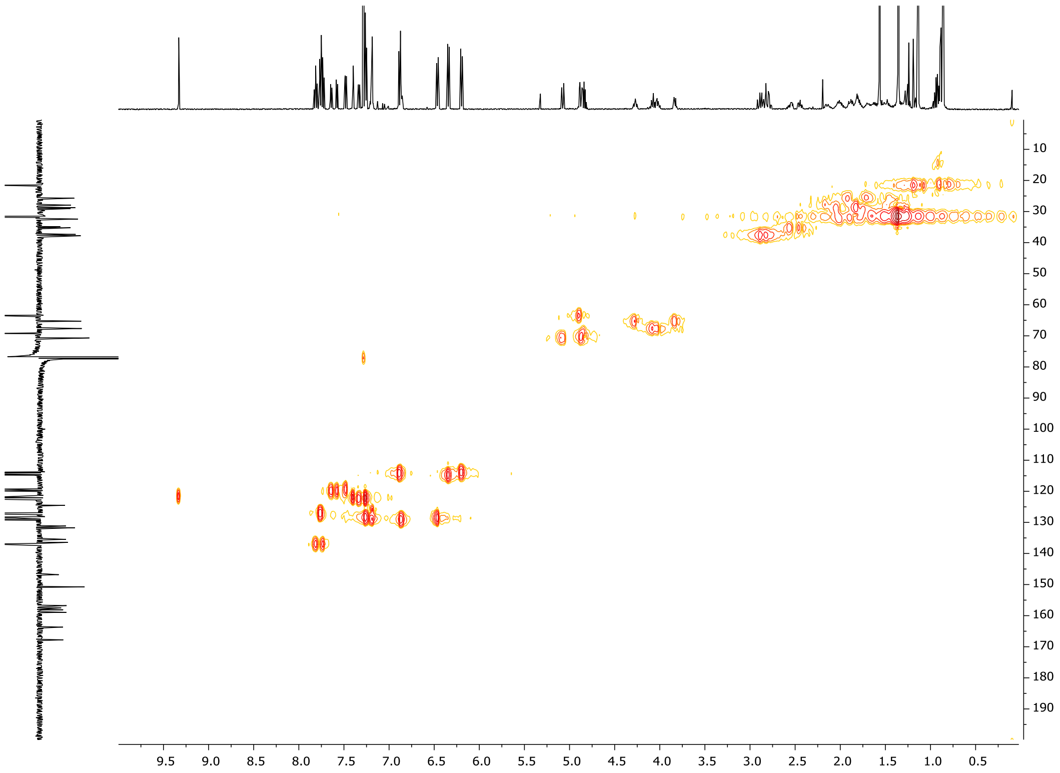

Figure S147. ^{1}H-^{13}C HSQC (126 MHz, $CDCl_3$, 298 K) (S,R_{mp}/S_{mp})-**S30**, following purification by chromatography.

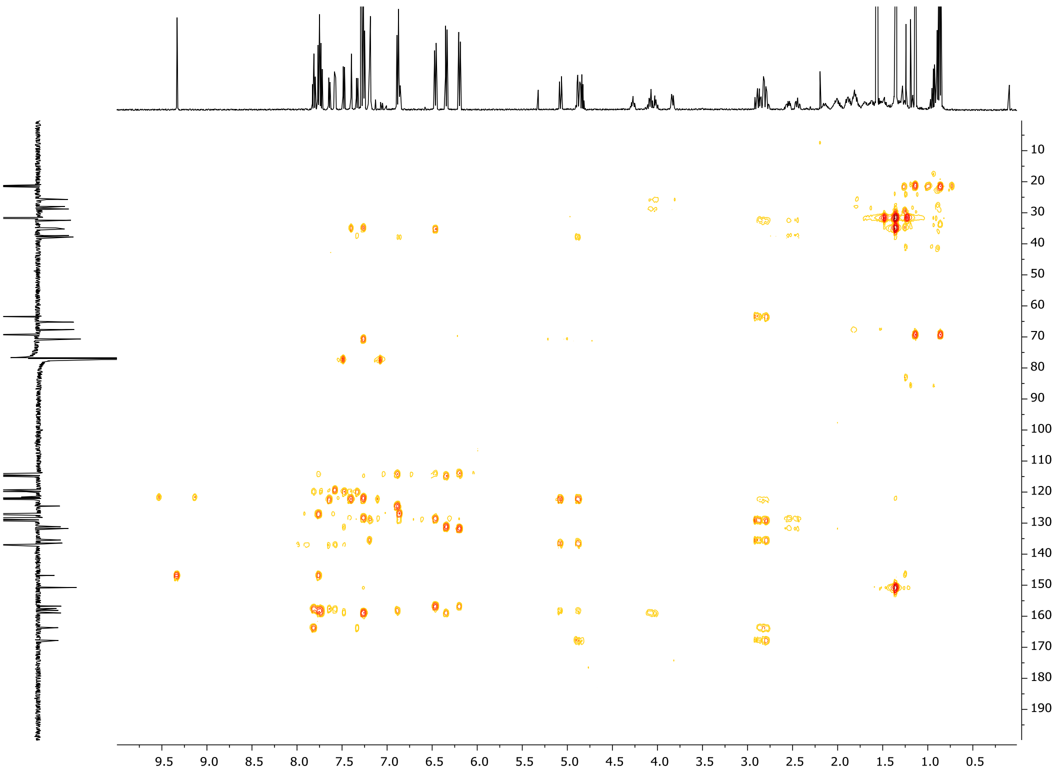

Figure S148. ^{1}H-^{13}C HMBC (126 MHz, $CDCl_3$, 298 K) (S,R_{mp}/S_{mp})-**S30**, following purification by chromatography.

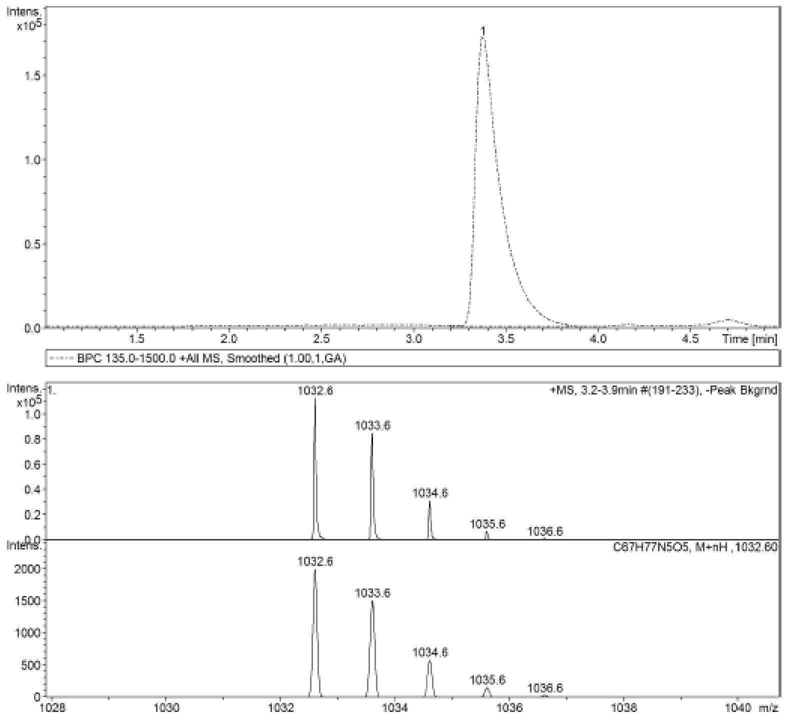

Figure S149. LCMS trace (C_{18} column, gradient 5 minutes (1 : 4 MeCN+0.2% formic acid-H_2O +0.2% formic acid →1 : 0 MeCN-H_2O +0.2% formic acid), UV 254 nm), of (S,R_{mp}/S_{mp})-**S30** following purification by chromatography (top), and ESI-MS isotopic pattern of (S,R_{mp}/S_{mp})-**S30**; observed (middle) and calculated (bottom).

4. Alkylation of axle S20 and rotaxanes 4

Axle **S31**‡

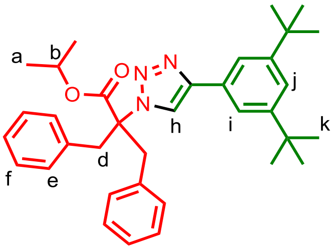

A dry sealed vessel was charged with axle (*S*)-**S20** (19.8 mg, 0.0442 mmol), LiHMDS (0.50 mL of a 1 M solution in THF, 0.50 mmol) and anhydrous THF (1 mL). The reaction mixture was stirred at −78 °C for 30 minutes, protected by a nitrogen atmosphere. Benzyl bromide (52 μL, 0.442 mmol) was added and the reaction mixture was stirred, with warming to rt for 16 h. Saturated NH_4Cl (20 mL) was added, and the aqueous layer was extracted with EtOAc (3 × 20 mL). The combined organic extracts were dried over $MgSO_4$, filtered, and had the solvent removed *in vacuo*. The residue was purified by chromatography (CH_2Cl_2 with 0→10% EtOH), to yield axle **S31** as a yellow oil (10.6 mg, 44%); ^{1}H NMR (400 MHz, $CDCl_3$, 298 K) δ 7.60 (s, 1H, H_g), 7.52 (d, 2H, *J* = 1.8, H_h), 7.39 (t, 1H, *J* = 1.8, H_i), 7.19-7.12 (m, 6H, H_e and H_f), 6.90-6.85 (m, 4H, H_d), 5.02 (sept, 1H, *J* = 6.1, H_b), 3.99 (d, 2H, *J* = 14, two of H_c), 3.71 (d, 2H, *J* = 14, two of H_c), 1.35 (s, 18H, H_j), 1.64 (d, 6H, *J* = 6.1, H_a); ^{13}C NMR (101 MHz, $CDCl_3$, 298 K) δ 168.5, 151.2, 147.1, 134.6, 130.1, 130.0, 128.6, 128.3, 127.4, 122.9, 122.3, 122.1, 120.2, 74.5, 70.7, 45.2, 34.9, 31.5, 21.7; LR-ESI-MS (+ve) *m/z* (%) = 538.6 $[M+H]^+$ (100); HR-ESI-MS (+ve) *m/z* = 538.3435 $[M+H]^+$ calc. 538.3428.

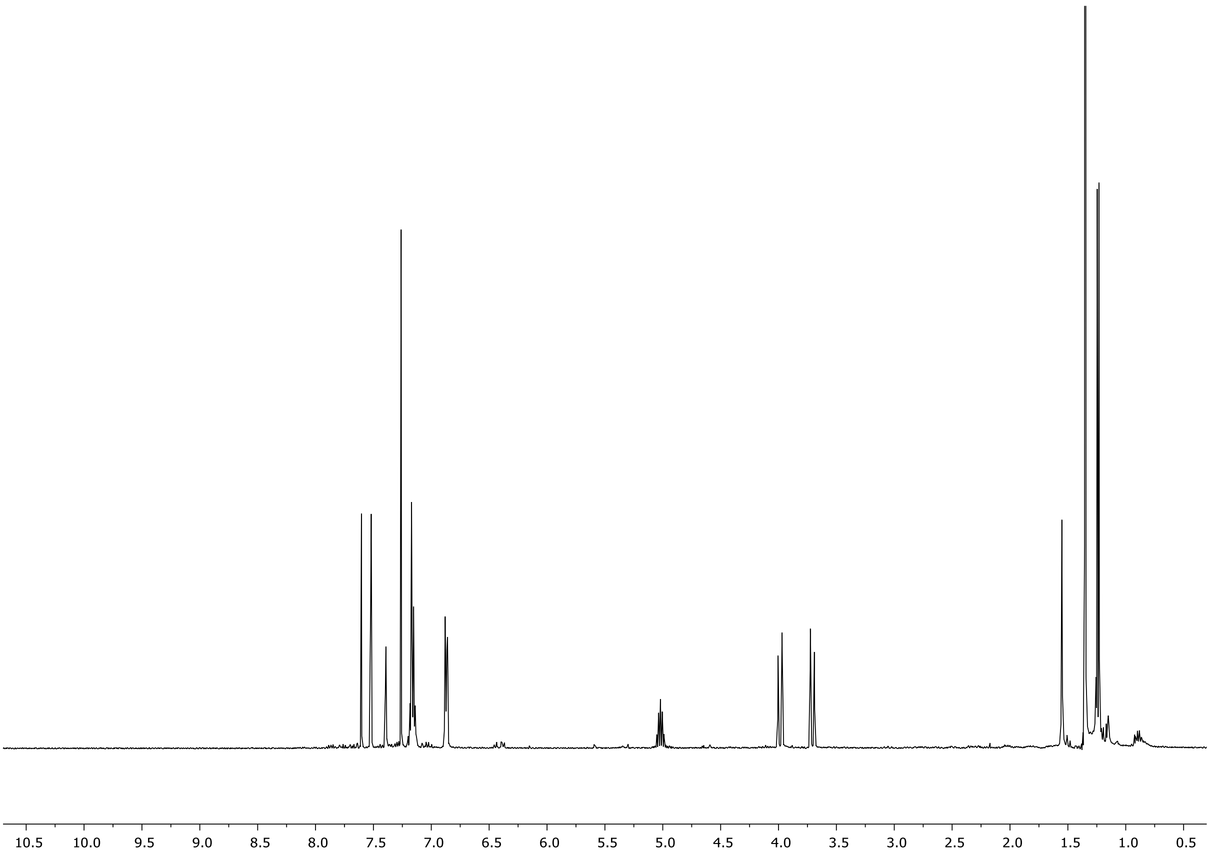

Figure S150. ^{1}H NMR (400 MHz, $CDCl_3$, 298 K) **S31**.

‡ For comparison with axle **S20**, no proton is designated as H_c.

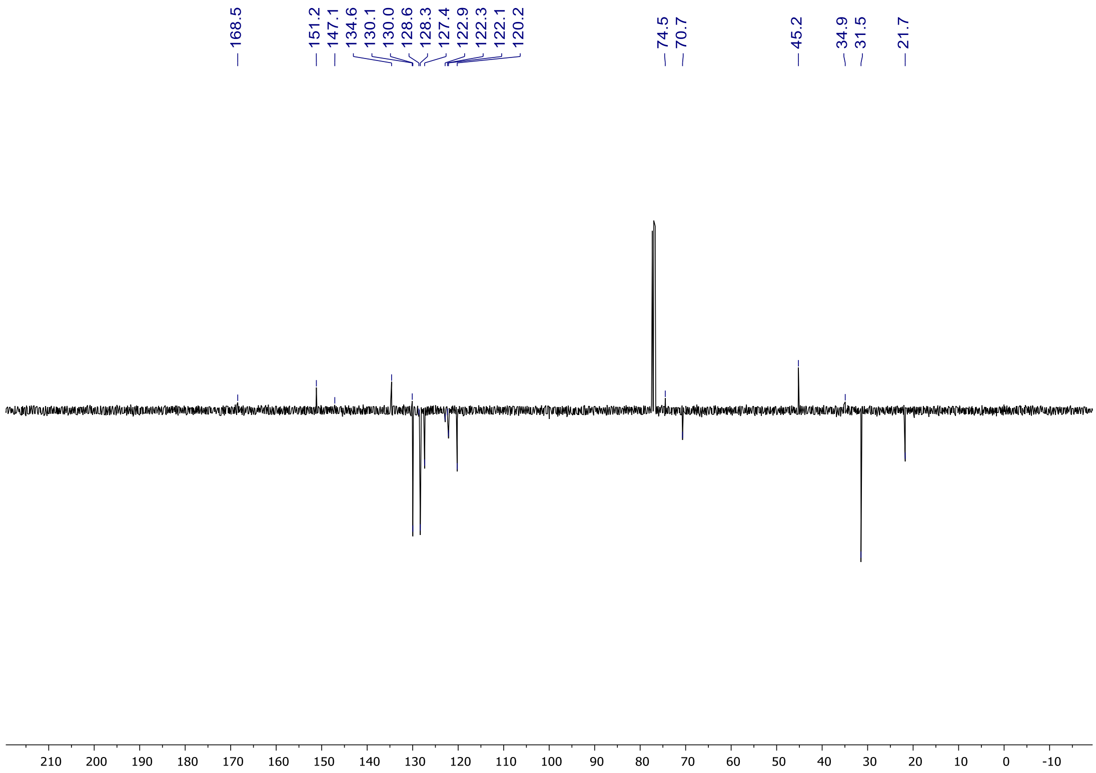

Figure S151. ^{13}C NMR (101 MHz, $CDCl_3$, 298 K) **S31**.

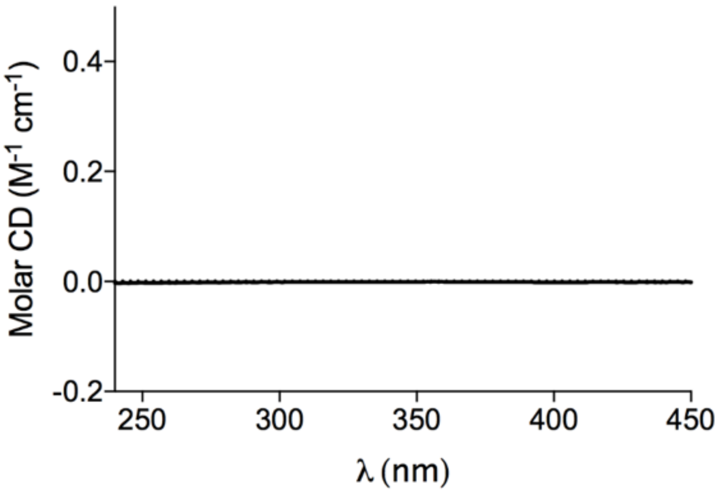

Figure S152. Circular dichroism spectrum of **S31** (70.6 μM in $CHCl_3$).

Rotaxane (S_{mp})-**5**‡

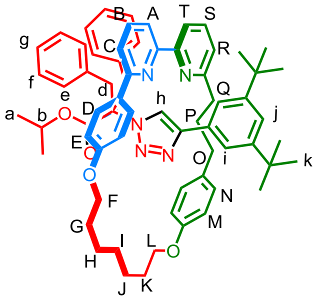

In a dry sealed vessel, (S,S_{mp})-**4** (33.8 mg, 0.0365 mmol), in LiHMDS (0.50 mL of a 1 M solution in THF, 0.50 mmol) was stirred at –78 °C for 10 min, protected by a nitrogen atmosphere. **S8** (62.4 mg, 0.286 mmol) in anhydrous THF (0.5 mL) and was added and the reaction mixture was stirred, with warming to rt for four days. Saturated NH_4Cl (20 mL) was added, and the aqueous layer was extracted with CH_2Cl_2 (3 × 50 mL). The combined organic extracts were dried over $MgSO_4$, filtered, and had the solvent removed *in vacuo*. The residue was purified by chromatography (petrol with 0→100% CH_2Cl_2, followed by 0→10% EtOH), to yield the product as a white foam (28.8 mg, 77%, 96.4% *ee*); ^{1}H NMR (400 MHz, $CDCl_3$, 298 K) δ 10.14 (s, 1H, H_h), 7.97 (br d, 2H, *J* = 1.5, H_i), 7.72 (app. t, 1H, *J* = 7.6, H_S), 7.71 (app. t, 1H, *J* = 7.8, H_B), 7.59 (d, 1H, *J* = 7.8, H_A), 7.55 (d, 1H, *J* = 7.6, H_T), 7.42 (d, 1H, *J* = 7.7, H_C), 7.32 (d, 1H, *J* = 7.8, H_R), 7.28 (d, 2H, *J* = 8.0, H_D), 7.20 (t, 1H, *J* = 1.5, H_j), 7.15-7.09 (m, 6H, H_f and H_g), 6.64 (d, 2H, J = 7.9, two of H_e), 7.56 (d, 2H, *J* = 7.9, two of H_e), 6.39 (d, 2H, *J* = 8.5, H_N), 6.23 (d, 2H, *J* = 8.8, H_M), 6.18 (d, 2H, *J* = 8.5, H_E), 4.79 (sept, 1H, *J* = 6.1, H_b), 4.55-4.43 (m, 1H, one of H_F), 4.09-4.02 (m, 1H, one of H_F), 4.00-3.88 (m, 2H, H_Q), 3.19 (d, 1H, *J* = 14.3, one of H_d), 3.15 (d, 1H, *J* = 14.3, one of H_d), 3.03 (d, 1H, *J* = 14.3, one of H_d), 2.94 (td, 1H, *J* = 13.7, 4.7, one of H_O), 2.83-2.71 (m, 2H, one of H_O and one of H_d), 2.58-2.47 (m, 1H, one of H_L), 2.48-2.33 (m, 2H, one of H_L and one of H_K), 2.20-2.03 (m, 3H, one of H_K and H_G), 2.03-1.58 (m, 8H, H_H, H_I, H_J, and H_P), 1.36-1.14 (bs, 18H, H_k), 1.04 (d, 3H, *J* = 6.1, three of H_a), 0.92 (d, 3H, *J* = 6.1, three of H_a); ^{13}C NMR (101 MHz, $CDCl_3$, 298 K) δ 168.6, 164.0, 159.0, 158.9, 157.9, 157.6, 157.2, 149.6, 147.2, 137.1, 137.0, 136.0, 135.6, 131.5, 131.5, 131.0, 130.8, 130.7, 128.7, 128.5, 127.8, 127.6, 126.5, 126.3, 124.0, 122.4, 120.6, 120.6, 120.1, 119.9, 119.3, 114.7, 114.5, 70.8, 69.6, 68.3, 66.3, 44.3, 41.8, 37.5, 35.4, 35.0, 31.7, 31.6, 31.1, 29.0, 28.6, 27.7, 25.9, 25.5, 21.5, 21.4.; LR-ESI-MS (+ve) *m/z* (%) = 1016.6 $[M+H]^+$ (100); HR-ESI-MS (+ve) *m/z* = 1016.6048 $[M+H]^+$ 1016.6048 ; Chiral SCFC (Chiralpak ID, 250 × 4.6 mm, 5 µm, 40 °C, MeOH (0.2% v/v NH_3)/CO_2 = 35%, 4 mL/min, λ = 210-400 nm) : tR [(R/S_{mp})-**5**] = 2.93 min, 3.69 min; tR [(S_{mp})-**5**] = 2.88 min.

‡ For comparison with rotaxane (S,S_{mp})-**4**, no proton is designated as H_c.

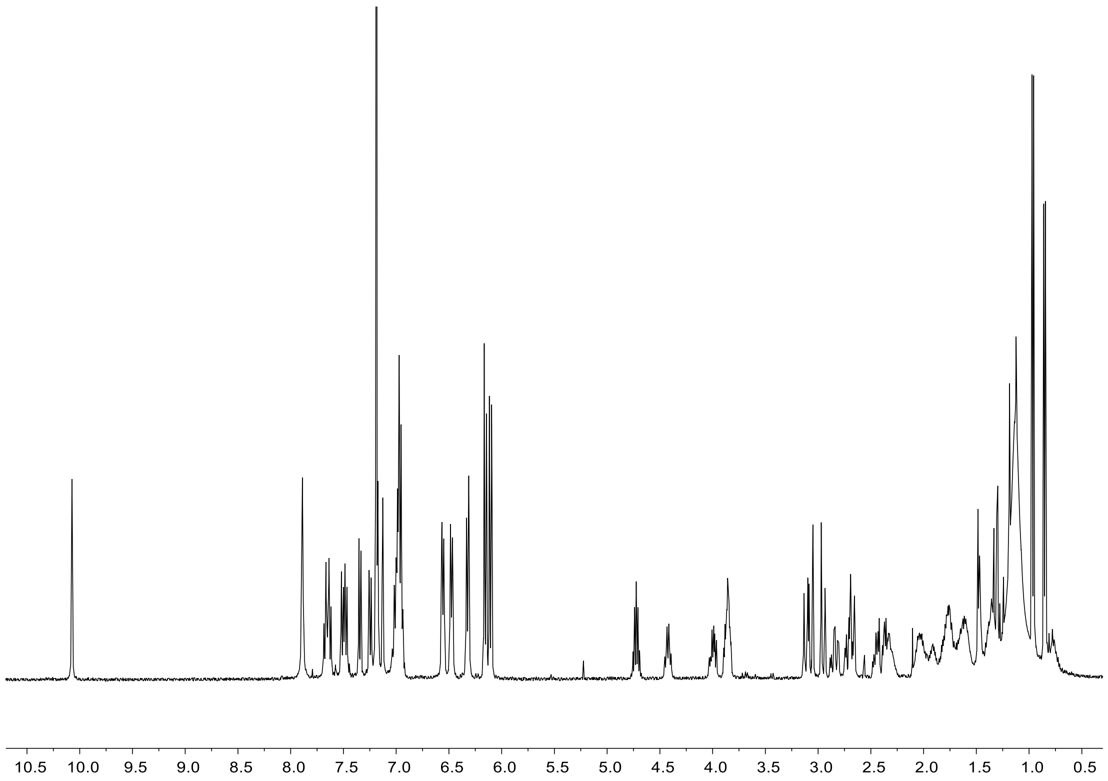

Figure S153. ^{1}H NMR (400 MHz, $CDCl_3$, 298 K) (S_{mp})-**5**

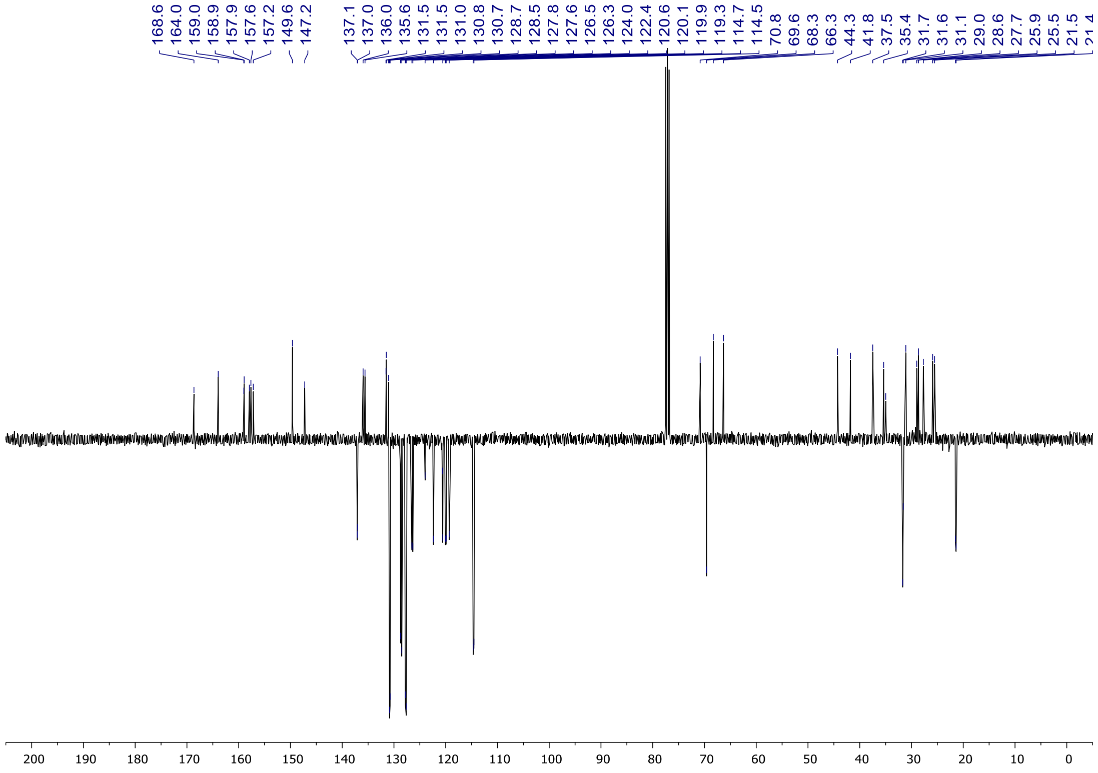

Figure S154. ^{13}C NMR (101 MHz, $CDCl_3$, 298 K) (S_{mp})-**5**.

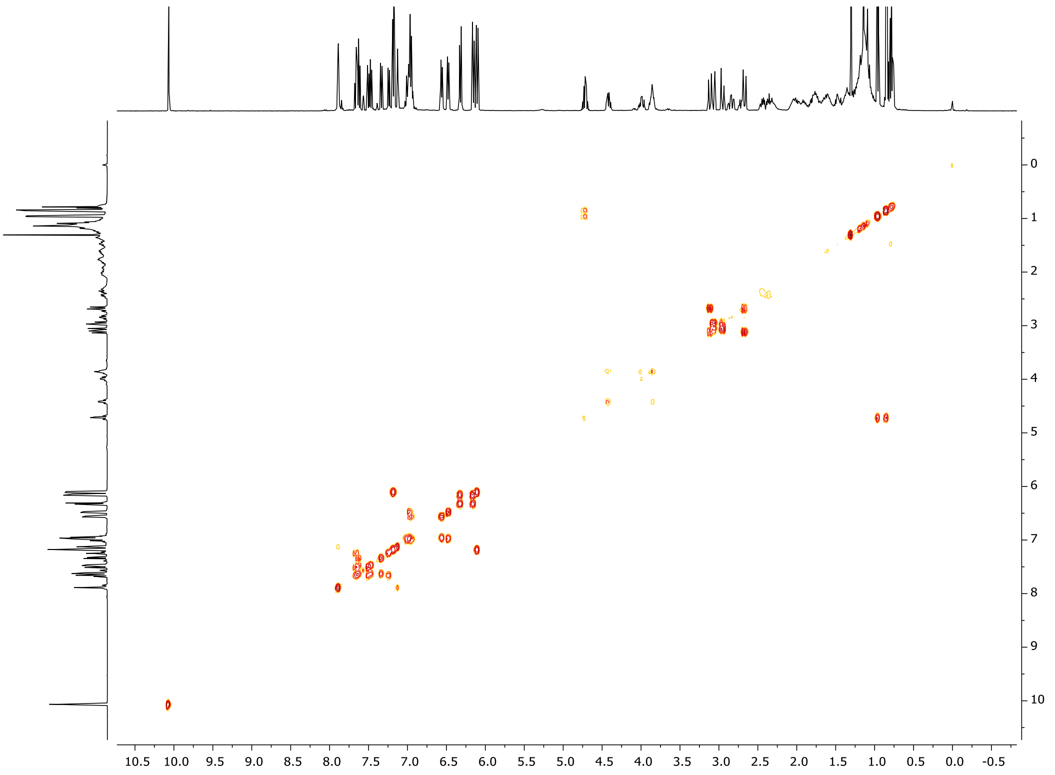

Figure S155. ^{1}H-^{1}H COSY NMR (400 MHz, $CDCl_3$, 298 K) (S_{mp})-**5**.

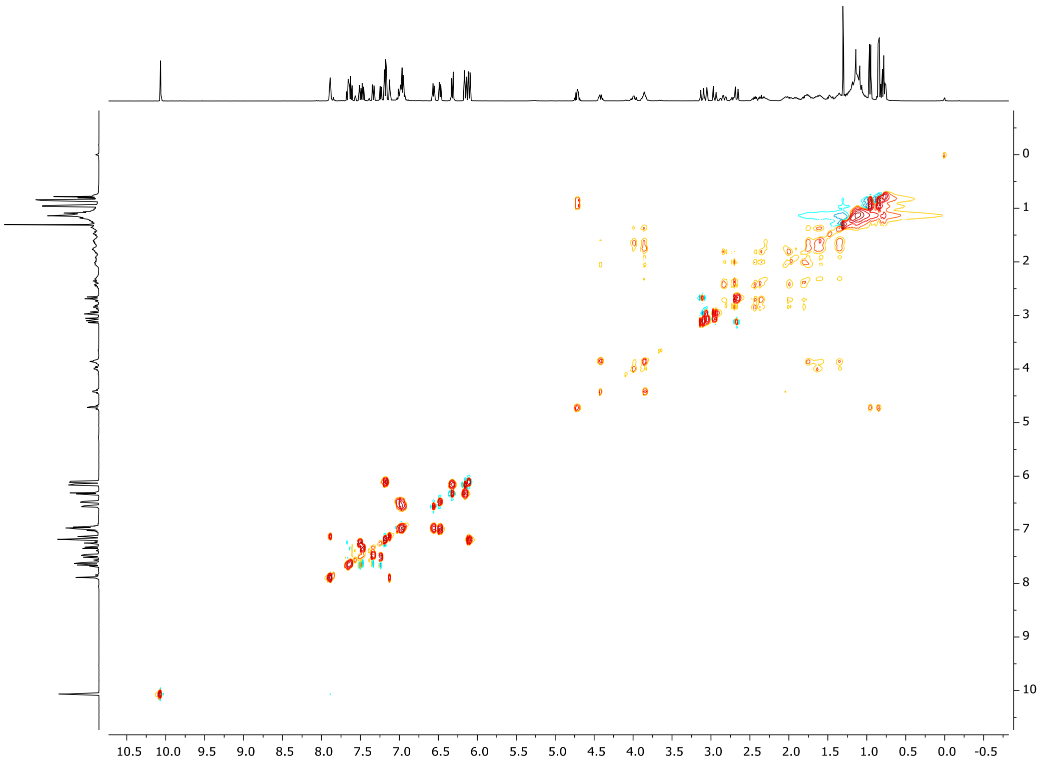

Figure S156. ^{1}H-^{1}H TOCSY NMR (400 MHz, $CDCl_3$, 298 K) (S_{mp})-**5**.

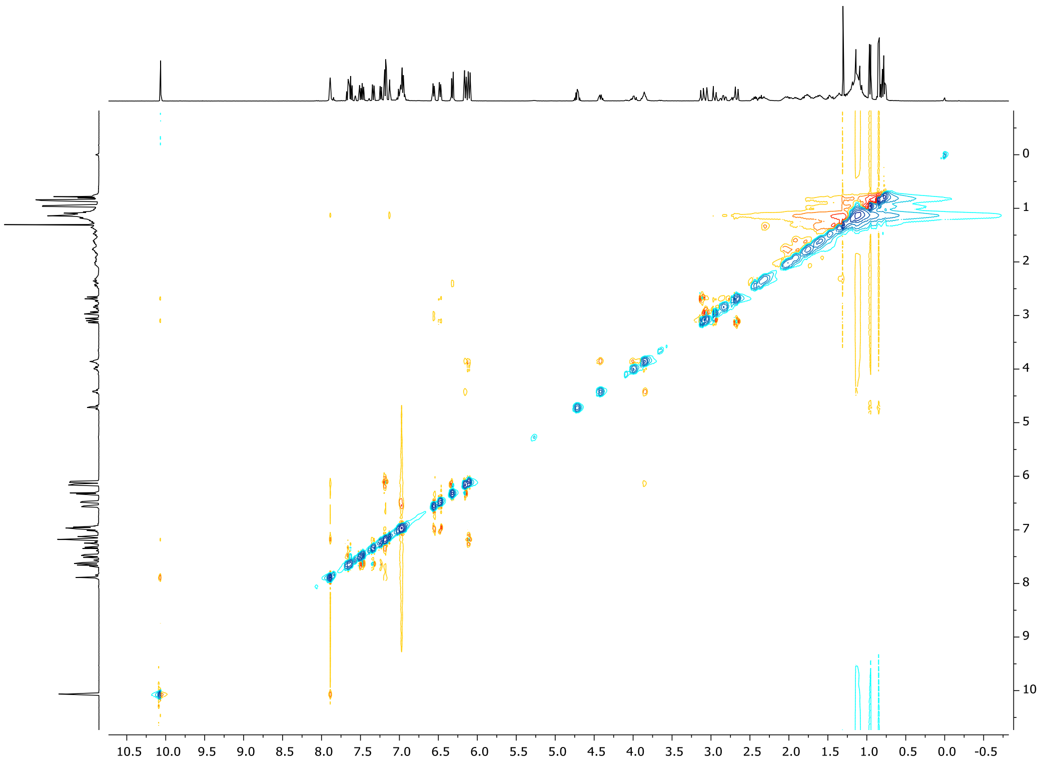

Figure S157. ^{1}H-^{1}H NOESY NMR (400 MHz, $CDCl_3$, 298 K) (S_{mp})-**5**.

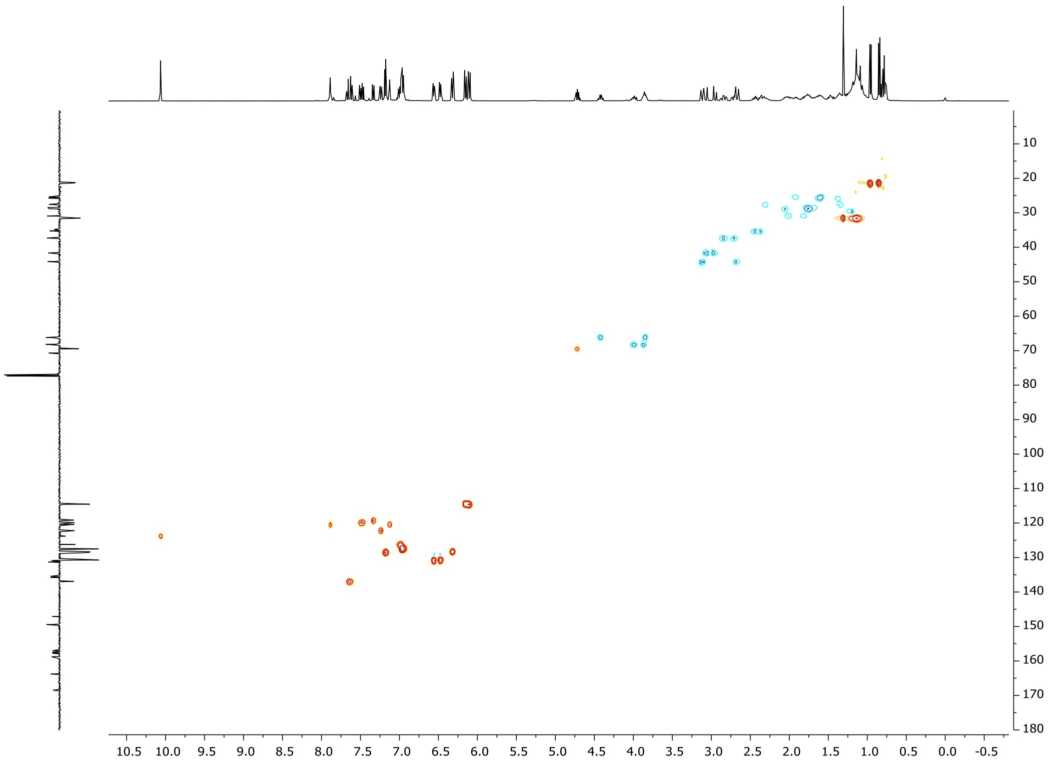

Figure S158. ^{1}H-^{13}C HSQC NMR (101 MHz, $CDCl_3$, 298 K) (S_{mp})-**5**.

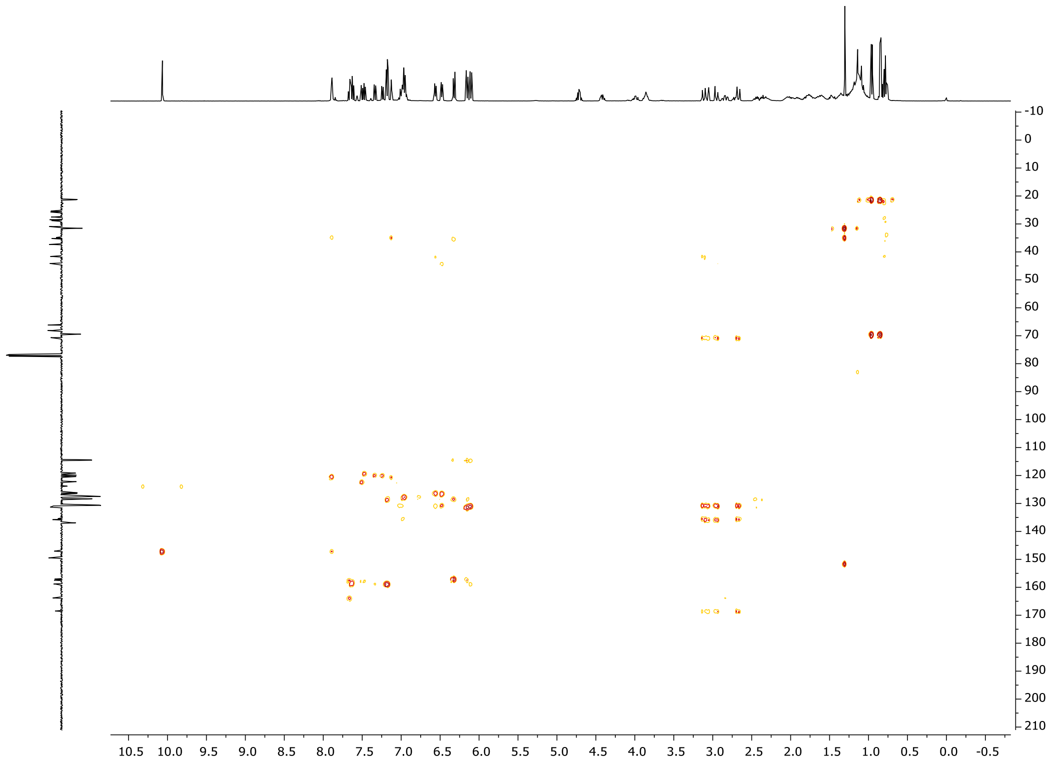

Figure S159. ^{1}H-^{13}C HMBC NMR (101 MHz, $CDCl_3$, 298 K) (S_{mp})-**5**.

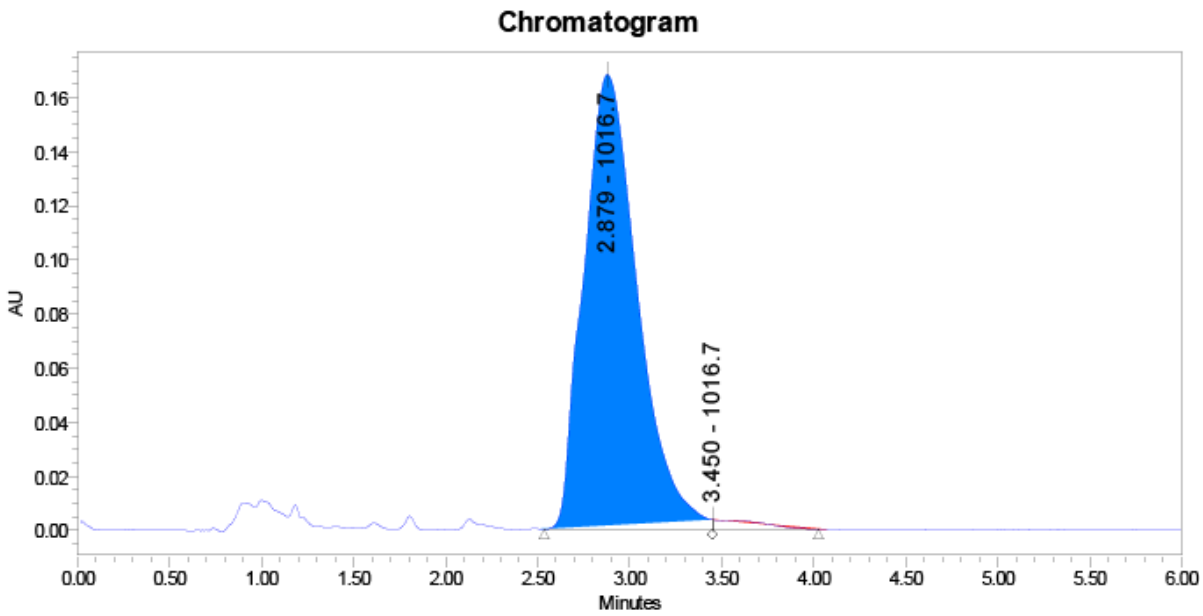

Peak Results

	Retention Time (min)	Area (µV*sec)	% Area	Width @ 50%
1	2.88	3422942	98.2	0.31333
2	3.45	63464	1.8	

Figure S160. Chiral SCFC chromatogram of (S_{mp})-**5**, following purification by chromatography.

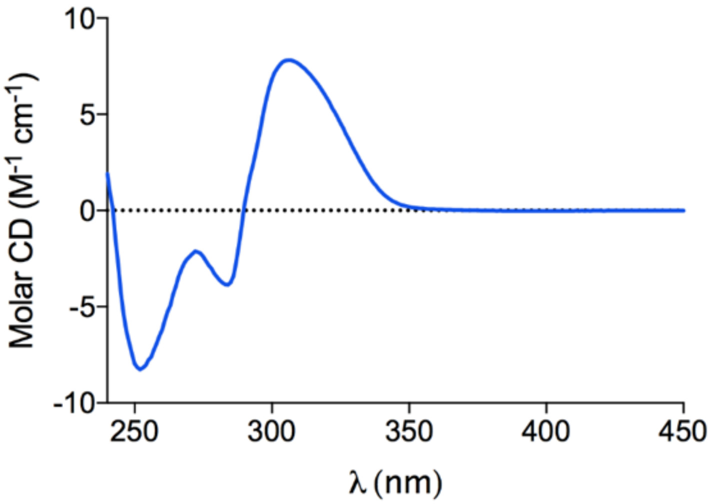

Figure S161. Circular dichroism spectrum of (S_{mp})-**5** (80.7 µM in $CHCl_3$).

Rotaxane (R_{mp})-**5**‡

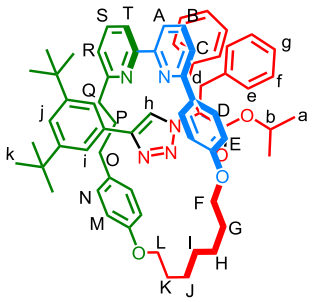

In a dry sealed vessel, (R,R_{mp})-**4** (23.5 mg, 0.0254 mmol), in LiHMDS (0.50 mL of a 1 M solution in THF, 0.50 mmol) was stirred at –78 °C for 15 minutes, protected by a nitrogen atmosphere. **S8** (30.1 mg, 0.140 mmol) in anhydrous THF (0.5 mL) was added and the reaction mixture was stirred, with warming to rt for two days. Saturated NH_4Cl (20 mL) was added, and the aqueous layer was extracted with CH_2Cl_2 (3 × 20 mL). The combined organic extracts were dried over $MgSO_4$, filtered, and had the solvent removed *in vacuo*. The residue was purified by chromatography (petrol with 0→100% CH_2Cl_2, followed by 0→10% EtOH), to yield (R_{mp})-**5** as a white foam, with identical spectra to (S_{mp})-**5**, with the exception of circular dichroism spectra (**Figure S163**) (21.4 mg, 82%, 96.0% ee); Chiral SCFC (Chiralpak ID, 250 × 4.6 mm, 5 µm, 40 °C, MeOH (0.2% v/v NH_3)/CO_2 = 5%, 4 mL/min, λ = 210-400 nm): tR [(R/S_{mp})-**5**] = 2.93 min, 3.69 min; tR [(R_{mp})-**5**] = 3.53 min.

‡ For comparison with rotaxane (R,R_{mp})-**4**, no proton is designated as H_c.

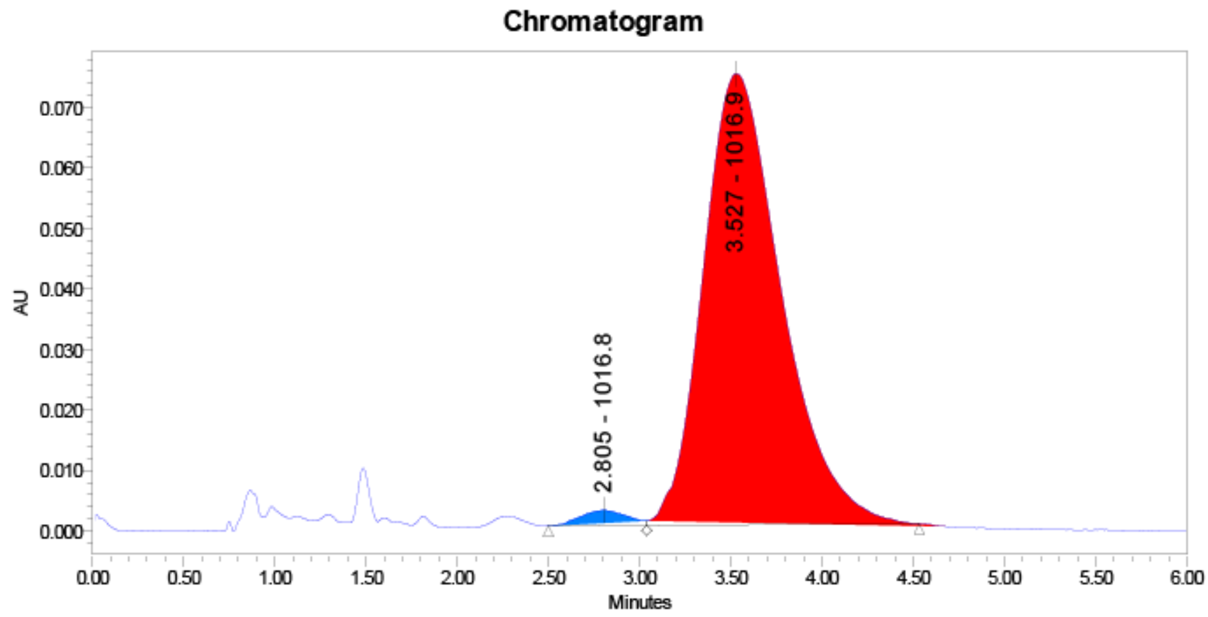

Peak Results

	Retention Time (min)	Area (µV*sec)	% Area	Width @ 50%
1	2.81	44619	2.0	0.29694
2	3.53	2224785	98.0	0.45267

Figure S162. Chiral SCFC chromatogram of (R_{mp})-**5**, following purification by chromatography.

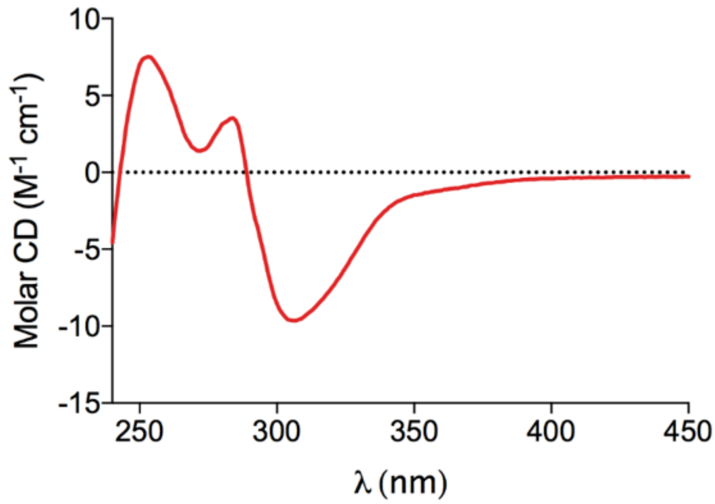

Figure S163. Circular dichroism spectrum of (R_{mp})-**5** (13.8 µM in $CHCl_3$).

Rotaxane (R/S_{mp})-**5**‡

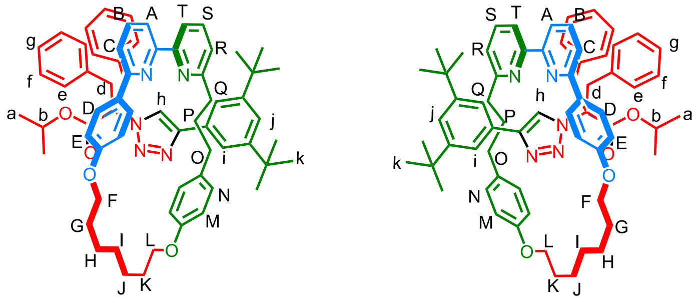

In a dry sealed vessel, (R*,R*$_{mp}$)-**4** (20.3 mg, 0.0219 mmol), in LiHMDS (0.40 mL of a 1 M solution in THF, 0.40 mmol) was stirred at –78 °C for 10 minutes, protected by a nitrogen atmosphere. **S8** (0.40 mL, of a 1 M solution in anhydrous THF, 0.40 mmol) was added and the reaction mixture was stirred, with warming to rt for 3 d. Saturated NH_4Cl (10 mL) was added, and the aqueous layer was extracted with CH_2Cl_2 (3 × 20 mL). The combined organic extracts were dried over $MgSO_4$, filtered,, and the solvent removed *in vacuo*. The residue was purified by chromatography (petrol with 0→100% CH_2Cl_2,followed by 0→10% EtOH), to yield (R/S_{mp})-**5** as a white foam with identical spectra to (R_{mp})-**5** and (S_{mp})-**5** (16.9 mg, 76%, 0.0% ee); Chiral SCFC (Chiralpak ID, 250 × 4.6 mm, 5 µm, 40 °C, MeOH (0.2% v/v NH_3)/CO_2 = 35%, 4 mL/min, λ = 210-400 nm): tR [(R/S_{mp})-**5**] = 2.93 min, 3.69 min.

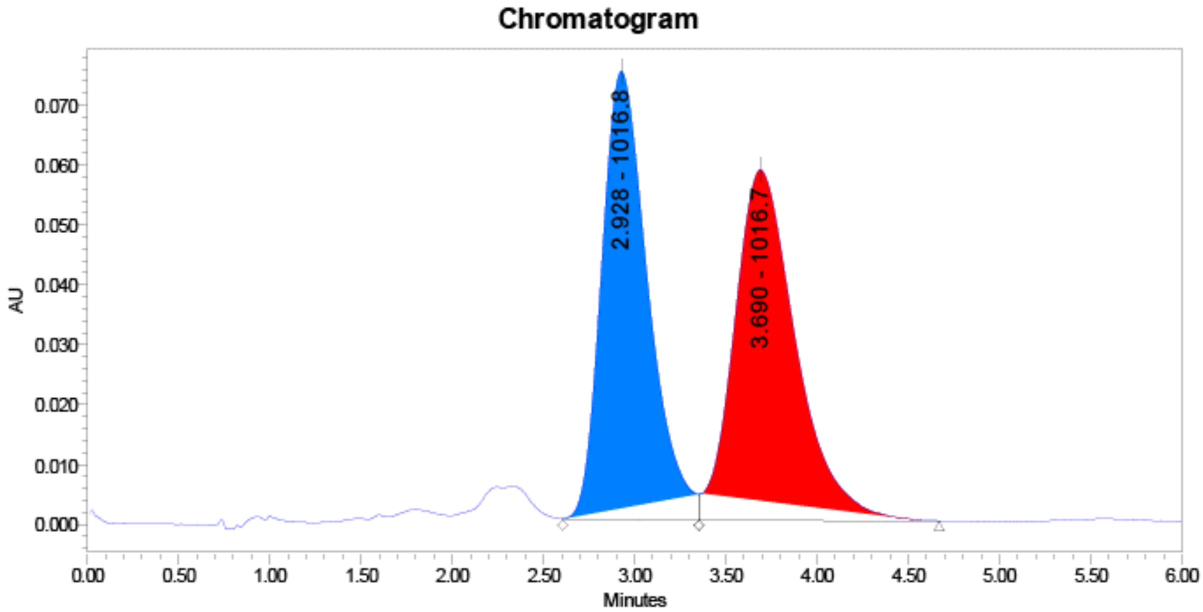

Peak Results

	Retention Time (min)	Area (µV*sec)	% Area	Width @ 50%
1	2.93	1327936	49.0	0.26972
2	3.69	1382473	51.0	0.35178

Figure S164. Chiral SCFC chromatogram of (R/S_{mp})-**5**, following purification by chromatography.

‡ For comparison with rotaxane (R*,R*$_{mp}$)-**4**, no proton is designated as H_c.

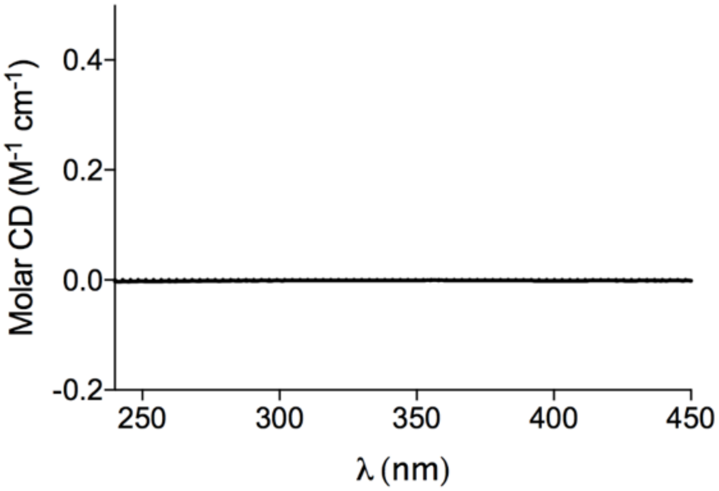

Figure S165. Circular dichroism spectrum of (R/S_{mp})-**5** (70.6 μM in $CHCl_3$).

5. Comparative ^{1}H-NMR stack plots of axles and rotaxanes

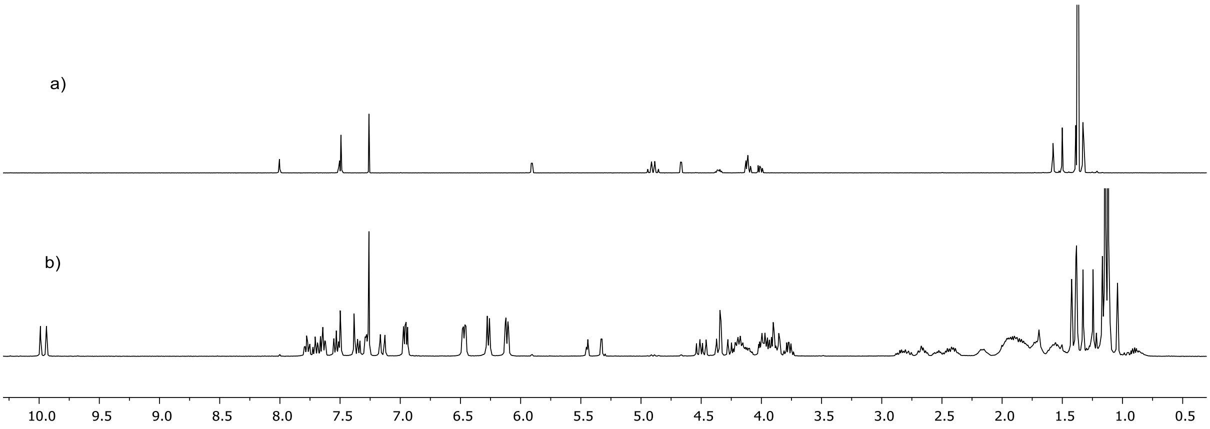

Figure S166. ^{1}H NMR (400 MHz, $CDCl_3$, 298 K); a) (D)-**S12** and b) (D,R_{mp}/S_{mp})-**S13**.

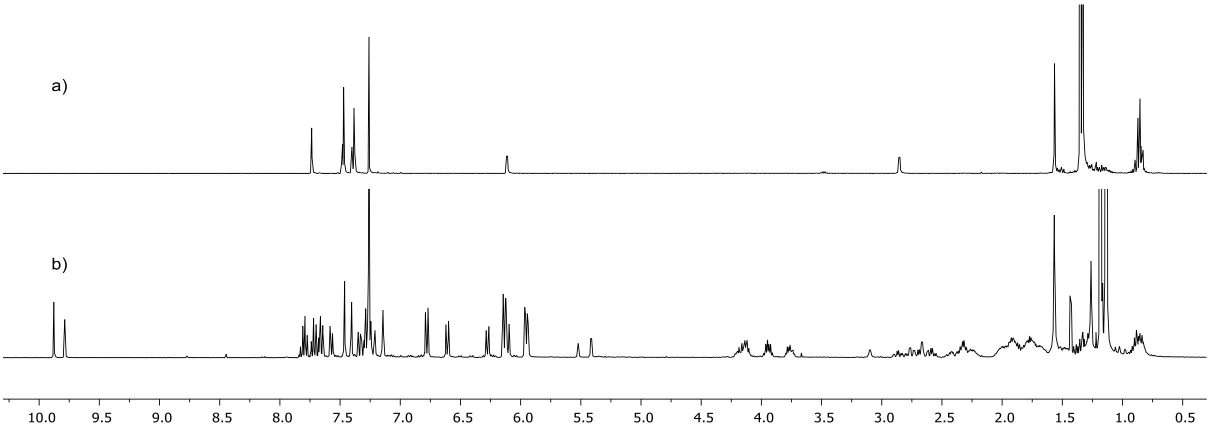

Figure S167. ^{1}H NMR (400 MHz, $CDCl_3$, 298 K); a) (*R*/*S*)-**S14** and b) (*R*/*S*,R_{mp}/S_{mp})-**S15**.

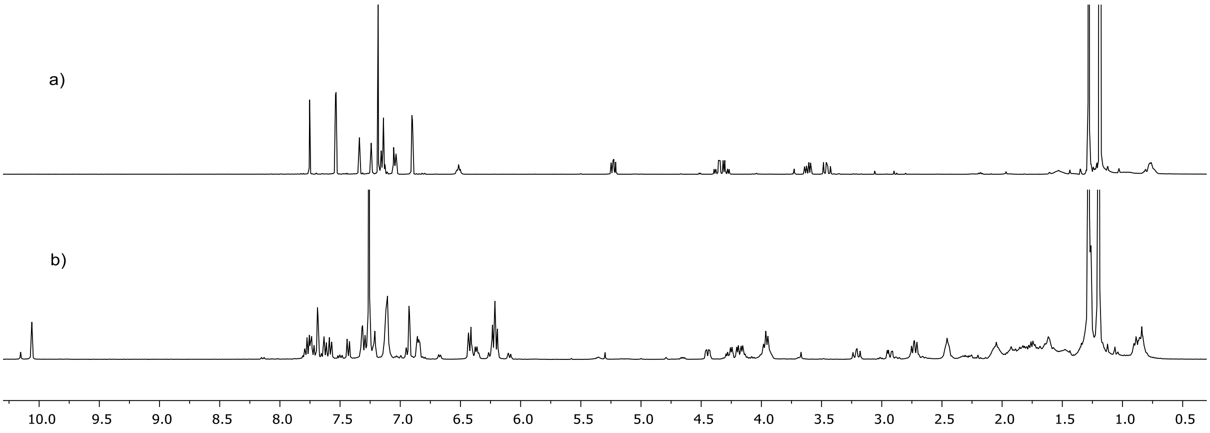

Figure S168. ^{1}H NMR (400 MHz, $CDCl_3$, 298 K); a) (*S*)-**S16** and b) (*S*,R_{mp}/S_{mp})-**S17**.

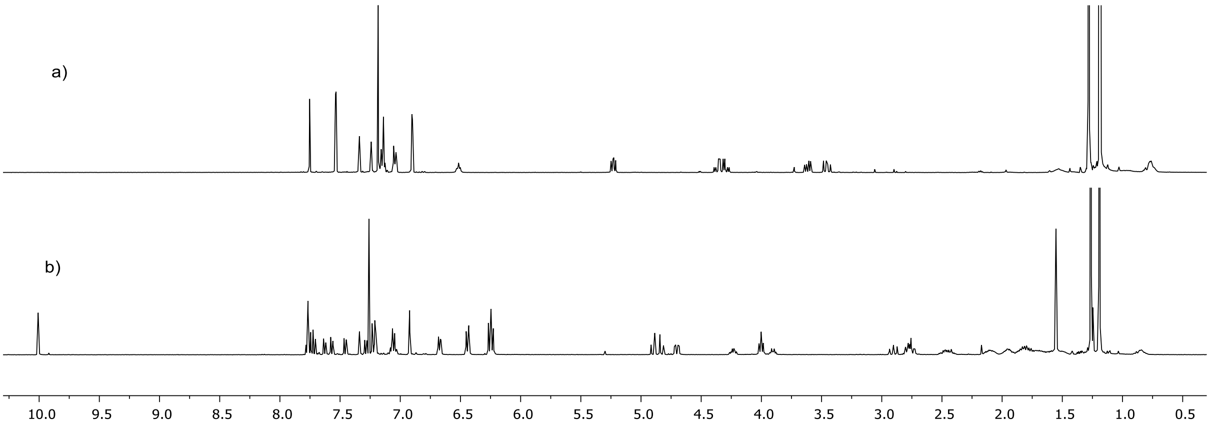

Figure S169. ^{1}H NMR (400 MHz, $CDCl_3$, 298 K); a) (*S*)-**S18** and b) (*S*,R_{mp}/S_{mp})-**S19**.

The ^{1}H NMR spectrum of (*S*,S_{mp})-**4** contains the expected features of such a crowded interlocked structure; many signals of the macrocycle and axle shift to lower ppm compared with the non-interlocked components (Figure 170) including, perhaps surprisingly, H_e. In contrast, H_h appears at higher ppm consistent with the C-H•••N H-bonding contact observed in the solid-state structure. Diastereotopic protons H_d, which are isochronous in the non-interlocked axle, appear separated at lower ppm in rotaxane (*S*,S_{mp})-**4**. A similar effect is observed in the case of diastereotopic ester protons H_a.

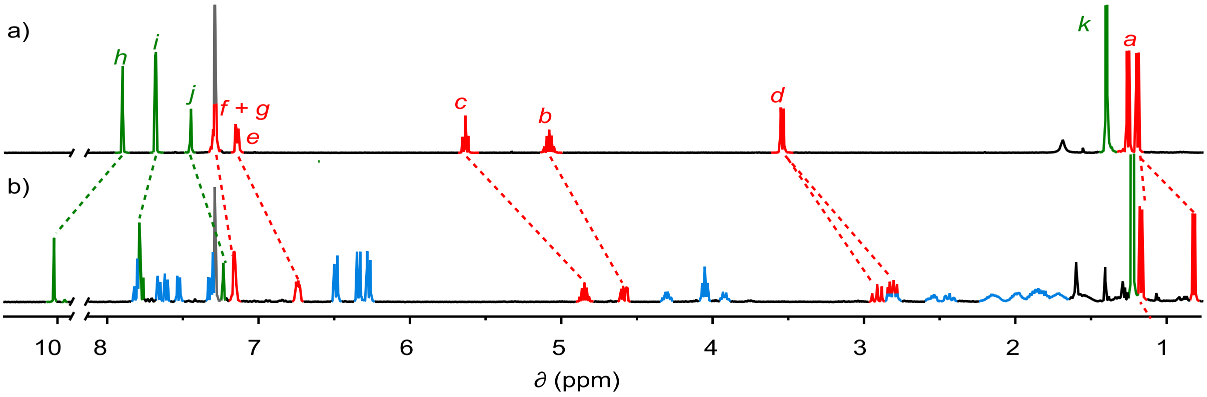

Figure S170. ^{1}H NMR (400 MHz, $CDCl_3$, 298 K); a) (*S*)-**S20** and b) (*S*,S_{mp})-**4**.

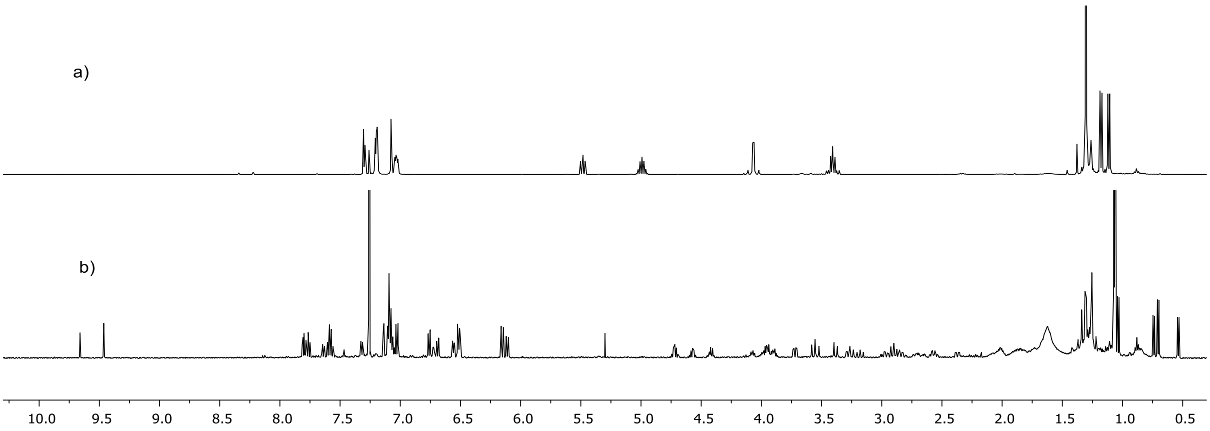

Figure S171. ^{1}H NMR (400 MHz, $CDCl_3$, 298 K); a) (*S*)-**S21** and b) (*S*,R_{mp}/S_{mp})-**S22**.

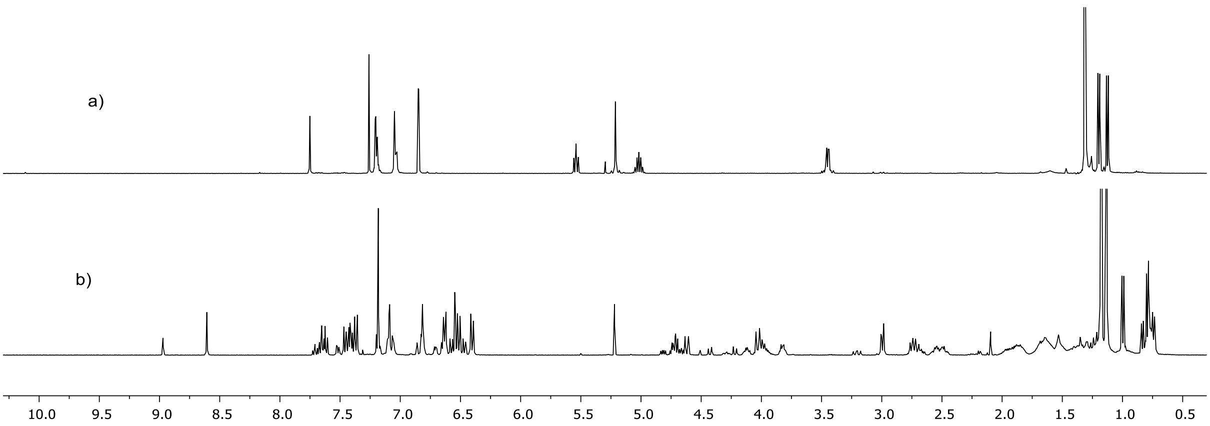

Figure S172. ^{1}H NMR (400 MHz, $CDCl_3$, 298 K); a) (*S*)-**S23** and b) (*S*,R_{mp}/S_{mp})-**S24**.

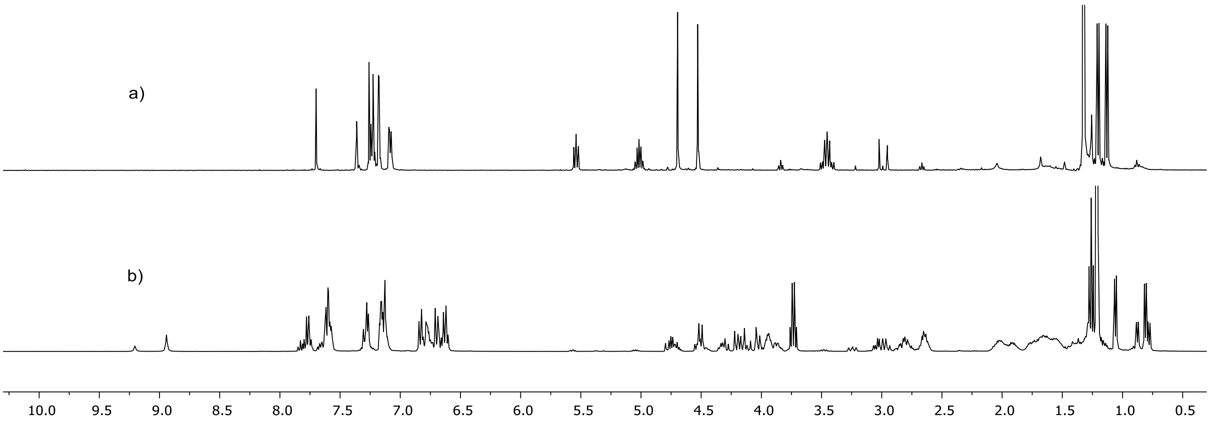

Figure S173. ^{1}H NMR (400 MHz, $CDCl_3$, 298 K); a) (*S*)-**S25** and b) (*S*,R_{mp}/S_{mp})-**S26**.

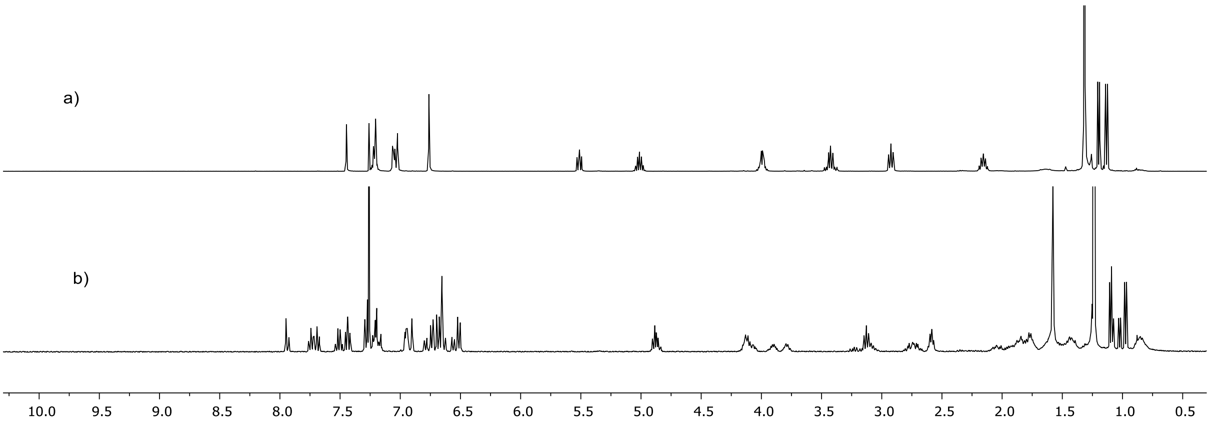

Figure S174. ^{1}H NMR (400 MHz, $CDCl_3$, 298 K); a) (*S*)-**S27** and b) (*S*,R_{mp}/S_{mp})-**S28**.

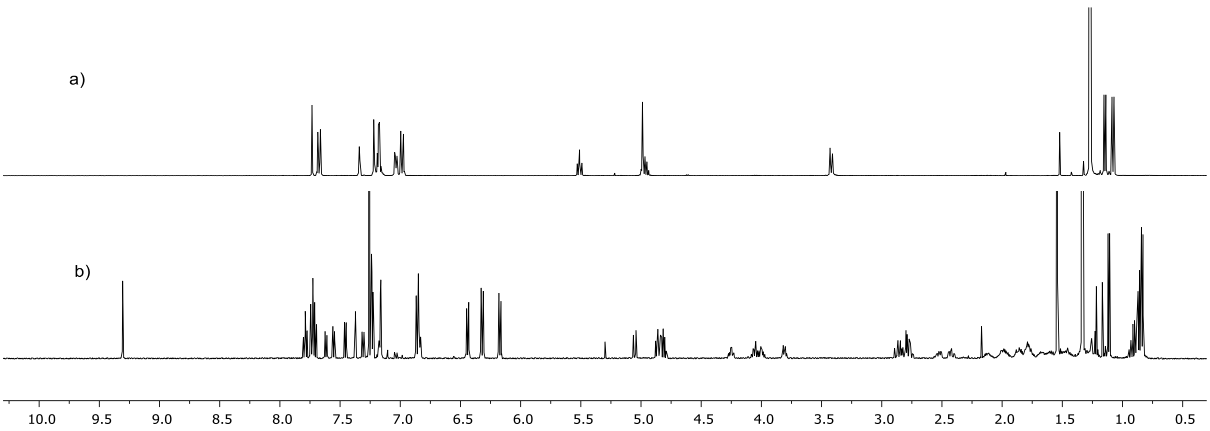

Figure S175. ^{1}H NMR (400 MHz, $CDCl_3$, 298 K); a) (*S*)-**S29** and b) **S30**.

Comparison of the ^{1}H NMR spectrum of rotaxane (S_{mp})-**5** with that of the corresponding axle (Figure S176) highlights the stereogenic nature of the mechanical bond. Protons H_a are diastereotopic in rotaxane (S_{mp})-**5** and appear as two doublets whereas they are enantiotopic in the corresponding axle and thus appear as a single resonance. Similarly, H_d, which appear as two enantiotopic environments in the non-interlocked axle, produce four signals in rotaxane (S_{mp})-**5** and phenyl protons H_e appear as two diastereotopic sets in rotaxane (S_{mp})-**5**.

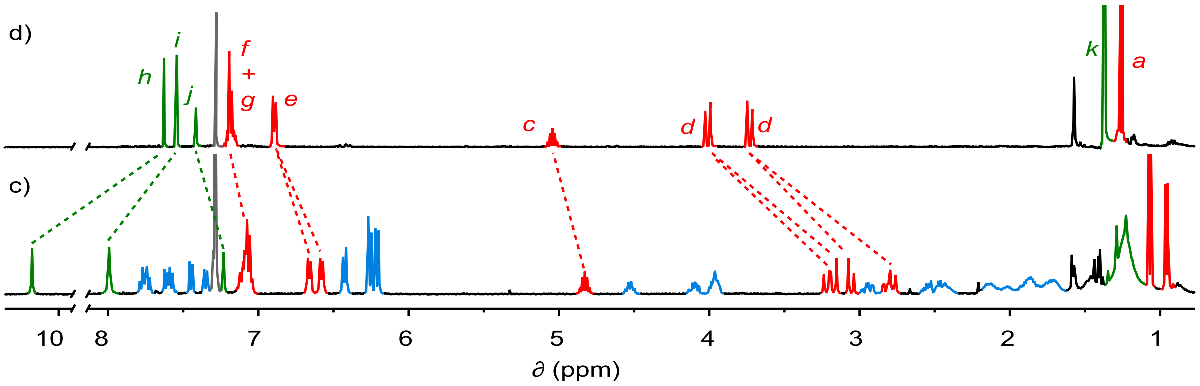

Figure S176. ^{1}H NMR (400 MHz, $CDCl_3$, 298 K); a) **S31** and b) (S_{mp})-**5**.

6. Comparative circular dichroism spectra

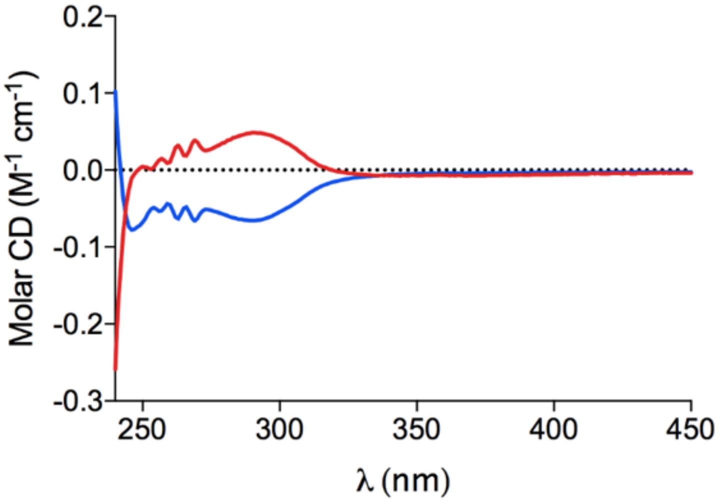

Figure S177. Circular dichroism spectrum ($CHCl_3$, 298 K); a) (*S*)-**3e** (blue) and b) (*R*)-**3e** (red).

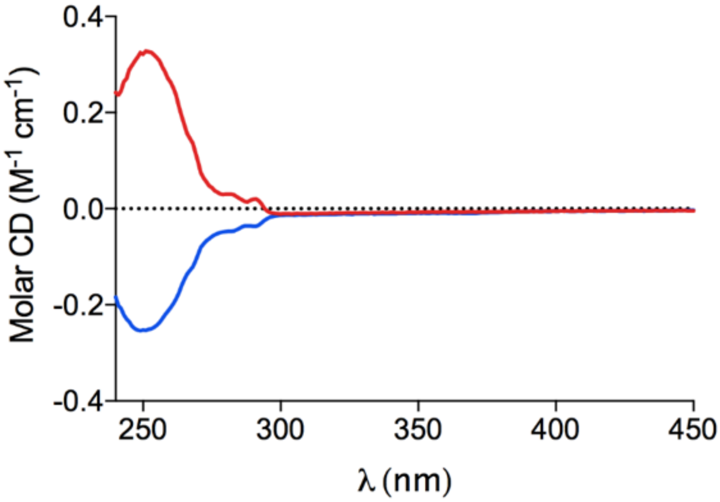

Figure S178. Circular dichroism spectrum ($CHCl_3$, 298 K); a) (*S*)-**S20** (blue) and b) (*R*)-**S20** (red).

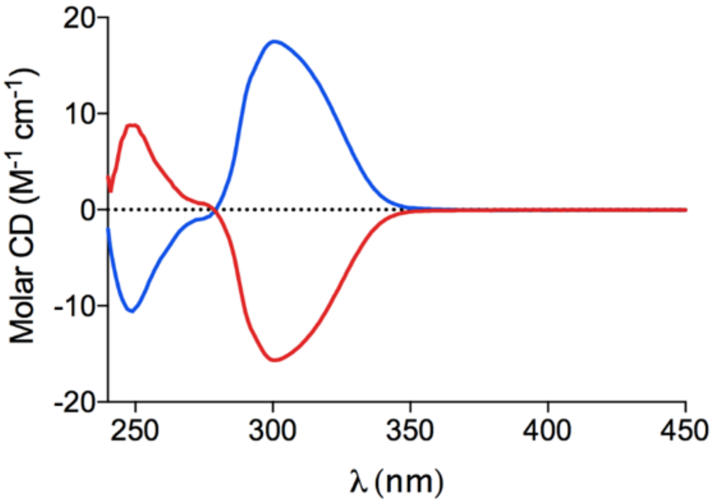

Figure S179. Circular dichroism spectrum ($CHCl_3$, 298 K); a) (*S*,*S*$_{mp}$)-**4** (blue) and b) (*R*,*R*$_{mp}$)-**4** (red).

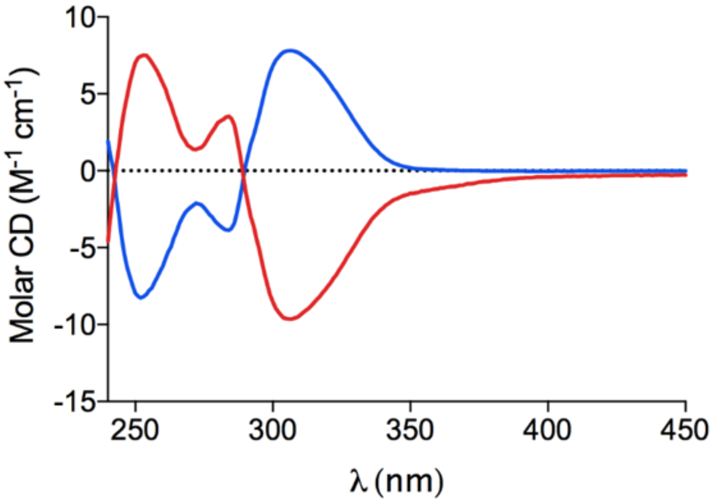

Figure S180. Circular dichroism spectrum ($CHCl_3$, 298 K); a) (*S*$_{mp}$)-**5** (blue) and b) (*R*$_{mp}$)-**5** (red).

7. Single crystal X-ray crystallographic data of (*S*,*S*$_{mp}$)-4

Single crystals of (*S*,*S*$_{mp}$)-**4** were grown by slow evaporation of 11 Petrol-$CHCl_3$. Data was collected at 100 K using a Rigaku 007 HF diffractometer equipped with a Saturn724+ enhanced sensitivity detector. Cell determination, data collection, data reduction, cell refinement and absorption correction were performed with CrysalisPro. Using Olex2 the structure was solved with the SHELXT program using charge flipping,[18] and refined with the SHELXL refinement package.[18] H atoms were placed in calculated positions and refined using a riding model.

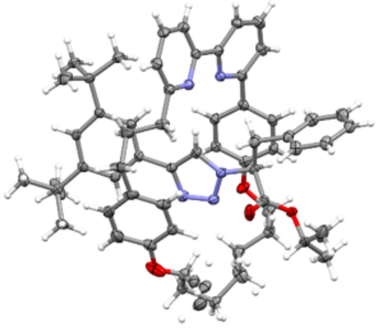

Figure S181. Single crystal X-ray structure of (*S*,*S*$_{mp}$)-**4** . Ellipsoids shown at 50% probability

Table S1. Crystal data and structure refinement for (*S*,*S*$_{mp}$)-**4**

Identification code	**4a**
Empirical formula	$C_{60}H_{71}N_5O_4$
Formula weight	926.21
Temperature/K	100(2)
Crystal system	orthorhombic
Space group	$P2_12_12_1$
a/Å	10.8301(2)
b/Å	21.0624(5)
c/Å	23.0291(5)
α/°	90
β/°	90
γ/°	90
Volume/Å^3	5253.12(19)
Z	4
ρ_{calc}g/cm^3	1.171
μ/mm^{-1}	0.073
F(000)	1992.0
Crystal size/mm^3	0.12 × 0.03 × 0.03
Radiation	MoKα (λ = 0.71073)
2Θ range for data collection/°	6.066 to 61.408
Index ranges	-13 ≤ h ≤ 15, -28 ≤ k ≤ 14, -33 ≤ l ≤ 27
Reflections collected	25297
Independent reflections	13684 [R_{int} = 0.0280, R_{sigma} = 0.0571]
Data/restraints/parameters	13684/513/658
Goodness-of-fit on F^2	0.920
Final R indexes [I>=2σ (I)]	R_1 = 0.0567, wR_2 = 0.1162
Final R indexes [all data]	R_1 = 0.0714, wR_2 = 0.1247
Largest diff. peak/hole / e Å^{-3}	0.23/-0.19

8. Synthesis of enantiopure rotaxane (R_{mp})-5

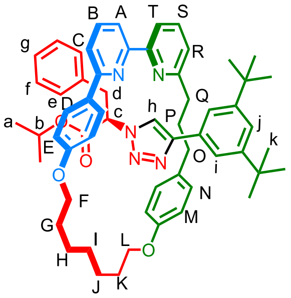

A dry sealed vessel was charged with **2a** (52.0 mg, 0.243 mmol), (*R*)-**3e** (54.2 mg, 0.243 mmol), macrocycle **1** (58.2 mg, 0.121 mmol), $[Cu(MeCN)_4]PF_6$ (43.5 mg, 0.116 mmol), DIPEA (85 µL, 0.486 mmol), and anhydrous CH_2Cl_2 (3 mL). The reaction mixture was stirred at rt for 16 h, protected by an argon atmosphere. TFA (0.10 mL, 1.31 mmol) was added and the reaction mixture was stirred for an additional 16 h. Saturated EDTA-NH_3 solution (10 mL) was added, and the aqueous layer was extracted with CH_2Cl_2 (3 × 20 mL), dried over $MgSO_4$, filtered,, and had the solvent. The residue was purified by chromatography (petrol with 0→100% CH_2Cl_2, followed by 0→10% EtOH), to yield the product as a white foam, with identical spectra to (R,R_{mp})-**4** but as a single diastereoisomer (64.1 mg, 55%, >991 diastereoisomeric ratio **Figure S182**, >99.9% ee); Chiral SCFC (LUX C1, 250 × 4.6 mm, 5 µm, 40 °C, MeOH (0.2% v/v NH_3)/CO_2 = 10→50%, 4 mL/min, λ = 210-400 nm): tR [(R/S,R_{mp}/S_{mp})-**4**] = 4.67 min, 5.00 min; tR [(S,S_{mp})-**4**] = 4.98.

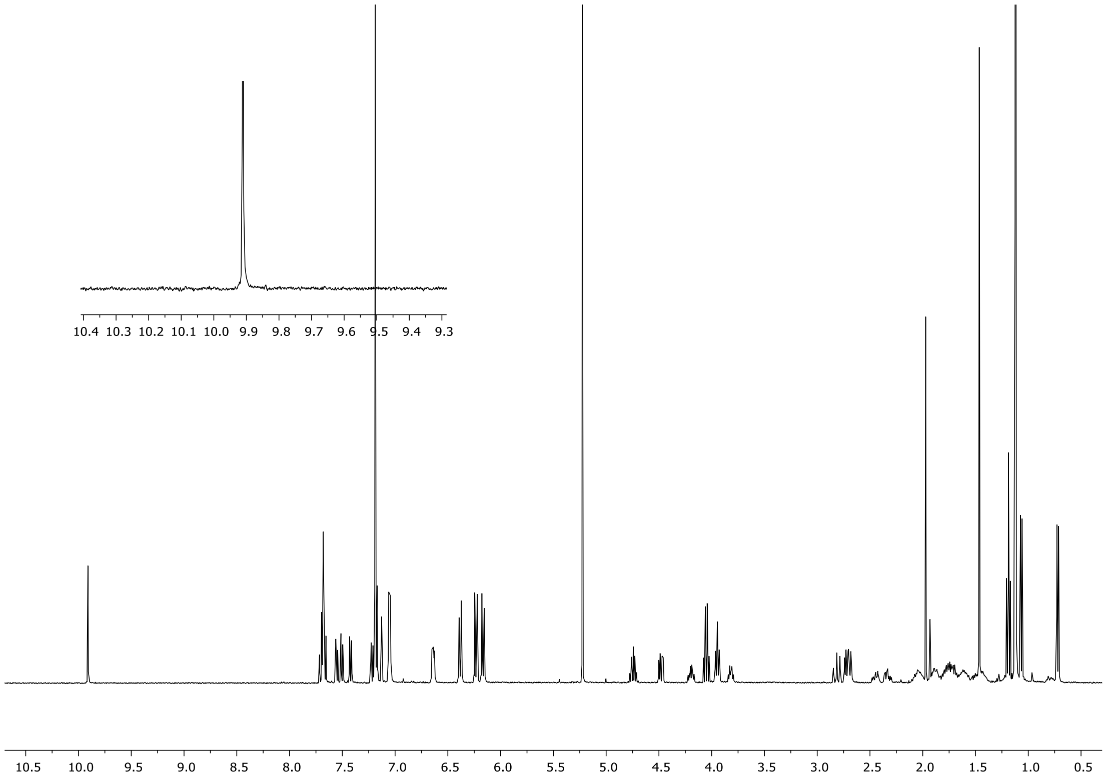

Figure S182. ^1H NMR (400 MHz, $CDCl_3$, 298 K) (R,R_{mp})-**4**, following purification by chromatography.

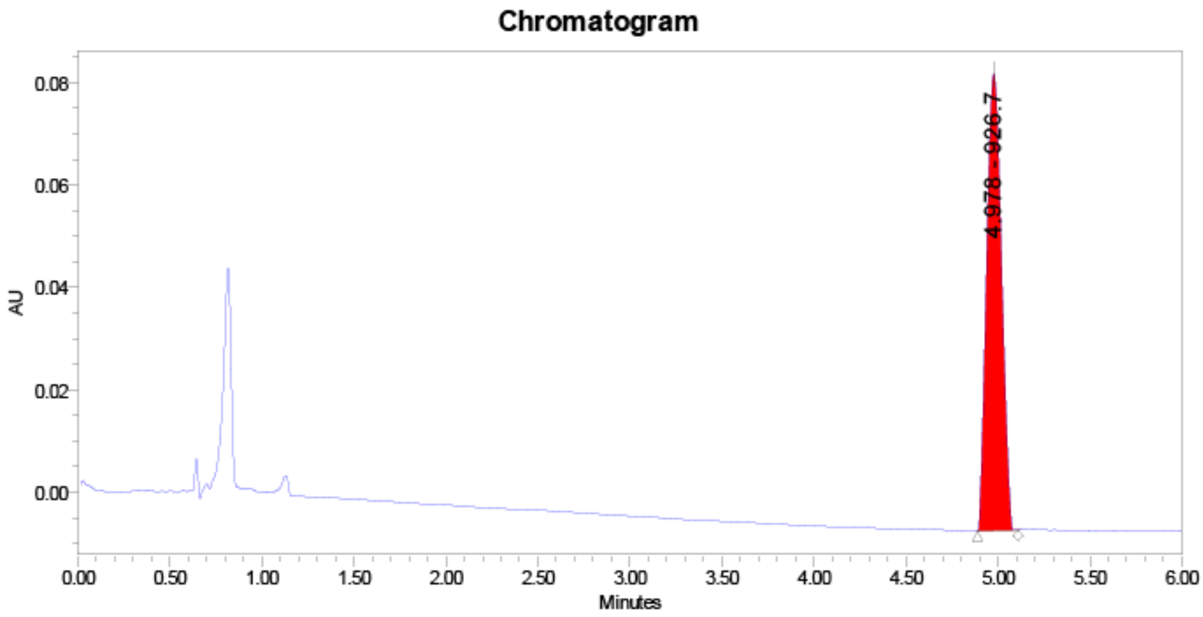

Peak Results

	Retention Time (min)	Area (μV*sec)	% Area	Width @ 50%
1	4.98	501681	100.0	0.09228

Figure S183. Chiral SCFC chromatogram of a single diastereoisomer of (*R*,R_{mp})-**4**, following purification by chromatography.

‡

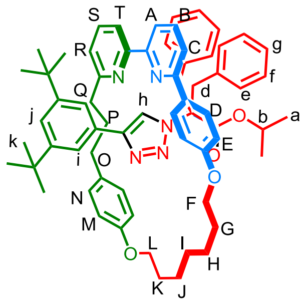

In a dry sealed vessel, (*R*,R_{mp})-**4** (21.7 mg, 0.00230 mmol), in LiHMDS (0.14 mL of a 1 M solution in THF, 0.14 mmol) was stirred at -78 °C for 15 minutes, protected by a nitrogen atmosphere. **S8** (30.1 mg, 0.140 mmol) in anhydrous THF (0.14 mL) was added and the reaction mixture was stirred, with warming to rt for 48 h. Saturated NH_4Cl (20 mL) was added, and the aqueous layer was extracted with CH_2Cl_2 (3 × 20 mL). The combined organic extracts were dried over $MgSO_4$, filtered, and the solvent removed *in vacuo*. Th residue was purified by chromatography (petrol with 0→100% CH_2Cl_2, followed by 0→10% EtOH), to yield the product as a white foam, with identical spectra to **5a** (23.5 mg, 62%, >99% ee); Chiral SCFC (Chiralpak ID, 250 × 4.6 mm, 5 μm, 40 °C, MeOH (0.2% v/v NH_3)/CO_2 = 35%, 4 mL/min, λ = 210-400 nm): tR [(*R*/S_{mp})-**5**] = 2.93 min, 3.69 min; tR (R_{mp})-**5**] = 3.73 min.

‡ For comparison with rotaxane (*R*,R_{mp})-**4**, no proton is designated as H_c.

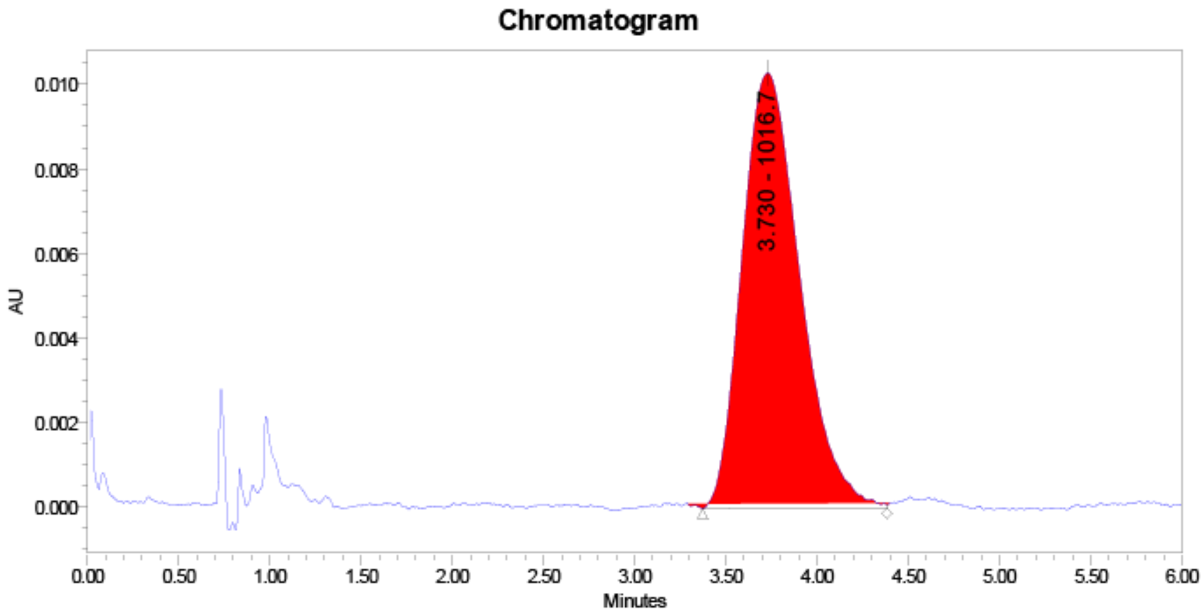

Peak Results

	Retention Time (min)	Area (µV*sec)	% Area	Width @ 50%
1	3.73	227883	100.0	0.34383

Figure S184. Chiral SCFC chromatogram of enantiomerically pure (R_{mp})-**5**, following purification by chromatography.

9. Racemisation of axle (*S*)-S20 under CuAAc conditions

In the synthesis of axle (*S*)-**S20**, partial racemization was observed. A small screen was performed to assess the effect of reaction conditions on the racemisation process. Strikingly, although no racemisation was observed in the AT-CuAAC synthesis of rotaxanes **4** or **5**, when a macrocycle **S9**[3] that is too large to be retained by the stoppers is employed (entry c), the racemisation process is exacerbated. Thus, it appears that the racemisation of the stereocentre α-to the ester is inhibited in rotaxane **4**, presumably due to the steric hindrance provided by the mechanical bond.

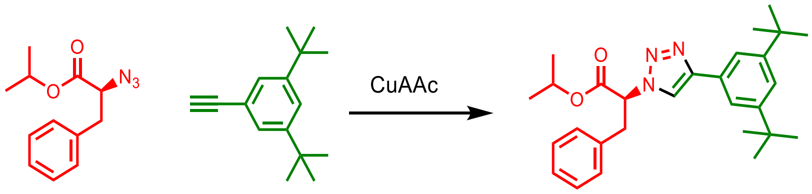

Table S2. Reaction condition screening for the synthesis of (*S*)-**S20**.[a]

Entry	Catalytic Mixture	Additive	ee (%)[b]
a	$CuSO_4.5H_2O$/ Sodium-*L*-ascorbate	None	95.6
b	$[Cu(MeCN)_4]PF_6$	4 eq, DIPEA	94.8
c	$[Cu(MeCN)_4]PF_6$	4 eq DIPEA, 0.5 eq. 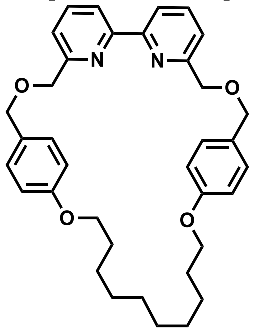	92.8

[a]0.5 eq catalytic mixture, 0.04 M CH_2Cl_2, rt, 16 h, under a nitrogen atmosphere; [b] determined by Chiral SCFC (Chiralpak IG, 250 × 4.6 mm, 5 µm, 40 °C, EtOH (0.2% v/v NH_3)/CO_2 = 1585, 4 mL/min, λ = 210-400 nm): tR [(*R*/*S*)-**S20**] = 1.69 min, 2.02 min; tR [(*S*)-**S20**] = 1.70.

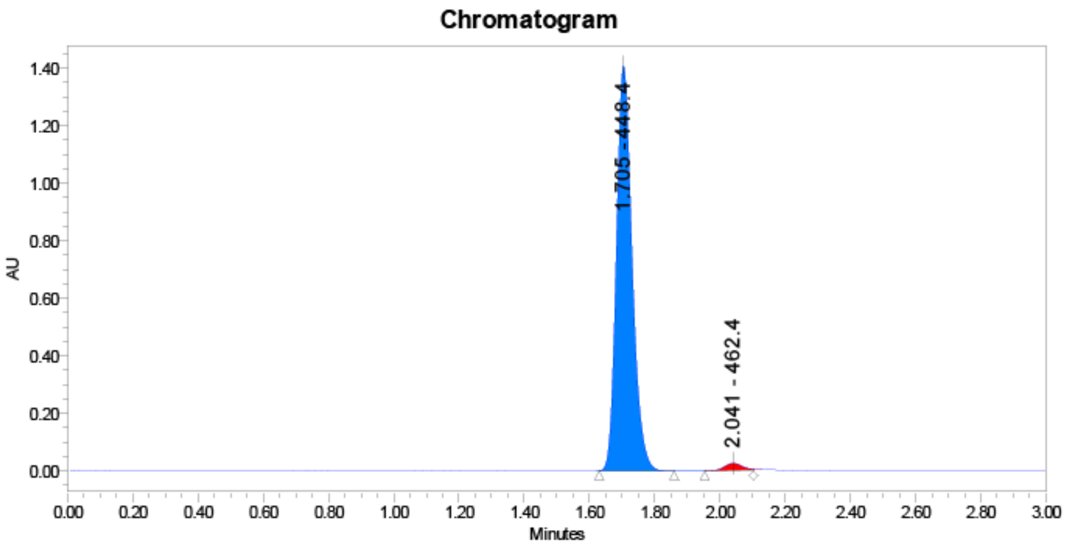

Peak Results

	Retention Time (min)	Area (µV*sec)	% Area	Width @ 50%
1	1.70	4675680	97.8	0.05062
2	2.04	106648	2.2	0.06229

Figure S185. Chiral SCFC chromatogram of **Table S2, entry a**, following purification by chromatography.

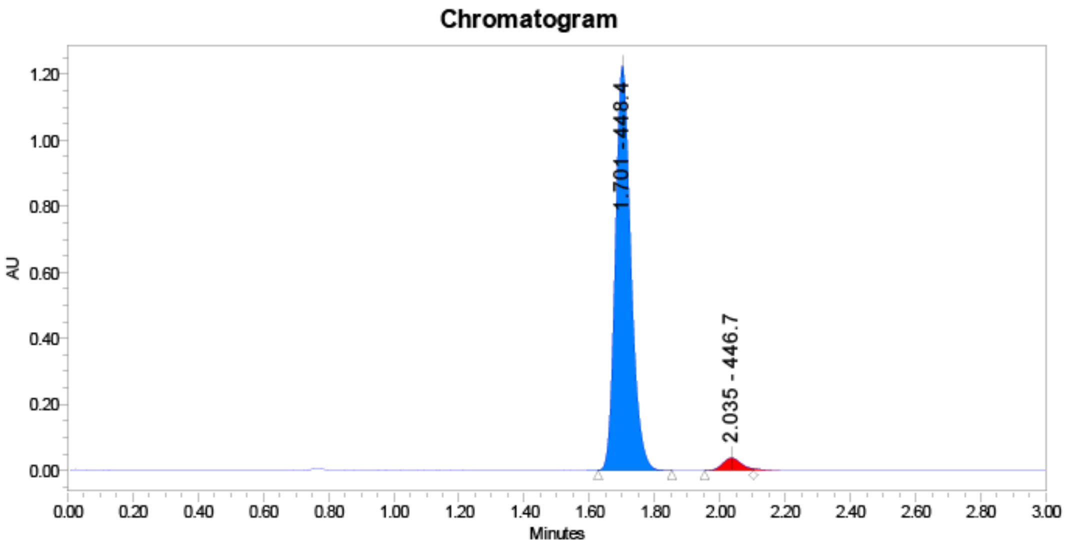

Peak Results

	Retention Time (min)	Area (µV*sec)	% Area	Width @ 50%
1	1.70	4056955	96.4	0.05036
2	2.04	150377	3.6	0.06123

Figure S186. Chiral SCFC chromatogram of **Table S2, entry b**, following purification by chromatography.

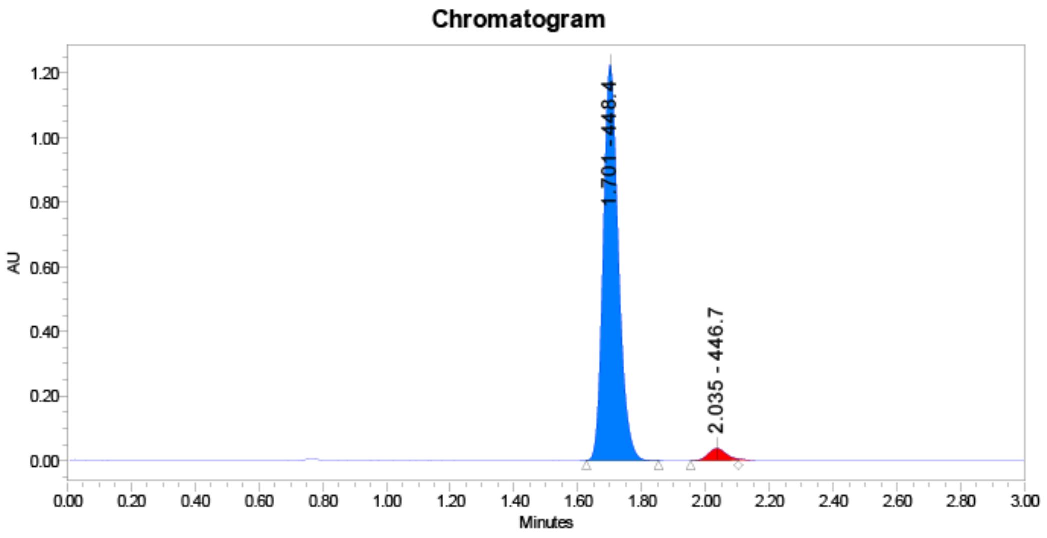

Peak Results

	Retention Time (min)	Area (µV*sec)	% Area	Width @ 50%
1	1.70	4056955	96.4	0.05036
2	2.04	150377	3.6	0.06123

Figure S187. Chiral SCFC chromatogram of **Table S2, entry c**, following purification by chromatography.

10. Diastereoselective Alkylation of Rotaxanes (*S,S$_{mp}$*)-4

Rotaxanes (*R/S,S$_{mp}$*)-**S33**‡

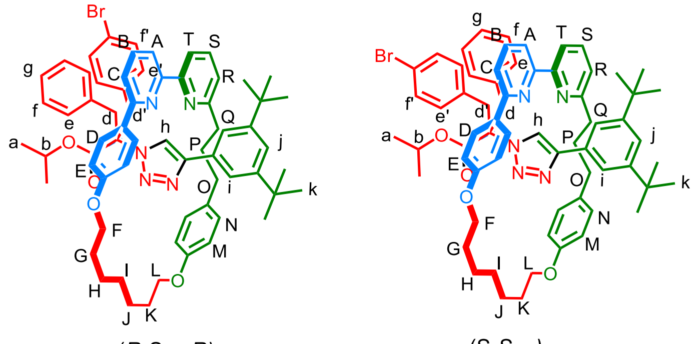

In a dry sealed vessel, (*S,S$_{mp}$*)-**4** (13.1 mg, 0.0141 mmol), in LiHMDS (0.20 mL of a 1 M solution in THF, 0.20 mmol) was stirred at −78 °C for 10 min, protected by a nitrogen atmosphere. **S32** (23.2 mg, 0.0882 mmol) in anhydrous THF (0.2 mL) and was added and the reaction mixture was stirred, with warming to rt for two days. Saturated NH_4Cl (10 mL) was added, and the aqueous layer was extracted with CH_2Cl_2 (3 × 30 mL). The combined organic extracts were dried over $MgSO_4$, filtered, and had the solvent removed *in vacuo*. The residue containing rotaxanes ((*R/S,S$_{mp}$*)-**S33** (in a 0.36 : 0.64 diastereoisomeric ratio by ^{1}H NMR, **Figure S188**) was purified by chromatography (*1st column:* petrol with 0→100% CH_2Cl_2, followed by 0→10% EtOH; *2nd column:* petrol with 0→100% CH_2Cl_2, followed by 0→20% MeCN), to yield the product as a yellow oil (9.9 mg, 64%, 0.36 : 0.64 diastereoisomeric ratio, **Figure S189**); ^{1}H NMR (500 MHz, $CDCl_3$, 298 K) δ 10.26 (s, 1H, H_h (*minor*)), 10.12 (s, 1H, H_h (*major*)), 8.00-7.92 (m, 2H, H_i), 7.77-7.70 (m, 2H, H_B and H_S), 7.59-7.52 (m, 2H, H_A and H_T), 7.49-7.44 (m, 1H, H_C or H_R), 7.42-7.37 (m, 1H, one of $H_{e'}$), 7.37-3.30 (m, 2H, one of $H_{e'}$ and H_C or H_R), 7.22-7.17 (m, 2H, H_D), 7.15-7.03 (m, 6H, H_f, H_g $H_{f'}$ and H_j), 6.66 (d, 2H, *J* = 7.4, H_e (*major*)), 6.55 (d, 2H, *J* = 7.4, H_e (*minor*)), 6.43-6.38 (m, 2H, H_N), 6.34-6.28 (m, 2H, $H_{e'}$), 6.27-6.23 (m, 2H, H_M), 6.19 (d, 2H, *J* = 8.8, H_E), 4.87-4.81 (m, 1H, H_b), 4.59-4.50 (m, 1H, one of H_F) 7.44-4.36 (m, 1H, one of H_F), 4.32-4.25 (m, 1H, one of H_Q), 4.15-4.10 (m, 1H, one of H_Q), 4.05-3.88 (m, 4H, H_d and $H_{d'}$), 3.33-3.26 (m, 1H), 3.04-2.97 (m, 1H, one of H_O), 2.91-2.88 (m, 1H, one of H_O), 2.94-2.86 (m, 2H, H_G), 2.84-2.76 (m, 1H, one of H_K), 2.74-2.70 (m, 1H, one of H_K), 2.59-2.21 (m, 8H, m, 8H, H_H, H_I, H_J, and H_P), 1.28-1.22 (m, 18H, H_k), 1.11 (d, 3H, *J* = 5.8, three of H_a (*major*)), 1.05 (d, 3H, *J* = 5.8, three of H_a (*major*)), 1.00-0.94 (m, 3H, three of H_a (*minor*));^{13}C NMR (126 MHz, CDCl3) δ 168.5, 168.5, 159.2, 159.0, 157.7, 157.1, 157.1, 149.7, 149.7, 149.7, 149.6, 147.3, 146.8, 146.5, 146.0, 137.3, 137.2, 137.2, 137.1, 135.4, 135.4, 134.8, 134.5, 132.7, 132.5, 131.5, 131.4, 131.4, 131.4, 131.3, 131.0, 130.8, 130.6, 130.5, 129.5, 129.5, 129.2, 128.7, 128.5, 128.5, 128.0, 127.9, 126.8, 126.7, 125.2, 124.6, 124.6, 124.5, 124.4, 124.0, 124.0, 123.9, 123.8, 123.4, 122.4, 120.7, 120.2, 120.1, 120.0, 119.5, 119.0, 114.7, 114.7, 114.6, 114.6, 114.5, 70.6, 70.5, 69.9, 69.9, 68.2, 68.1, 66.6, 66.0, 45.9, 43.2, 42.0, 39.9, 37.5, 37.3, 35.2, 32.4, 32.1, 31.7, 31.1, 29.8, 29.5, 29.2, 28.9, 28.6, 27.6, 26.5, 25.9, 25.8, 25.5, 25.0, 23.6, 22.8, 21.6, 21.5, 21.4, 14.3; LR-ESI-MS (+ve) *m/z* (%) = 1096.5 $[M+H]^+$ (100).

‡ For comparison with rotaxane (*S,S$_{mp}$*)-**4**, no proton is designated as H_c. $H_{e'}$ and $H_{f'}$ are to distinguish between the protons on the *p*-bromobenzyl group and the benzyl protons (H_e and H_f).

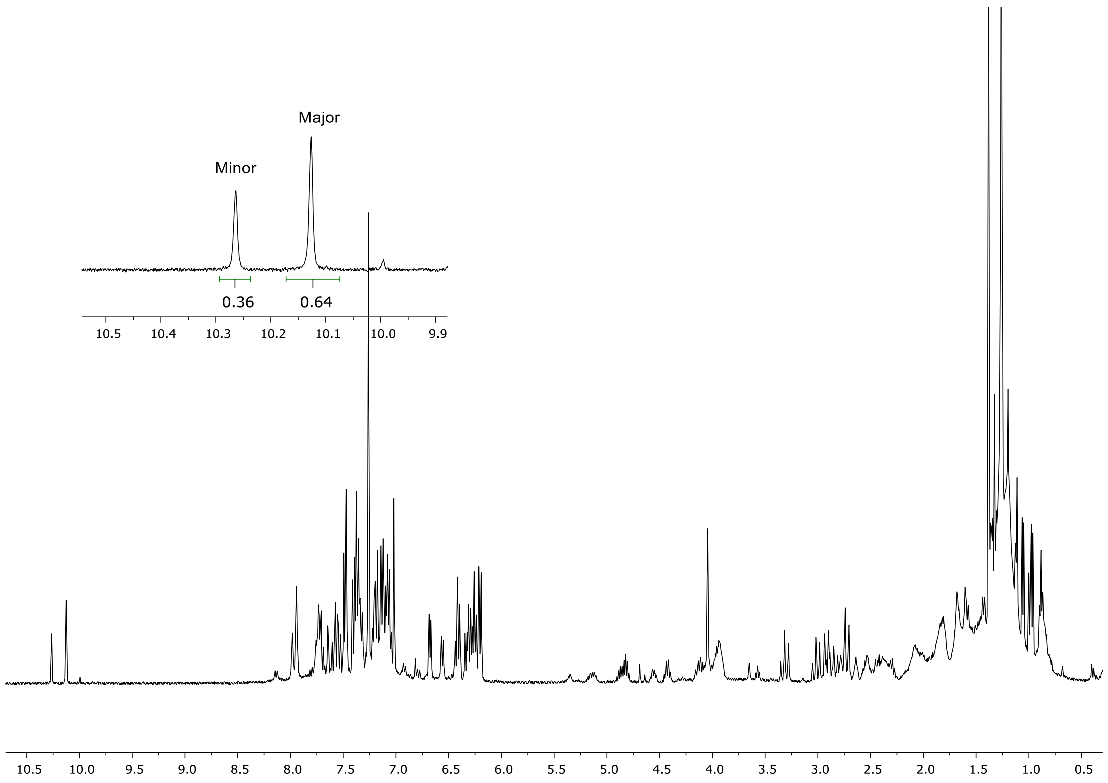

Figure S188. ^{1}H NMR (400 MHz, $CDCl_3$, 298 K) (*R/S,S$_{mp}$*)-**S33** prior to purification by chromatography.

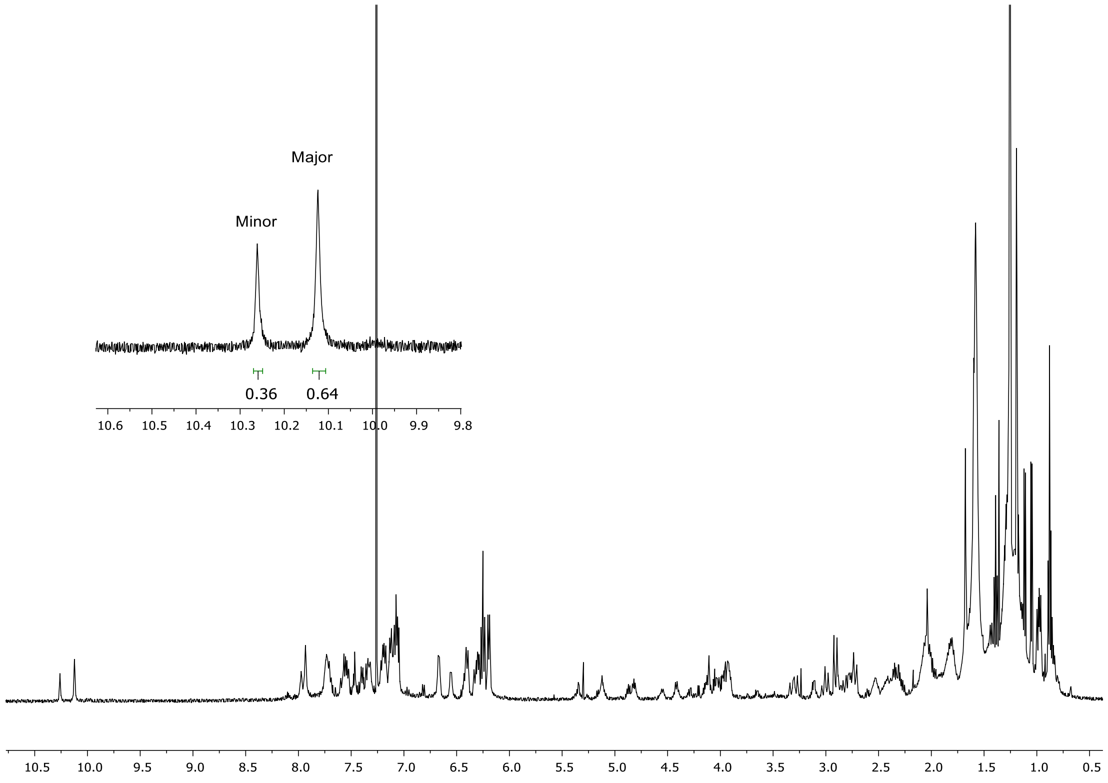

Figure S189. ^{1}H NMR (500 MHz, $CDCl_3$, 298 K) (*R/S,S$_{mp}$*)-**S33**, following purification by chromatography.

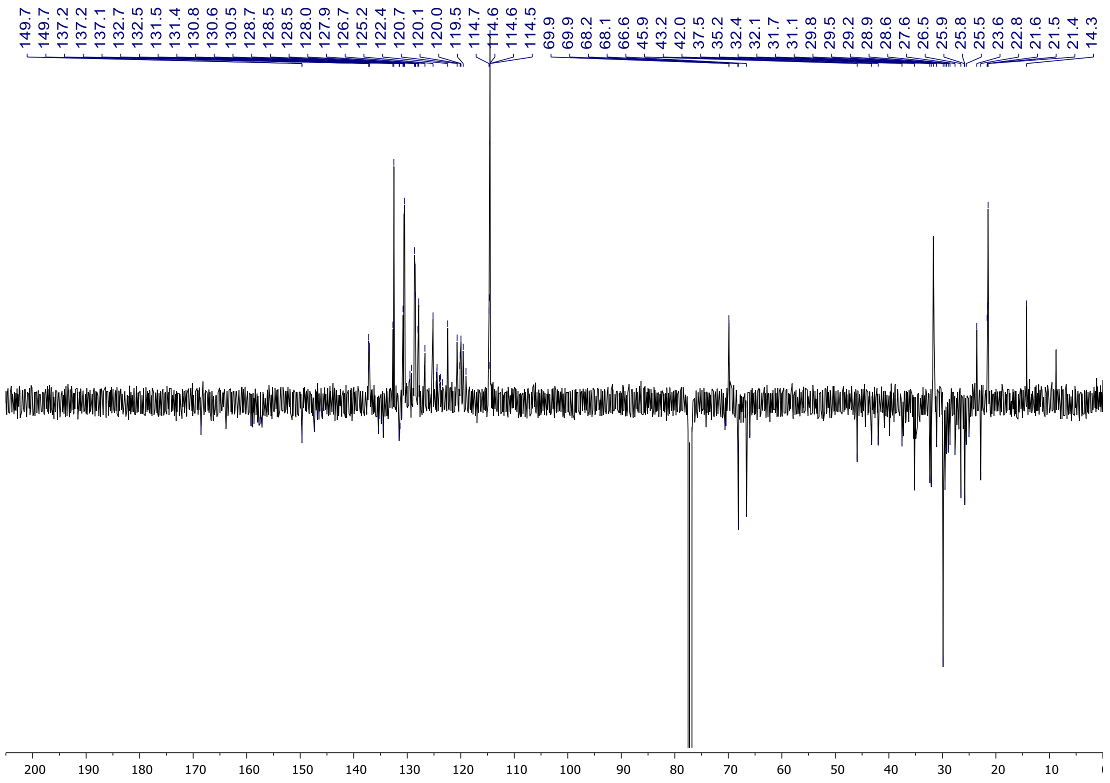

Figure S190. ^{13}C NMR (126 MHz, $CDCl_3$, 298 K) (*R/S,S$_{mp}$*)-**S33**, following purification by chromatography.

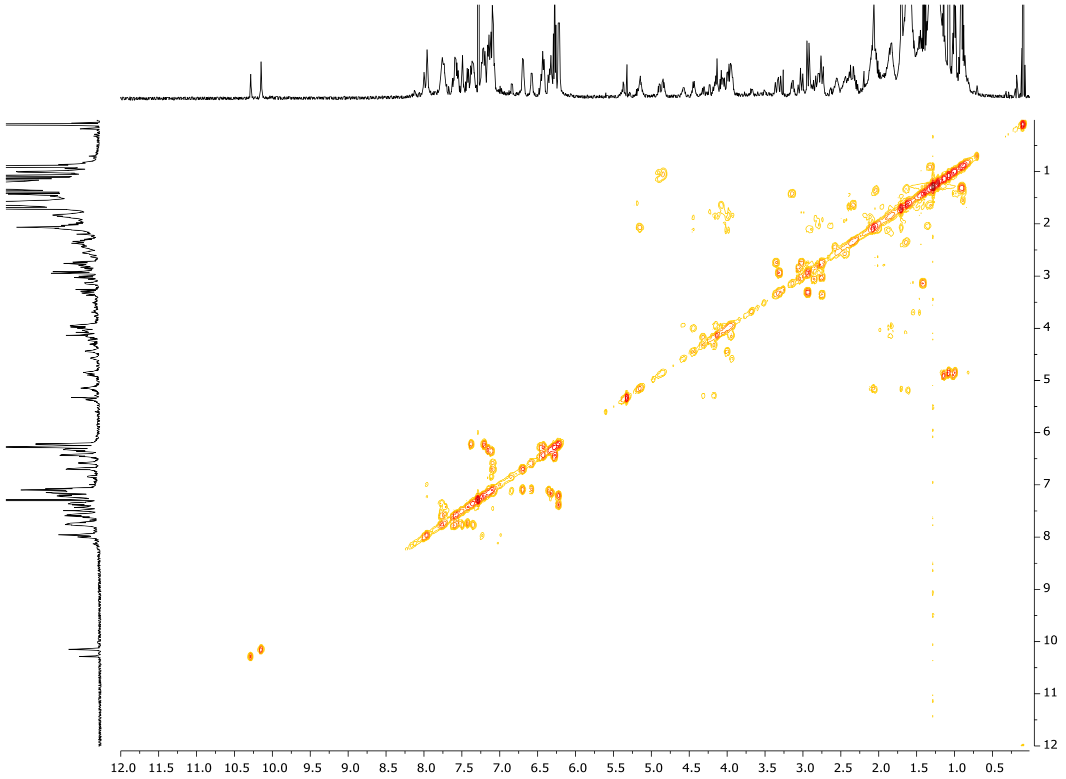

Figure S191. ^{1}H-^{1}H COSY NMR (126 MHz, $CDCl_3$, 298 K) (*R/S,S$_{mp}$*)-**S33**, following purification by chromatography.

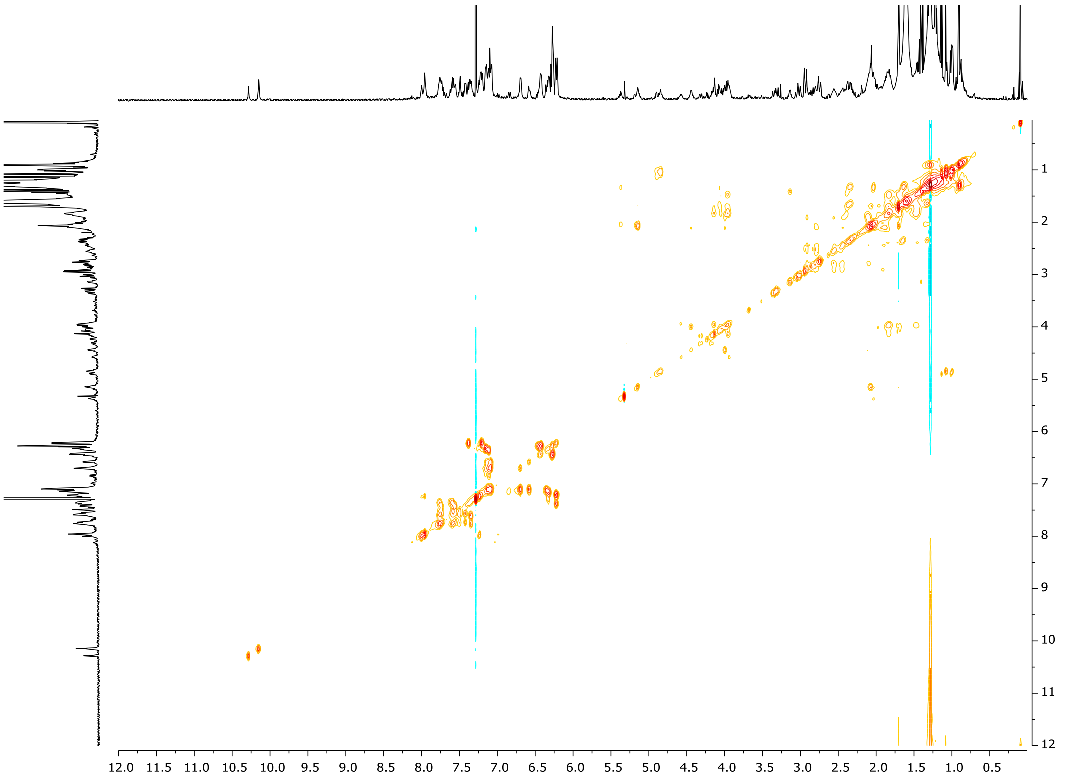

Figure S192. ^{1}H-^{1}H TOCSY NMR (126 MHz, $CDCl_3$, 298 K) (*R/S,S*$_{mp}$)-**S33**, following purification by chromatography.

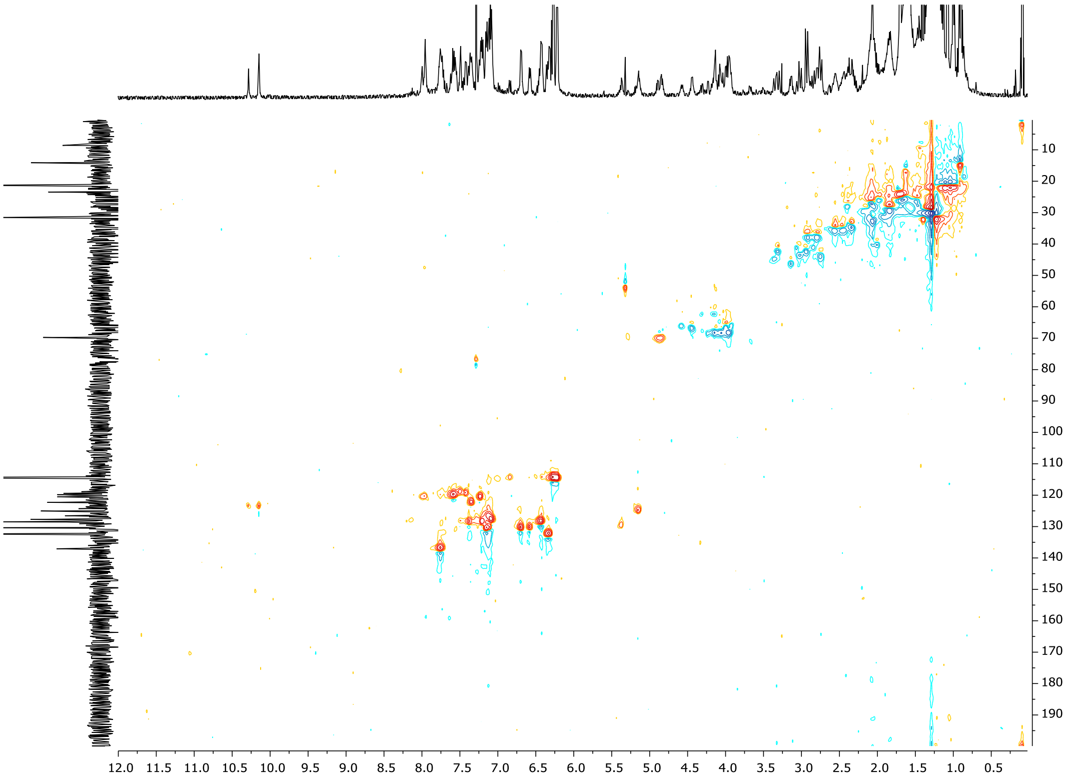

Figure S193. ^{1}H-^{13}C HSQC NMR (126 MHz, $CDCl_3$, 298 K) (*R/S,S*$_{mp}$)-**S33**, following purification by chromatography.

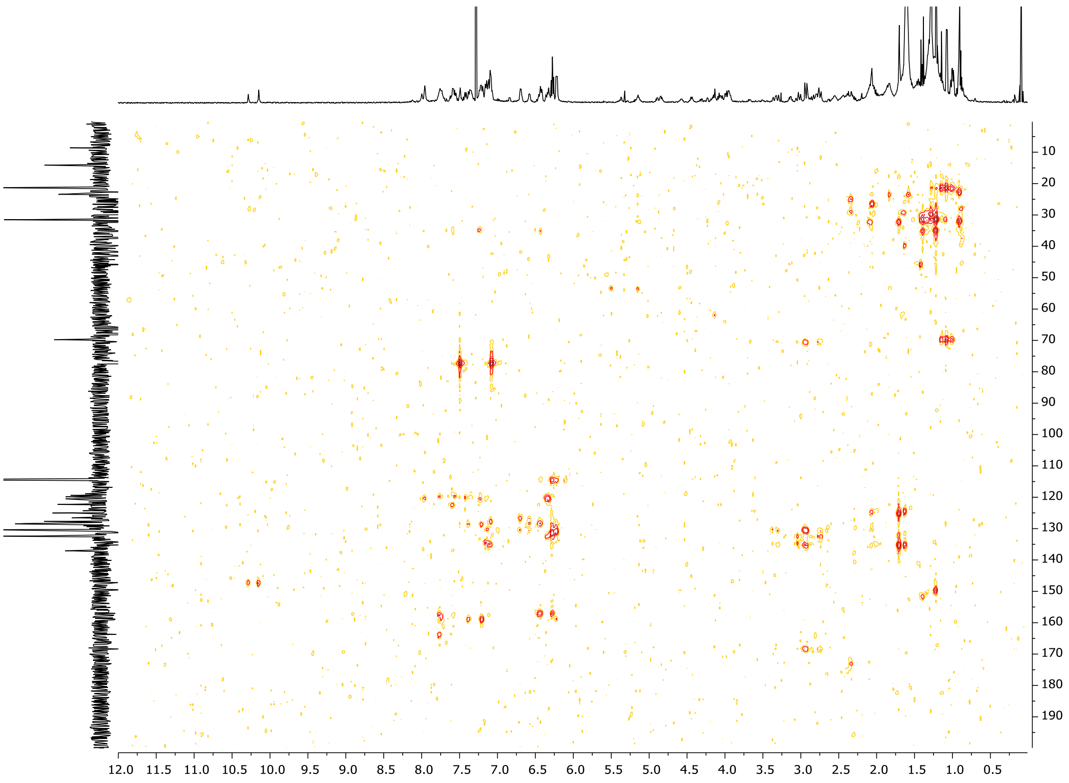

Figure S194. ^{1}H-^{13}C HMBC NMR (126 MHz, $CDCl_3$, 298 K) (*R/S,S$_{mp}$*)-**S33**, following purification by chromatography.

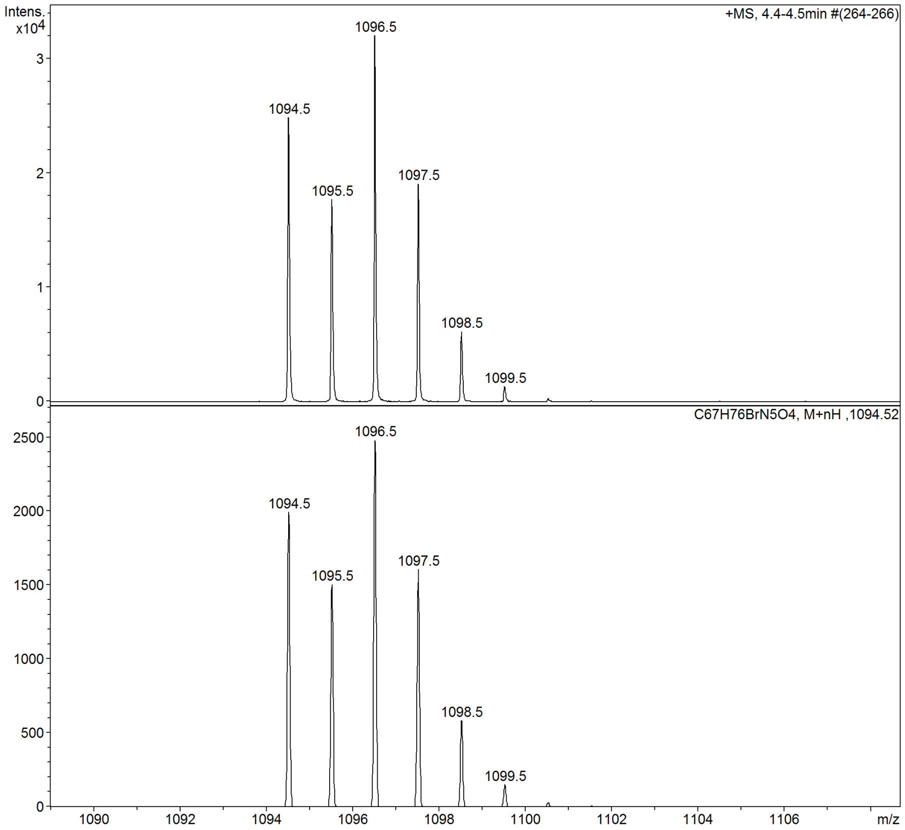

Figure S195. ESI-MS isotopic pattern of (*R/S,S$_{mp}$*)-**S33**; observed (top) and calculated (bottom).

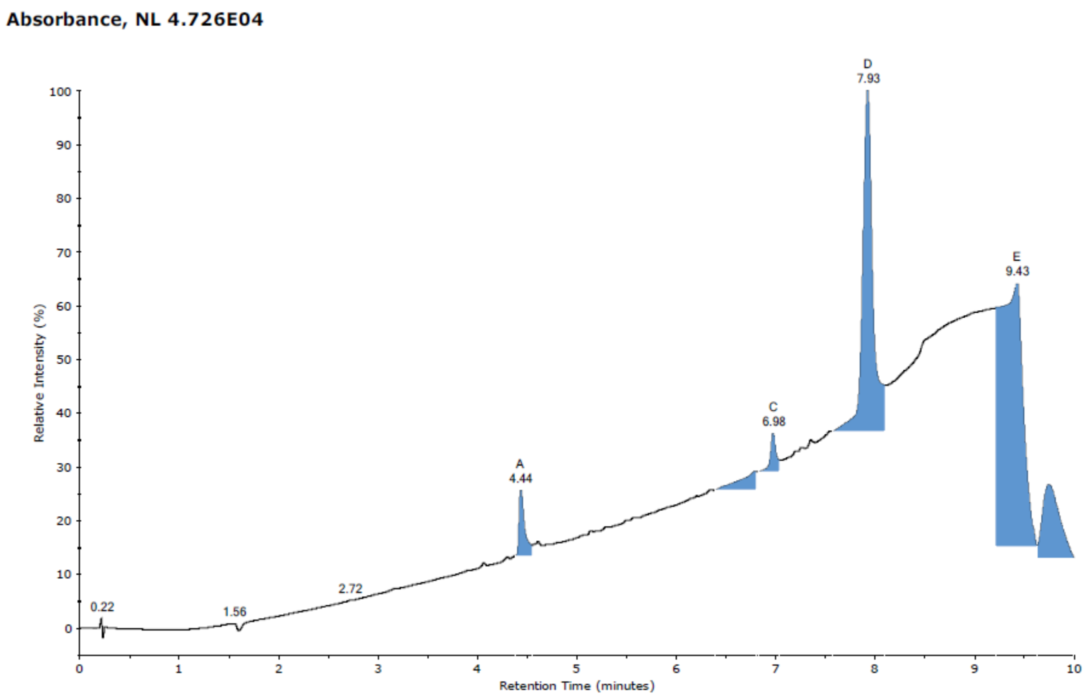

Figure S196. LCMS trace (C18 column, gradient 5 minutes (1 : 4 MeCN+0.2% formic acid-H_2O +0.2% formic acid →1 : 0 MeCN-H_2O +0.2% formic acid), UV 254 nm), of (D,R$_{mp}$/S_{mp})-**S13** following purification by chromatography.

11. Preliminary Molecular Modelling

Based on previous work,[19] the AT-CuAAC reaction of macrocycle **1**, acetylene **2a** and azide **3e** is thought to proceed *via* Cu^{I}-acetylides **I** and **II** which are irreversibly converted to Cu^{I}-triazolides **III** and **IV** and ultimately, after protolytic work-up, to rotaxanes (S,S_{mp})-**4** (major) and (S,R_{mp})-**4** respectively (Scheme S1). Based on this proposed mechanism, two obvious sources of diastereoselectivity can be identified; i) a significant energy difference between **I** and **II** which results in a biased pre-equilibrium (Cu^{I} acetylide formation can be expected to be reversible in the presence of N^{i}PrEt$_2$) prior to irreversible covalent bond formation; ii) a significant difference in reaction rate for the conversion of **I→III** and **II→IV**.

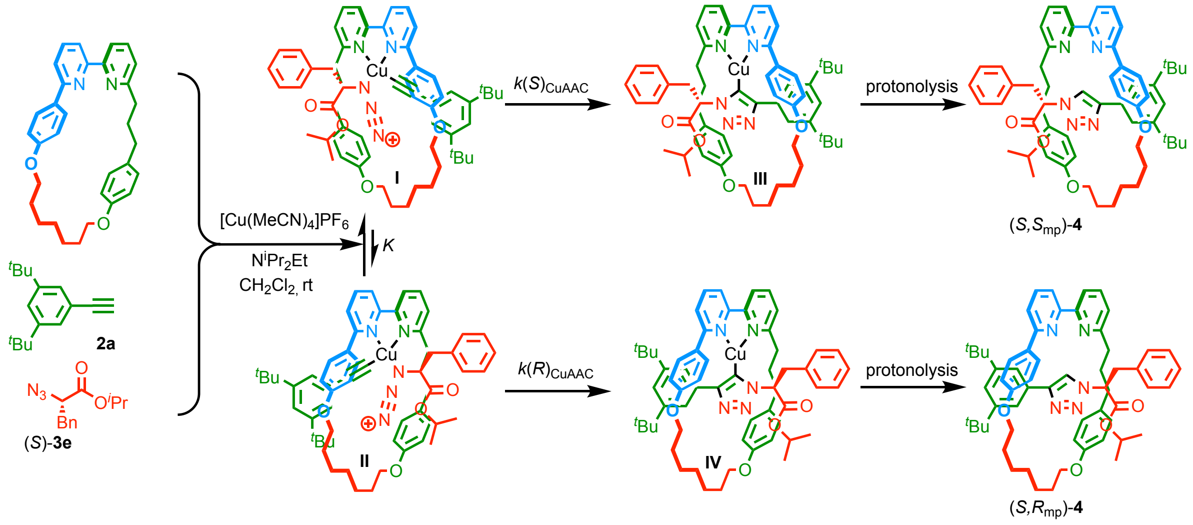

Scheme S1. Schematic mechanism of the AT-CuAAC reaction *via* key acetylide and triazolide intermediates

To probe the origin of stereoselectivity in this AT-CuAAC reaction we carried out preliminary calculations to determine the relative energies of **I**/**II** and of the reaction ΔG **I→III** and **II→IV**. It should be noted that, given the controversial nature of the cycloaddition mechanism and the size of the molecules concerned, accurately identifying the transition states energies for the cycloaddition step lies beyond the scope of this preliminary study. However, linear-free energy relationship considerations suggest that reactions proceeding *via* the same pathway but with a larger ΔG of reaction can be expected to have a lower reaction barrier, although the difference in reaction energies is likely to be significantly larger than the difference in activation energies as the reaction is predicted to have an early barrier (Hammond postulate). Thus, although these calculations cannot be used to derive the difference in reaction rates for **I→III** and **II→IV**, the comparison of the reaction energies **I→III** and **II→IV** gives an indication of whether a difference in reaction rates is to be expected.

Table S3. Computed energies of molecular models of the AT-CuAAC reaction intermediates of macrocycle **1**, actylene **2a** and azide **3e**.[a]

Entry	Acetylide intermediate		Triazolide intermediate
1	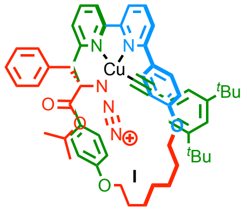	ΔG = –271.1 kJmol^{-1}	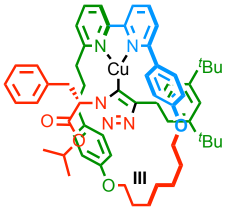
	285.5 kJmol^{-1}		14.4 kJmol^{-1}
2	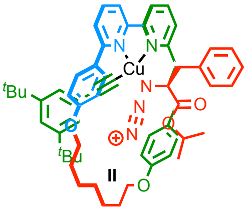	ΔG = –269.0 kJmol^{-1}	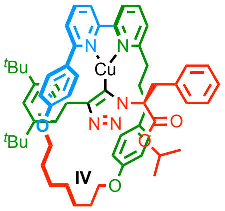
	289.6 kJmol^{-1}		20.6 kJmol^{-1}
ΔE =	4.1 kJmol^{-1}	2.0 kJmol^{-1}	6.2 kJmol^{-1}

[a] Modelling was carried out using Spartan '10 (Wavefunction). Molecular models (Figures S197-S200) of the intermediates (Cu^{I}-acetylide and -triazolide) were prepared and subjected a conformer distribution (MMFF) search. The lowest energy conformation was selected and the energy minimised (PM6, gas phase). The energies obtained (Table S3) were compared.

Based on the results in Table S3 the origin of the stereoselectivity is expected to be a combination of a biased pre-equilibrium *and* the difference in reaction rates of the cycloaddition step, which is perhaps unsurprising. Furthermore, comparison of the models of **I** and **II** and **III** and **IV** reveals that, as might be expected, the stereoselectivity seems to be driven by avoiding steric clash between the more rigid aryl-pyridine motif and the benzyl group of the azide component.

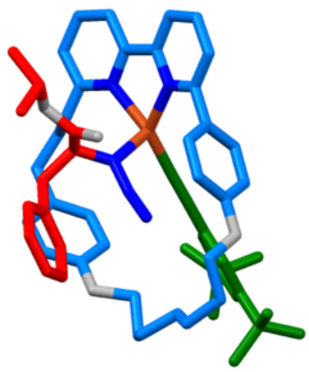

Figure S197. PM6 Model of Intermediate **I** (Scheme S1)

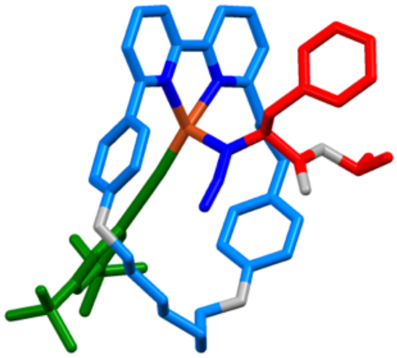

Figure S198. PM6 Model of Intermediate **II** (Scheme S1)

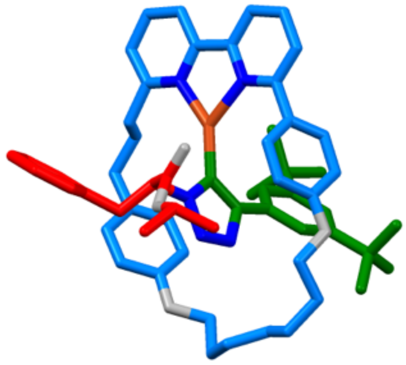

Figure S199. PM6 Model of Intermediate **III** (Scheme S1)

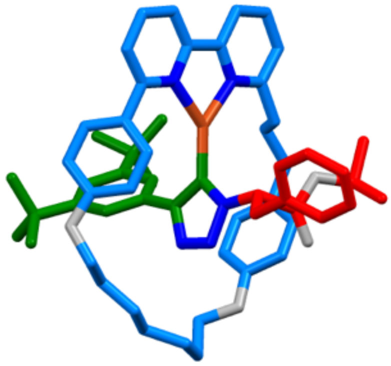

Figure S200. PM6 Model of Intermediate **IV** (Scheme S1)

To examine the reaction further, we modelled the same intermediates (**V-VIII**) for the reaction macrocycle **1**, azide **3e** and phenyl acetylene (Table S4); our experimental results (Table 1, entry 11) suggest that the *m*-^{t}Bu groups of alkyne **2a** are important for high stereoselectivity.

Table S4. Computed energies of molecular models of the AT-CuAAC reaction intermediates of macrocycle **1**, azide **3e** and phenyl acetylene.[a]

Entry	Acetylide intermediate		Triazolide intermediate
1	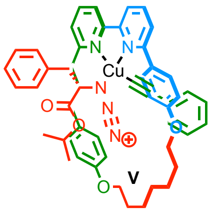 487.9 kJmol^{-1}	ΔG = –269.6 kJmol^{-1} →	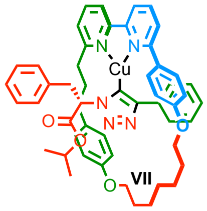 218.4 kJmol^{-1}
2	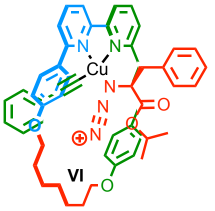 487.8 kJmol^{-1}	ΔG = –264.6 kJmol^{-1} →	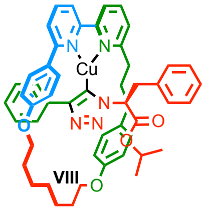 223.2 kJmol^{-1}
ΔE =	−0.1 kJmol^{-1}	5.0 kJmol^{-1}	4.8 kJmol^{-1}

[a] Modelling was carried out using Spartan '10 (Wavefunction). Molecular models (Figures S201-S204) of the intermediates (Cu^{I}-acetylide and -triazolide) were prepared and subjected a conformer distribution (MMFF) search. The lowest energy conformation was selected and the energy minimised (PM6, gas phase).

Pleasingly, the results in Table S5 are in broad agreement with experiment in that the pre-equilbrium of acetylides derived from phenylacetylene is predicted to be essentially unbiased removing this source of stereoselectivity, whereas a significant difference in reaction free energy is predicted. Thus, the modelling predicts that the stereoselectivity in this reaction is determined by the difference in reaction rates in the reactions **V→VII** and **VI→VIII** without the benefit of a biased equilibrium between **V** and **VI**.

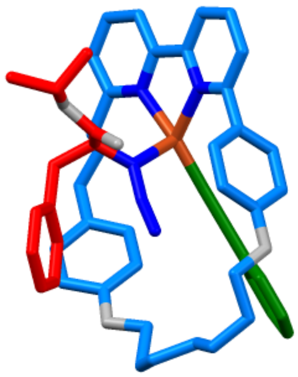

Figure S201. PM6 Model of Intermediate **V** (Table S4)

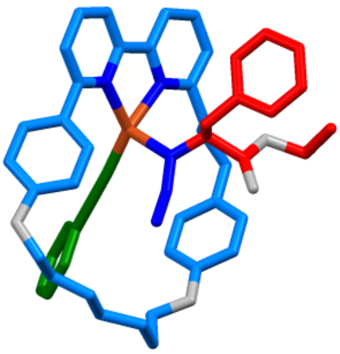

Figure S202. PM6 Model of Intermediate **VI** (Table S4)

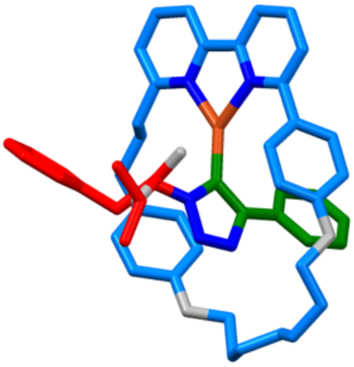

Figure S203. PM6 Model of Intermediate **VII** (Table S4)

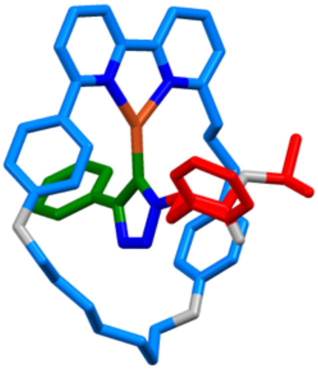

Figure S204. PM6 Model of Intermediate **VIII** (Table S4)

The results of this preliminary molecular modelling suggest that it may be possible to qualitatively predict the stereochemical efficiency of a directing group by identifying candidates that produce a biased pre-equilbrium of Cu^{I}-acetylides. However, further comparisons of experiment and theory are required before this can be confirmed.

12. References

[1] A. Pigorsch, M. Köckerling, *Cryst. Growth Des.* **2016**, *16*, 4240–4246.

[2] G. T. Potter, G. C. Jayson, G. J. Miller, J. M. Gardiner, *J. Org. Chem.* **2016**, *81*, 3443–3446.

[3] J. E. M. Lewis, R. J. Bordoli, M. Denis, C. J. Fletcher, M. Galli, E. A. Neal, E. M. Rochette, S. M. Goldup, *Chem. Sci.* **2016**, *7*, 3154–3161.

[4] S. Ogi, T. Ikeda, R. Wakabayashi, S. Shinkai, M. Takeuchi, *Chem. - A Eur. J.* **2010**, *16*, 8285–8290.

[5] R. S. Nandurdikar, A. V. Subrahmanyam, K. P. Kaliappan, *European J. Org. Chem.* **2010**, *2010*, 2788–2799.

[6] M. Juríček, M. Felici, P. Contreras-Carballada, J. Lauko, S. R. Bou, P. H. J. Kouwer, A. M. Brouwer, A. E. Rowan, *J. Mater. Chem.* **2011**, *21*, 2104–2111.

[7] Q. Liu, M. Li, R. Xiong, F. Mo, *Org. Lett.* **2017**, *19*, 6756–6759.

[8] J. Hornung, D. Fankhauser, L. D. Shirtcliff, A. Praetorius, W. B. Schweizer, F. Diederich, *Chem. - A Eur. J.* **2011**, *17*, 12362–12371.

[9] S. Eising, F. Lelivelt, K. M. Bonger, *Angew. Chemie Int. Ed.* **2016**, *55*, 12243–12247.

[10] S. Grunder, D. Muñoz Torres, C. Marquardt, A. Błaszczyk, R. Krupke, M. Mayor, *European J. Org. Chem.* **2011**, *2011*, 478–496.

[11] B. H. Lipshutz, M. Hageman, J. C. Fennewald, R. Linstadt, E. Slack, K. Voigtritter, *Chem. Commun.* **2014**, *50*, 11378–11381.

[12] A. Isidro-Llobet, K. Hadje Georgiou, W. R. J. D. Galloway, E. Giacomini, M. R. Hansen, G. Méndez-Abt, Y. S. Tan, L. Carro, H. F. Sore, D. R. Spring, *Org. Biomol. Chem.* **2015**, *13*, 4570–4580.

[13] Z. Zhang, J. Tian, H. Xiao, N. Zheng, X. Gao, R. Zhu, H. Xiao, *J. Chromatogr. B* **2016**, *1033–1034*, 382–389.

[14] E. E. Ricks, M. C. Estrada-Valdes, T. L. McLean, G. A. Iacobucci, *Biotechnol. Prog.* **1992**, *8*, 197–203.

[15] J. A. Hickin, A. Ahmed, K. Fucke, M. Ashcroft, K. Jones, *Chem. Commun.* **2014**, *50*, 1238–1240.

[16] J. Chen, J.-H. Lin, J.-C. Xiao, *Org. Lett.* **2018**, *20*, 3061–3064.

[17] M. Kitamura, S. Kato, M. Yano, N. Tashiro, Y. Shiratake, M. Sando, T. Okauchi, *Org. Biomol. Chem.* **2014**, *12*, 4397.

[18] G. M. Sheldrick, *Acta Crystallogr. Sect. C Struct. Chem.* **2015**, *71*, 3–8.

[19] E. A. Neal, S. M. Goldup, *Chem. Sci.* **2015**, *6*, 2398. E. A. Neal, S. M. Goldup, *Angew. Chem. Int. Ed.* **2016**, *55*, 12488.
